# Supplementary material for: Clonal chromosomal mosaicism and loss of chromosome Y in elderly men increase vulnerability for SARS-CoV-2
Source: Commun Biol. 2024 Feb 19;7:202. doi: 10.1038/s42003-024-05805-6 (PMC10876565; doi:10.1038/s42003-024-05805-6)
Supplement: Supplementary file 2 — Supplementary Material [file 42003_2024_5805_MOESM2_ESM.pdf]

Clonal chromosomal mosaicism and loss of chromosome Y in men are risk factors for SARS-CoV-2 vulnerability in the elderly

Supplementary material

Luis A. Pérez-Jurado; Alejandro Cáceres; Laura Balagué-Dobón; Tonu Esko; Miguel López de Heredia; Inés Quintela; Raquel Cruz; Pablo Lapunzina; Ángel Carracedo; SCOURGE Cohort Group; Juan R. González

Contents

Supplementary Data 1-6 **1**

Supplementary Tables S1-S8 **3**

Supplementary Figures S1-S3 **10**

Supplementary Data

Supplementary Data 1: Patients with mCAs. In each case, detected mCAs are indicated by chromosome (chr), start and end coordinates of the event, estimated proportion of cells carrying the mCA (cellularity) and type of event (gain, loss, or copy neutral loss of heterozygosity – cnloh). Patient age, COVID-19 severity, and patient status (dead or alive) 90 days after disease are shown in the three last columns. NA: data not available.
[See Supplementary Data Excel File]

Supplementary Data 2: Male patients with XCM due to LOY. In addition to the patient identifier (ID) and patient age at diagnosis of COVID-19, columns show the estimated proportion of cells with XCM (LOY proportion), COVID-19 severity, and patient status (dead or alive) 90 days after disease. NA: data not available.
[See Supplementary Data Excel File]

Supplementary Data 3: Individuals with germline (non-mosaic) complete aneuploidies (chromosome 21 and gonosomes), and developmental mosaic X-chromosome aneuploidies.
[See Supplementary Data Excel File]

Supplementary Data 4: Y-linked genes with homologs in the X-chromosome and a possible role in immunity.
[See Supplementary Data Excel File]

Supplementary Data 5: Y-linked genes with homologs in the X-chromosome and no known role in immunity.
[See Supplementary Data Excel File]

Supplementary Data 6: Top 30 differentially expressed genes in blood of individuals with mCAs.
[See Supplementary Data Excel File]

Supplementary Tables

Table S1: Comparison of analytical parameters between age-matched individuals with and without LOY in the EGCUT biobank.

	normal N=502	LOY N=28	p.overall
WBC	6.18 [5.24;7.27]	6.49 [5.25;7.65]	0.443
RBC	5.07 [4.82;5.30]	4.72 [4.50;5.13]	0.004
PLT	217 [190;248]	239 [219;270]	0.015
EO%	2.30 [1.50;3.55]	2.60 [1.60;4.00]	0.426
BASO%	0.40 [0.30;0.60]	0.30 [0.20;0.43]	0.027
MONO%	8.50 [7.00;10.1]	8.70 [7.38;9.62]	0.720
LYMPH%	30.6 (7.53)	27.6 (8.83)	0.088
NEUT%	57.4 (8.73)	59.8 (9.38)	0.184
EO (total)	0.15 [0.09;0.23]	0.15 [0.11;0.28]	0.300
BASO (total)	0.03 [0.02;0.04]	0.02 [0.02;0.02]	0.016
MONO (total)	0.52 [0.43;0.65]	0.58 [0.46;0.75]	0.146
LYMPH (total)	1.83 [1.53;2.28]	1.66 [1.34;2.14]	0.179
NEUT (total)	3.46 [2.81;4.39]	3.99 [2.77;4.90]	0.247
RDW-SD	40.4 [38.9;42.4]	42.5 [41.5;46.0]	0.007
HGB	152 [146;159]	151 [138;156]	0.401
PDW	13.1 [11.8;14.4]	13.6 [12.2;15.2]	0.596
MPV	10.9 [10.2;11.5]	11.1 [10.5;11.6]	0.523
LCR	31.8 [26.7;37.2]	32.4 [29.0;38.5]	0.587
PCT	0.24 [0.21;0.27]	0.25 [0.24;0.27]	0.096
Hb	151 (14.5)	147 (13.2)	0.247
Hct	45.0 [43.0;47.0]	44.0 [42.0;47.0]	0.454
MCV	88.0 [85.6;91.2]	90.6 [89.0;94.6]	<0.001
MCH	30.1 [29.2;30.9]	30.2 [29.5;30.9]	0.384
MCHC	340 [335;346]	332 [325;337]	<0.001
RDW-CV	12.7 [12.3;13.2]	13.1 [12.7;13.4]	0.006
Alb	47.0 [45.0;49.0]	44.0 [42.0;45.2]	<0.001
ALAT	23.0 [17.0;32.8]	25.0 [15.5;36.2]	0.615
ALP	66.0 [56.0;80.8]	73.5 [59.5;81.5]	0.156
ASAT	24.0 [20.0;28.0]	24.0 [18.0;30.2]	0.778
Bil	9.00 [6.00;12.0]	8.00 [7.00;13.2]	0.951
GGT	21.0 [15.0;31.0]	22.5 [15.8;30.0]	0.738
Hcy	10.6 [8.43;13.6]	14.8 [11.6;18.3]	<0.001
Chol	5.20 [4.40;6.10]	5.20 [4.80;5.98]	0.396
HDL-Chol	1.31 [1.09;1.55]	1.39 [1.12;1.76]	0.135
LDL-Chol	3.33 [2.71;4.25]	3.25 [2.93;3.73]	0.912
Trigl	1.41 [0.98;2.13]	1.22 [1.01;1.67]	0.120
Crea	77.0 [70.0;86.0]	81.5 [69.8;89.2]	0.253
UA	335 [295;383]	350 [325;388]	0.182
Urea	5.40 [4.50;6.40]	6.15 [5.20;7.25]	0.002
Fer	107 [59.0;179]	103 [54.8;199]	0.840
Fe	18.1 [14.1;23.0]	19.2 [15.0;23.7]	0.591
Transf	2.62 [2.38;2.92]	2.54 [2.32;3.02]	0.711
Transf-sR	2.90 [2.40;3.50]	2.90 [2.48;3.60]	0.928
Fol	15.9 [12.8;20.3]	15.8 [13.8;19.9]	0.850
B12	301 [232;378]	262 [226;312]	0.067
TSH	1.07 [0.75;1.53]	1.49 [1.09;1.85]	0.007
CRP	1.57 [0.97;3.04]	2.23 [0.87;3.11]	0.697
Gluc	5.40 [5.00;6.00]	5.30 [4.80;5.90]	0.659
CysC	1.04 [0.92;1.14]	1.11 [1.06;1.13]	0.050
EPO	9.59 [7.24;11.8]	9.56 [7.40;10.4]	0.836
Testo	15.2 [10.2;20.7]	14.4 [10.4;16.7]	0.585

continued on next page

Table S1 – *continued from previous page*

	normal N=502	LOY N=28	p.overall
Ins	7.80 [4.80;15.0]	9.60 [5.20;13.7]	0.797

Table S2: Top 30 differentially expressed genes in blood of individuals with LOY

logFC	CI.L	CI.R	AveExpr	t	P.Value	adj.P.Val	B	SE	Chromosome	Symbol
3	2.1	3.8	-0.32	7.1	4.6×10^{-9}	0.00022	5.3	0.23	12	VWF
-2.5	-3.3	-1.7	-0.24	-6.4	5.7×10^{-8}	0.0014	4	0.15	Y	CSF2RA
2.3	1.5	3.1	0.25	5.5	1.3×10^{-6}	0.021	2.3	0.26	20	MYL9
-2.2	-3	-1.3	-0.22	-5.4	2.3×10^{-6}	0.028	2	0.21	Y	CSF2RA
0.71	0.43	0.98	-0.017	5.2	3.9×10^{-6}	0.038	1.7	0.17	2	SPC25
1.3	0.74	1.8	0.17	4.9	1.2×10^{-5}	0.099	1	0.18	X	BEND2
2.5	1.4	3.5	0.089	4.7	2.1×10^{-5}	0.14	0.72	0.18	17	ITGA2B
3.3	1.9	4.8	0.11	4.7	2.4×10^{-5}	0.14	0.64	0.29	4	PPBP
1.1	0.6	1.5	-0.18	4.6	2.6×10^{-5}	0.14	0.6	0.19	14	SYNE3
0.71	0.39	1	-0.062	4.5	4.4×10^{-5}	0.18	0.29	0.19	12	ANKS1B
-1	-1.5	-0.55	0.02	-4.5	4.5×10^{-5}	0.18	0.28	0.23	11	POU2AF1
0.69	0.37	1	-0.11	4.4	6.8×10^{-5}	0.26	0.041	0.28	X	GAGE4
-1.6	-2.4	-0.87	-0.034	-4.3	8.2×10^{-5}	0.26	-0.07	0.2	17	KRT23
-0.8	-1.2	-0.43	0.032	-4.3	8.3×10^{-5}	0.26	-0.073	0.2	8	ENPP2
0.75	0.4	1.1	-0.14	4.3	8.9×10^{-5}	0.26	-0.11	0.22	16	CDH8
0.79	0.42	1.2	-0.056	4.3	9.2×10^{-5}	0.26	-0.14	0.18	15	TMOD3
2.3	1.2	3.4	0.04	4.2	0.0001	0.27	-0.21	0.37	11	JAM3
-0.92	-1.4	-0.47	-0.12	-4.1	0.00017	0.41	-0.49	0.19	Y	SFRS17A
-1	-1.5	-0.52	-0.1	-4	0.00019	0.44	-0.56	0.17	2	WIPF1
1.7	0.83	2.5	0.34	4	0.00023	0.48	-0.68	0.3	18	GTSCR1
1.1	0.55	1.7	-0.14	4	0.00024	0.48	-0.71	0.22	1	ADAM15
-1.4	-2.1	-0.7	0.25	-4	0.00024	0.48	-0.71	0.24	Y	TMSB4Y
1.5	0.75	2.3	0.054	4	0.00025	0.48	-0.71	0.2	4	GUCY1A3
0.78	0.38	1.2	-0.033	3.9	0.00026	0.5	-0.76	0.22	5	PIK3R1
0.87	0.42	1.3	0.035	3.9	0.00029	0.5	-0.81	0.22	17	MEOX1
1	0.5	1.6	0.012	3.9	0.00031	0.5	-0.85	0.19	15	ACSBG1
0.92	0.44	1.4	-0.04	3.8	0.00035	0.5	-0.92	0.28	19	ZNF266
2.6	1.3	4	-0.73	3.8	0.00036	0.5	-0.94	0.17	8	DEFCR24
0.55	0.26	0.84	0.038	3.8	0.00037	0.5	-0.96	0.17	10	CYP26A1
-0.73	-1.1	-0.34	0.13	-3.8	0.00041	0.5	-1	0.2	16	TAOK2

Table S3: Top 30 differentially expressed Y-linked genes in blood of individuals with LOY

logFC	CI.L	CI.R	AveExpr	t	P.Value	adj.P.Val	B	SE	Chromosome	Symbol
-2.5	-3.3	-1.7	-0.24	-6.4	5.7×10^{-8}	0.0014	4	0.15	Y	CSF2RA
-2.2	-3	-1.3	-0.22	-5.4	2.3×10^{-6}	0.028	2	0.21	Y	CSF2RA
-0.92	-1.4	-0.47	-0.12	-4.1	0.00017	0.41	-0.49	0.19	Y	SFRS17A
-1.4	-2.1	-0.7	0.25	-4	0.00024	0.48	-0.71	0.24	Y	TMSB4Y
-2.3	-3.5	-1.1	0.11	-3.7	0.00049	0.5	-1.1	0.2	Y	EIF1AY
-5.2	-8	-2.3	1.1	-3.6	0.00074	0.53	-1.4	0.18	Y	EIF1AY
-0.8	-1.3	-0.31	0.036	-3.3	0.0018	0.69	-1.9	0.21	XY	ASMTL
-0.89	-1.5	-0.33	0.079	-3.2	0.0024	0.77	-2.1	0.2	Y	TLNGY
-0.66	-1.1	-0.22	-0.044	-3	0.0041	0.89	-2.4	0.25	Y	BCORL2
-1.9	-3.3	-0.53	0.43	-2.8	0.0074	0.99	-2.7	0.17	Y	KDM5D
-0.88	-1.5	-0.24	-0.15	-2.8	0.0076	0.99	-2.7	0.24	Y	SFRS17A
-1.7	-3	-0.46	0.48	-2.7	0.0087	1	-2.8	0.19	Y	TXLNGY
-5.5	-9.7	-1.3	2	-2.6	0.011	1	-3	0.21	Y	RPS4Y1
-1.7	-3.1	-0.38	0.2	-2.6	0.013	1	-3	0.36	Y	RPS4Y2
-0.75	-1.3	-0.17	-0.086	-2.6	0.013	1	-3	0.15	Y	ZBED1
-0.77	-1.4	-0.17	0.01	-2.6	0.013	1	-3.1	0.21	Y	ZFY
0.49	0.1	0.87	-0.0077	2.5	0.014	1	-3.1	0.23	Y	SHOX
-0.38	-0.68	-0.073	0.058	-2.5	0.016	1	-3.2	0.18	Y	TTTY14
0.39	0.054	0.73	-0.018	2.3	0.024	1	-3.4	0.33	Y	TTTY8
-1	-2	-0.11	0.29	-2.2	0.029	1	-3.5	0.27	Y	PRKY
-0.48	-0.91	-0.04	0.038	-2.2	0.033	1	-3.6	0.21	Y	UTY
0.41	0.022	0.81	-0.095	2.1	0.039	1	-3.7	0.18	Y	PCDH11Y
-0.41	-0.79	-0.019	0.011	-2.1	0.04	1	-3.7	0.2	Y	RBMY1A1
0.45	0.0065	0.9	-0.068	2	0.047	1	-3.8	0.25	XY	SHOX
-0.4	-0.82	0.028	0.16	-1.9	0.066	1	-4	0.21	Y	PPP2R3B
-0.65	-1.4	0.083	0.2	-1.8	0.081	1	-4.1	0.29	Y	UTY
0.32	-0.042	0.68	-0.034	1.8	0.082	1	-4.1	0.32	Y	BPY2B
-0.48	-1	0.079	-0.066	-1.7	0.09	1	-4.2	0.15	Y	ASMTL
-0.28	-0.61	0.05	-0.085	-1.7	0.094	1	-4.2	0.21	Y	TTTY2
-0.52	-1.2	0.12	-0.19	-1.6	0.11	1	-4.3	0.19	Y	GTPBP6

Table S4: Association between cell-type composition estimated using bulk transcriptomic data (immunedecov R package) and LOY status.

	effect	inf	sup	pvalue
B cell naive	-1.57	-2.97	-0.16	0.03454
Neutrophil	-1.15	-2.23	-0.06	0.04477
NK cell	0.05	-0.01	0.10	0.08817
T cell NK	-0.85	-1.86	0.16	0.1077
Monocyte	0.97	-0.20	2.15	0.1115
Macrophage	1.05	-0.27	2.37	0.1258
T cell CD8+ naive	1.00	-0.39	2.38	0.1653
T cell regulatory (Tregs)	1.12	-0.49	2.74	0.1809
Eosinophil	-0.75	-1.89	0.40	0.207
B cell memory	-0.85	-2.25	0.55	0.2414
B cell plasma	-0.53	-1.43	0.37	0.258
T cell CD4+ (non-regulatory)	0.53	-0.38	1.45	0.2599
Macrophage M2	0.35	-0.27	0.98	0.2738
T cell CD4+ central memory	0.45	-0.39	1.30	0.3009
T cell CD4+ Th2	-0.26	-0.77	0.24	0.3128
Plasmacytoid dendritic cell	-0.73	-2.18	0.72	0.3304
T cell CD4+ effector memory	0.83	-0.85	2.51	0.3401
Myeloid dendritic cell	0.53	-0.60	1.66	0.365
T cell CD8+	-0.51	-1.79	0.76	0.4345
Mast cell	0.27	-0.41	0.95	0.4372
Common lymphoid progenitor	0.18	-0.31	0.67	0.4699
Macrophage M1	0.33	-0.72	1.38	0.5399
Cancer associated fibroblast	-0.43	-1.83	0.98	0.5559
T cell CD8+ central memory	0.35	-0.89	1.59	0.5845
T cell gamma delta	0.00	-0.00	0.00	0.6374
Class-switched memory B cell	-0.27	-1.74	1.20	0.7227
T cell CD4+ naive	0.15	-1.35	1.65	0.8456
Common myeloid progenitor	0.11	-1.18	1.39	0.8695
Hematopoietic stem cell	-0.04	-0.52	0.45	0.884
T cell CD4+ Th1	0.11	-1.59	1.81	0.9036
T cell CD8+ effector memory	-0.00	-0.00	0.00	0.9219
Granulocyte-monocyte progenitor	-2.22	-3.09	-1.35	1.085e-05
Endothelial cell	1.40	0.77	2.02	7.856e-05

Table S5: GO enrichment analysis of differentially expressed genes in individuals with LOY. GO terms significant at 5% FDR are shown.

Description	pvalue	p.adjust
blood coagulation	6.3×10^{-9}	2.6×10^{-6}
hemostasis	7.3×10^{-9}	2.6×10^{-6}
coagulation	7.6×10^{-9}	2.6×10^{-6}
platelet degranulation	1.6×10^{-7}	4.1×10^{-5}
regulation of body fluid levels	4.1×10^{-7}	8.3×10^{-5}
leukocyte migration	1.9×10^{-6}	0.00032
platelet activation	8.6×10^{-6}	0.0013
oxygen transport	1.4×10^{-5}	0.0017
gas transport	2.9×10^{-5}	0.0033
platelet aggregation	3.4×10^{-5}	0.0035
blood coagulation, fibrin clot formation	8.5×10^{-5}	0.0079
homotypic cell-cell adhesion	0.0001	0.009
cellular oxidant detoxification	0.00028	0.022
cellular detoxification	0.00037	0.027
detoxification	0.00046	0.031
cell-substrate adhesion	0.00054	0.035
cell-matrix adhesion	0.00059	0.035

Table S6: KEGG enrichment analysis of differentially expressed genes in individuals with LOY. KEGG terms significant at 5% FDR are shown

Description	pvalue	p.adjust
ECM-receptor interaction	3.5×10^{-5}	0.0024
Platelet activation	0.00018	0.0063
Hematopoietic cell lineage	0.00085	0.015
Viral protein interaction with cytokine and cytokine receptor	0.00088	0.015
Malaria	0.0013	0.016
Cytokine-cytokine receptor interaction	0.0015	0.016
Focal adhesion	0.0016	0.016

Table S7: Top GO enrichment of differentially expressed genes in the two cell lines infected with SAS-CoV-2 (NHBE and A549).

Description	pvalue	p.adjust
response to virus	1.7×10^{-21}	5.9×10^{-18}
defense response to other organism	1.5×10^{-19}	2.6×10^{-16}
type I interferon signaling pathway	8.8×10^{-17}	7.5×10^{-14}
cellular response to type I interferon	8.8×10^{-17}	7.5×10^{-14}
response to type I interferon	2×10^{-16}	1.4×10^{-13}
defense response to virus	2.6×10^{-16}	1.5×10^{-13}
response to molecule of bacterial origin	3.9×10^{-16}	1.9×10^{-13}
response to lipopolysaccharide	1.1×10^{-15}	4.6×10^{-13}
response to interferon-gamma	8×10^{-14}	3×10^{-11}
regulation of inflammatory response	6.3×10^{-13}	2.2×10^{-10}
positive regulation of response to external stimulus	2.5×10^{-12}	7.8×10^{-10}
negative regulation of viral genome replication	9.4×10^{-12}	2.7×10^{-9}
acute-phase response	1.4×10^{-11}	3.6×10^{-9}
acute inflammatory response	6.2×10^{-11}	1.5×10^{-8}
regulation of innate immune response	8.8×10^{-11}	2×10^{-8}
cellular response to lipopolysaccharide	1.1×10^{-10}	2.4×10^{-8}
positive regulation of inflammatory response	1.2×10^{-10}	2.5×10^{-8}
cellular response to molecule of bacterial origin	2.1×10^{-10}	4×10^{-8}
regulation of viral genome replication	2.4×10^{-10}	4.3×10^{-8}
neutrophil mediated immunity	2.7×10^{-10}	4.6×10^{-8}
negative regulation of viral process	4.1×10^{-10}	6.6×10^{-8}
negative regulation of viral life cycle	8.3×10^{-10}	1.3×10^{-7}
cellular response to biotic stimulus	1.3×10^{-9}	1.8×10^{-7}
neutrophil activation	1.3×10^{-9}	1.8×10^{-7}
positive regulation of MAPK cascade	1.3×10^{-9}	1.8×10^{-7}
regulation of response to cytokine stimulus	1.8×10^{-9}	2.3×10^{-7}
positive regulation of peptidyl-tyrosine phosphorylation	1.9×10^{-9}	2.4×10^{-7}
cornification	1.9×10^{-9}	2.4×10^{-7}
positive regulation of innate immune response	2.8×10^{-9}	3.2×10^{-7}
regulation of multi-organism process	2.8×10^{-9}	3.2×10^{-7}
humoral immune response	2.9×10^{-9}	3.2×10^{-7}
regulation of lipid storage	3×10^{-9}	3.2×10^{-7}
positive regulation of cytokine production	3.2×10^{-9}	3.3×10^{-7}
neutrophil degranulation	3.5×10^{-9}	3.5×10^{-7}
neutrophil activation involved in immune response	4×10^{-9}	3.9×10^{-7}
viral genome replication	4.5×10^{-9}	4.2×10^{-7}
cell chemotaxis	4.5×10^{-9}	4.2×10^{-7}
positive regulation of DNA-binding transcription factor activity	5.8×10^{-9}	5.3×10^{-7}
positive regulation of smooth muscle cell proliferation	6.3×10^{-9}	5.5×10^{-7}
leukocyte migration	7.8×10^{-9}	6.7×10^{-7}

Table S8: Intersection between significant genes in the blood transcriptomic comparison of LOY versus no LOY individuals and in the two cell lines infected with SAS-CoV-2 (NHBE and A549)

Gene	Chr	logFC (LOY)	P.value	logFC (NHBE)	P.value	logFC (A549)	P.value
CXCL5	4	1.7	0.014	3.5	7.1×10^{-31}	0.77	1.7×10^{-17}
IFI44L	1	-1.6	0.021	2.3	0.00028	5.7	2.5×10^{-5}
IFI6	1	-1	0.049	2.3	3.3×10^{-5}	4.3	2.3×10^{-261}
IFIT1	10	-1.2	0.032	0.82	0.00025	4.3	3×10^{-141}
IFIT3	10	-1.4	0.017	0.73	5.1×10^{-5}	2	1×10^{-32}
ITGB3	17	1.7	0.013	1.2	9.1×10^{-7}	-0.24	0.42
KRT23	17	-1.6	8.2×10^{-5}	0.59	4.5×10^{-7}	1.6	0.37
KYNU	2	1.2	0.0058	0.84	7.8×10^{-10}	0.48	1.2×10^{-5}
PROS1	3	1.5	0.0022	-0.66	4.2×10^{-6}	0.3	0.027
S100P	4	2.4	0.033	0.69	3.2×10^{-10}	-0.11	0.51
SLPI	20	-1.2	0.037	0.58	7.3×10^{-9}	0.42	0.018
TSC22D3	X	1	0.024	-0.72	6.9×10^{-10}	-0.17	0.2
VNN1	6	1.6	0.00099	1.9	3.4×10^{-8}	0.46	0.41

Supplementary Figures

Figure S1: Plots of the whole-genome molecular karyotype obtained by SNParray of blood DNA from all 133 individuals of SCOURGE with detectable mCAs

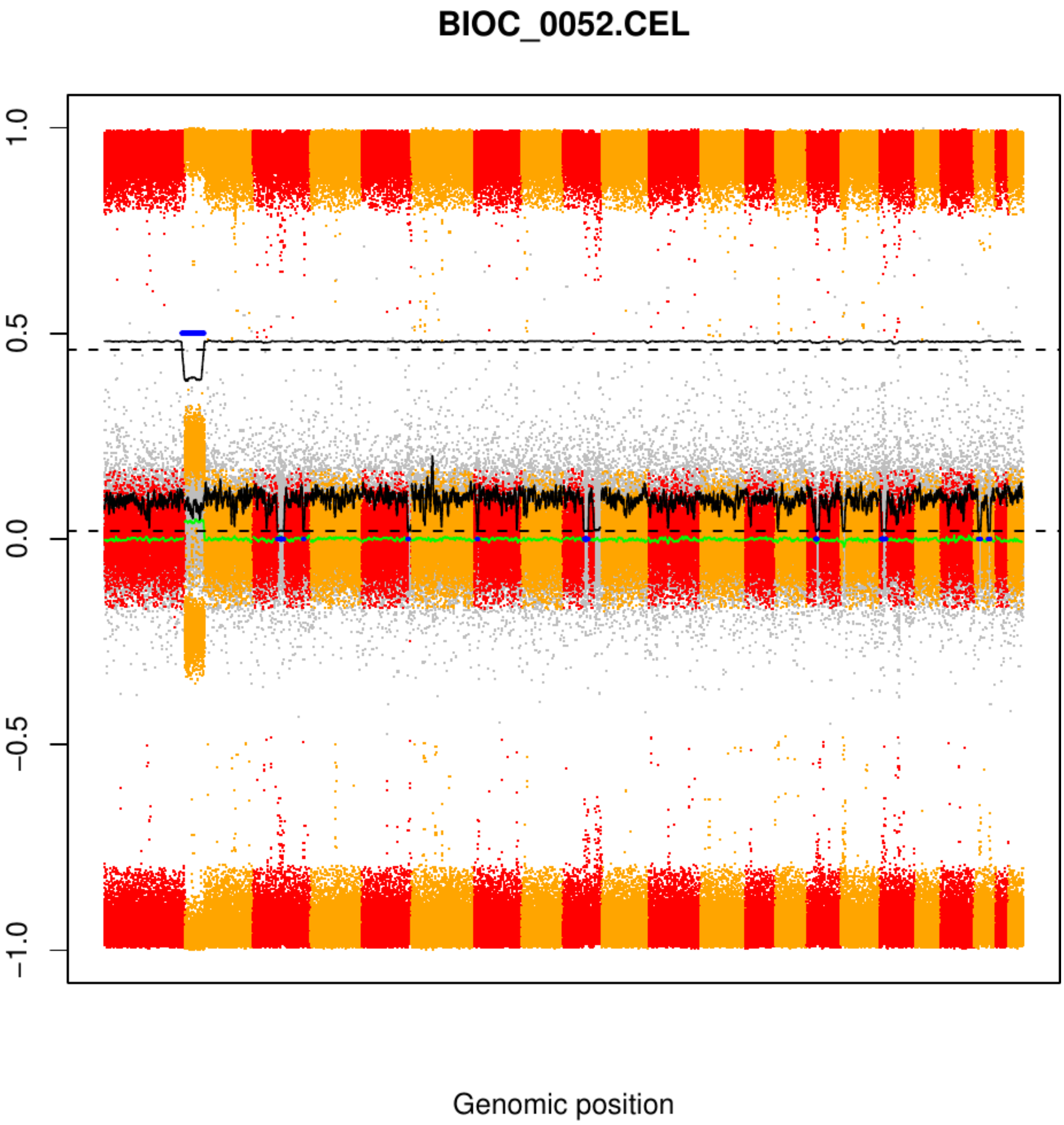

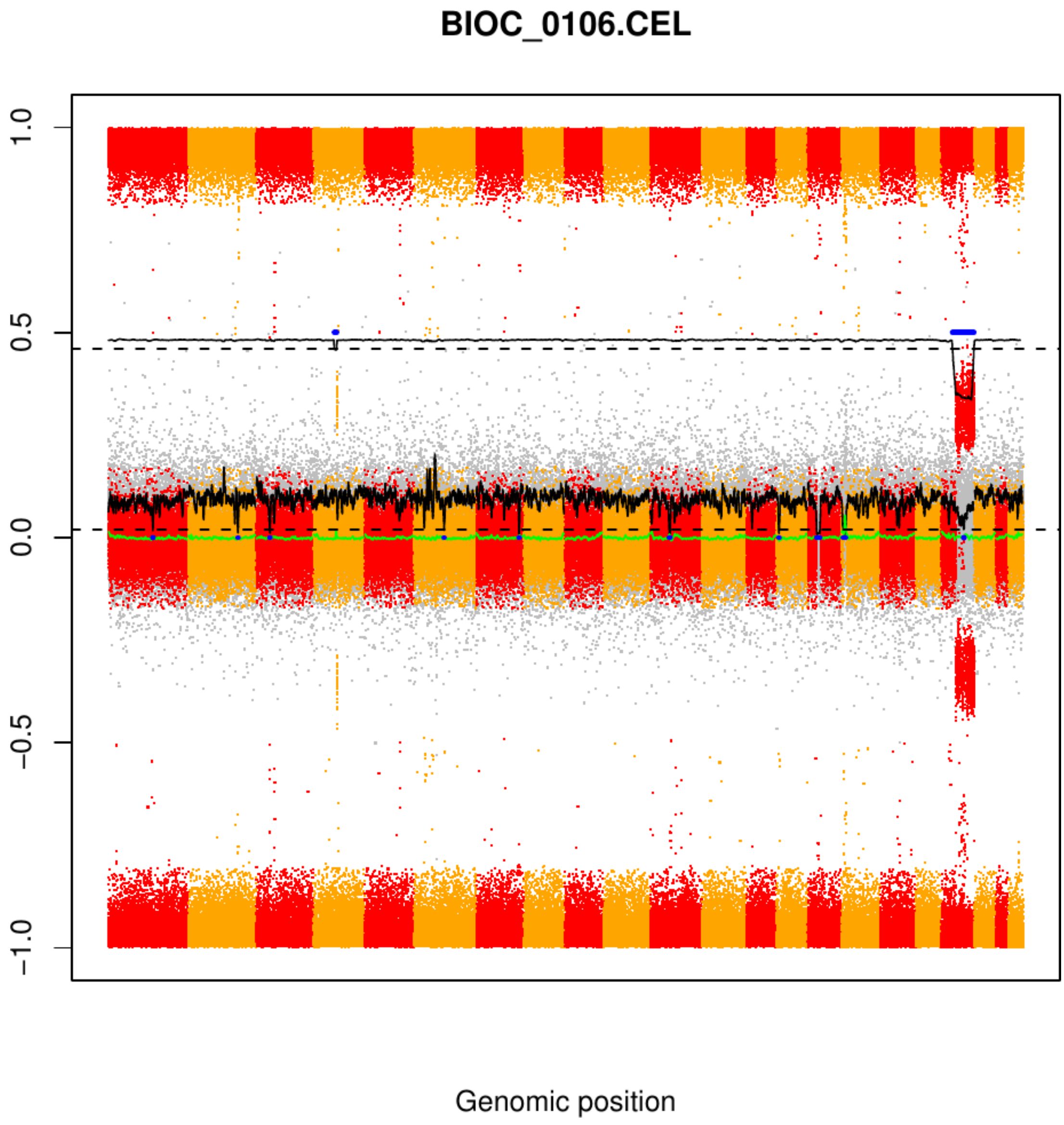
BIOC_0106.CEL
1.0
0.5
0.0
−0.5
−1.0
Genomic position

CAUL_0105.CEL

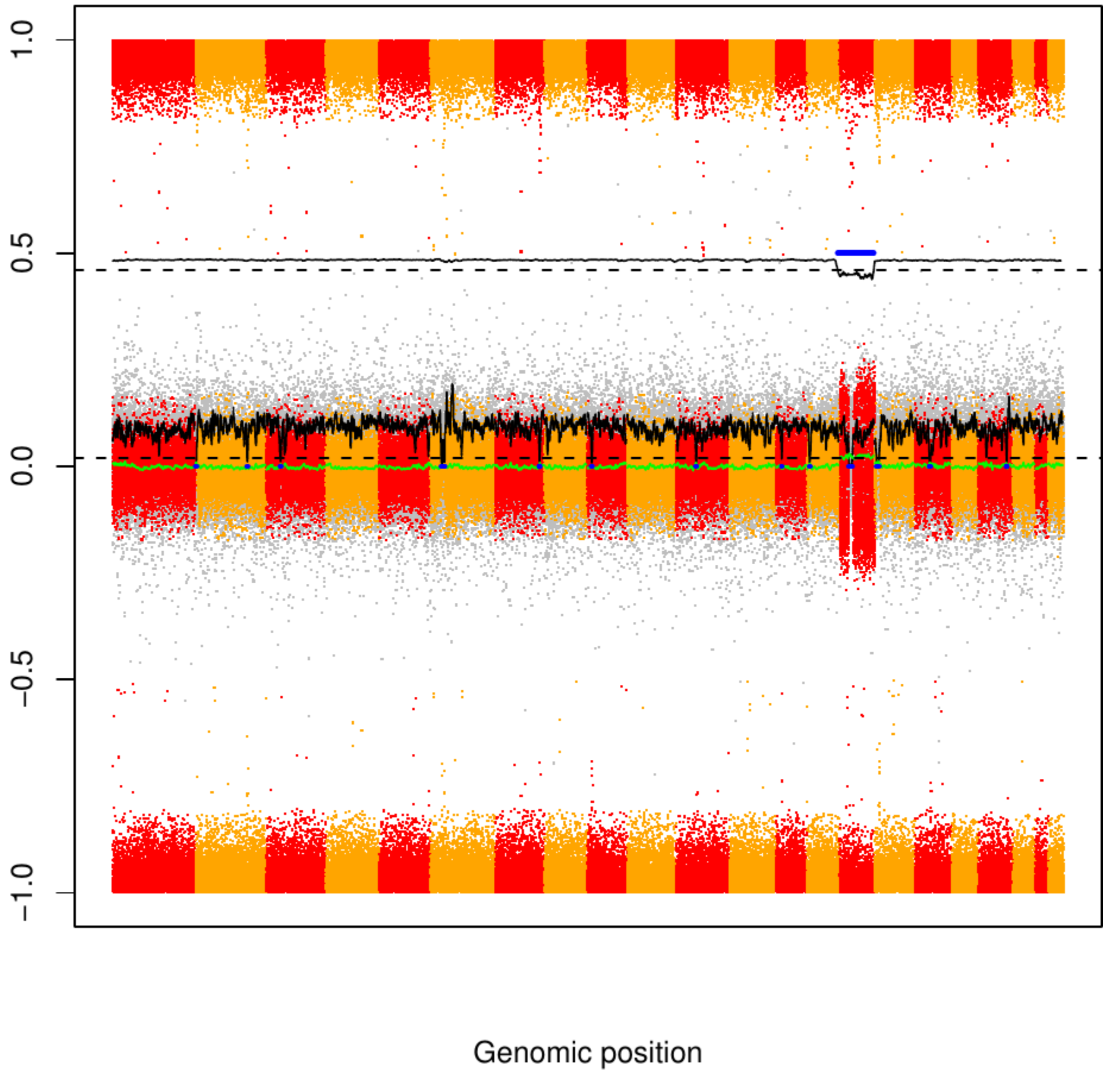

CAUL_0260.CEL

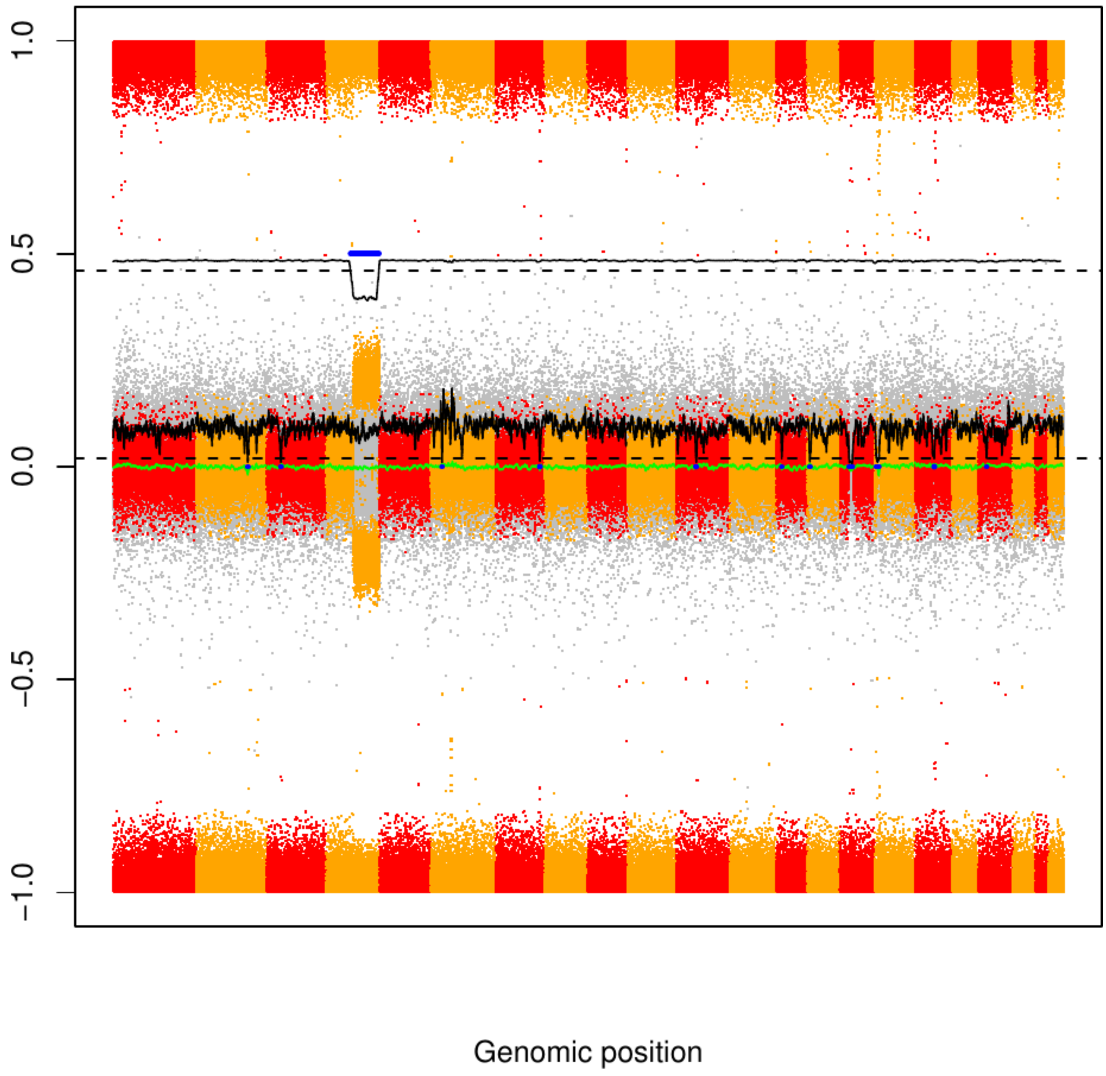

CAUL_0282.CEL

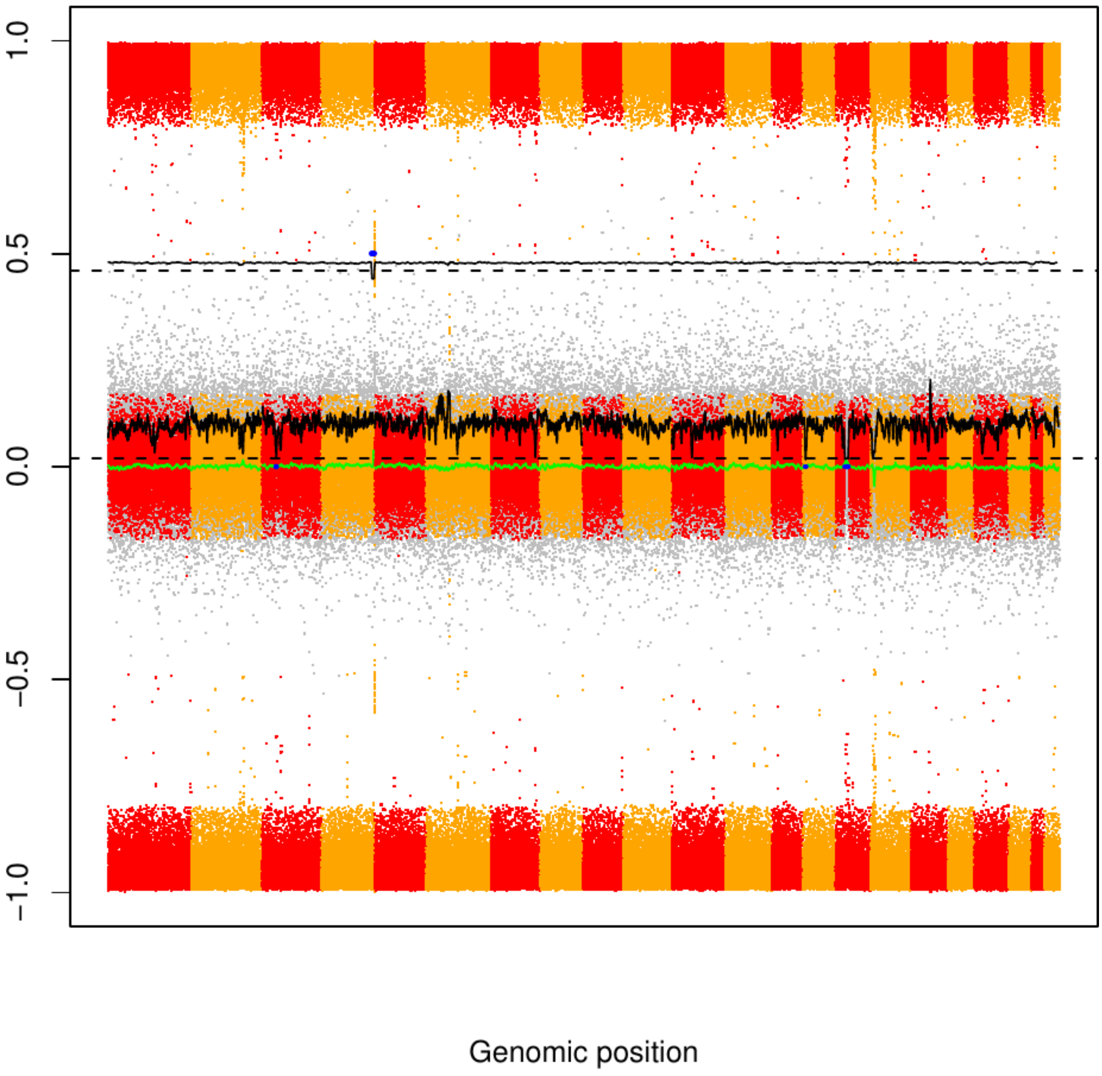

CAUL_0430.CEL

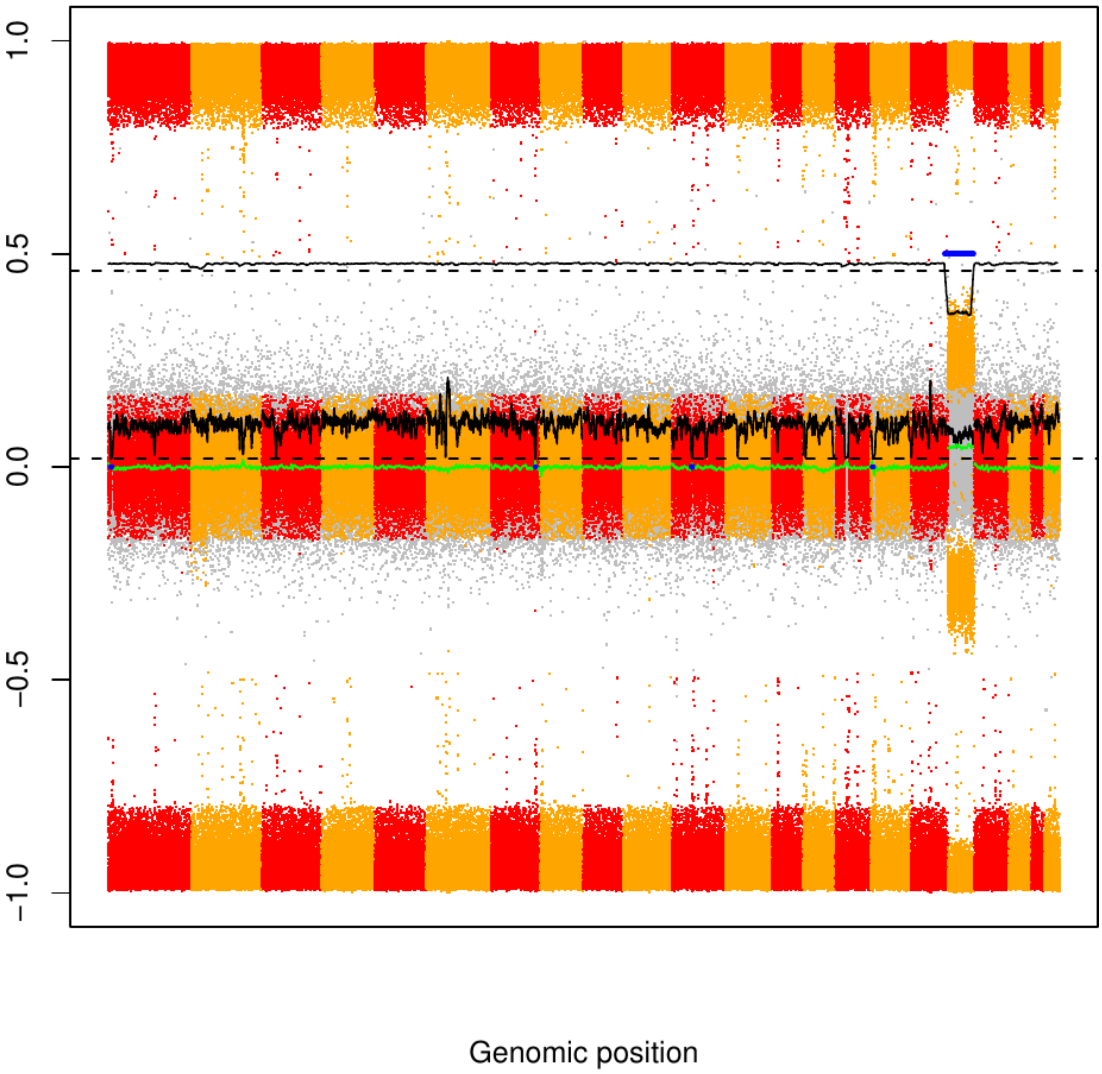

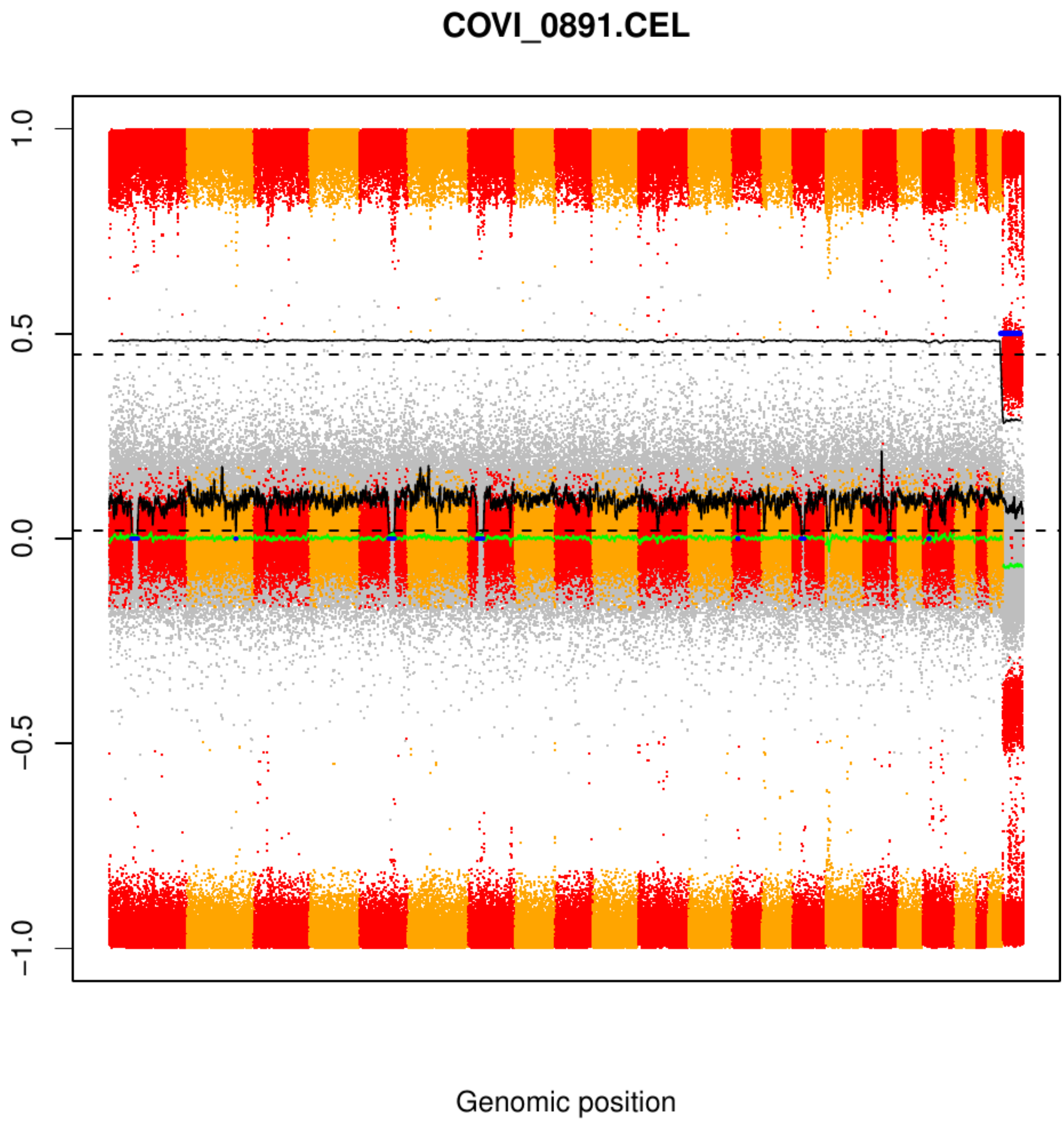
COVI_0891.CEL
1.0
0.5
0.0
−0.5
−1.0
Genomic position

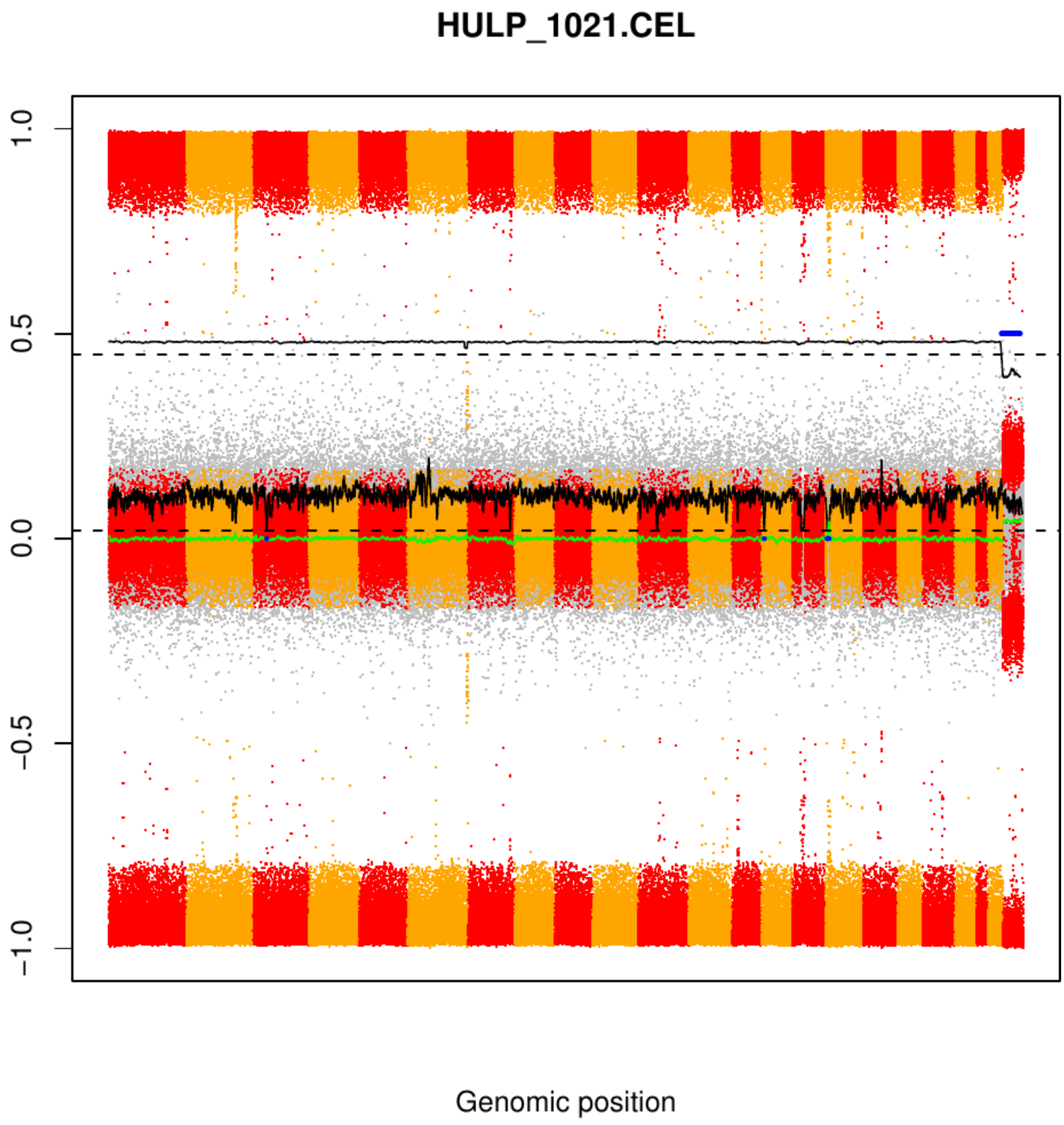
HULP_1021.CEL
1.0
0.5
0.0
−0.5
−1.0
Genomic position

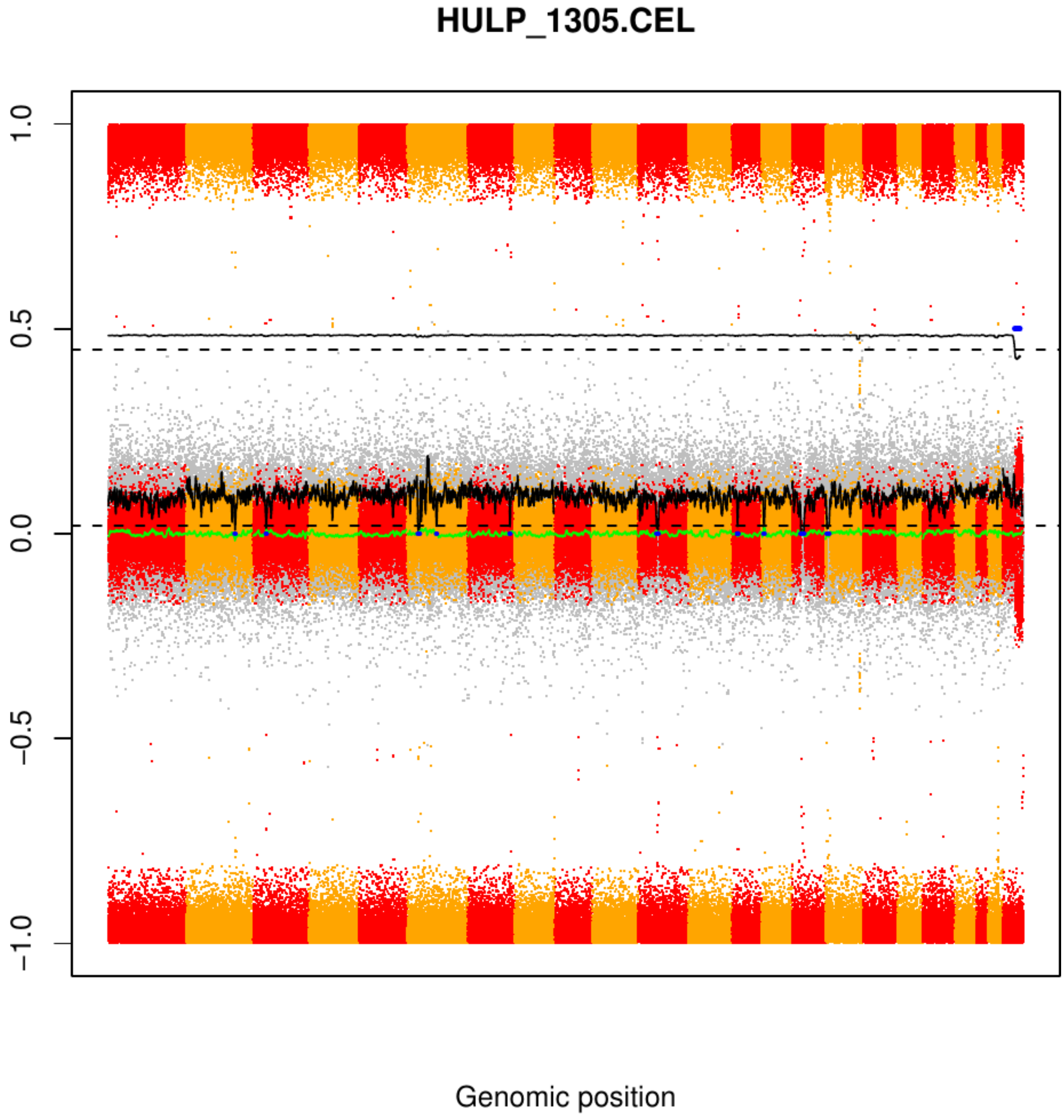
HULP_1305.CEL
1.0
0.5
0.0
−0.5
−1.0
Genomic position

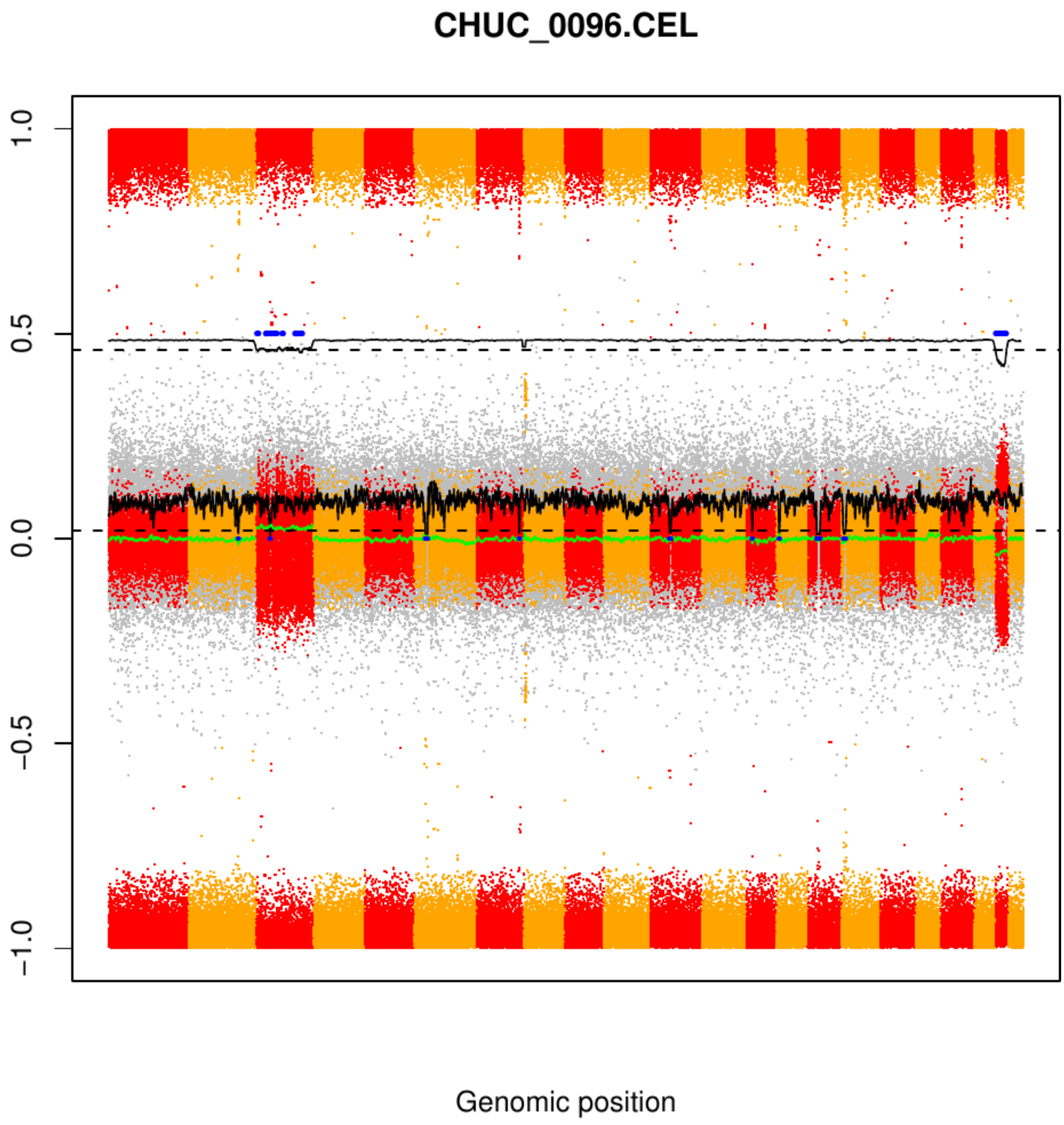
CHUC_0096.CEL
1.0
0.5
0.0
−0.5
−1.0
Genomic position

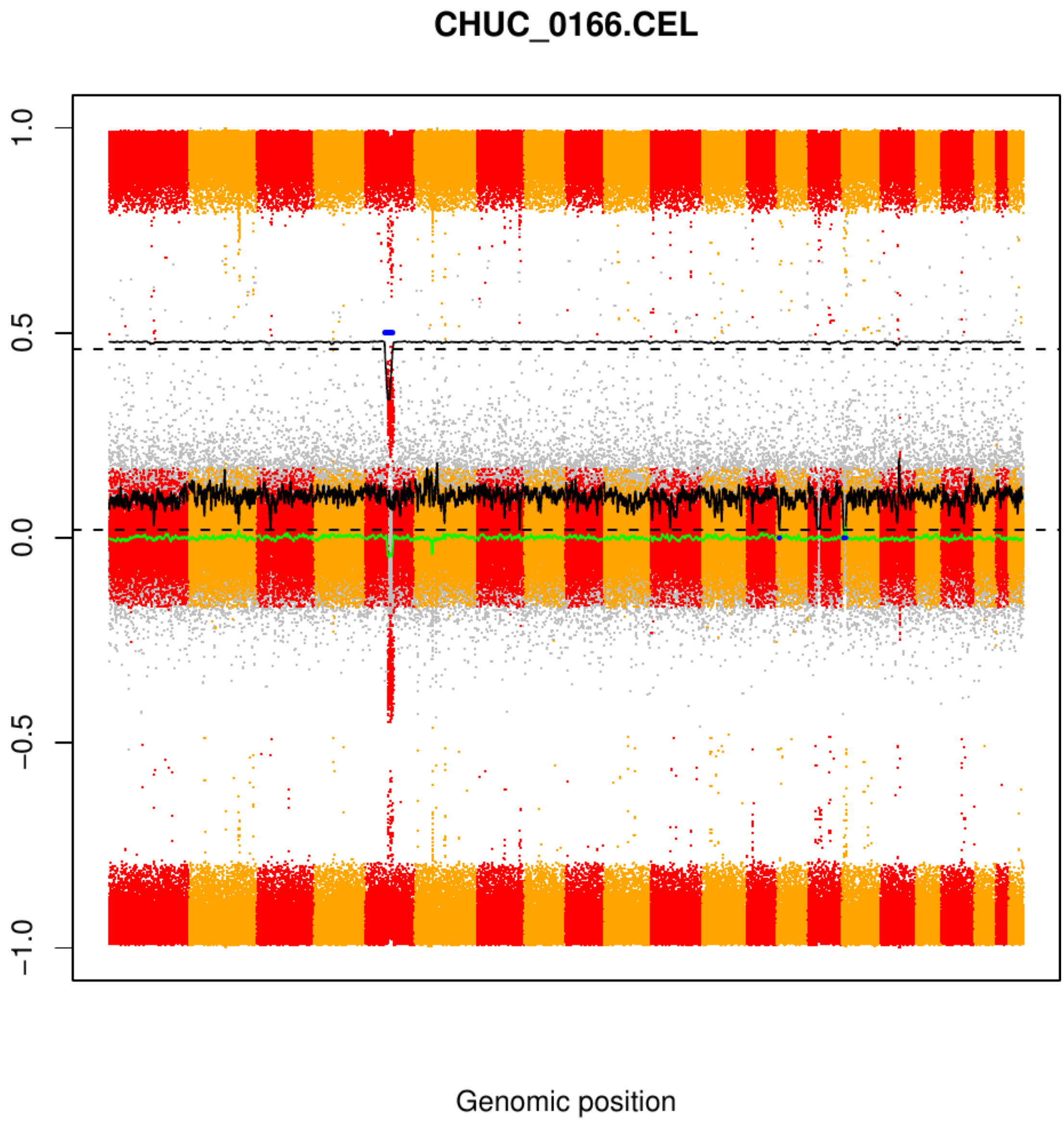

CHUC_0166.CEL
1.0
0.5
0.0
−0.5
−1.0
Genomic position

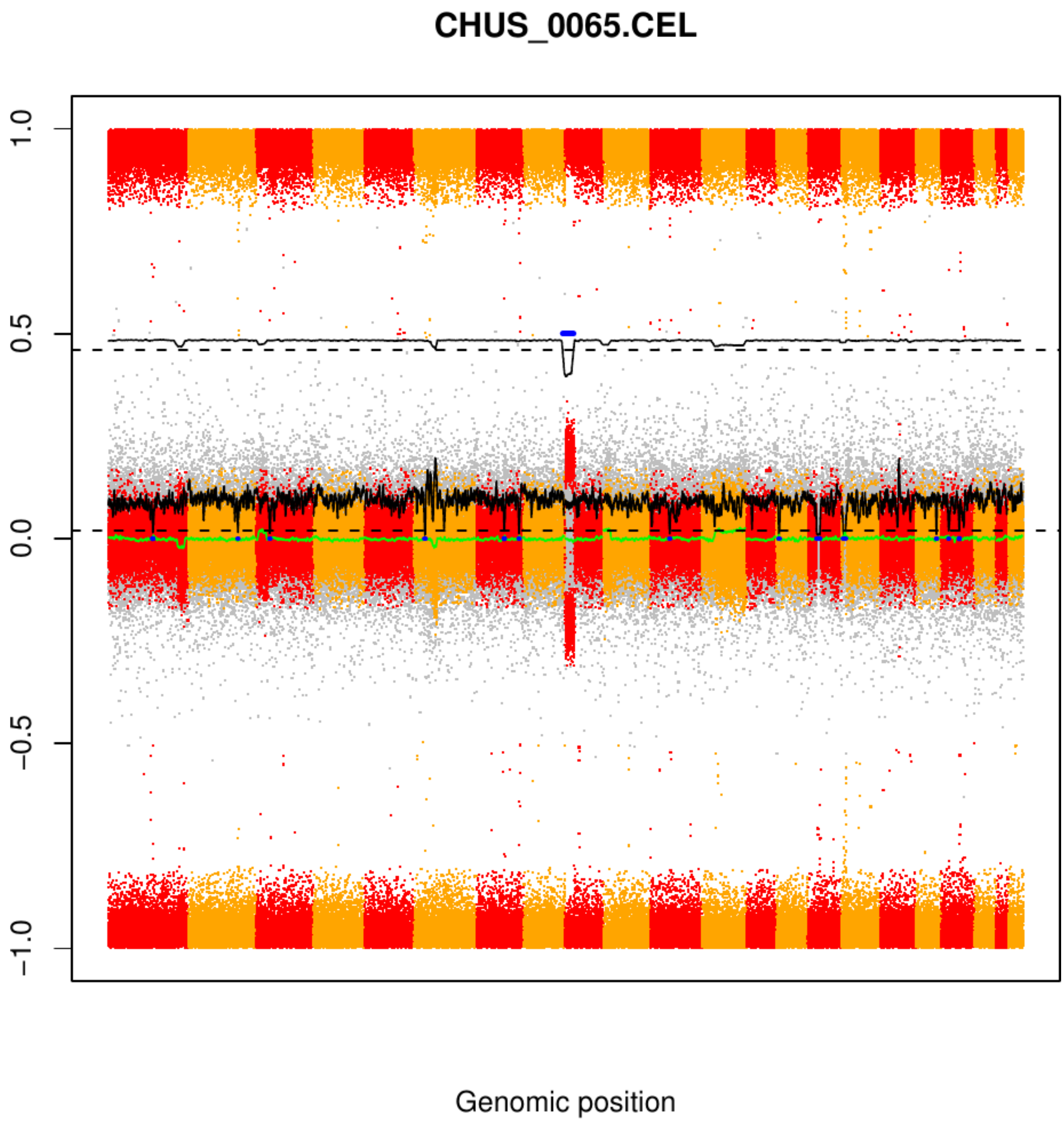
CHUS_0065.CEL
1.0
0.5
0.0
−0.5
−1.0
Genomic position

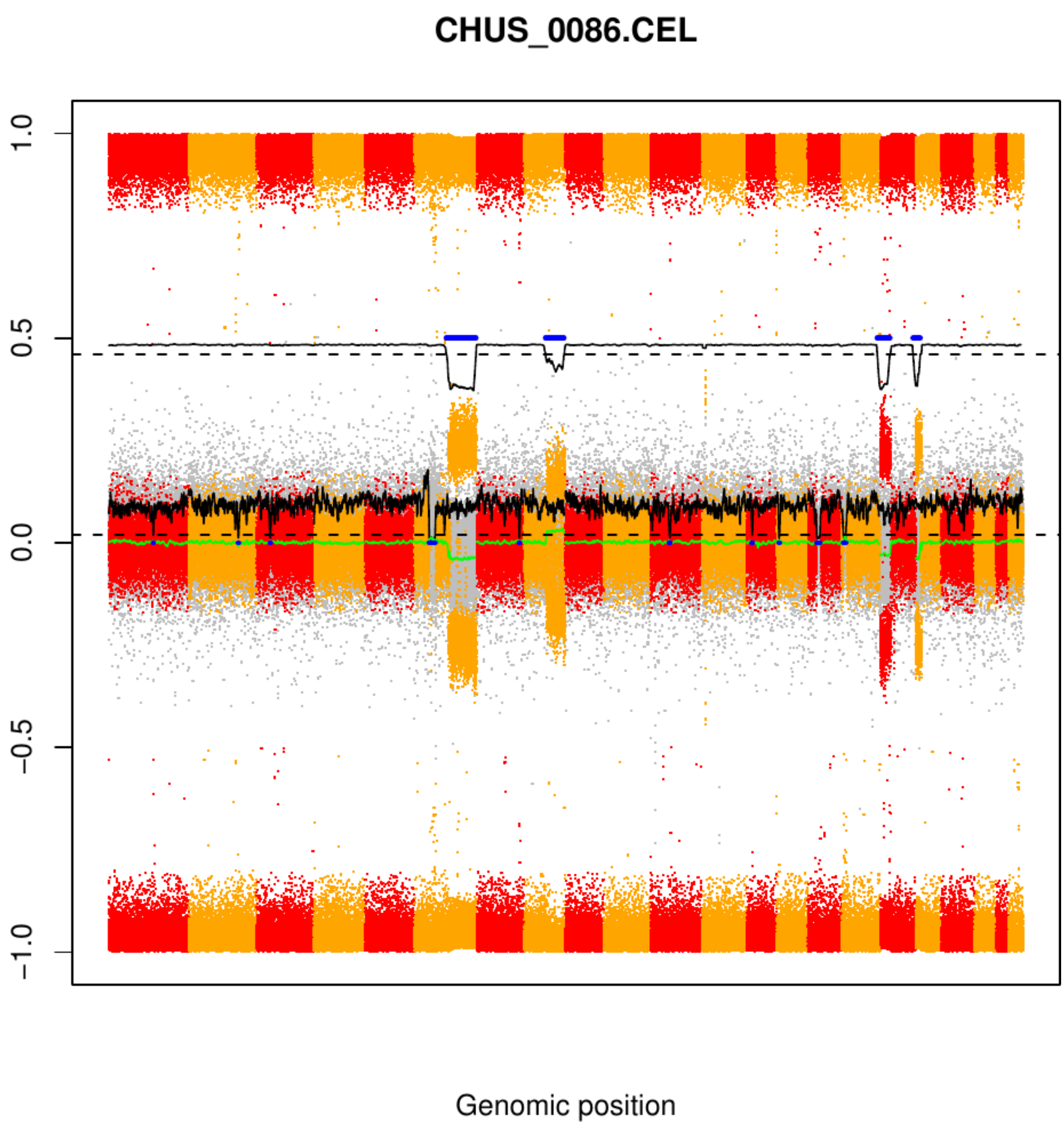
CHUS_0086.CEL
1.0
0.5
0.0
−0.5
−1.0
Genomic position

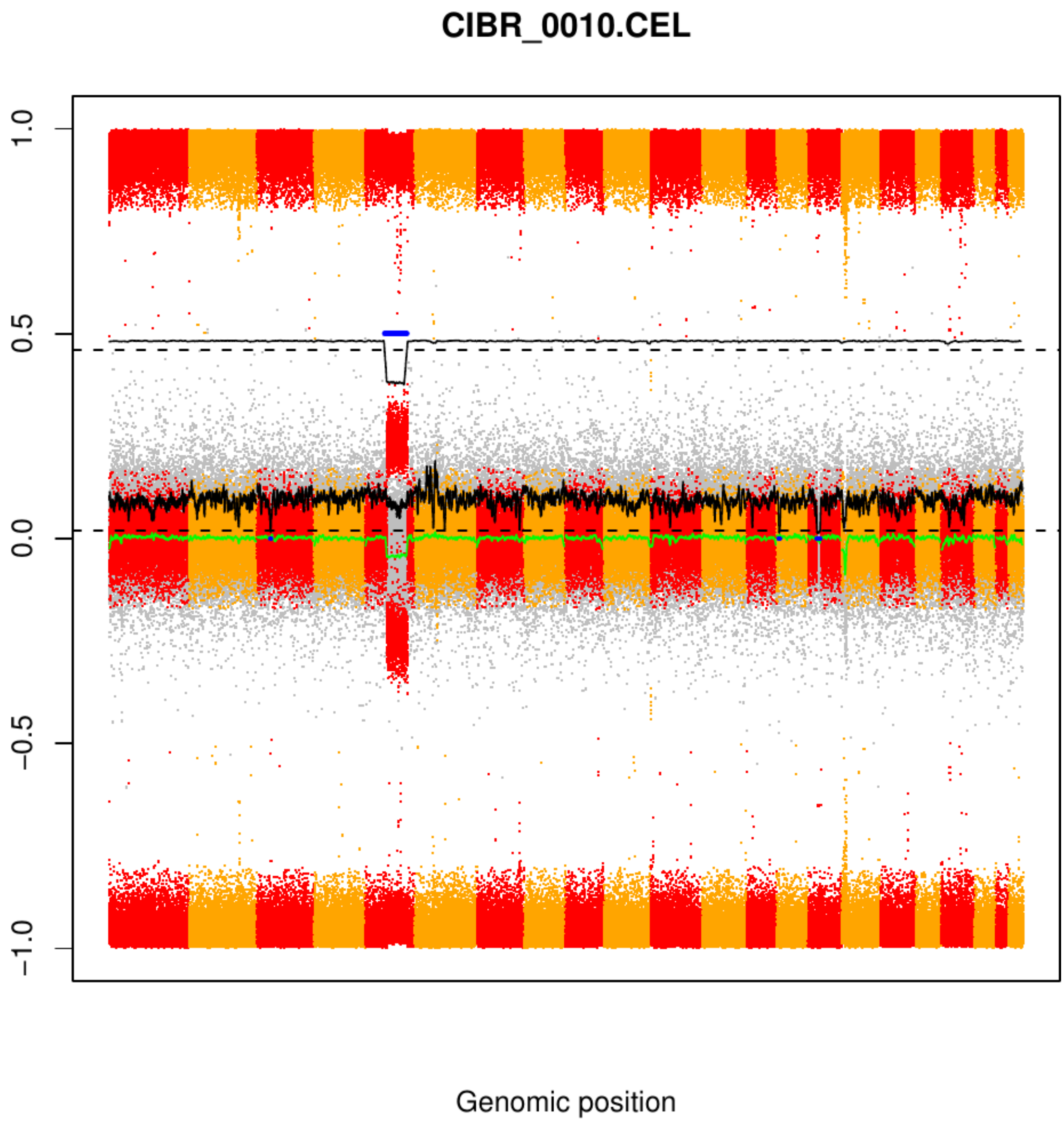
CIBR_0010.CEL
1.0
0.5
0.0
−0.5
−1.0
Genomic position

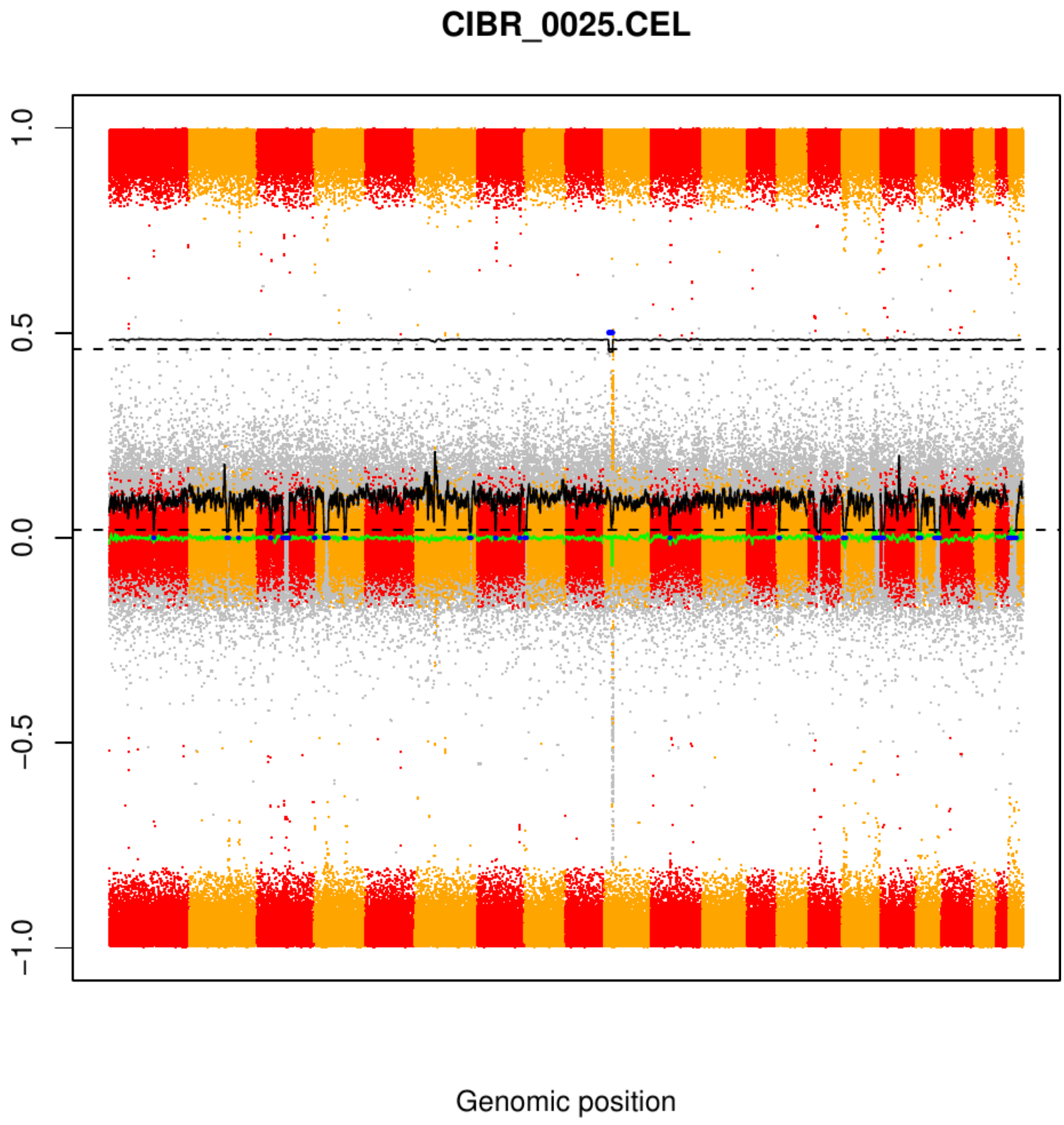
CIBR_0025.CEL
1.0
0.5
0.0
−0.5
−1.0
Genomic position

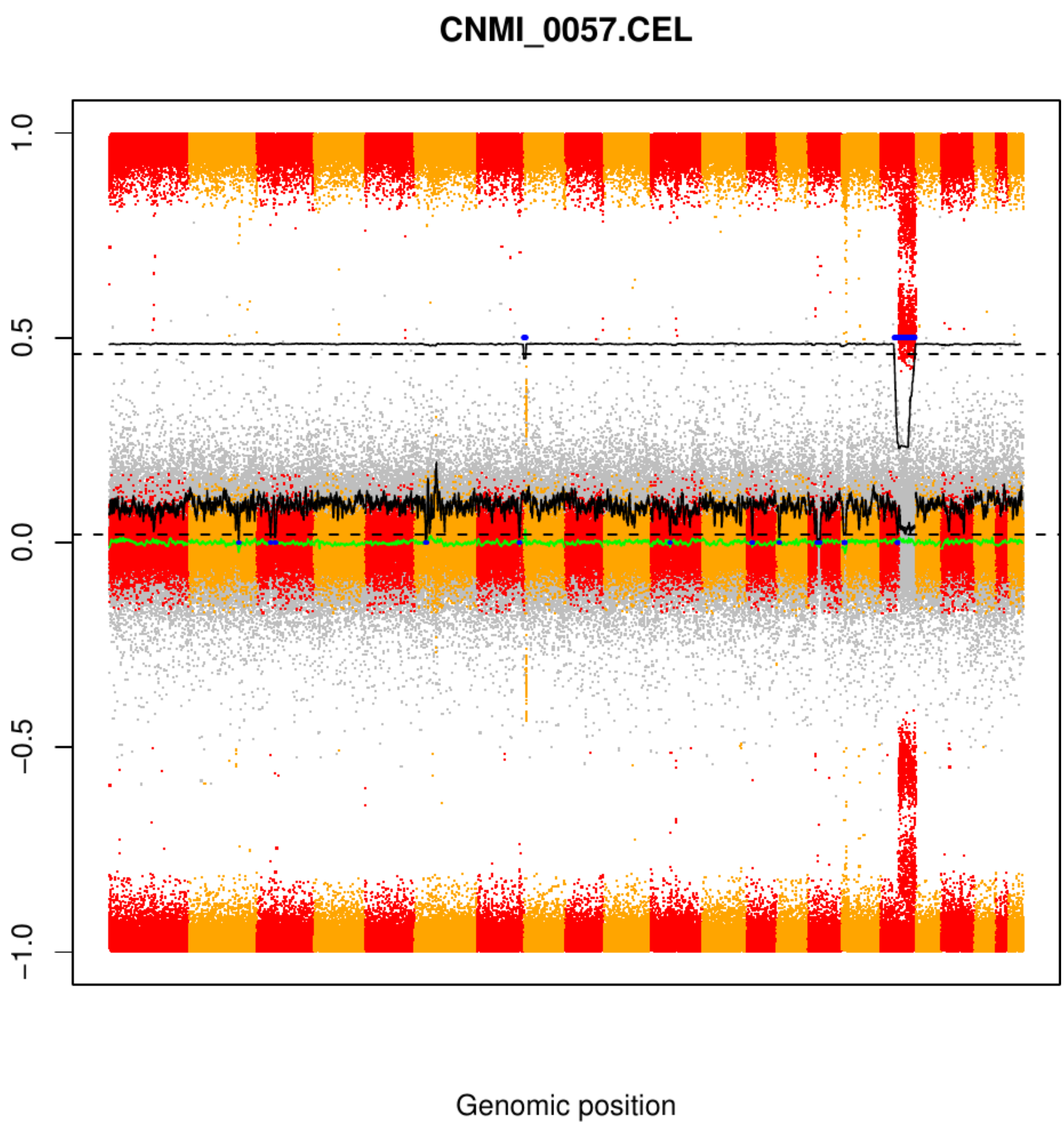
CNMI_0057.CEL
1.0
0.5
0.0
−0.5
−1.0
Genomic position

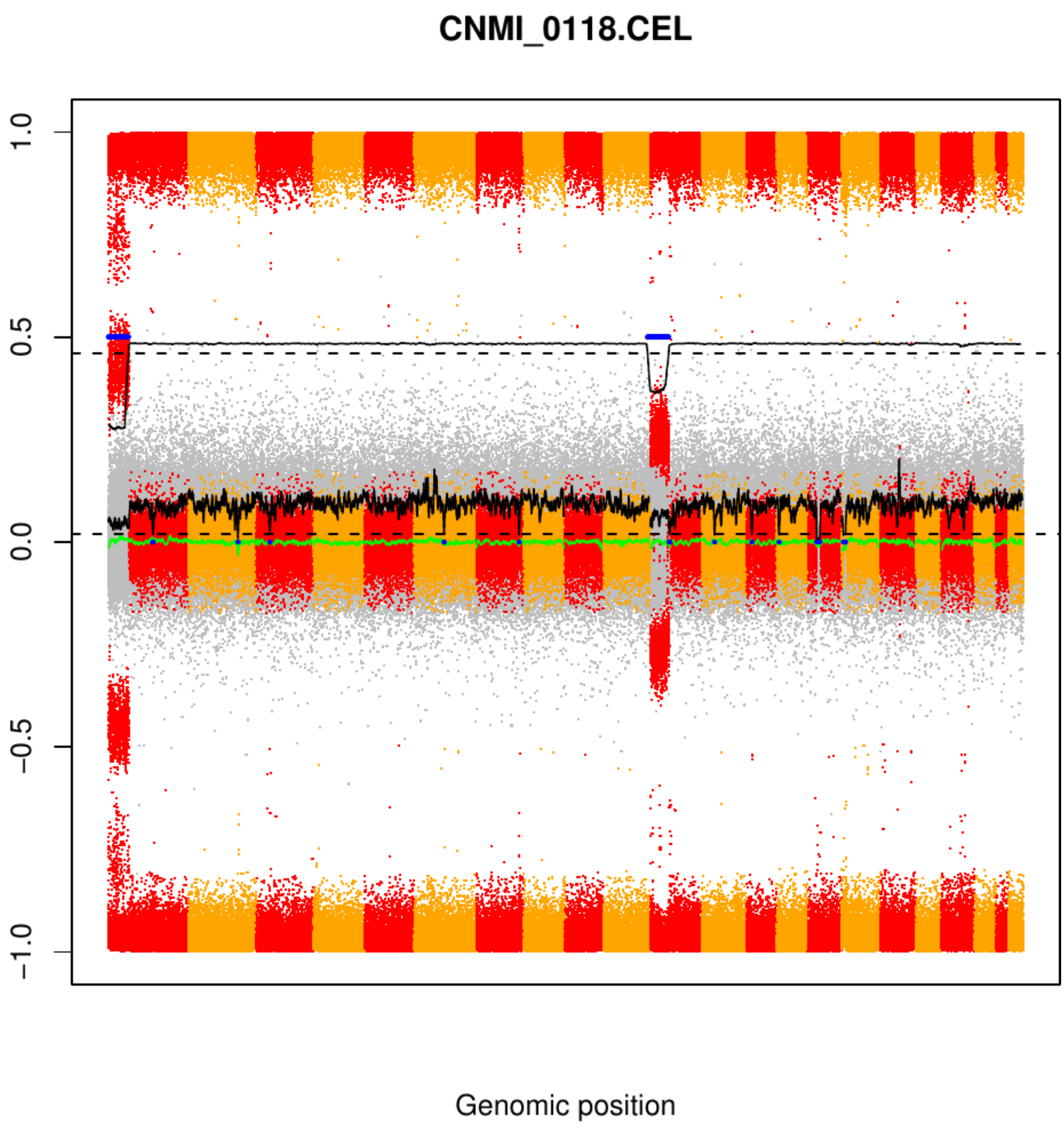
CNMI_0118.CEL
1.0
0.5
0.0
−0.5
−1.0
Genomic position

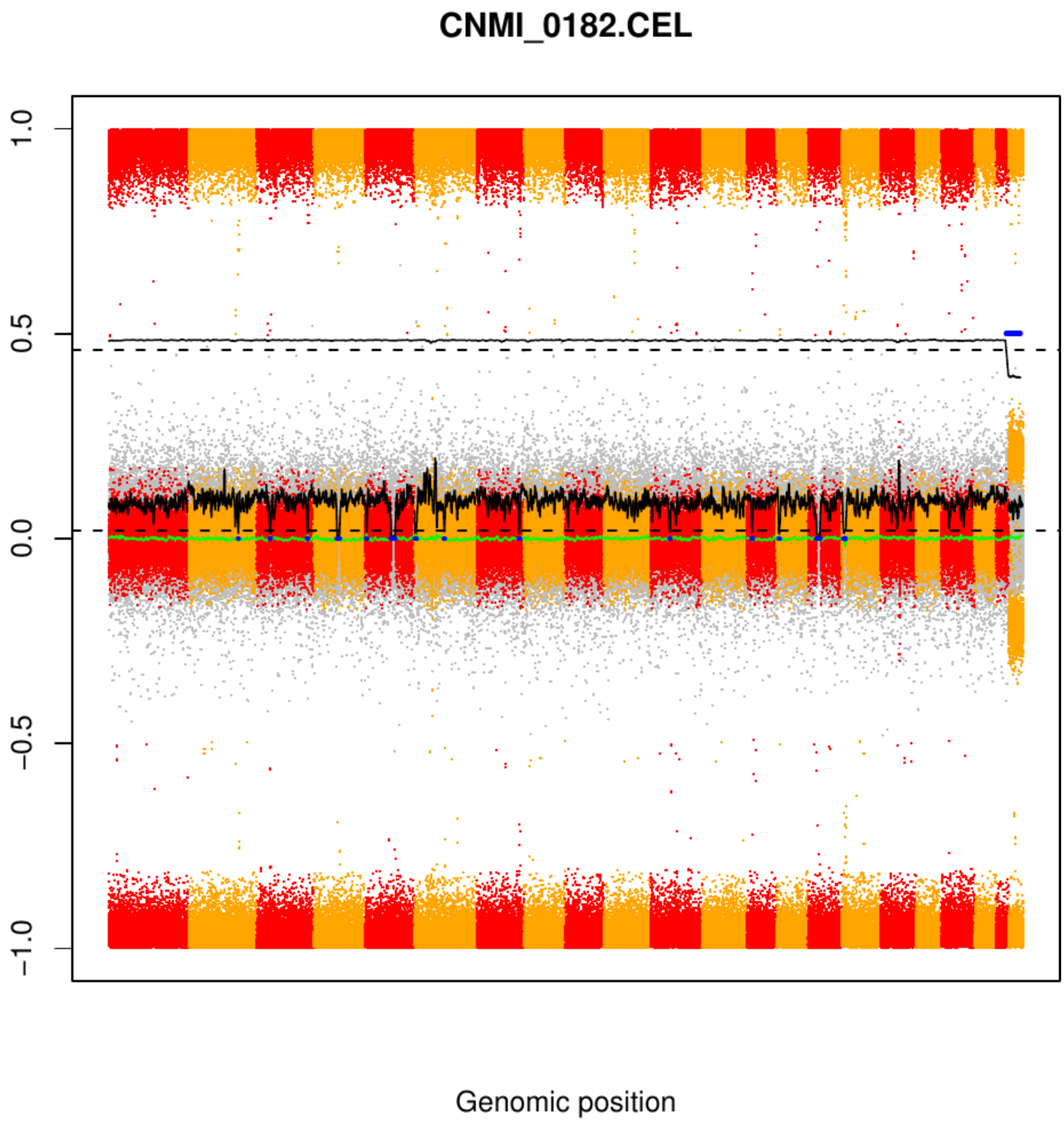
CNMI_0182.CEL
1.0
0.5
0.0
−0.5
−1.0
Genomic position

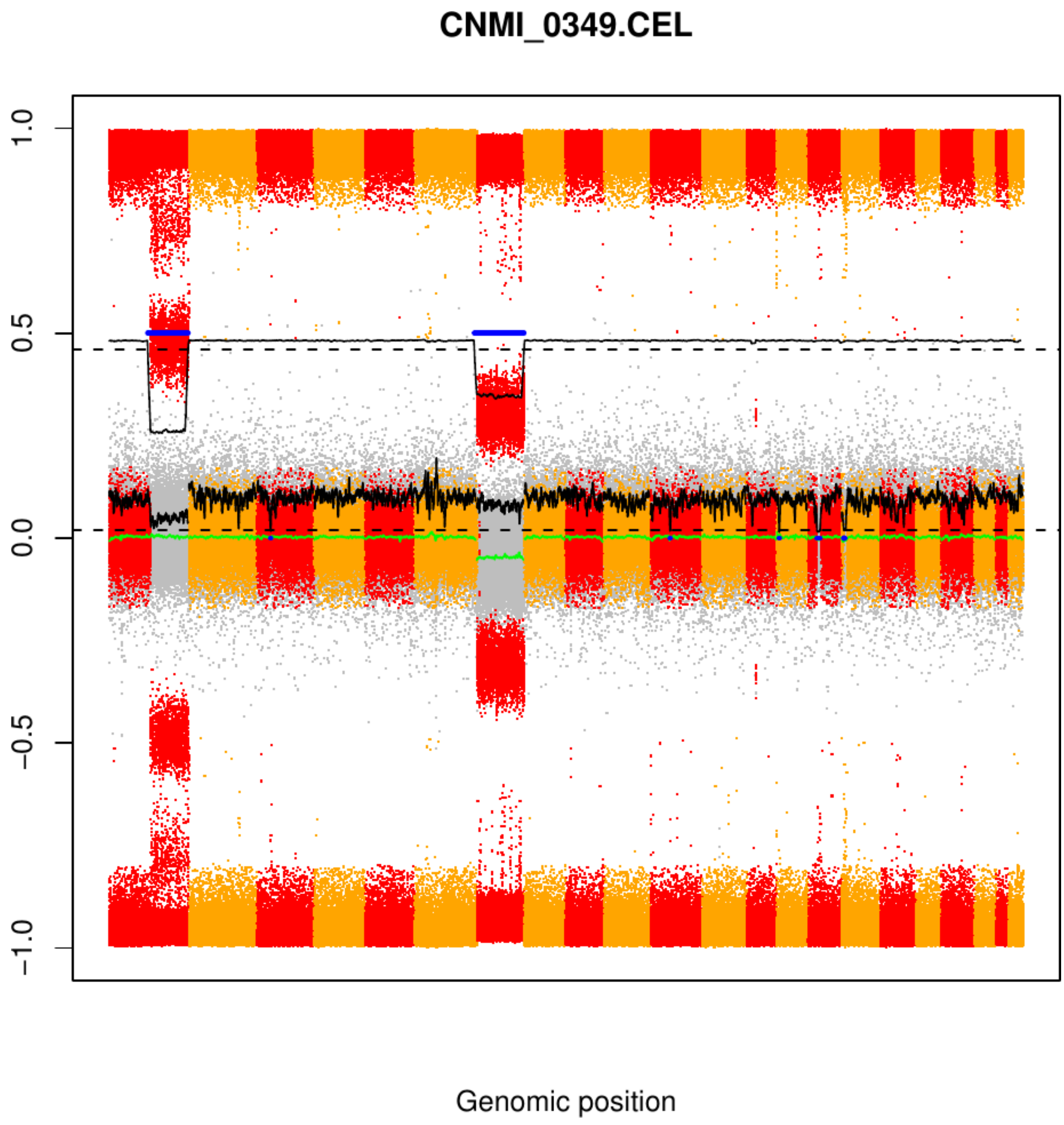
CNMI_0349.CEL
1.0
0.5
0.0
−0.5
−1.0
Genomic position

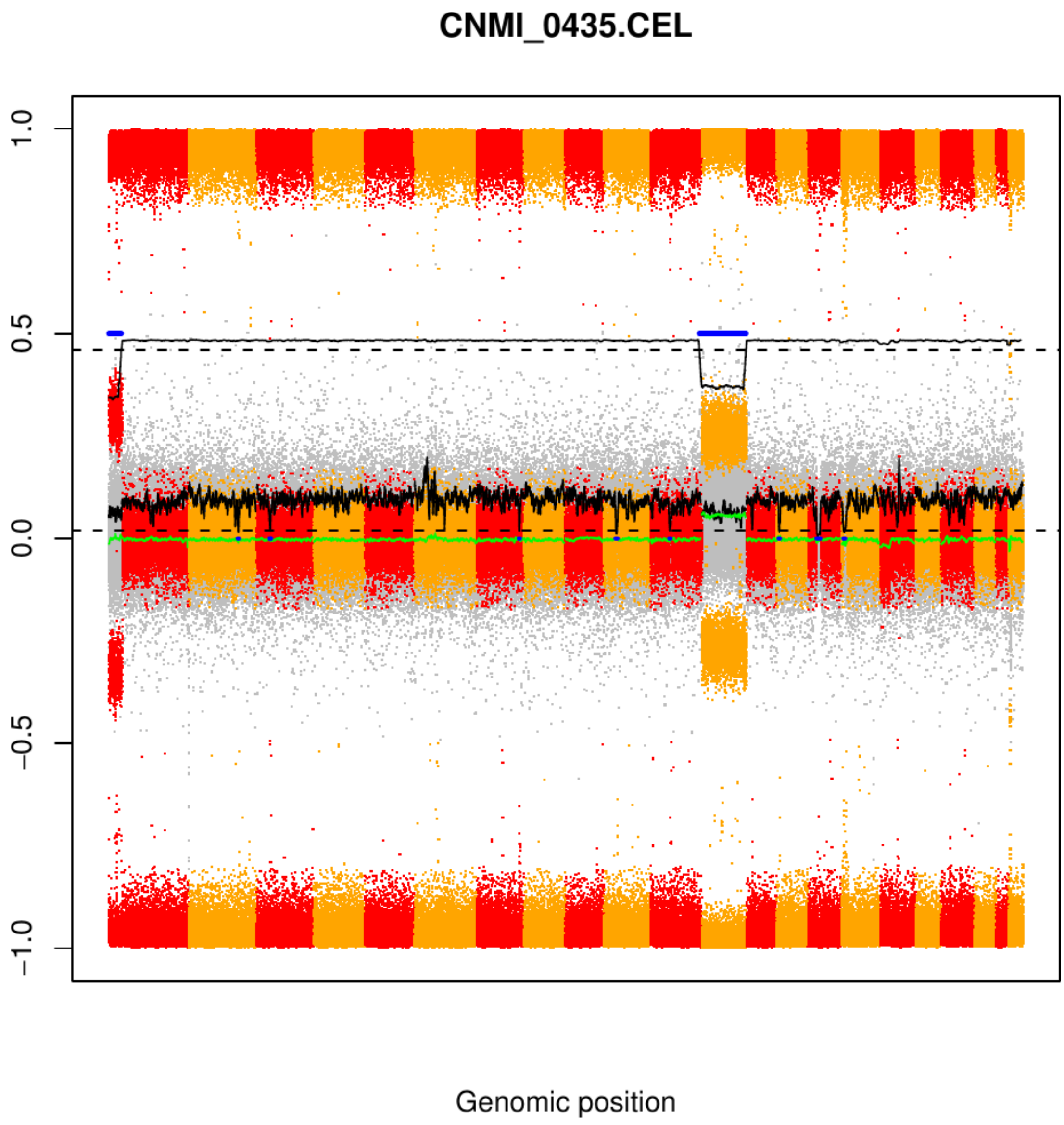

CNMI_0435.CEL
1.0
0.5
0.0
−0.5
−1.0
Genomic position

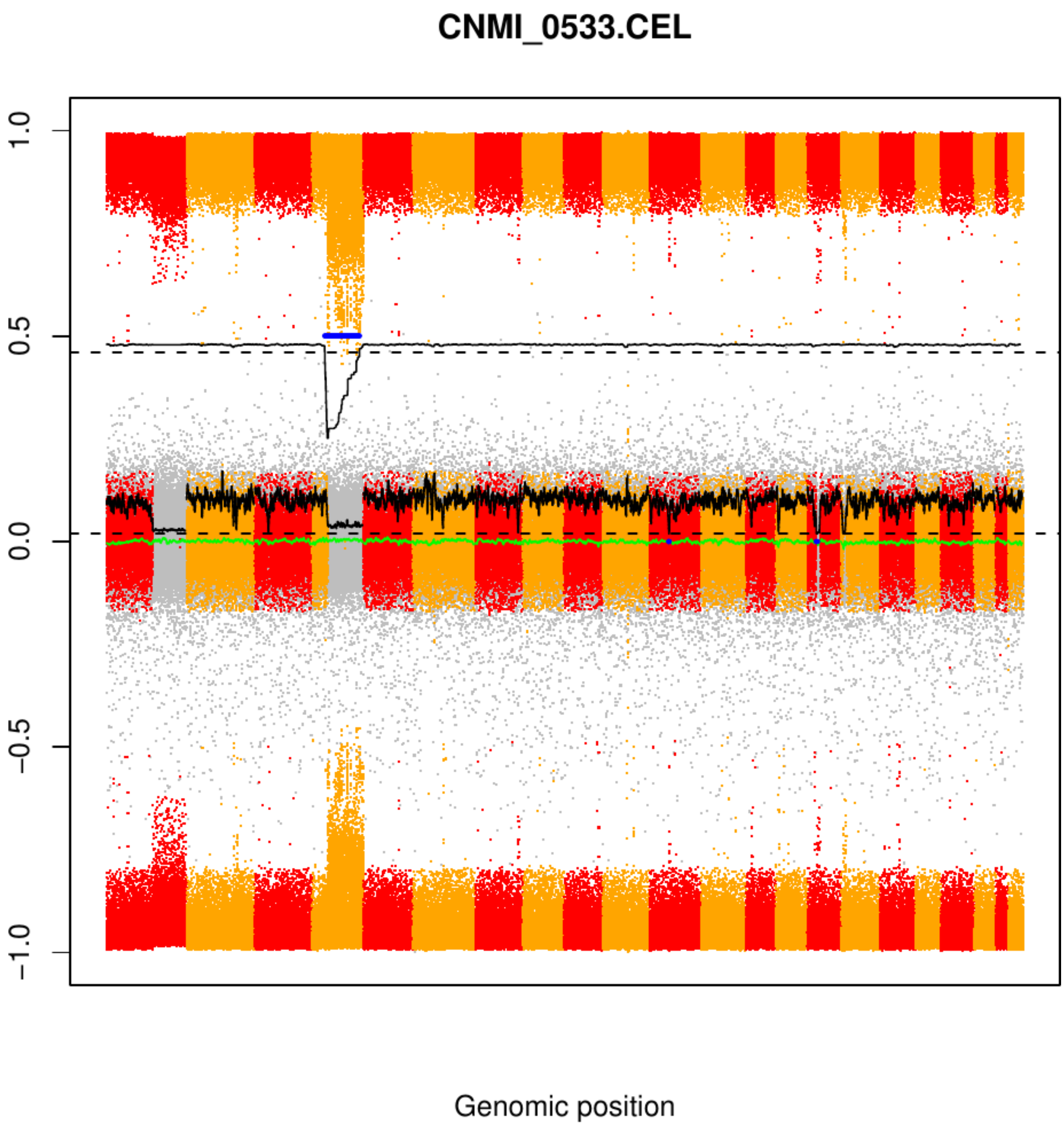
CNMI_0533.CEL
1.0
0.5
0.0
−0.5
−1.0
Genomic position

CNMI_0621.CEL

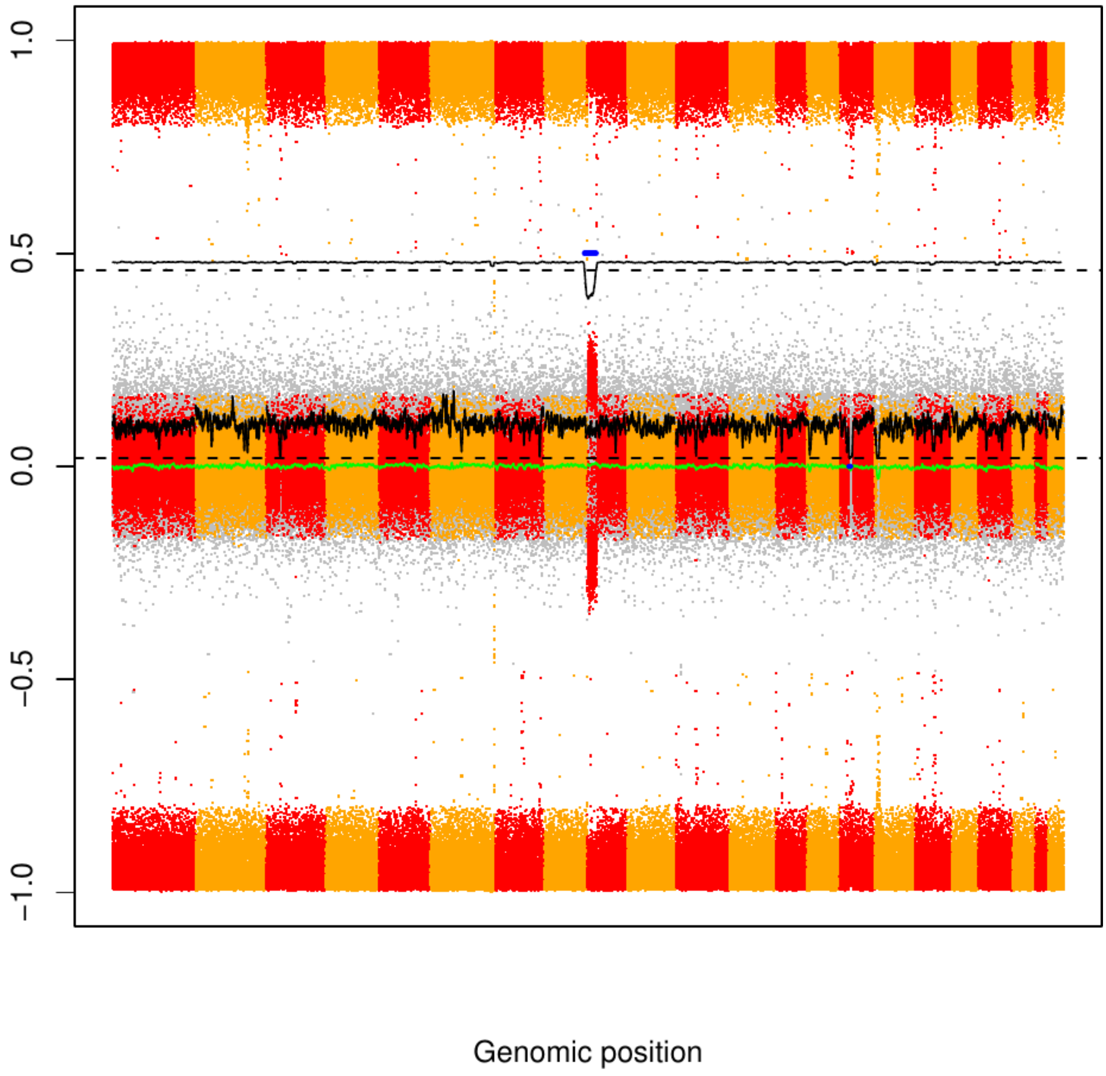

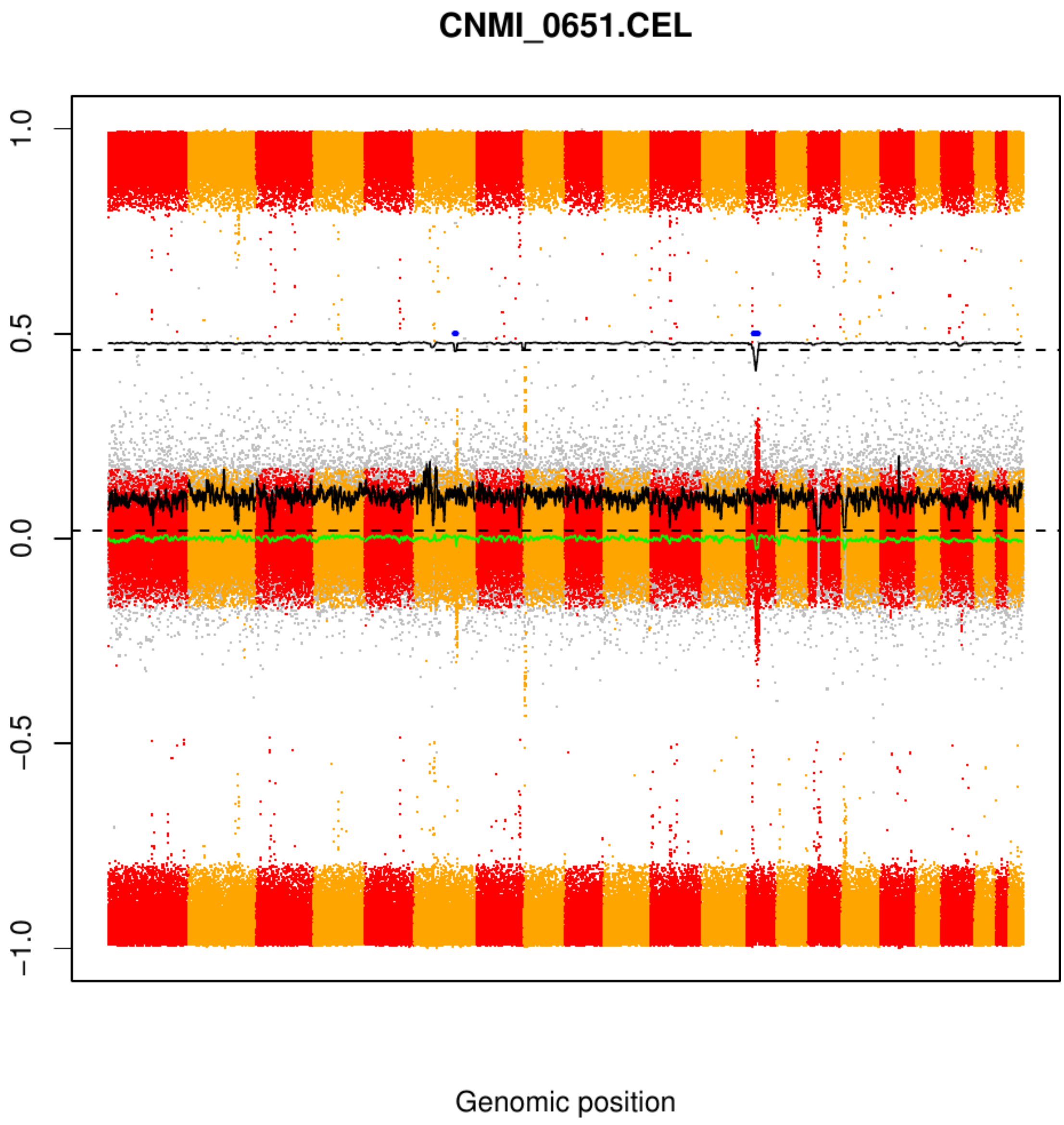
CNMI_0651.CEL
1.0
0.5
0.0
−0.5
−1.0
Genomic position

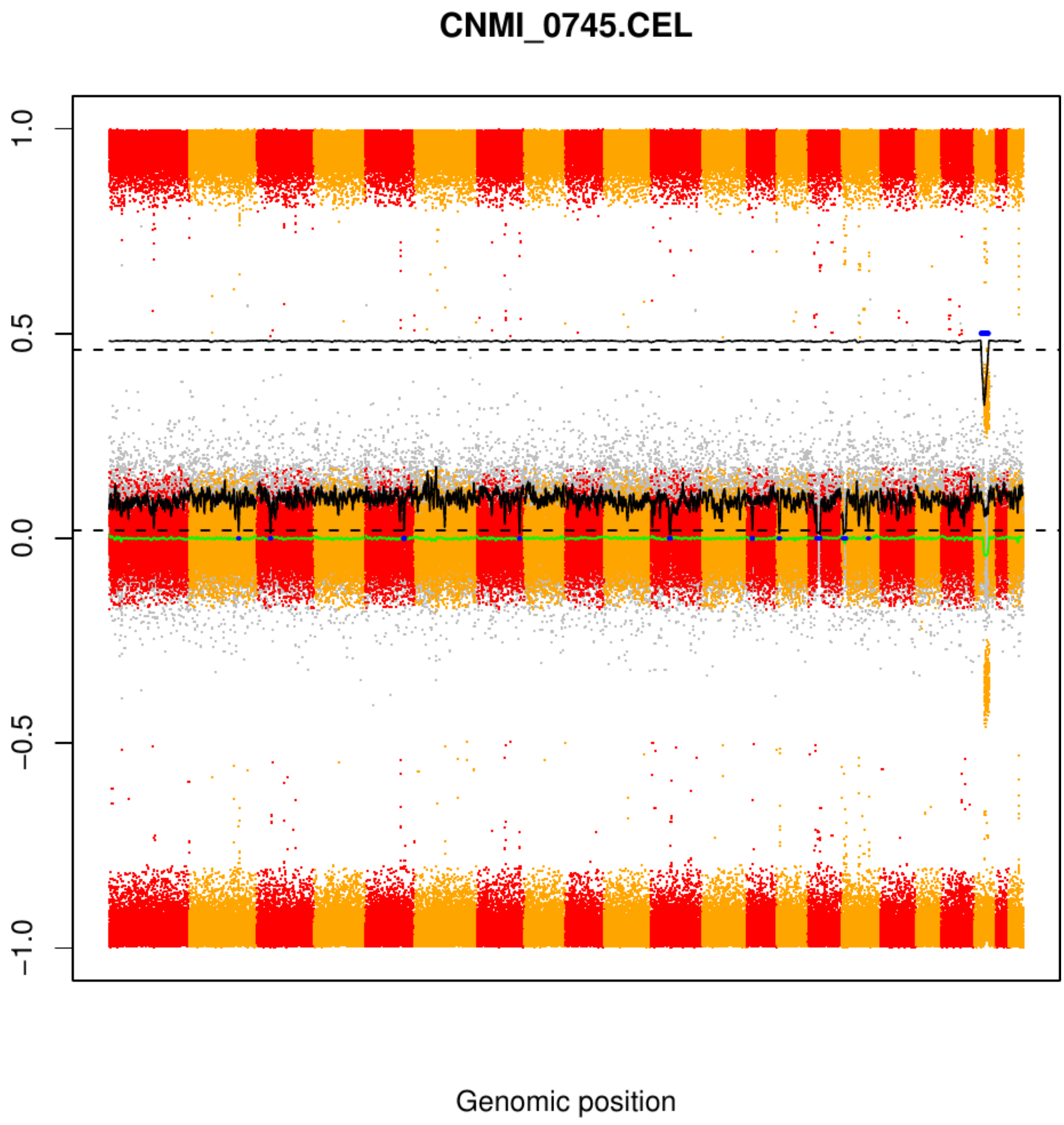
CNMI_0745.CEL
1.0
0.5
0.0
−0.5
−1.0
Genomic position

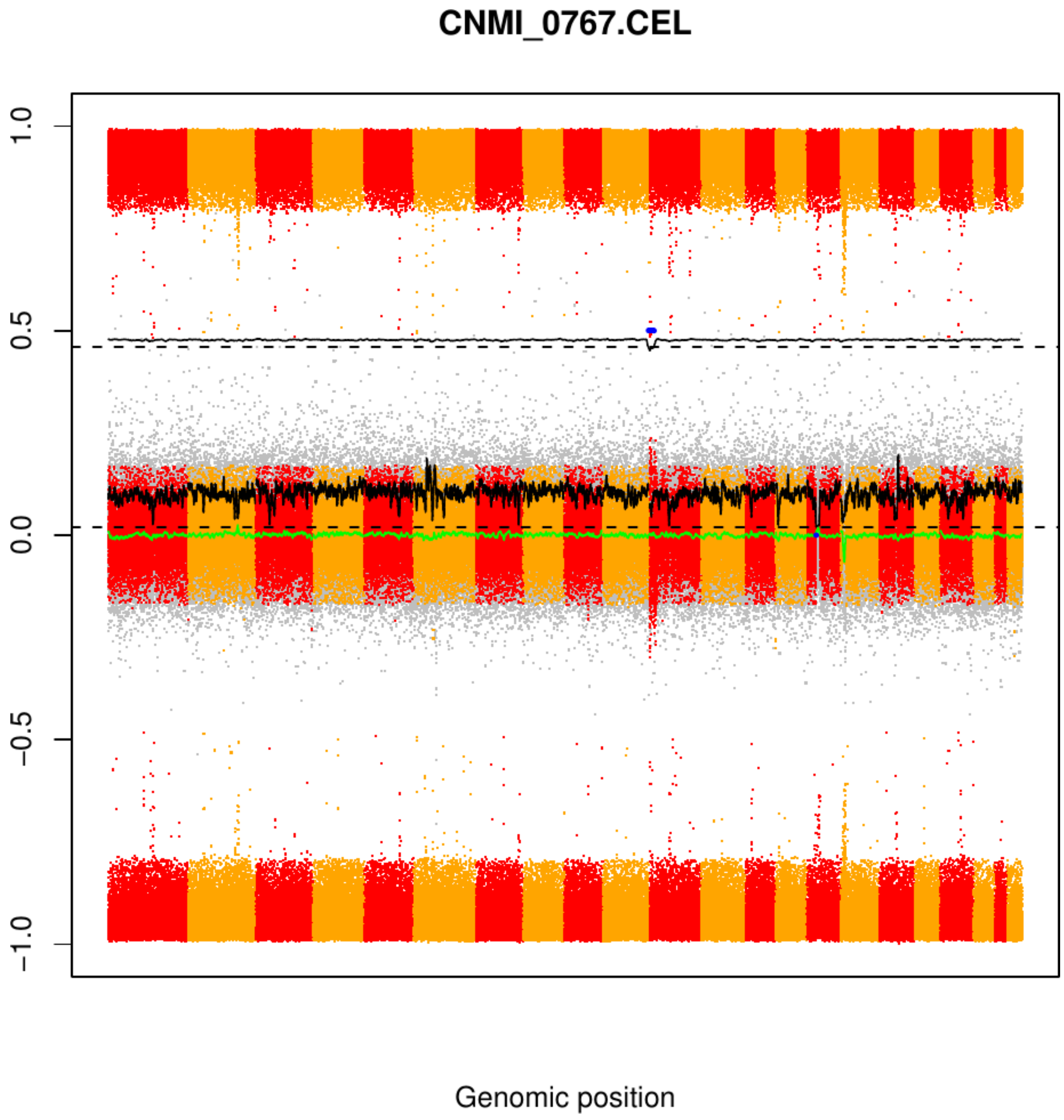

CNMI_0767.CEL
1.0
0.5
0.0
−0.5
−1.0
Genomic position

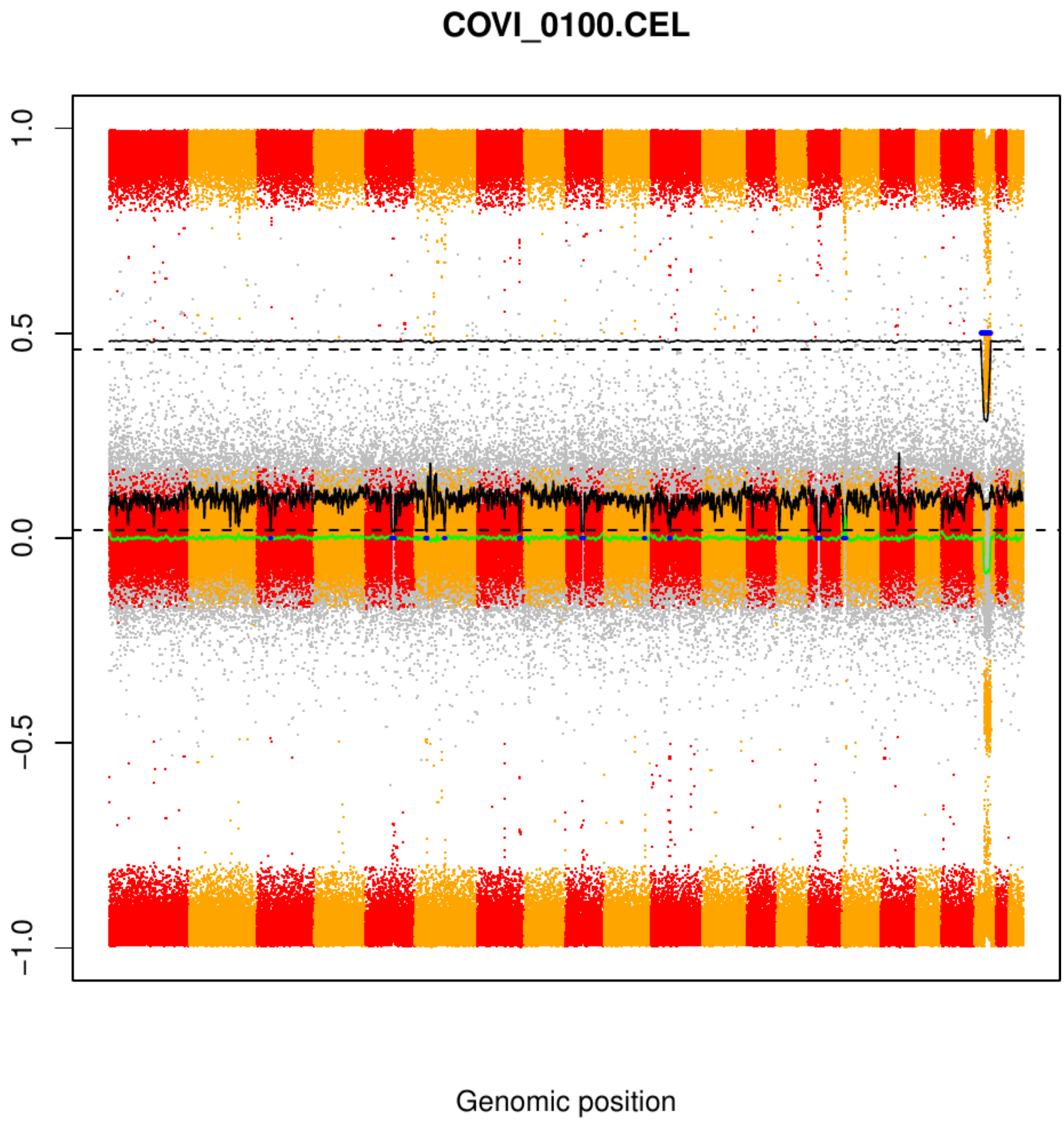

COVI_0100.CEL
1.0
0.5
0.0
−0.5
−1.0
Genomic position

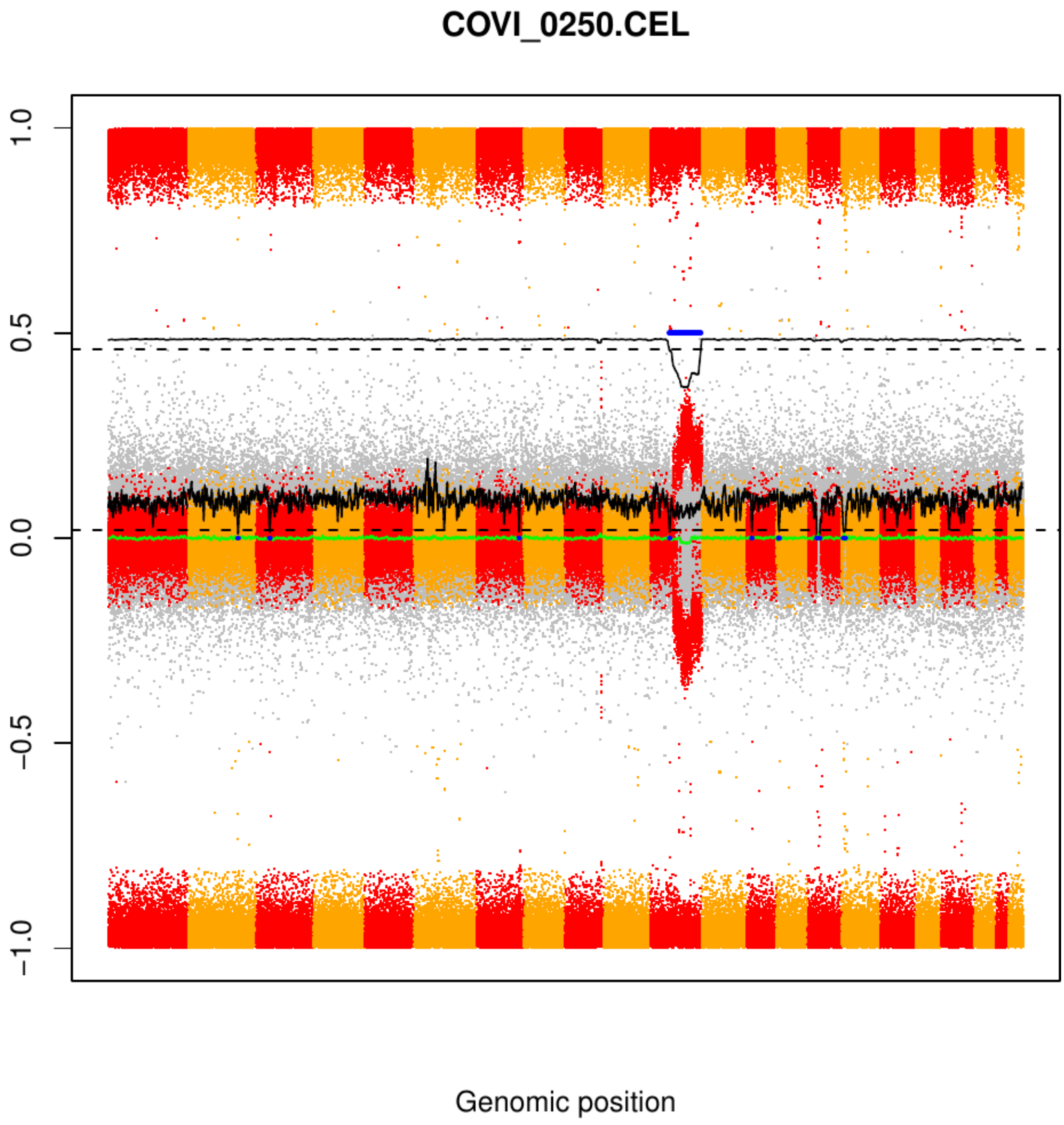
COVI_0250.CEL
1.0
0.5
0.0
−0.5
−1.0
Genomic position

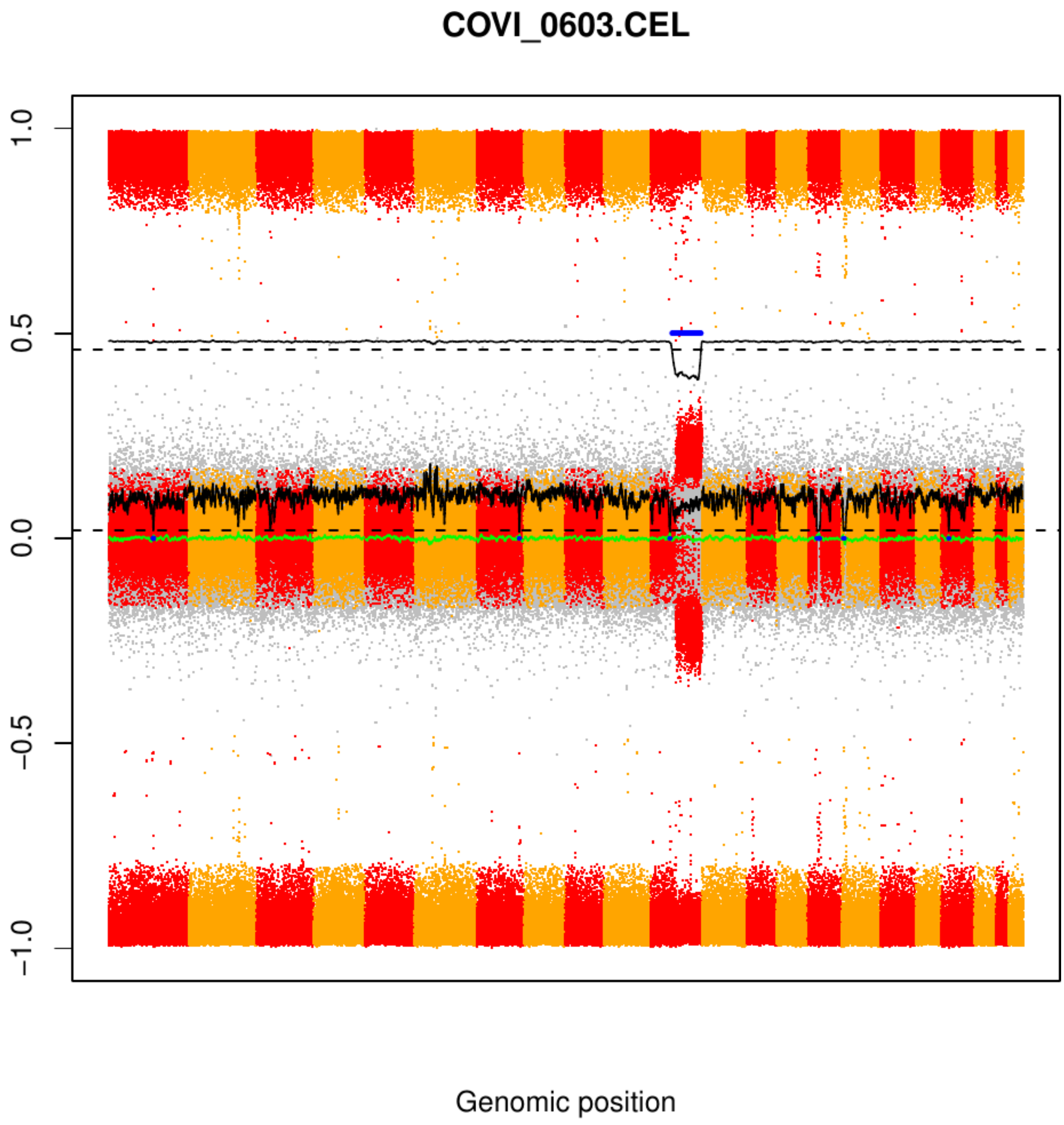
COVI_0603.CEL
1.0
0.5
0.0
−0.5
−1.0
Genomic position

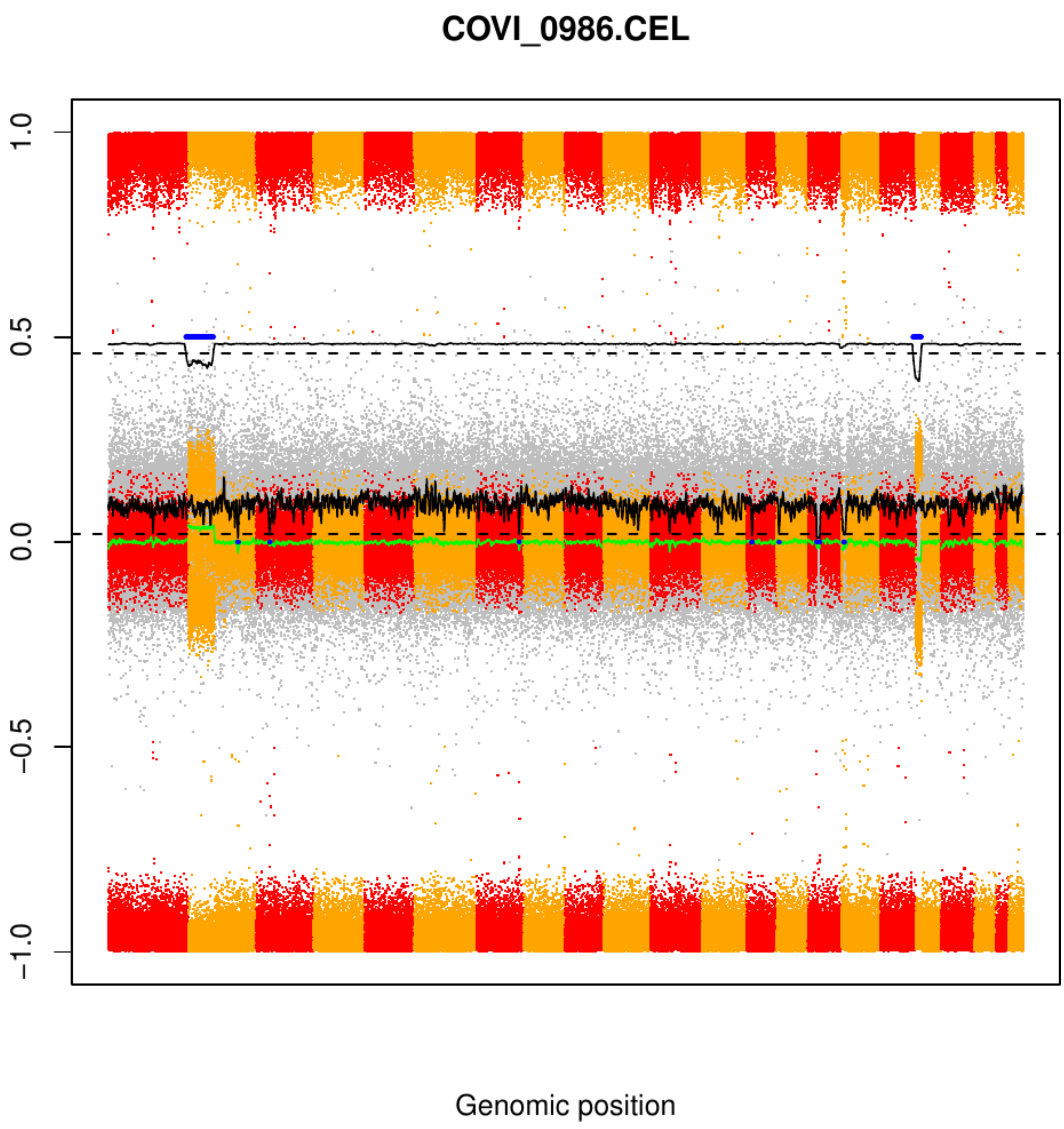
COVI_0986.CEL
1.0
0.5
0.0
−0.5
−1.0
Genomic position

COVI_1116.CEL

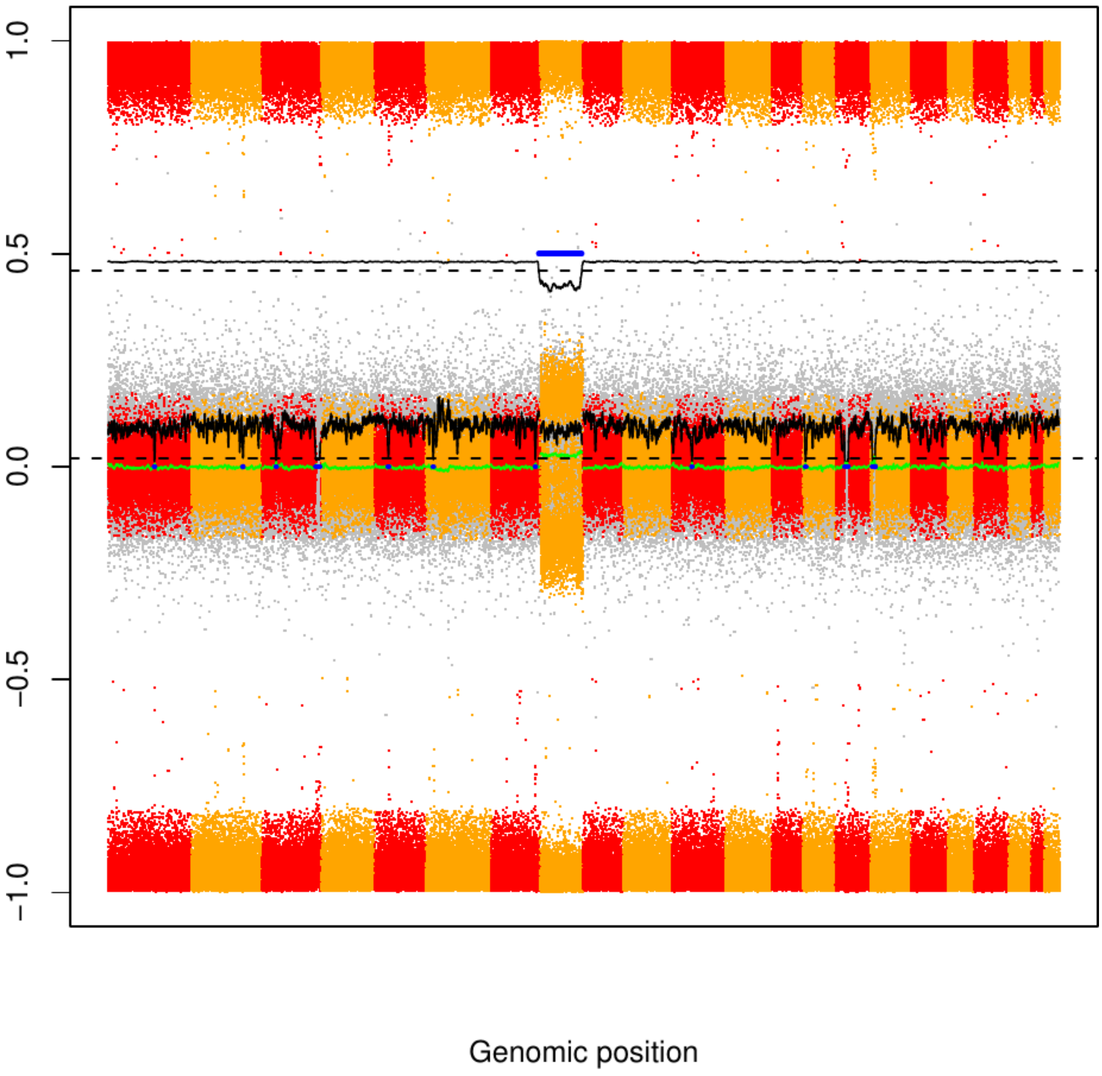

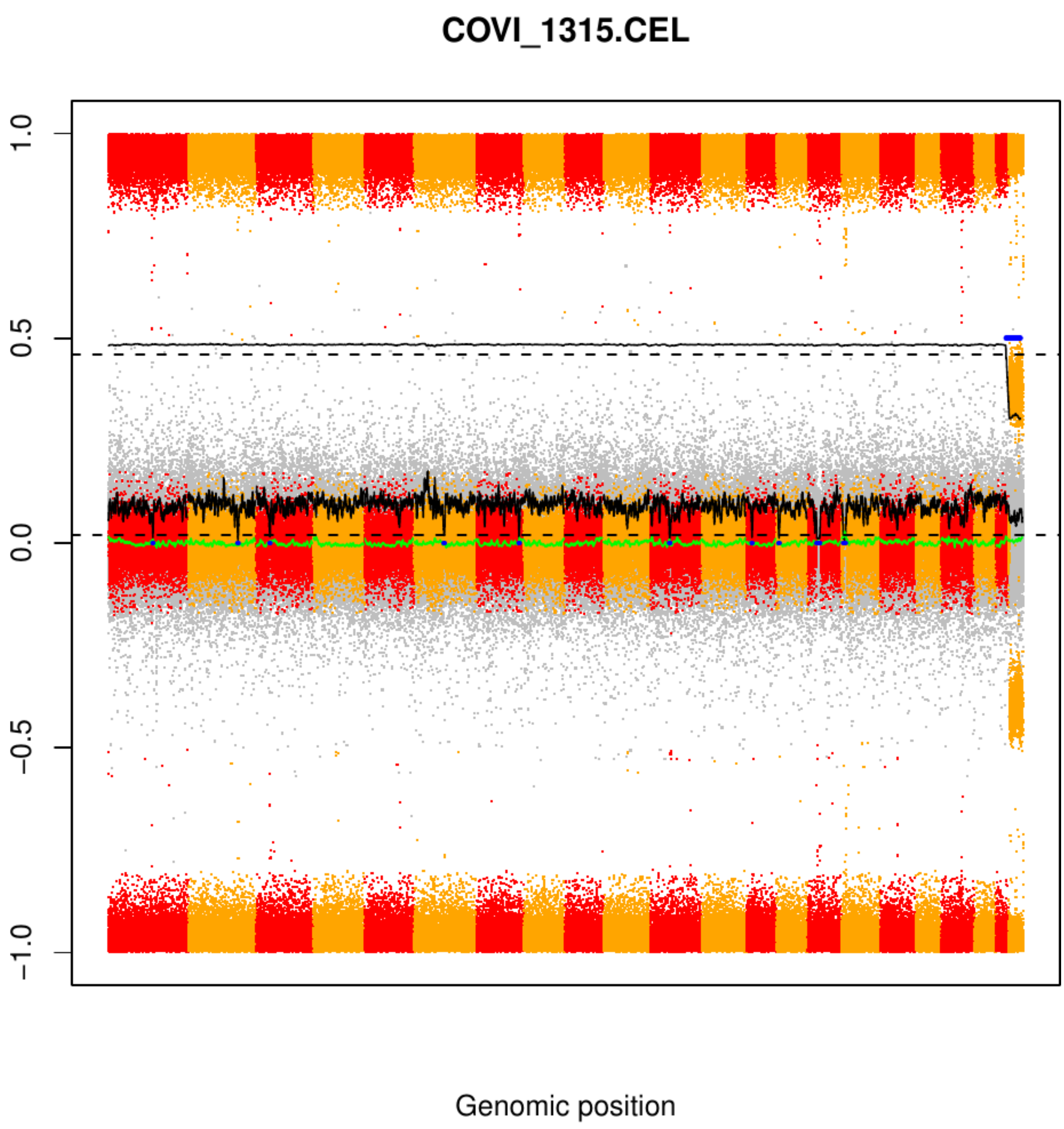
COVI_1315.CEL
1.0
0.5
0.0
−0.5
−1.0
Genomic position

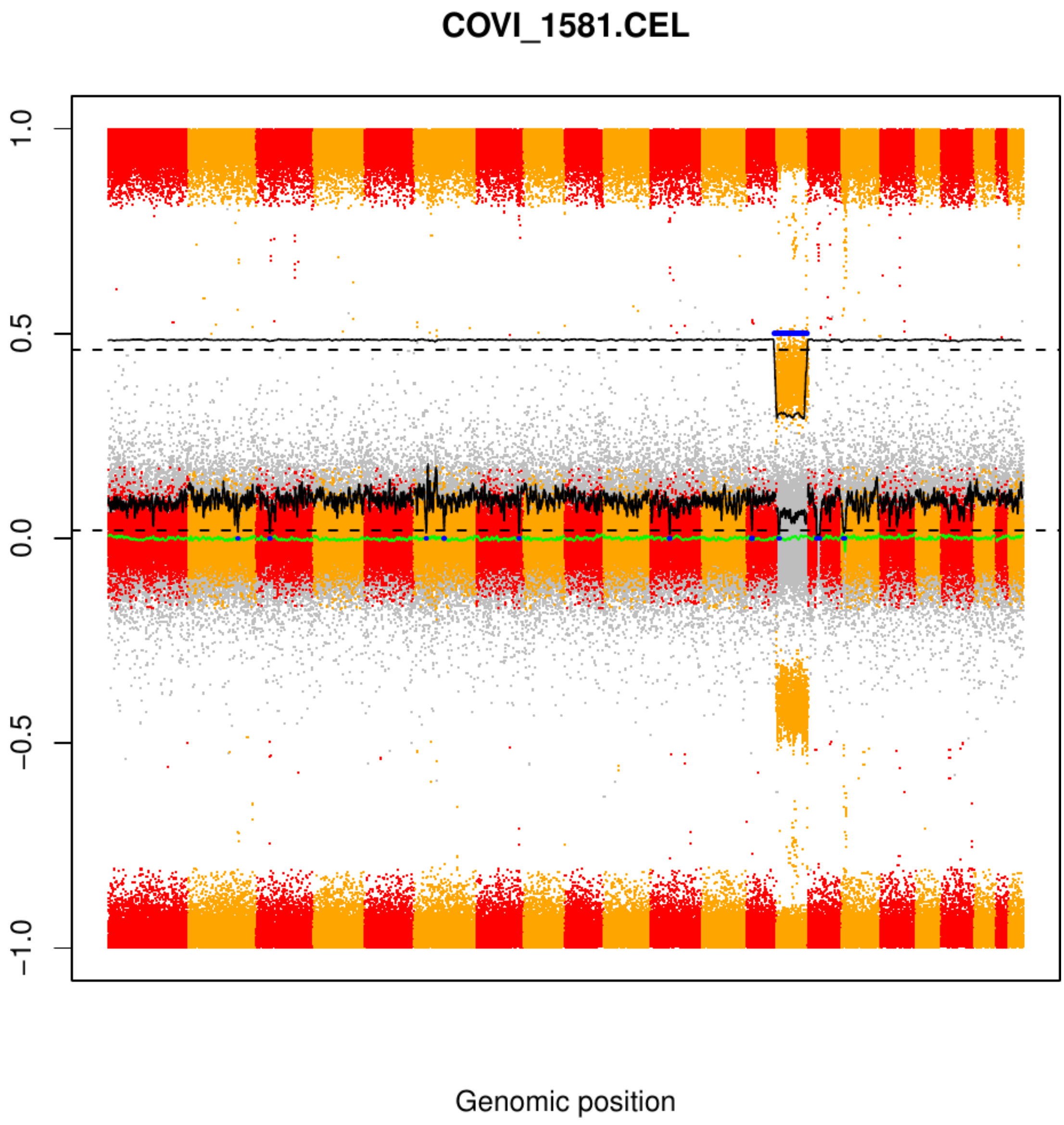

COVI_1581.CEL
1.0
0.5
0.0
−0.5
−1.0
Genomic position

FJD_0077.CEL

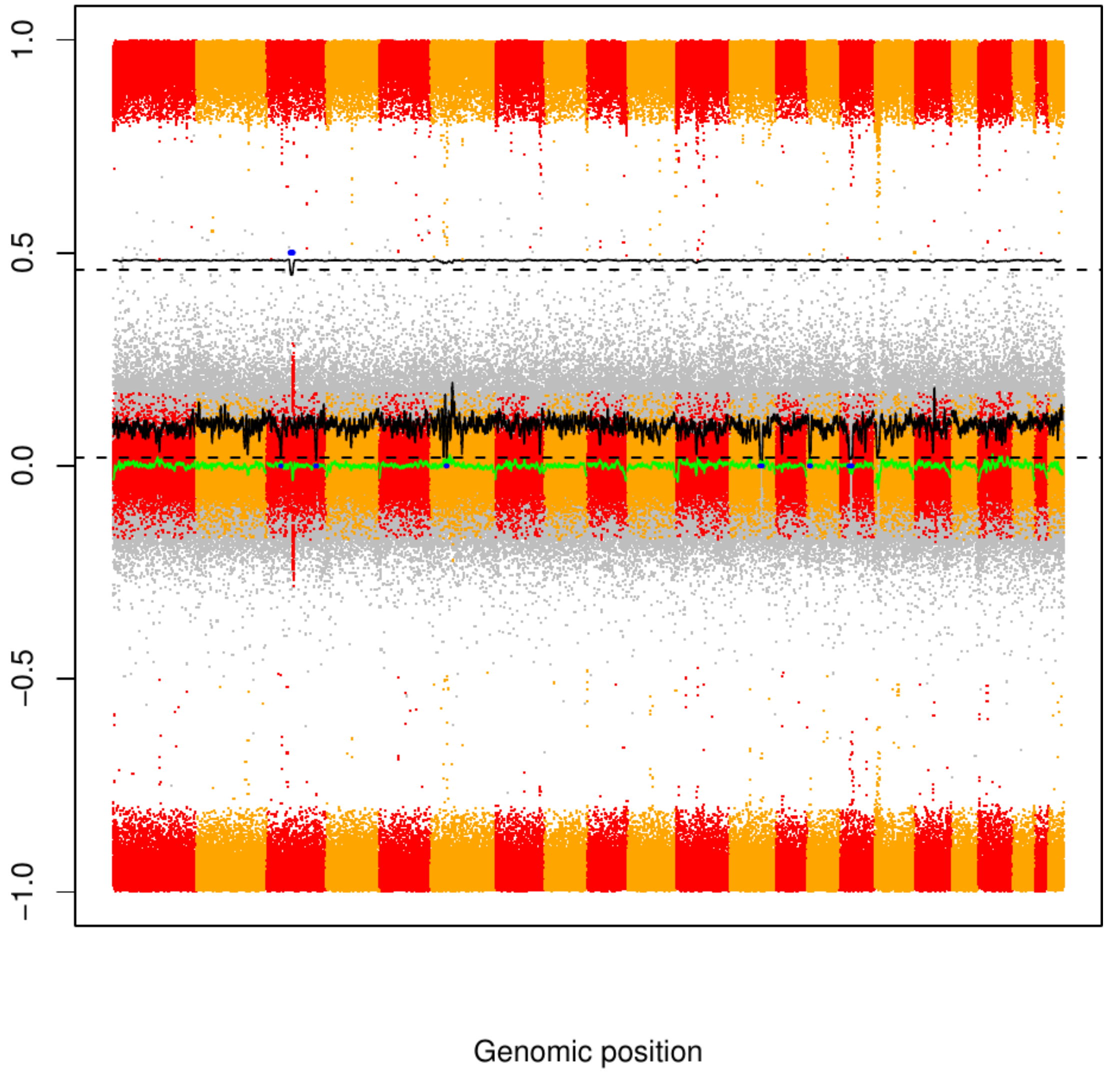

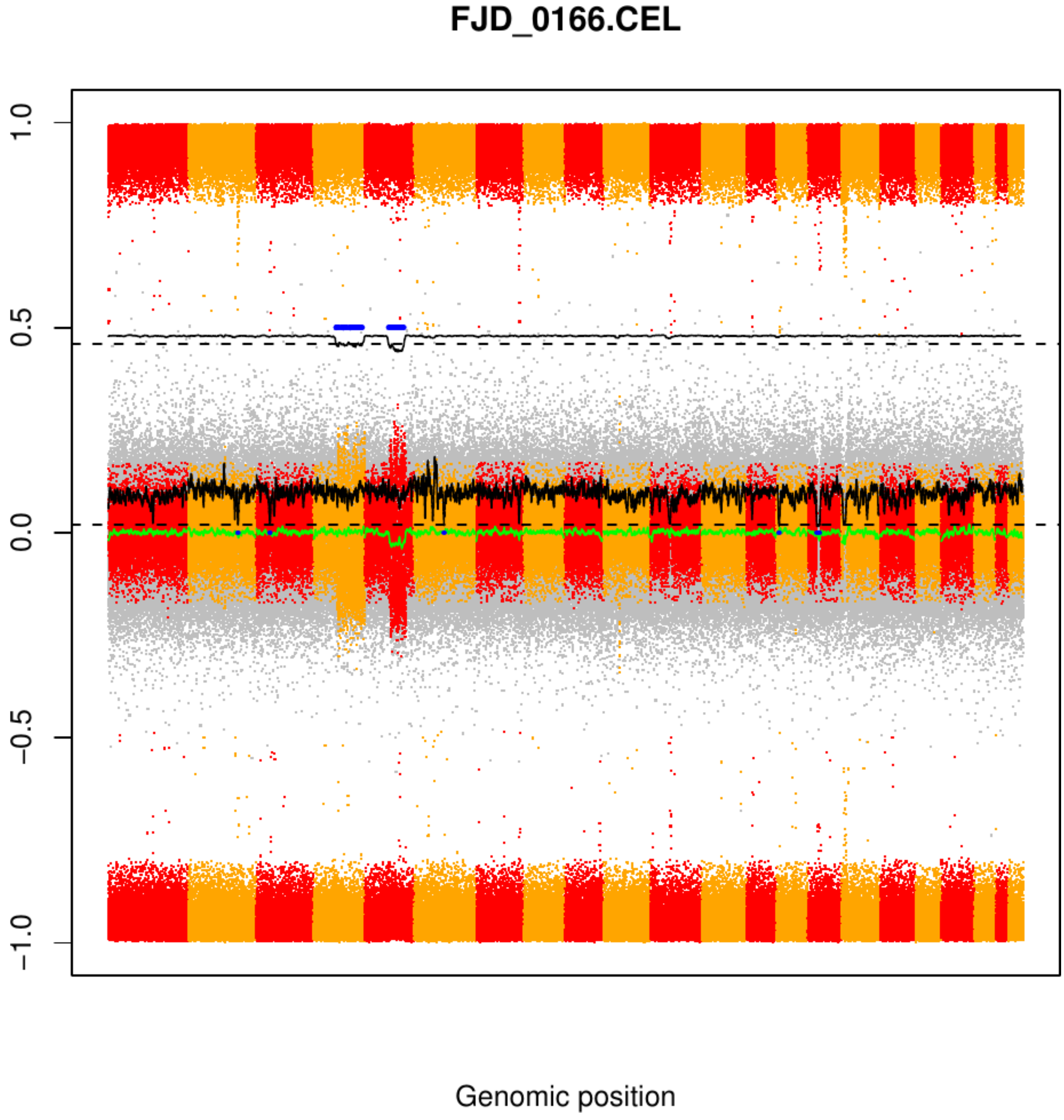
FJD_0166.CEL
1.0
0.5
0.0
−0.5
−1.0
Genomic position

FJD_0202.CEL

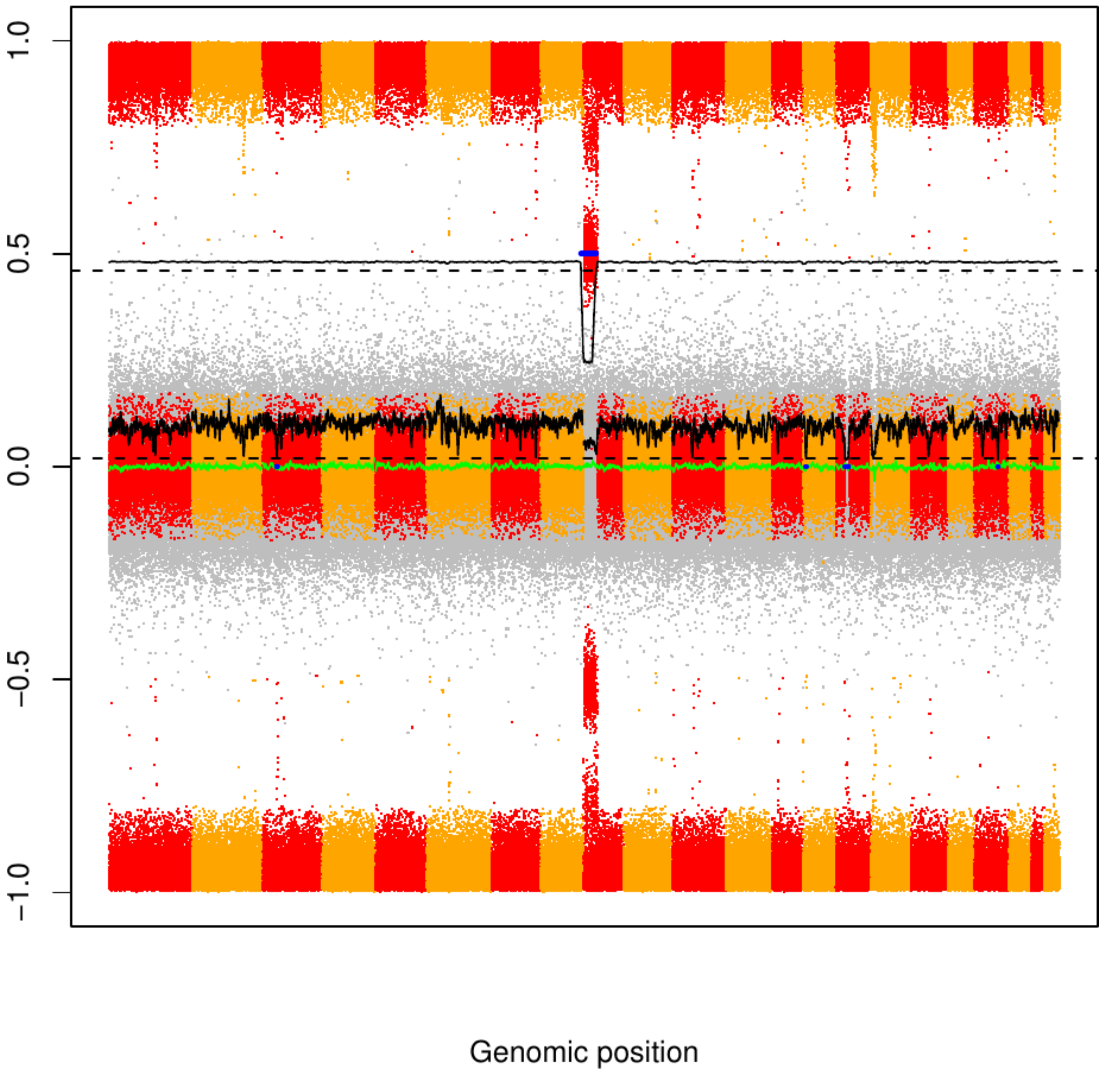

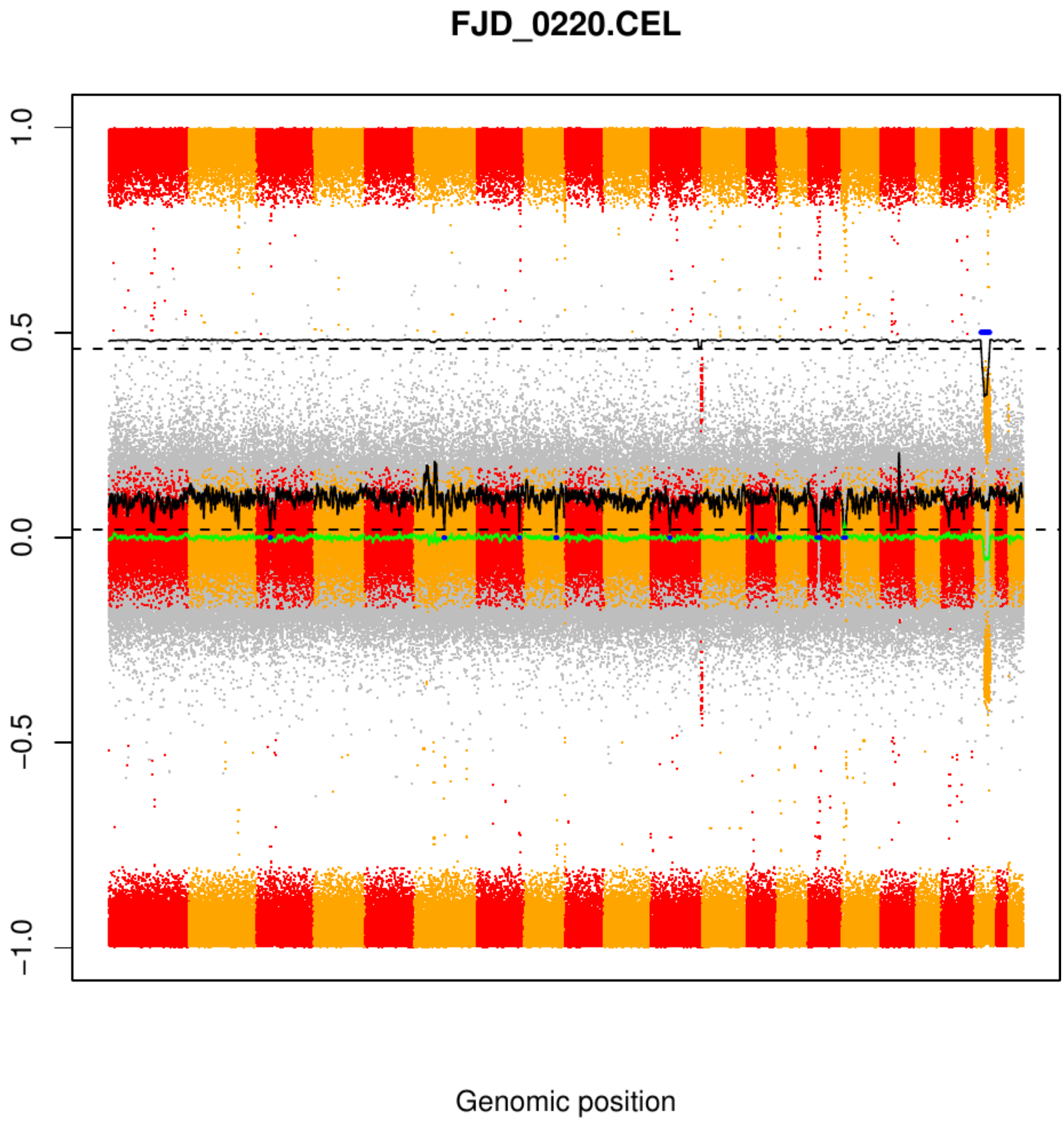
FJD_0220.CEL
1.0
0.5
0.0
−0.5
−1.0
Genomic position

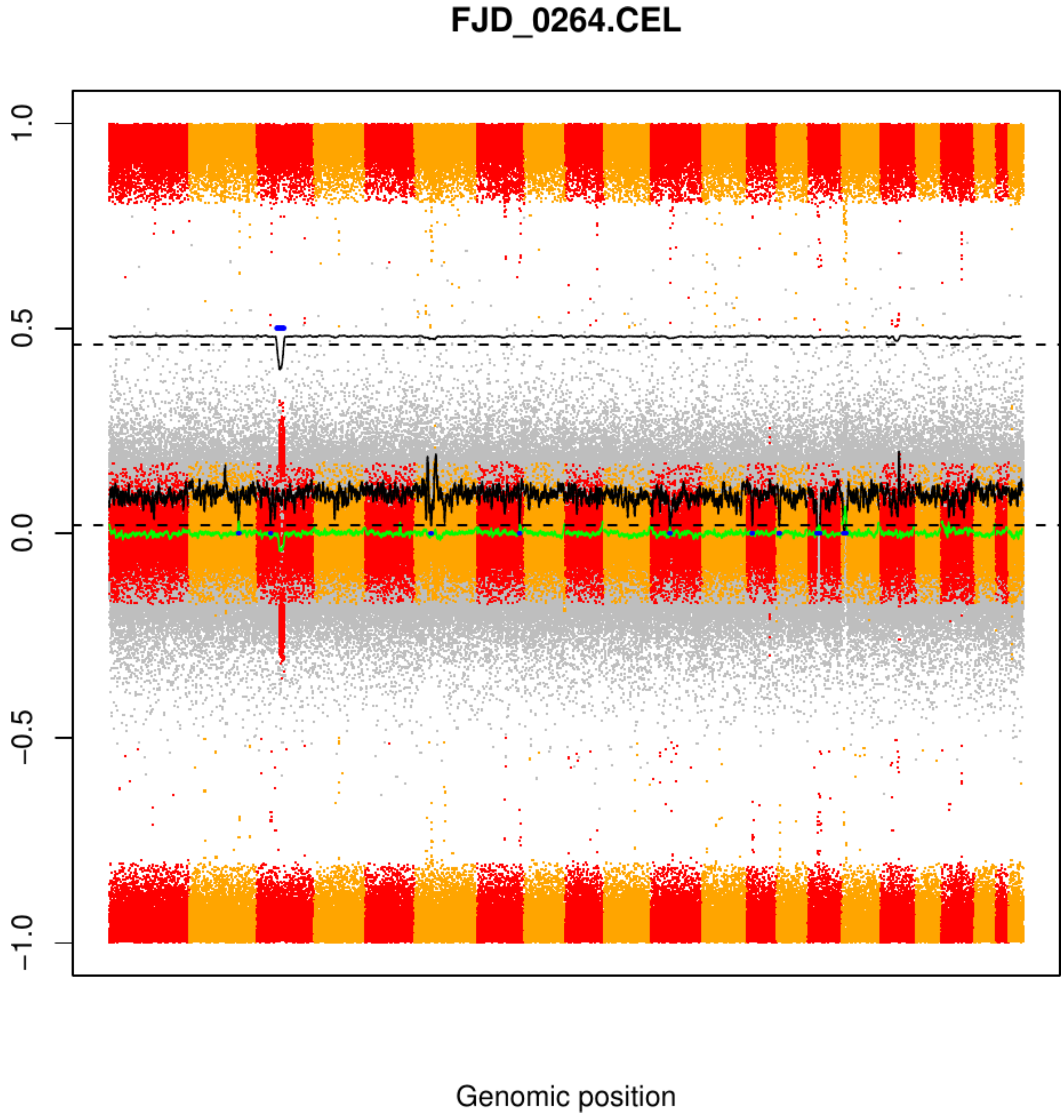

FJD_0264.CEL
1.0
0.5
0.0
−0.5
−1.0
Genomic position

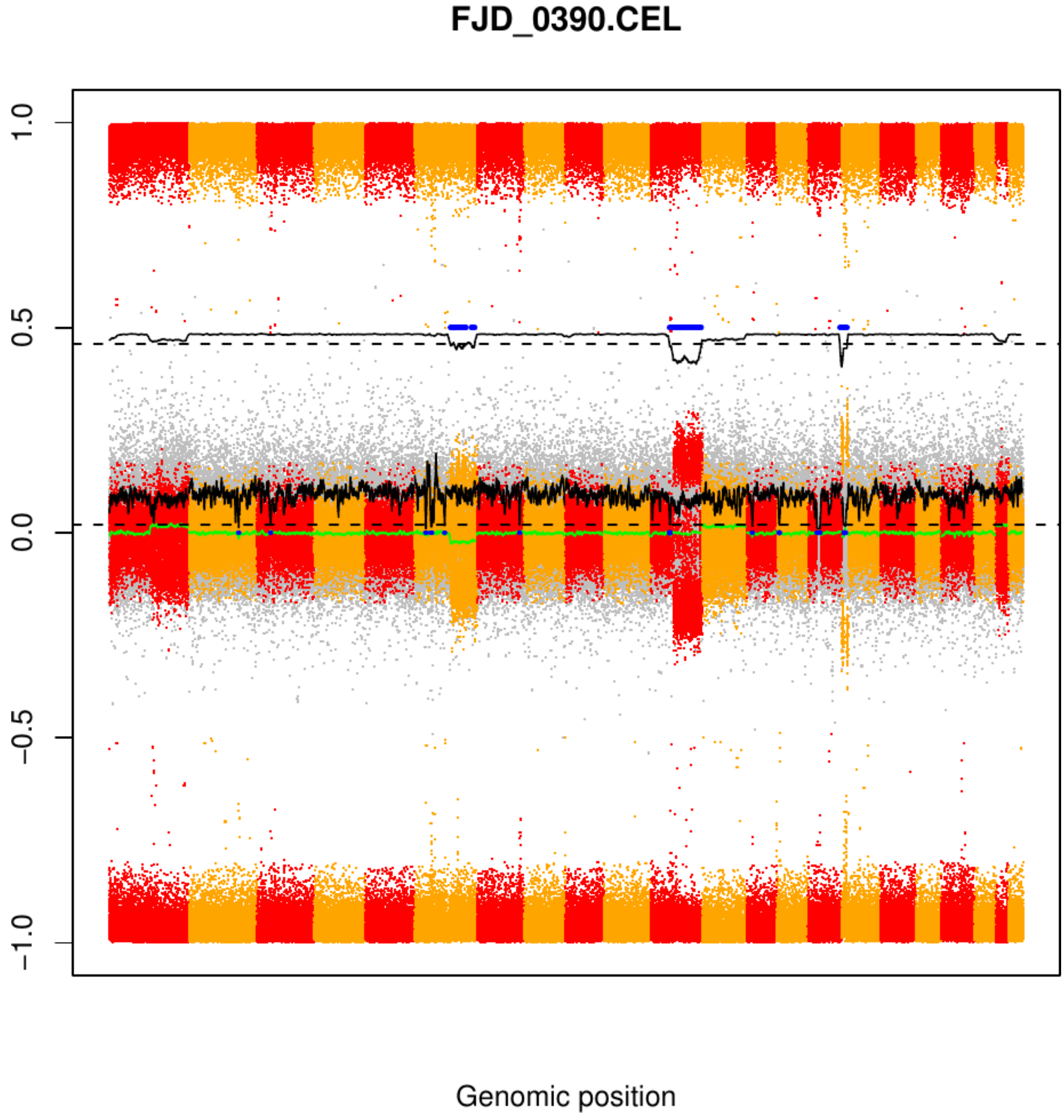
FJD_0390.CEL
1.0
0.5
0.0
−0.5
−1.0
Genomic position

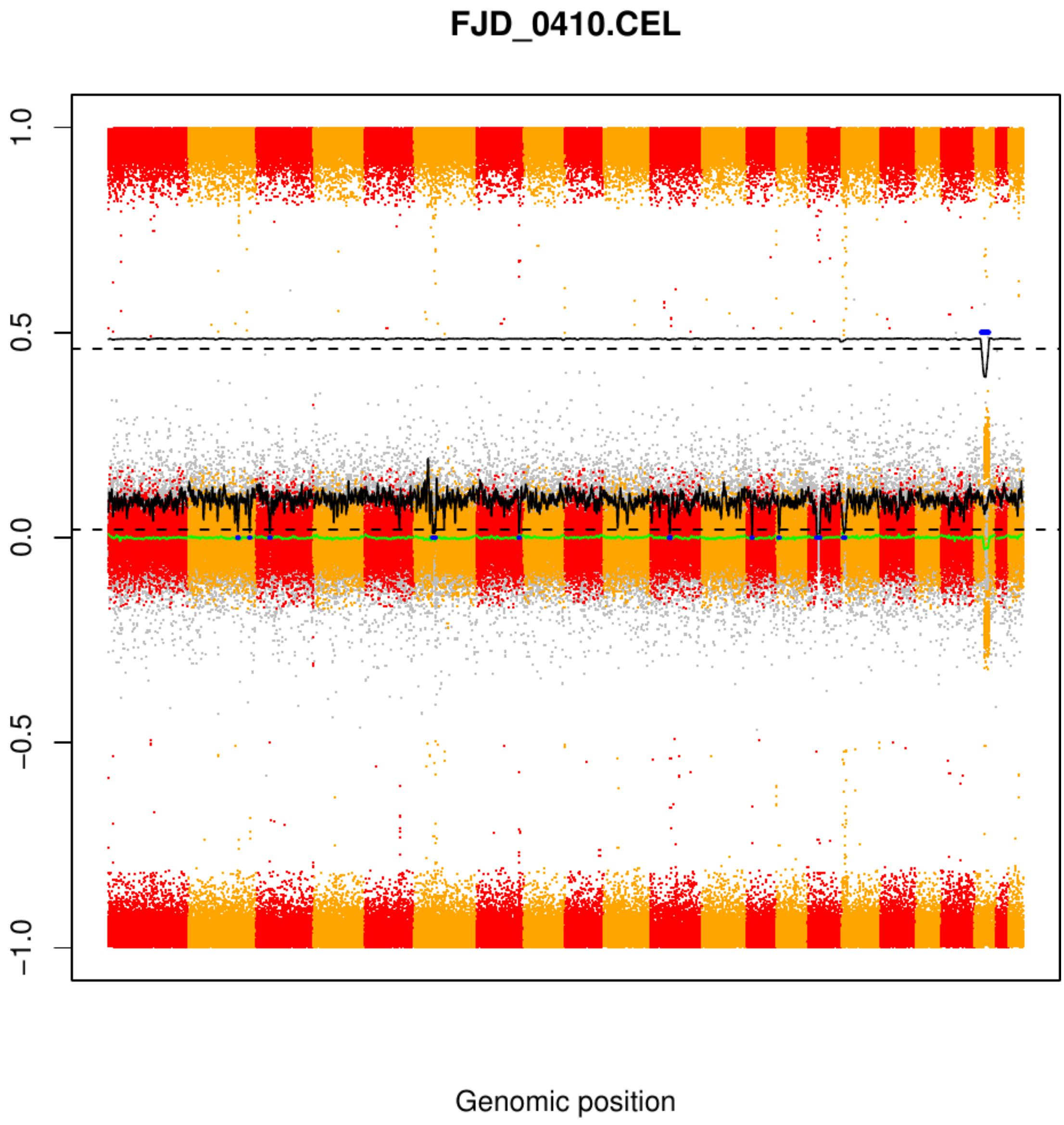

FJD_0410.CEL
1.0
0.5
0.0
−0.5
−1.0
Genomic position

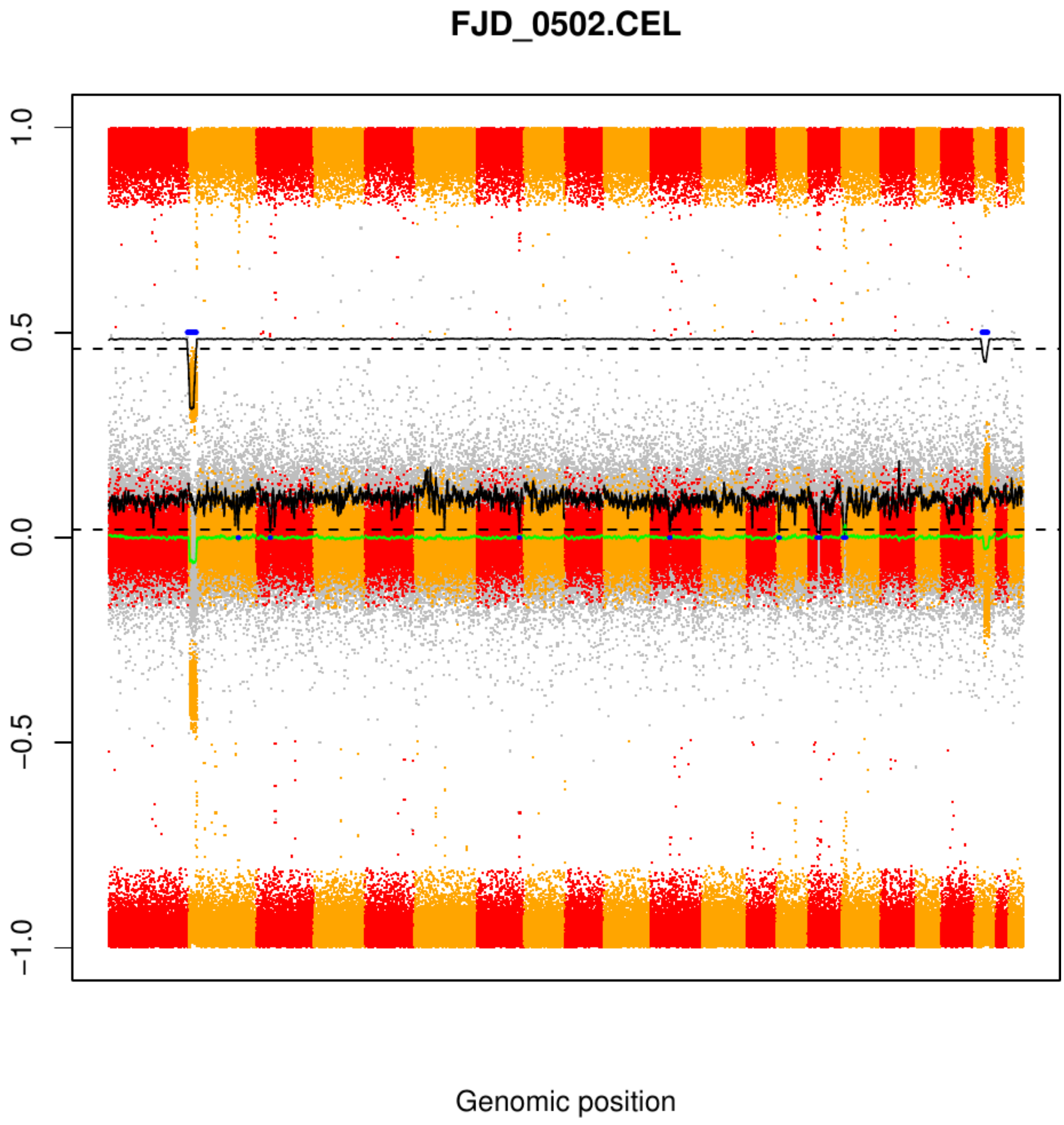
FJD_0502.CEL
1.0
0.5
0.0
−0.5
−1.0
Genomic position

FJD_0675.CEL

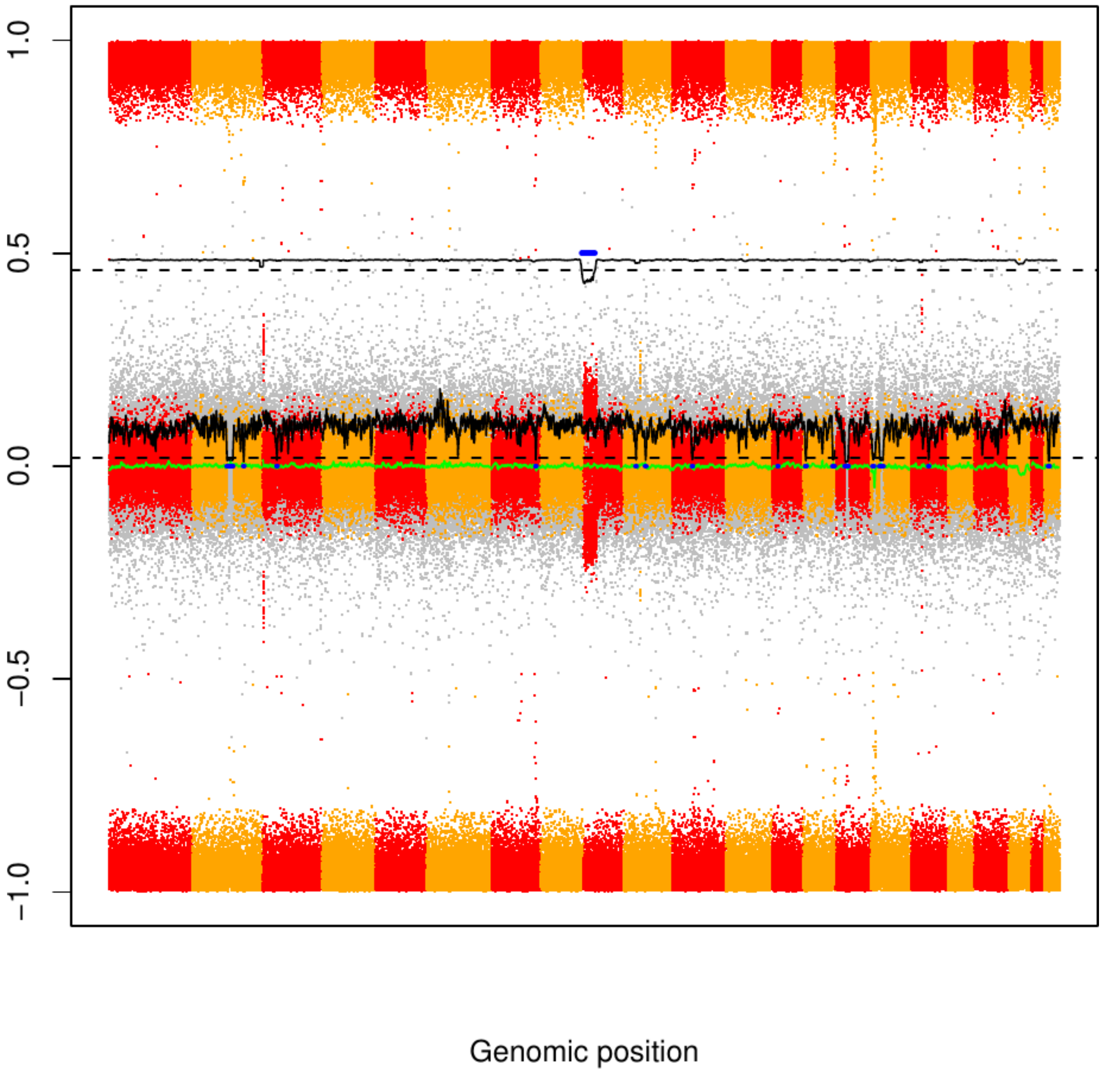

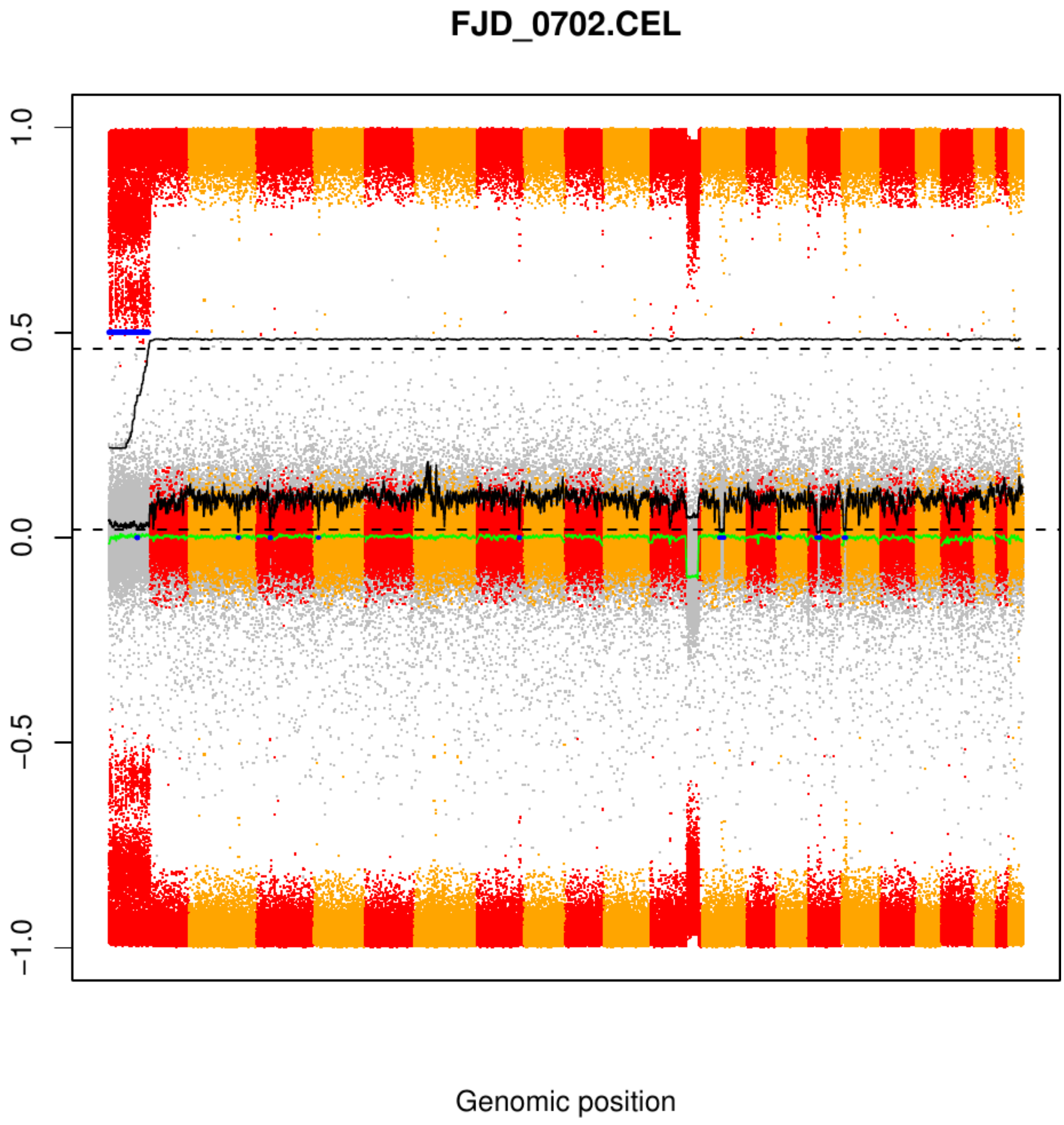

FJD_0702.CEL
1.0
0.5
0.0
−0.5
−1.0
Genomic position

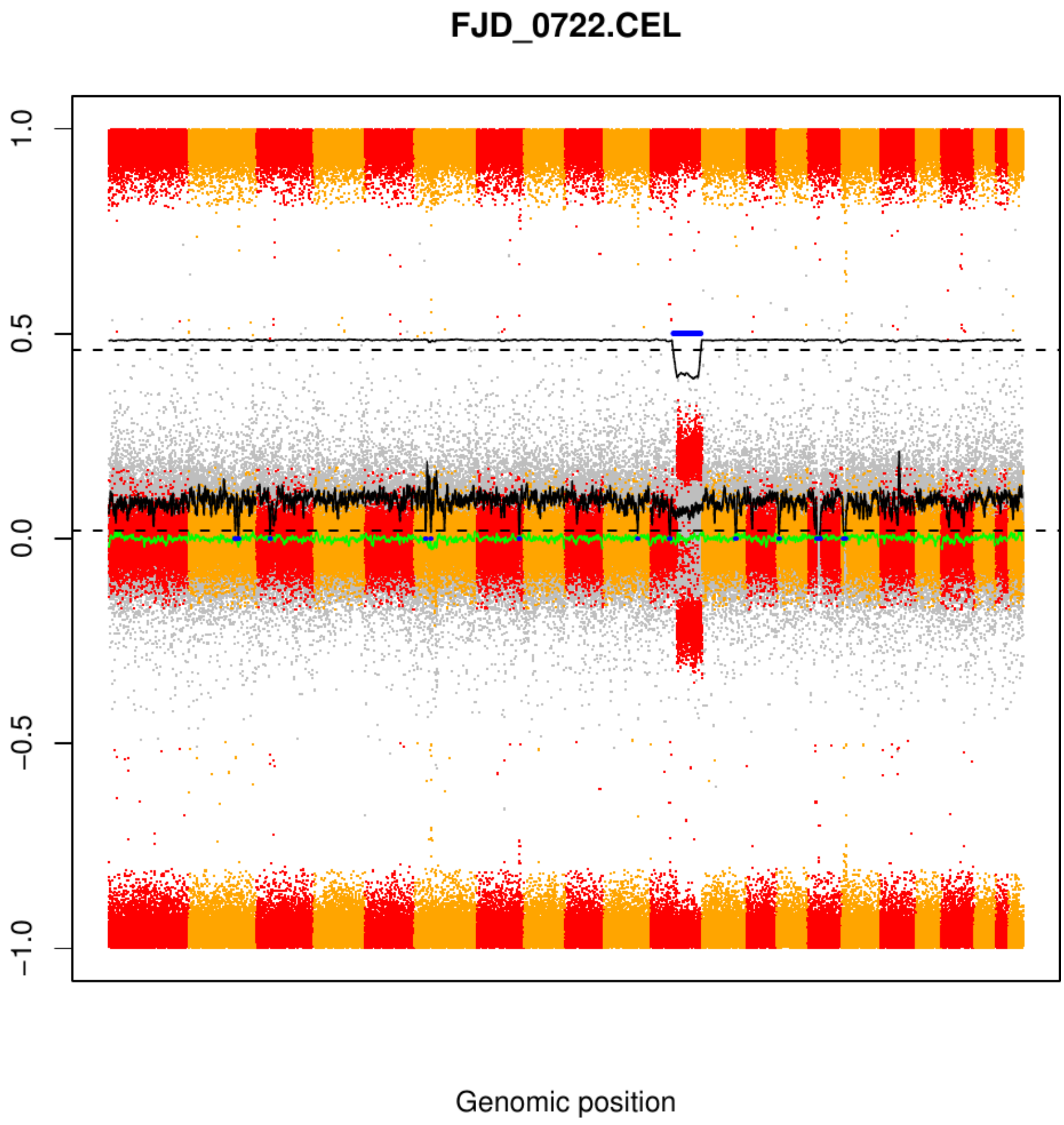
FJD_0722.CEL
1.0
0.5
0.0
−0.5
−1.0
Genomic position

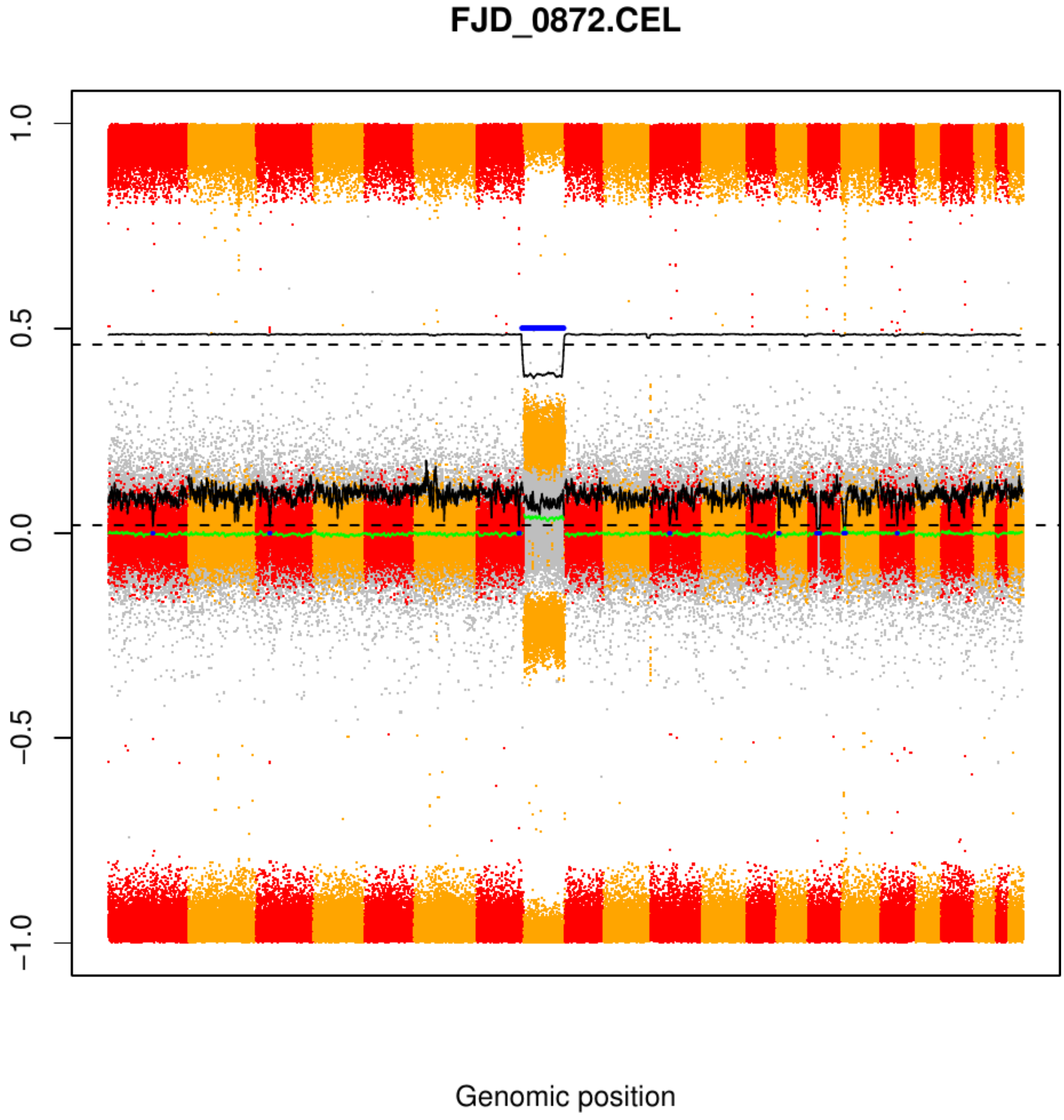
FJD_0872.CEL
1.0
0.5
0.0
−0.5
−1.0
Genomic position

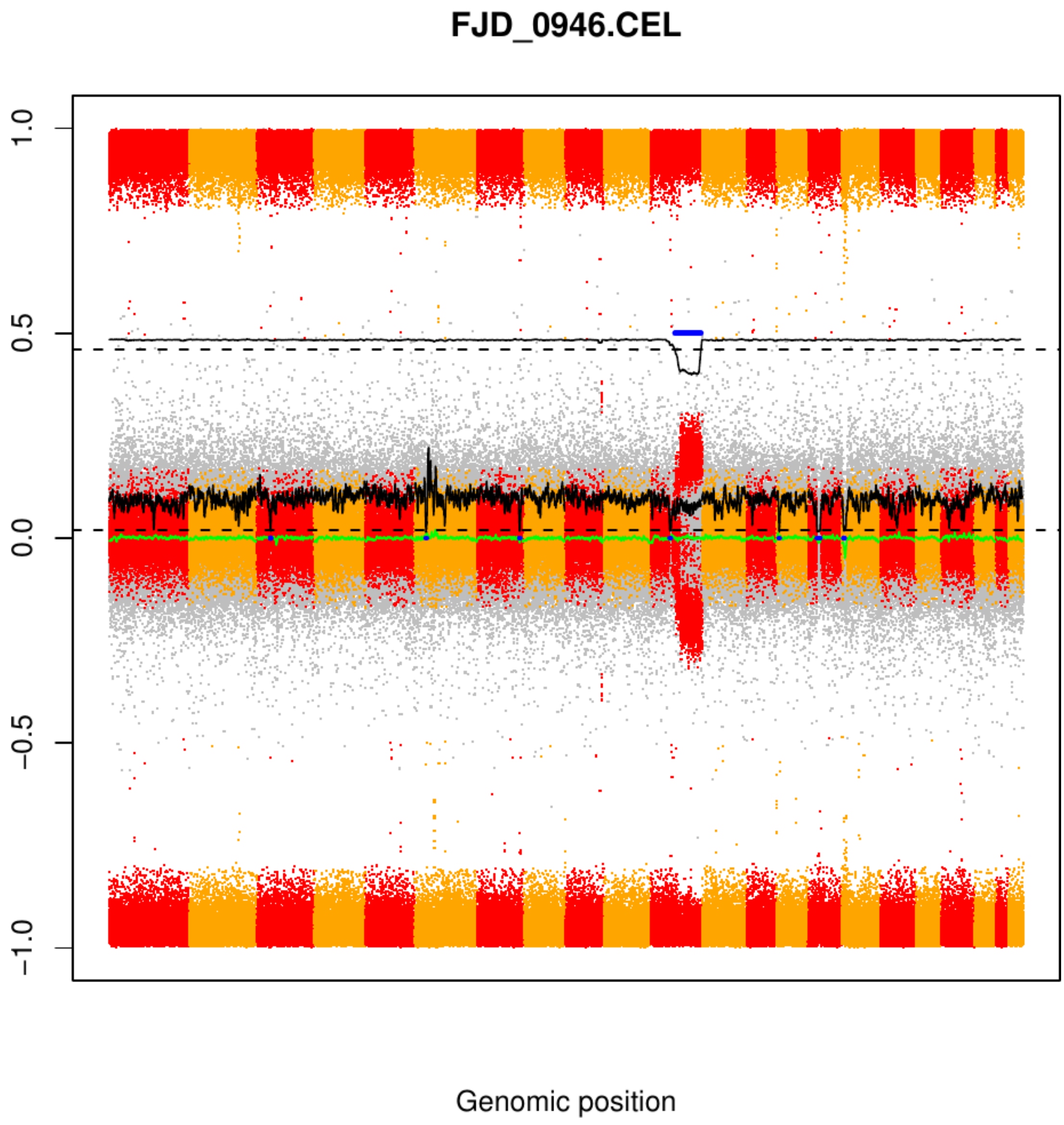

FJD_0946.CEL
1.0
0.5
0.0
−0.5
−1.0
Genomic position

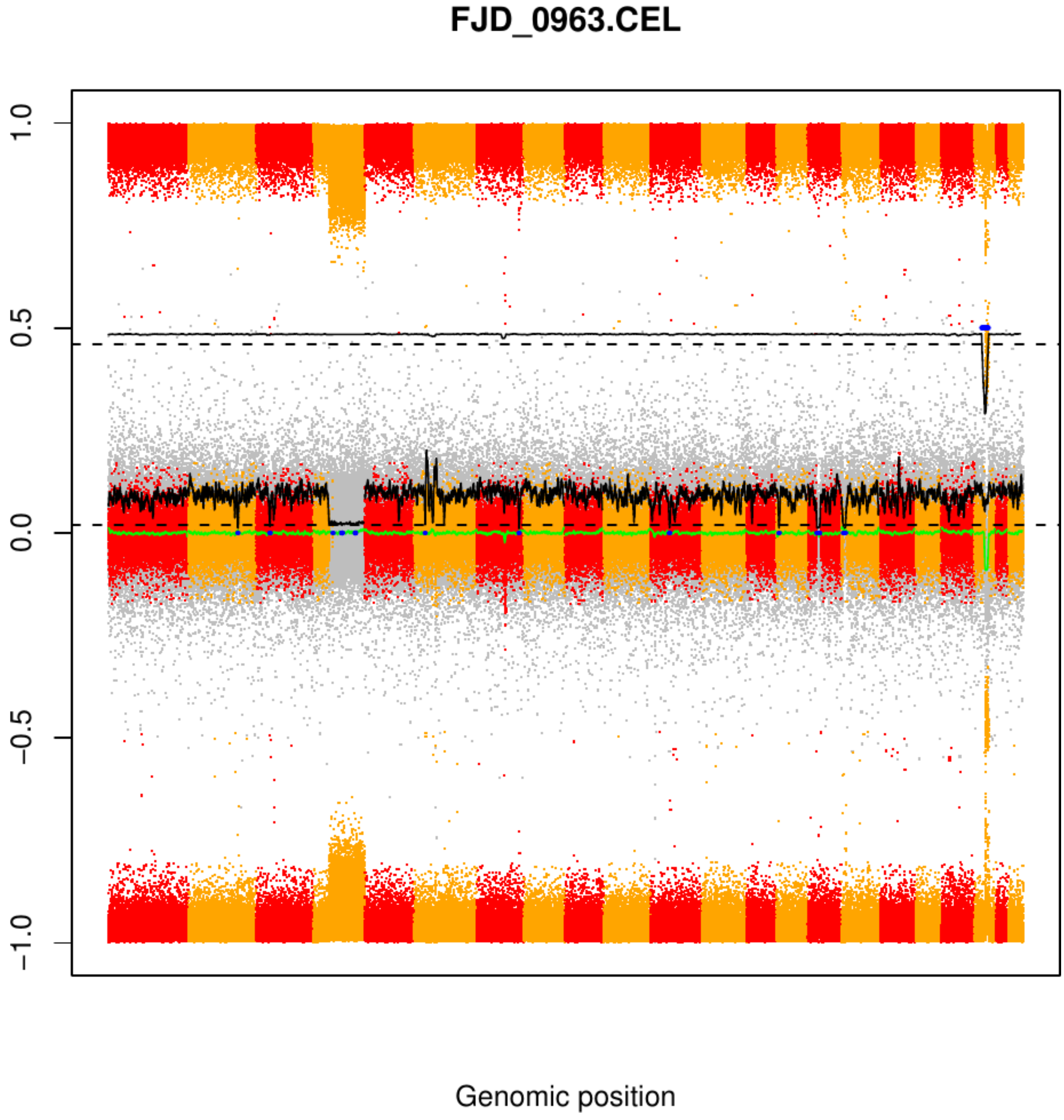
FJD_0963.CEL
1.0
0.5
0.0
−0.5
−1.0
Genomic position

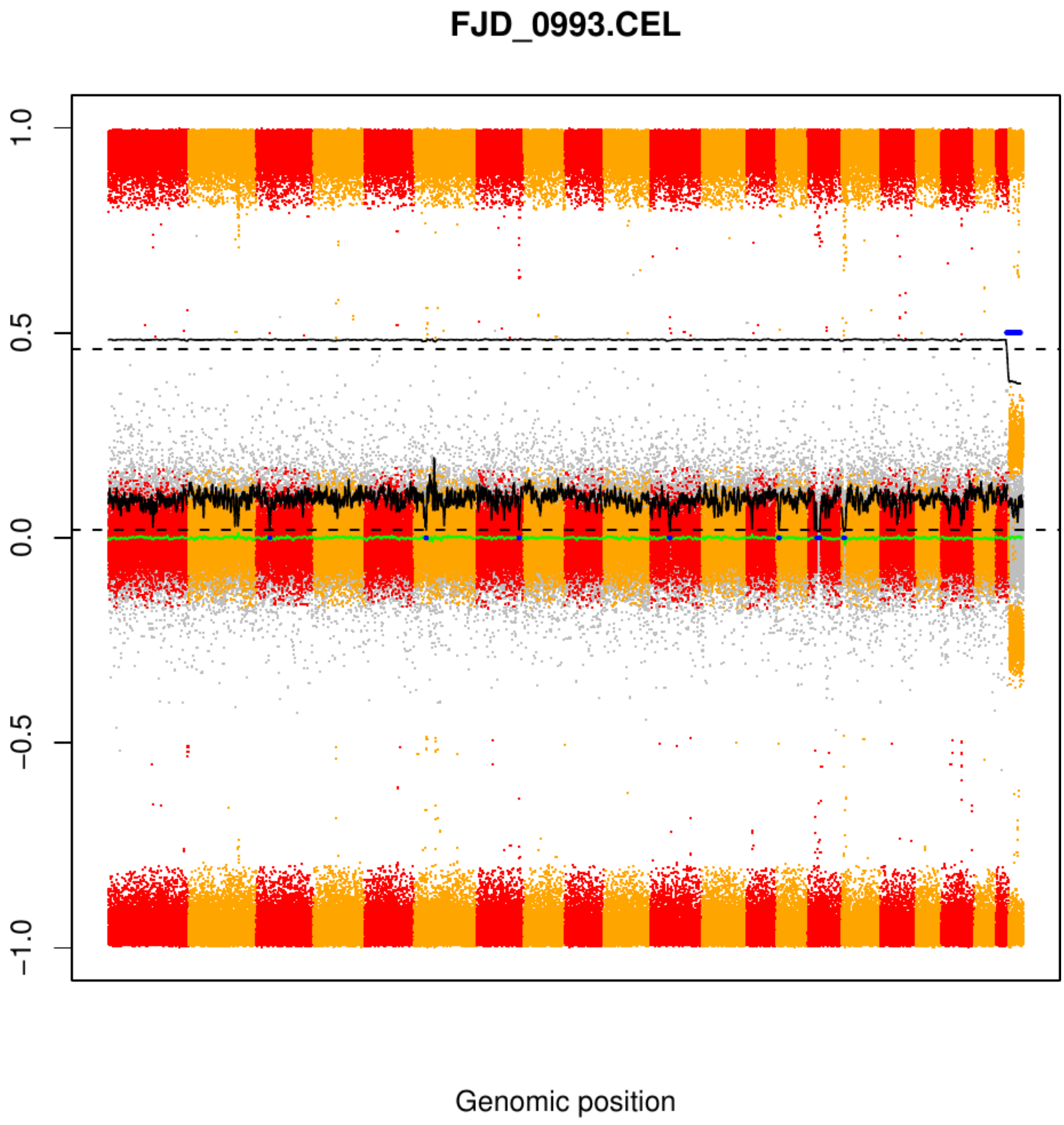
FJD_0993.CEL
1.0
0.5
0.0
−0.5
−1.0
Genomic position

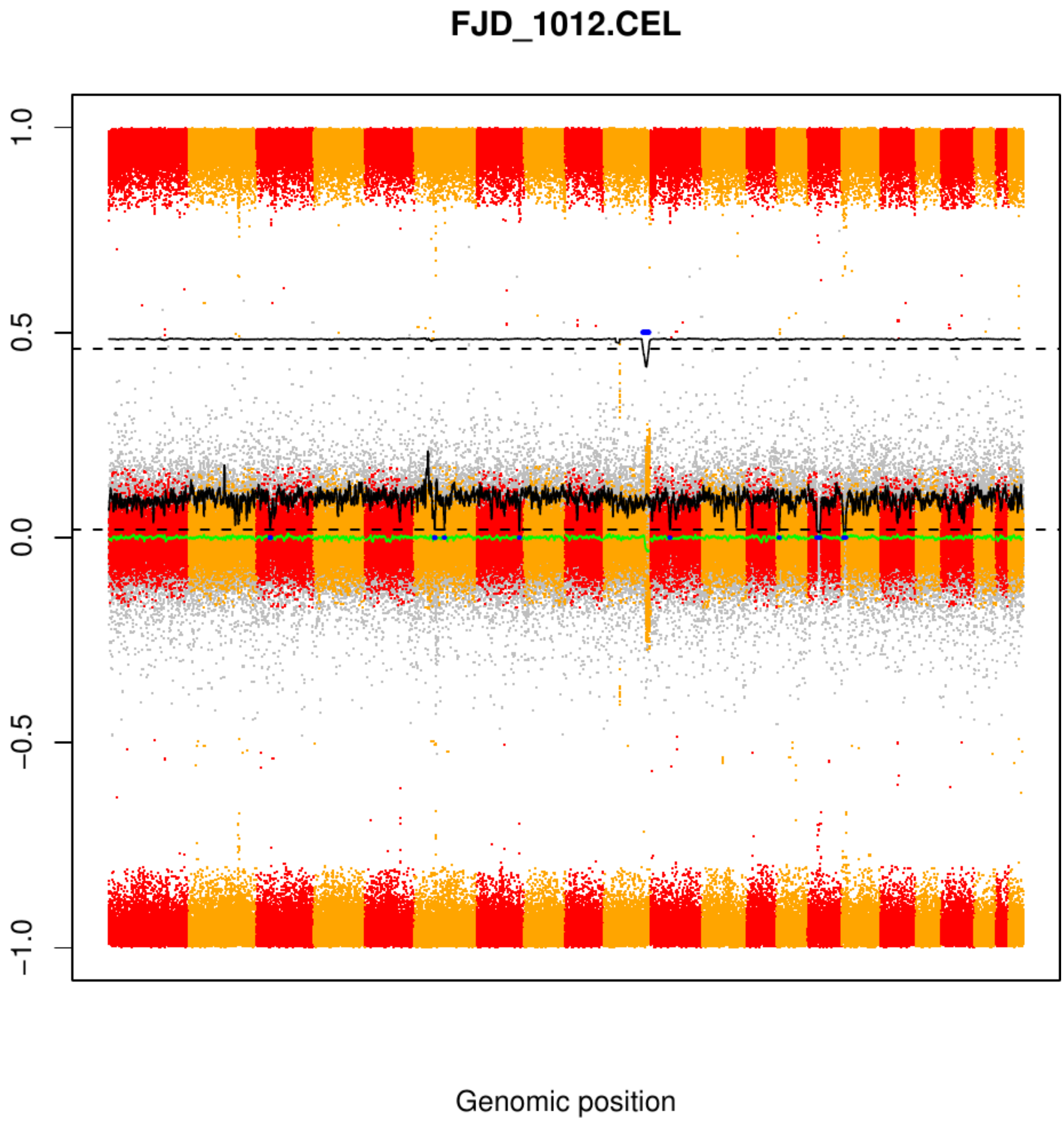

FJD_1012.CEL
1.0
0.5
0.0
−0.5
−1.0
Genomic position

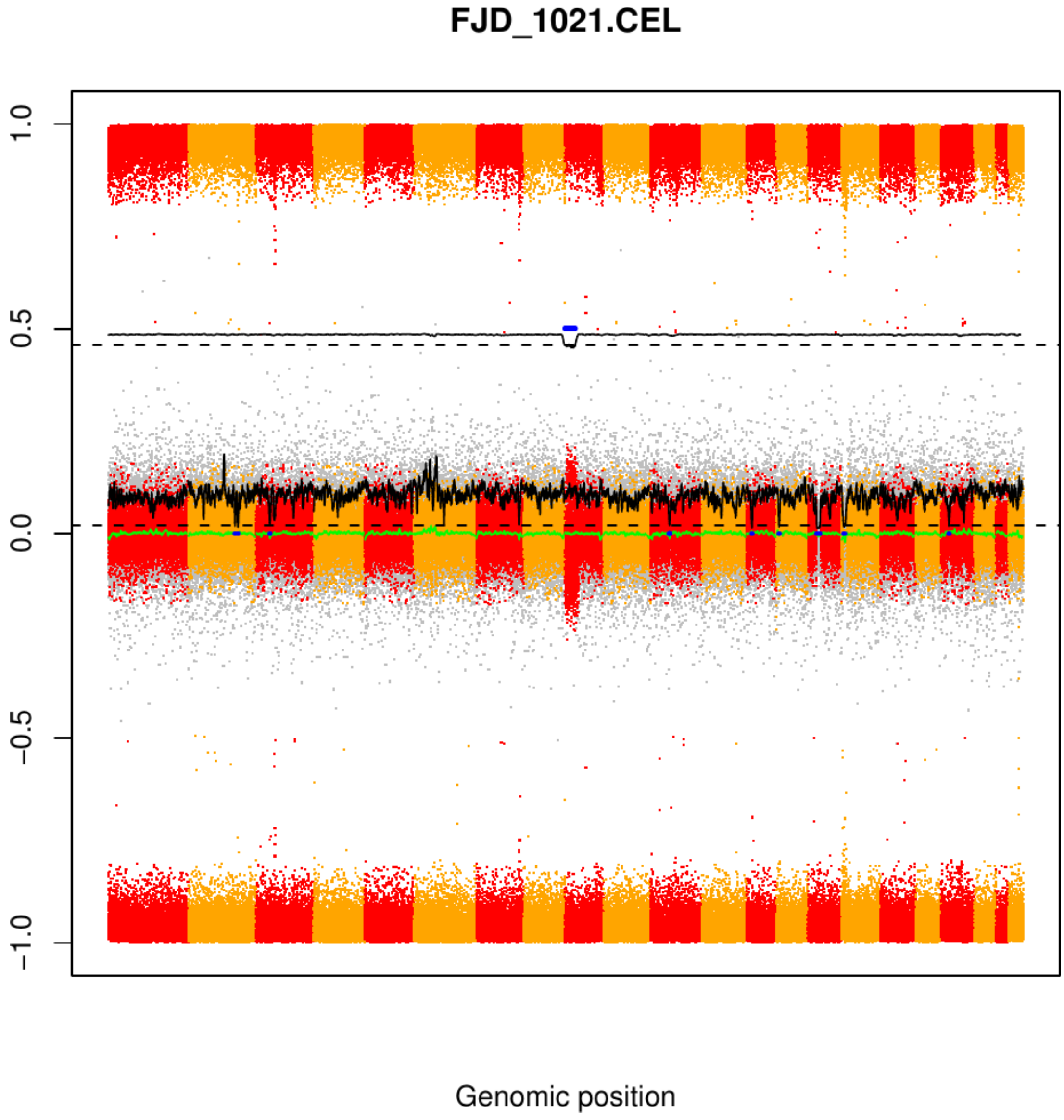
FJD_1021.CEL
1.0
0.5
0.0
−0.5
−1.0
Genomic position

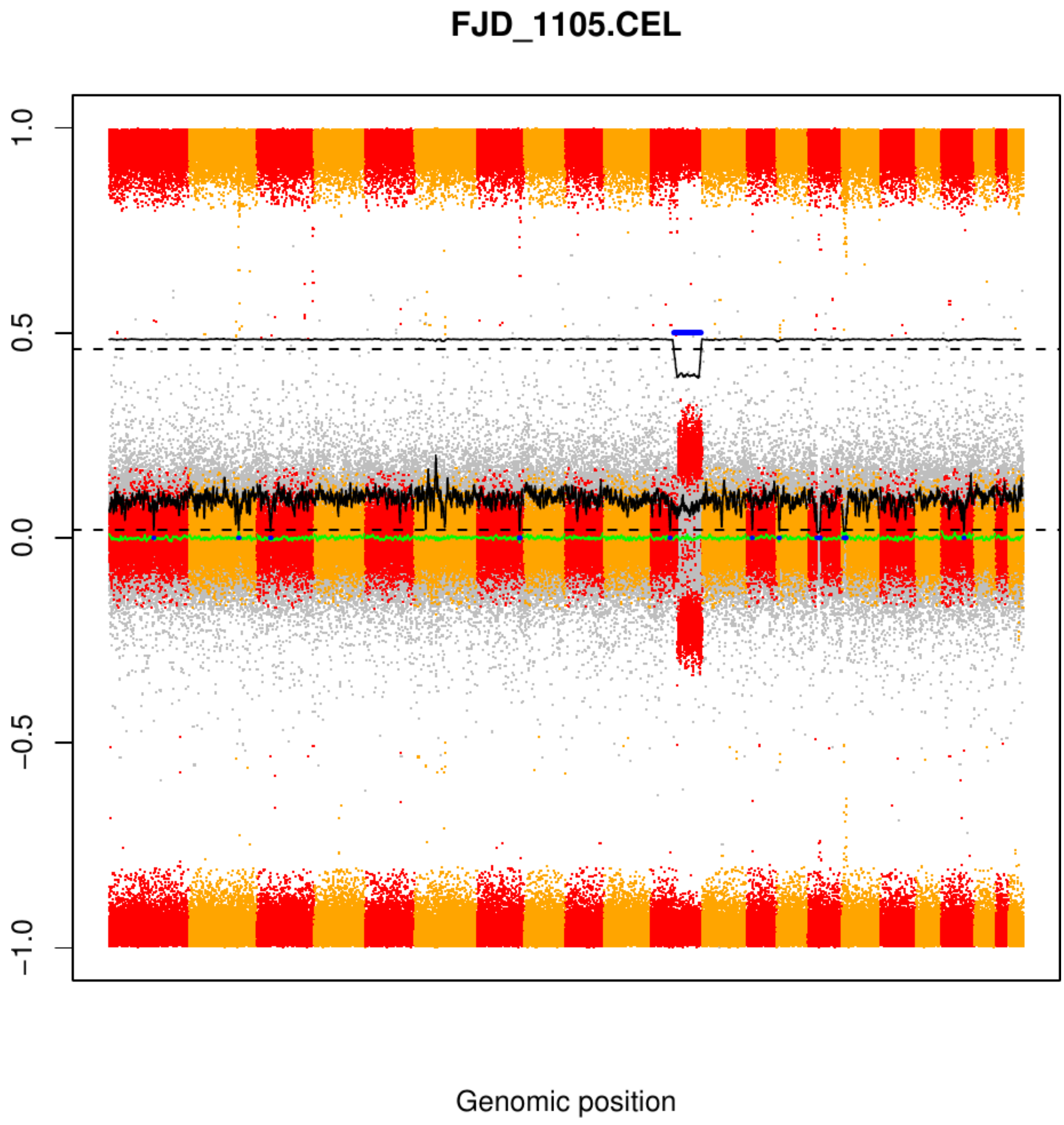
FJD_1105.CEL
1.0
0.5
0.0
−0.5
−1.0
Genomic position

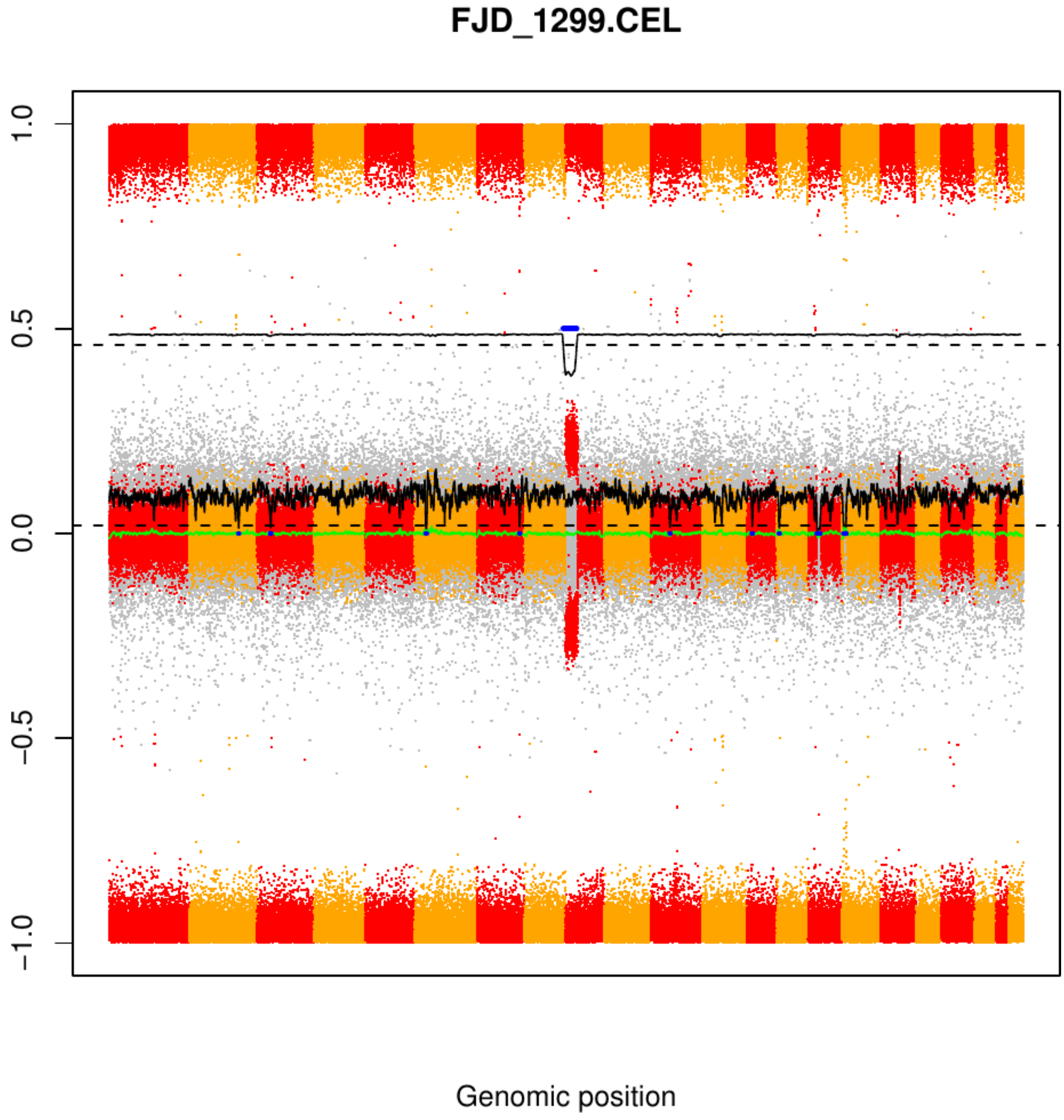
FJD_1299.CEL
1.0
0.5
0.0
−0.5
−1.0
Genomic position

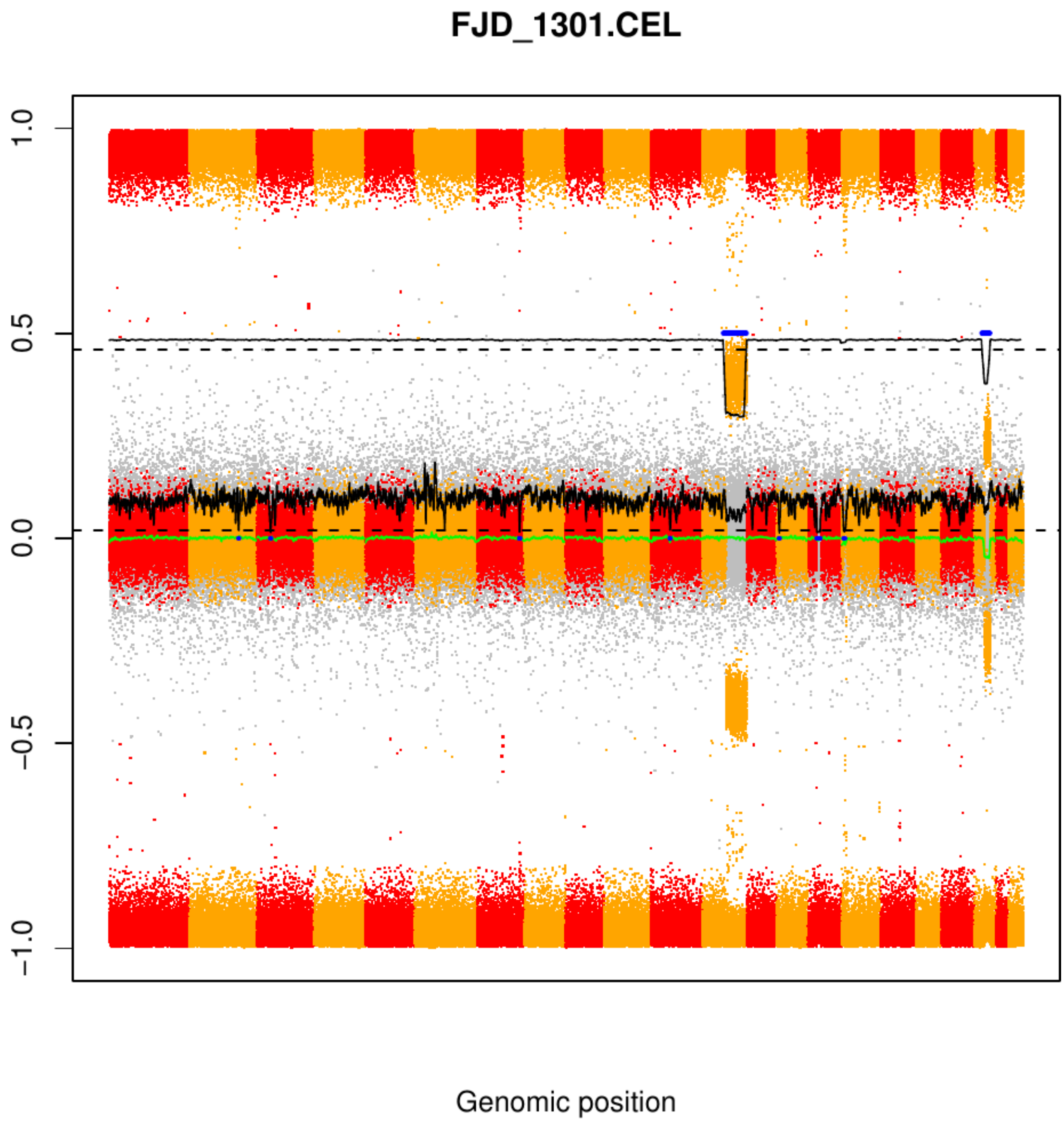
FJD_1301.CEL
1.0
0.5
0.0
−0.5
−1.0
Genomic position

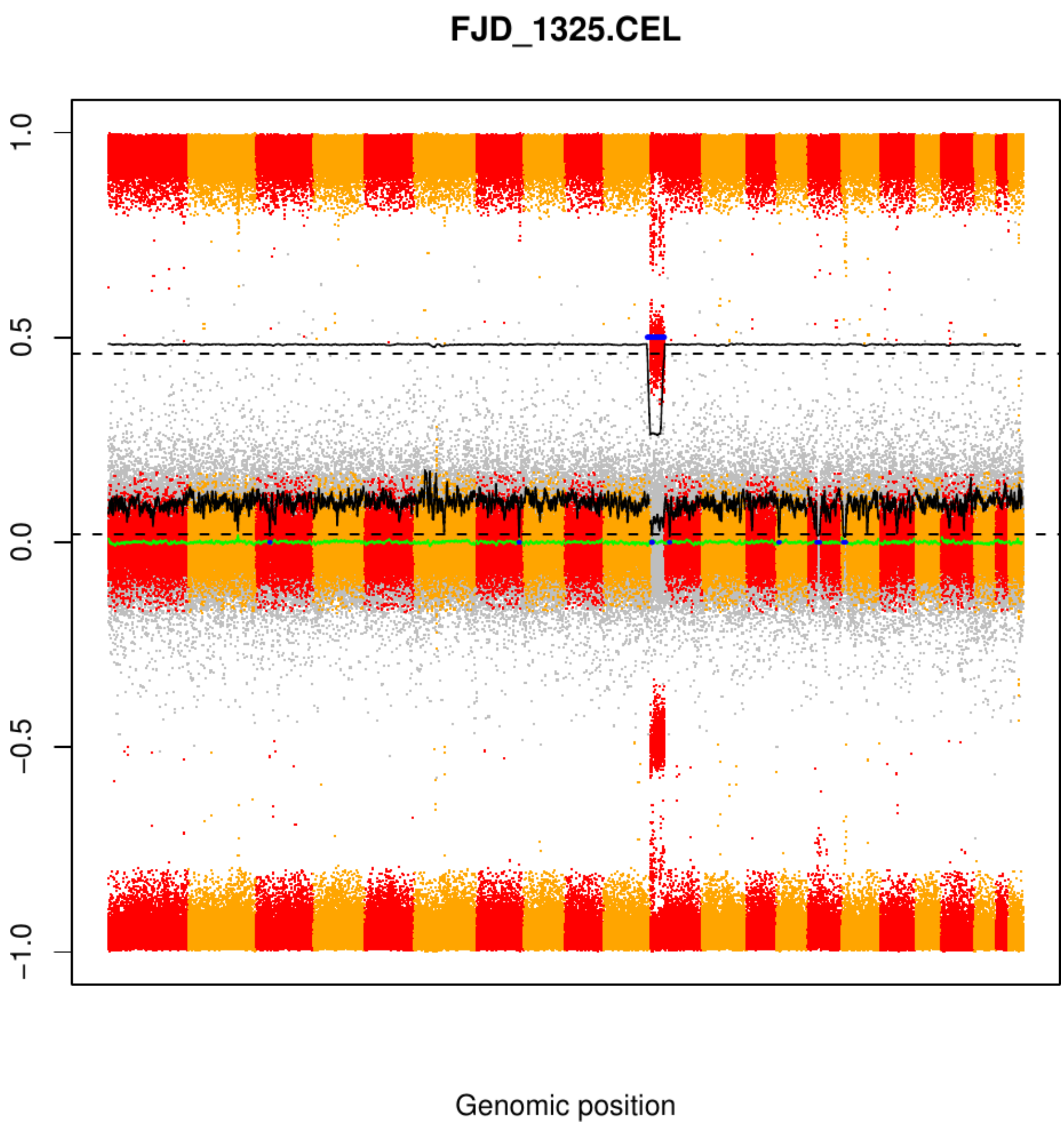
FJD_1325.CEL
1.0
0.5
0.0
−0.5
−1.0
Genomic position

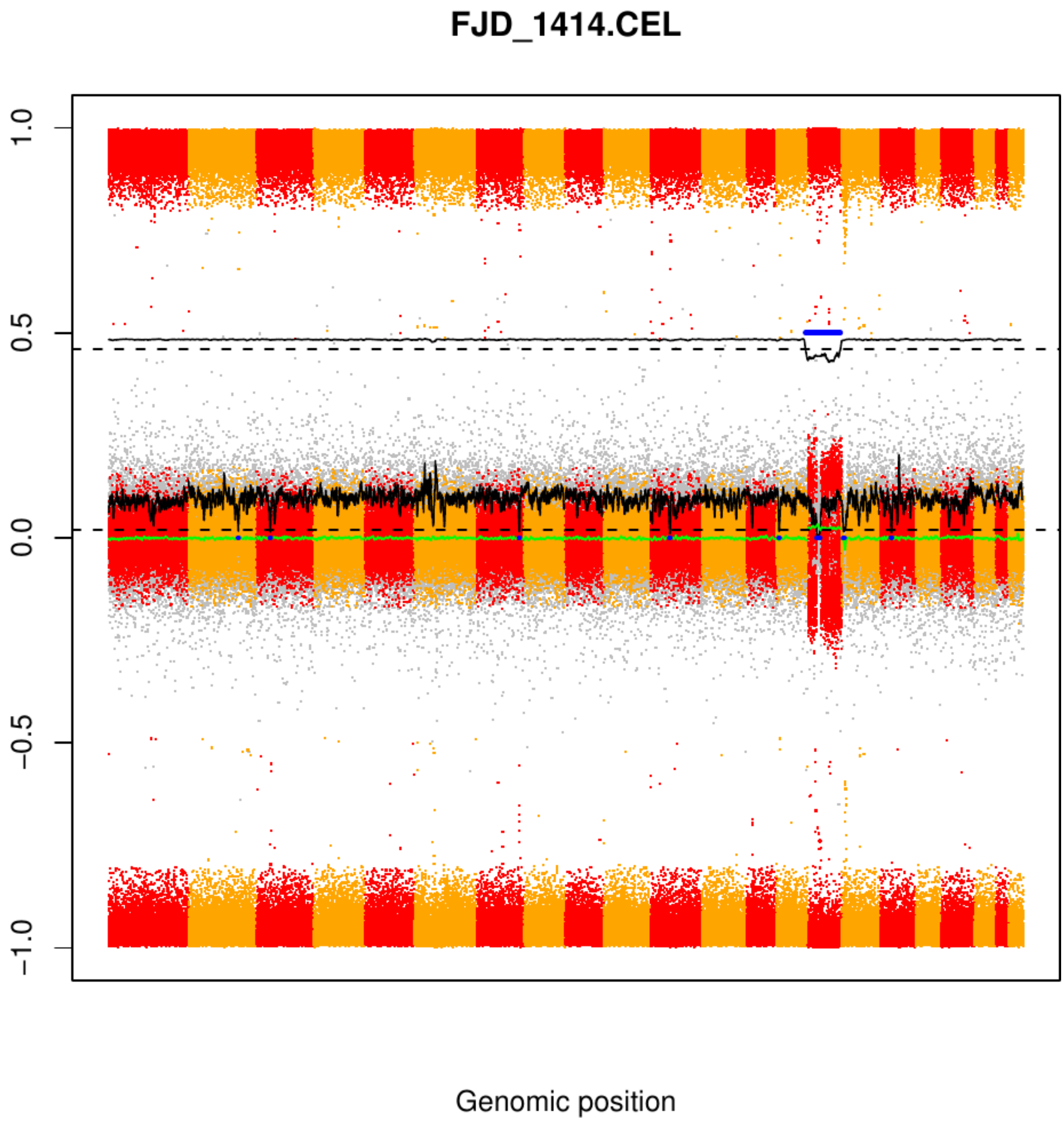
FJD_1414.CEL
1.0
0.5
0.0
−0.5
−1.0
Genomic position

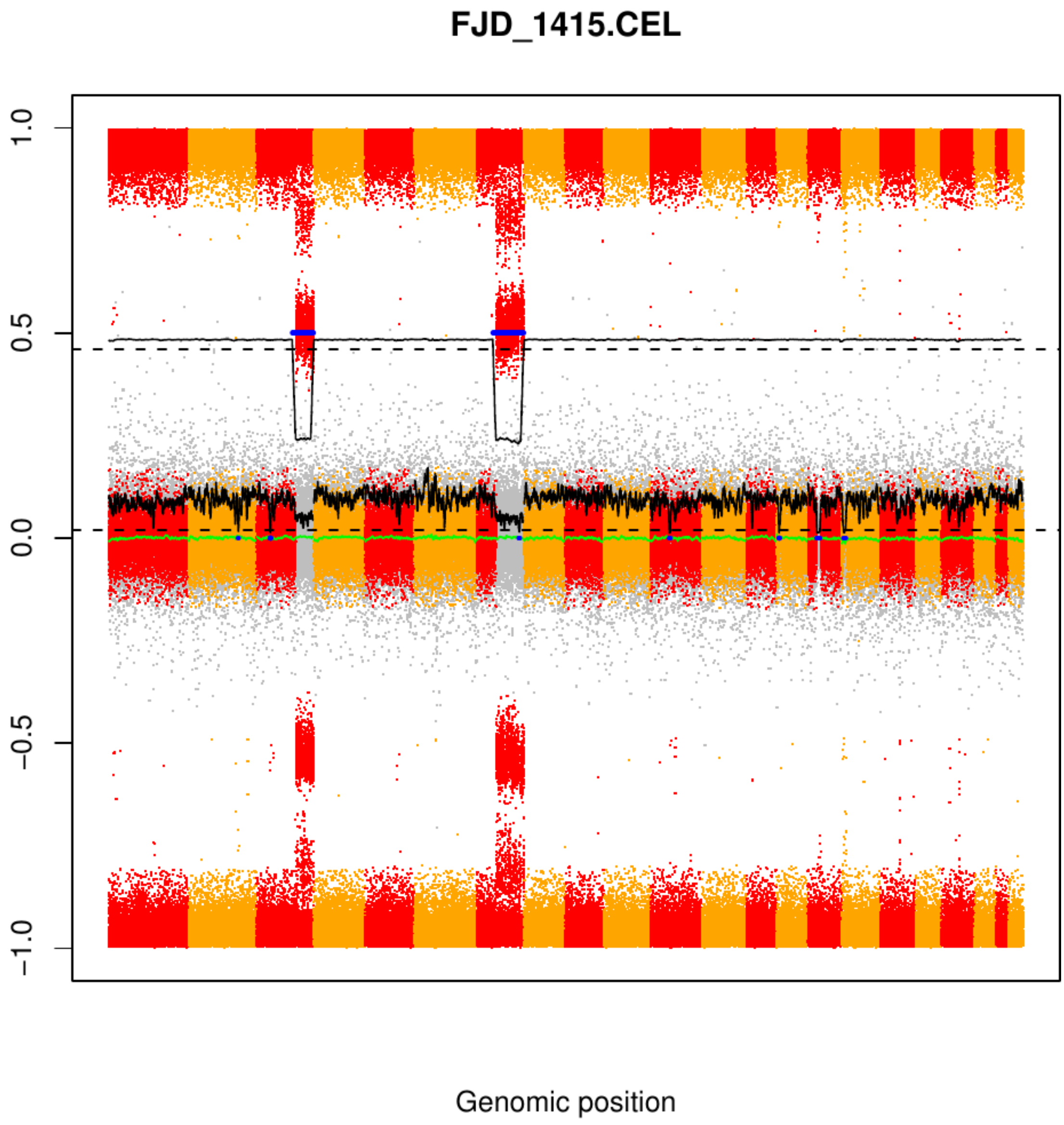

FJD_1415.CEL
1.0
0.5
0.0
−0.5
−1.0
Genomic position

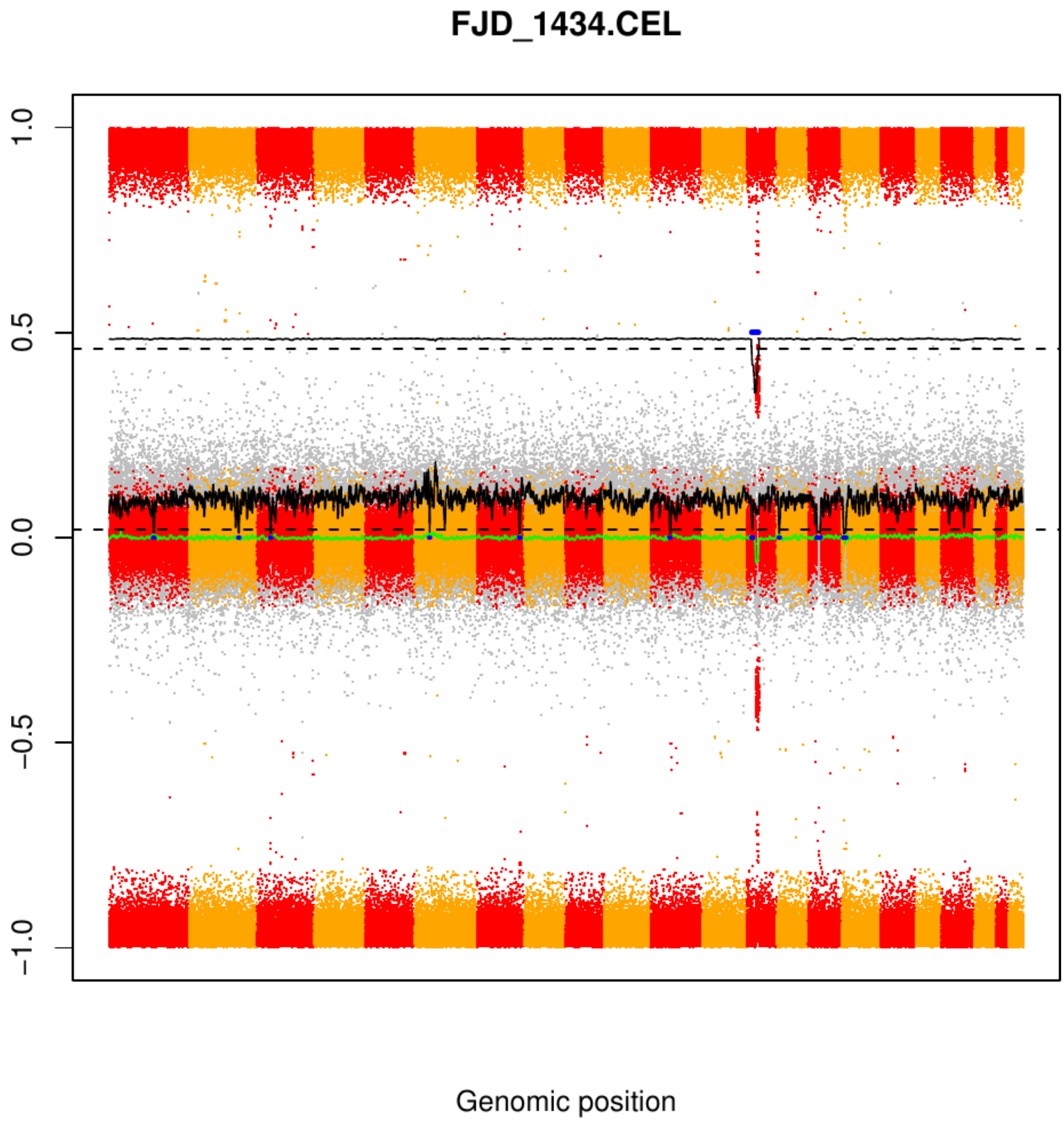
FJD_1434.CEL
1.0
0.5
0.0
−0.5
−1.0
Genomic position

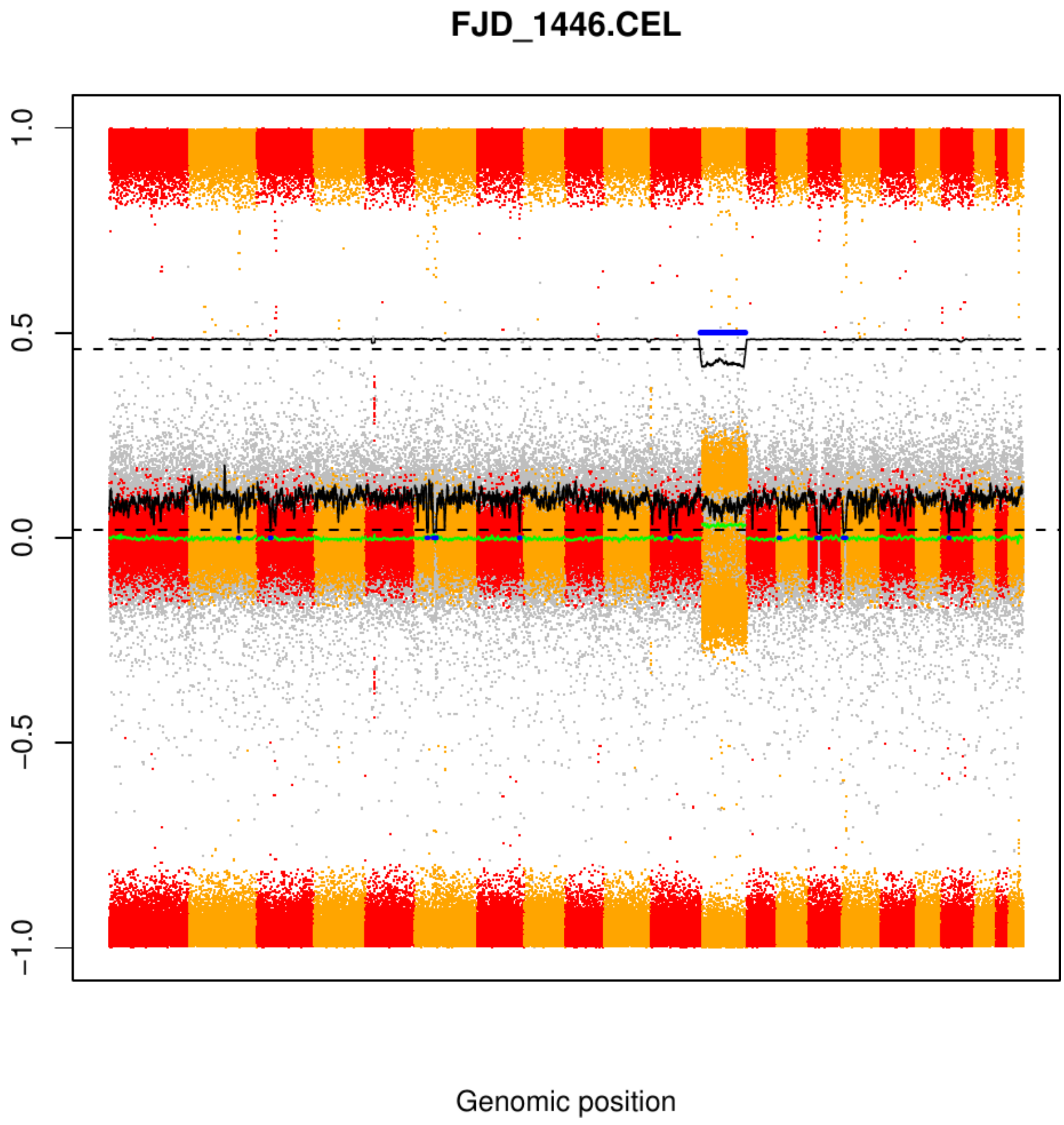
FJD_1446.CEL
1.0
0.5
0.0
−0.5
−1.0
Genomic position

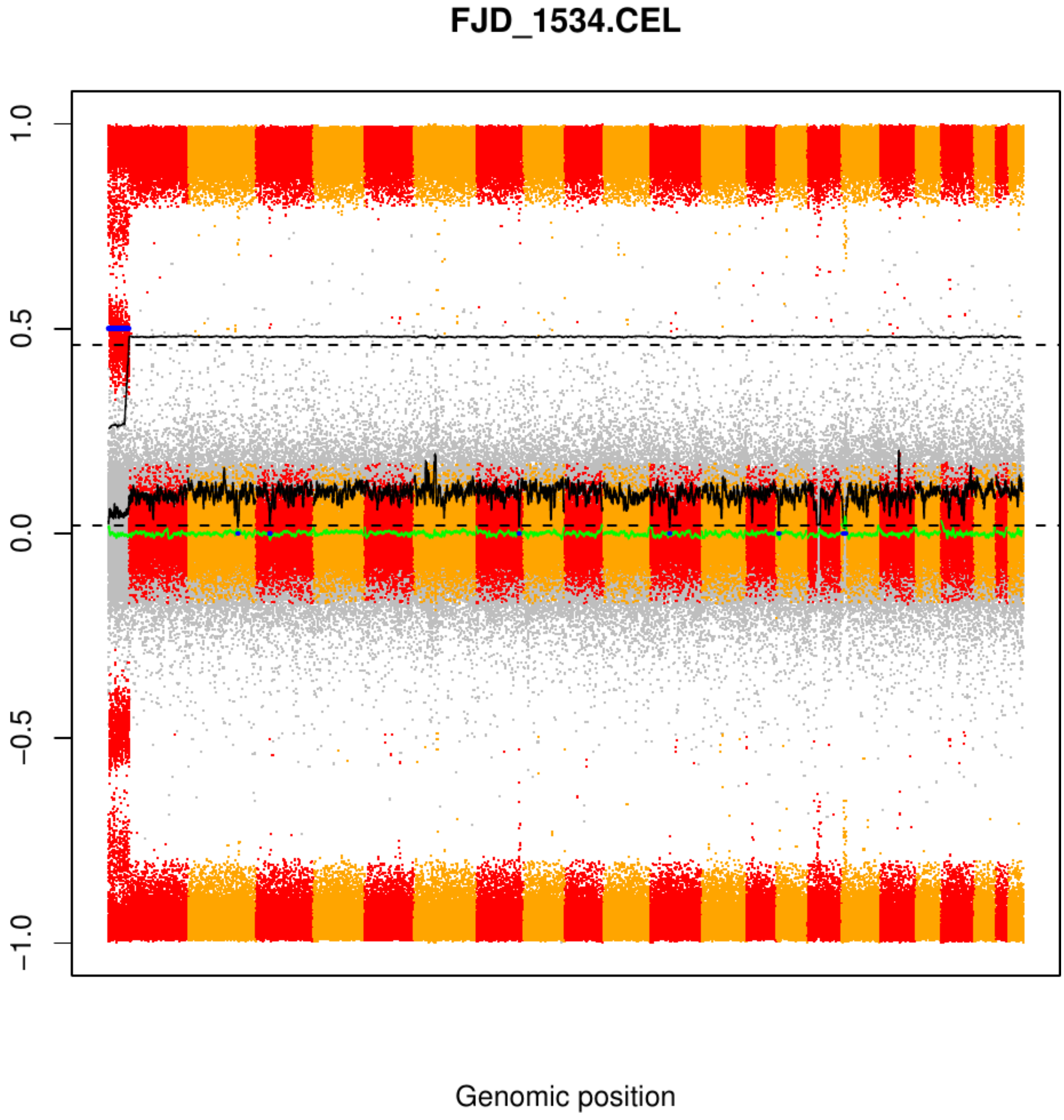
FJD_1534.CEL
1.0
0.5
0.0
−0.5
−1.0
Genomic position

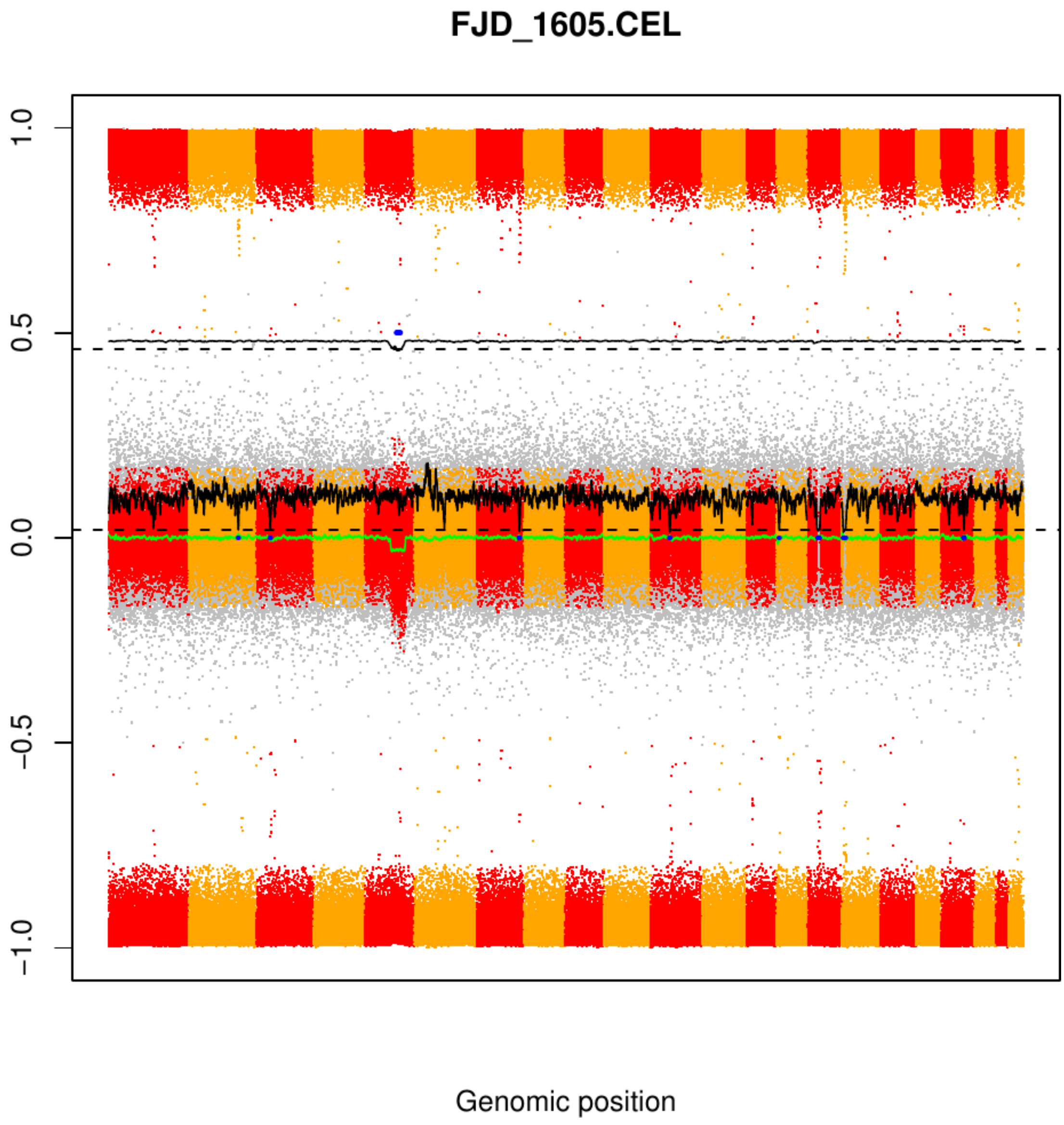
FJD_1605.CEL
1.0
0.5
0.0
−0.5
−1.0
Genomic position

FJD_1695.CEL

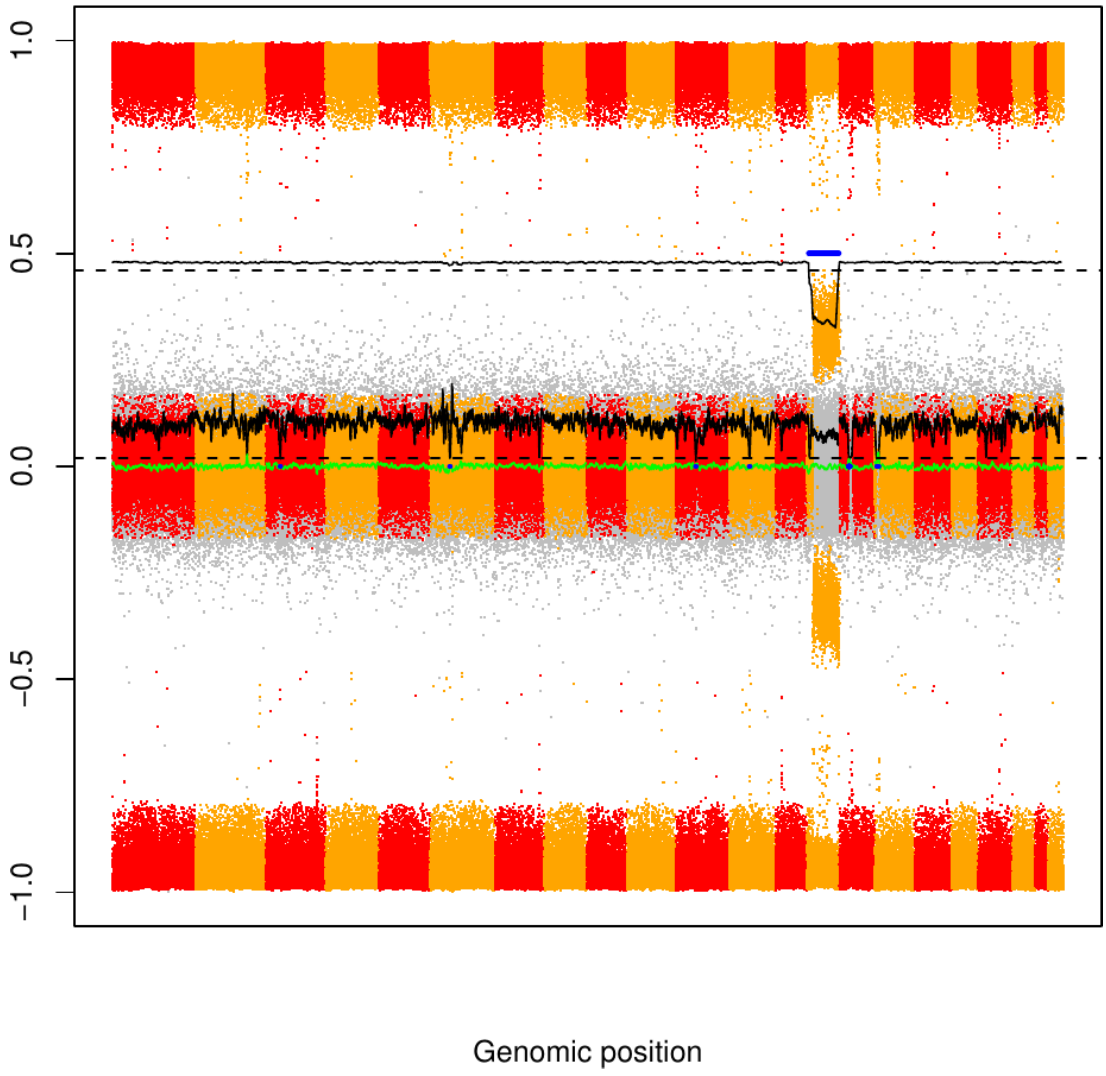

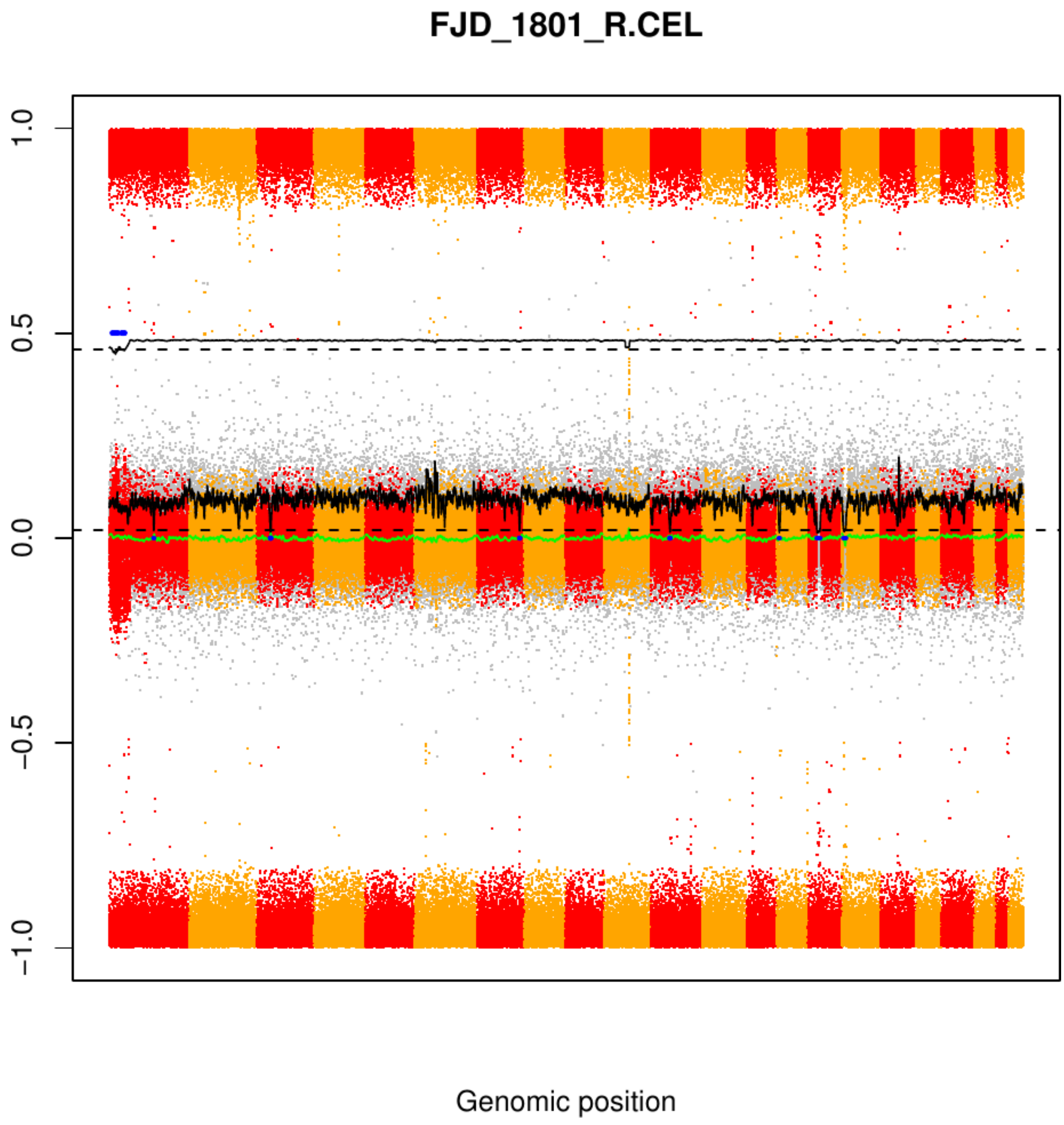
FJD_1801_R.CEL
1.0
0.5
0.0
−0.5
−1.0
Genomic position

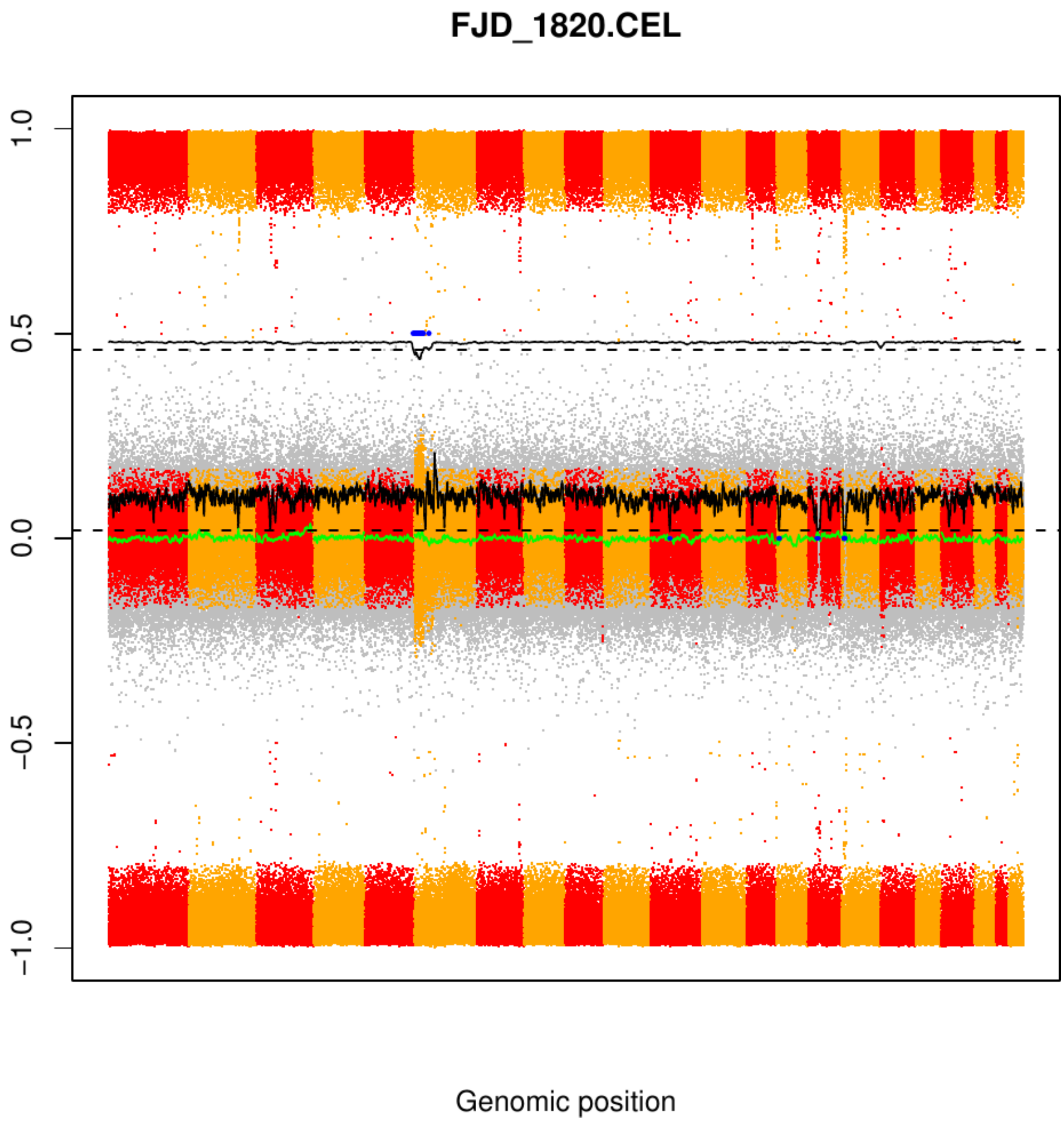
FJD_1820.CEL
1.0
0.5
0.0
−0.5
−1.0
Genomic position

FJD_1885.CEL

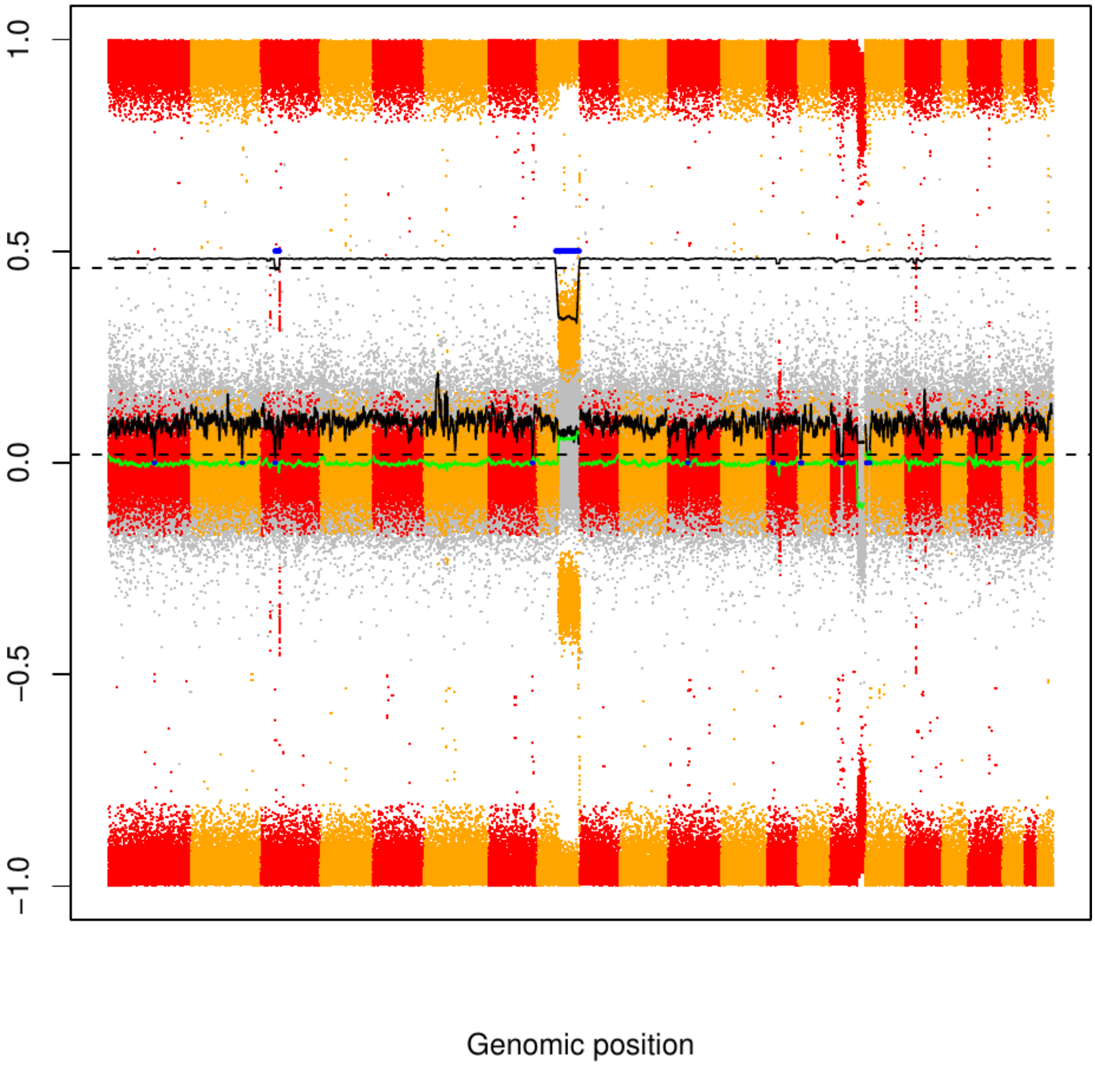

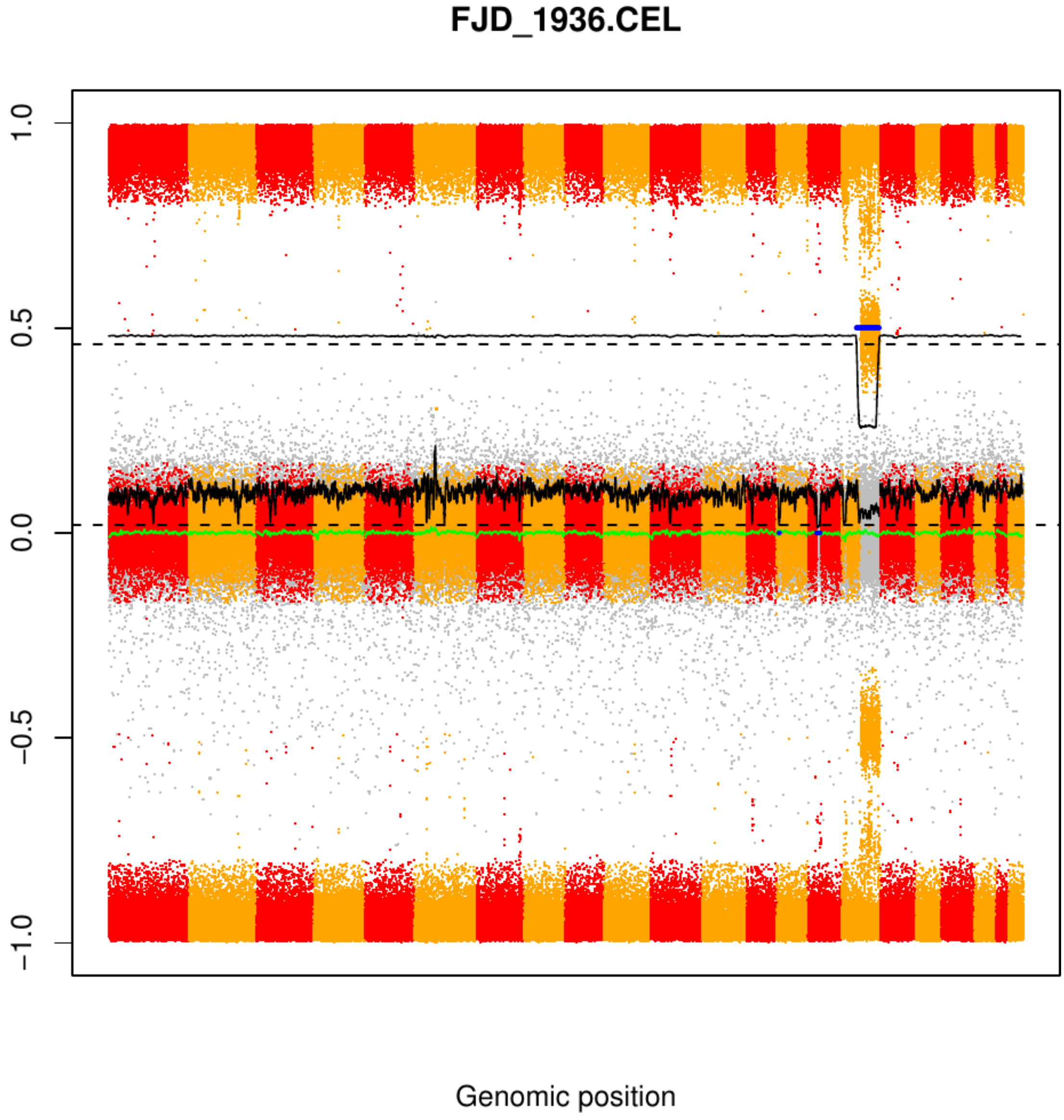
FJD_1936.CEL
1.0
0.5
0.0
−0.5
−1.0
Genomic position

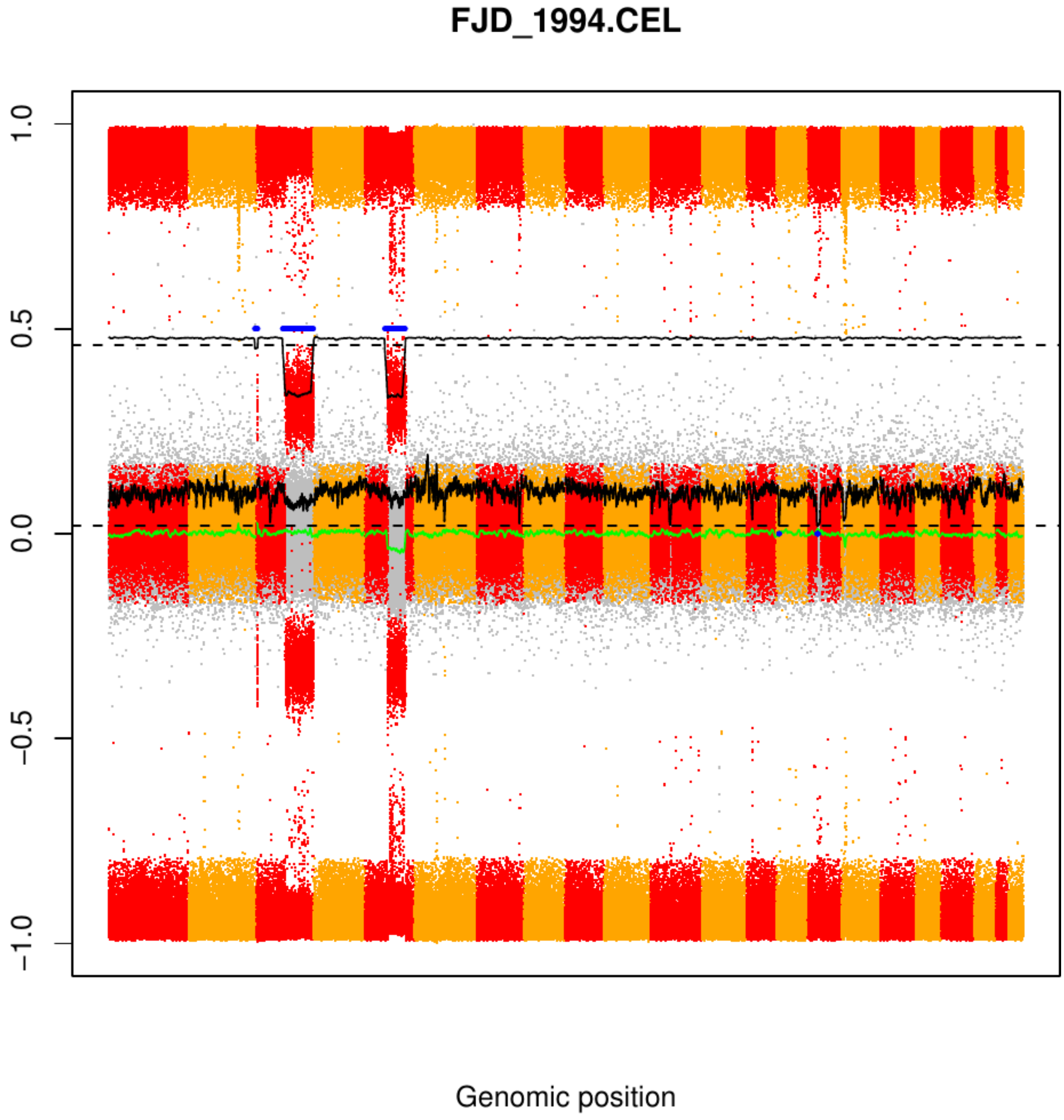
FJD_1994.CEL
1.0
0.5
0.0
−0.5
−1.0
Genomic position

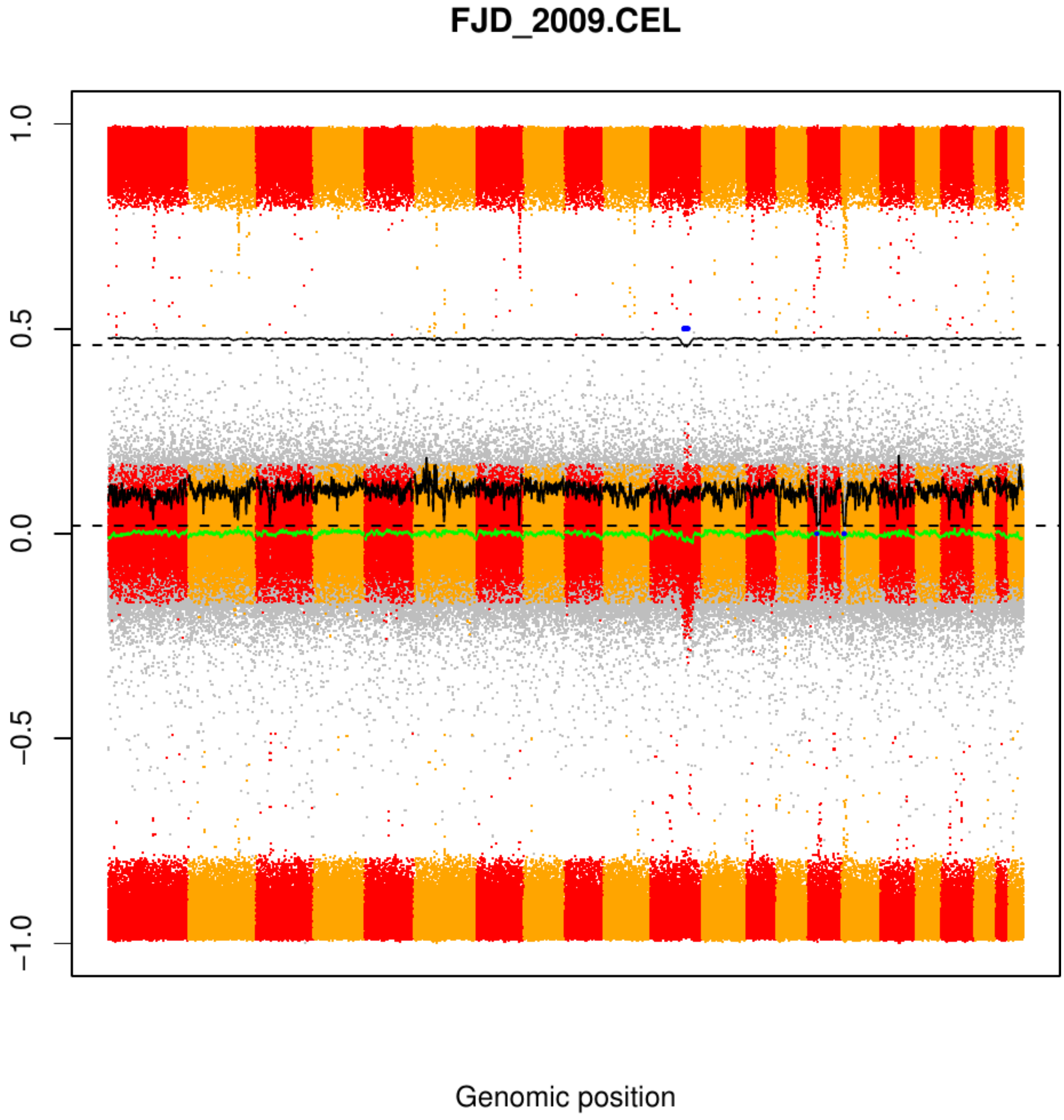
FJD_2009.CEL
1.0
0.5
0.0
−0.5
−1.0
Genomic position

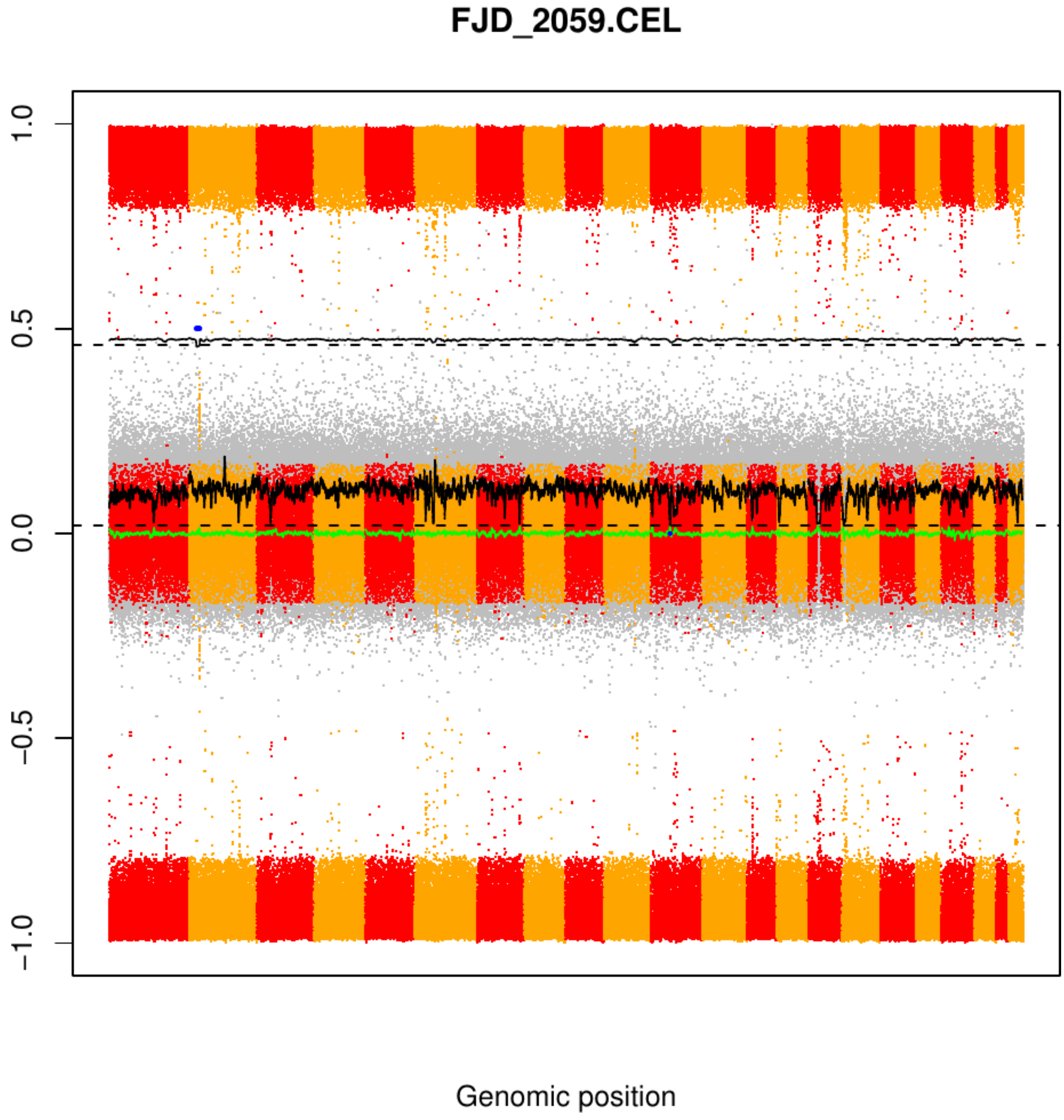
FJD_2059.CEL
1.0
0.5
0.0
−0.5
−1.0
Genomic position

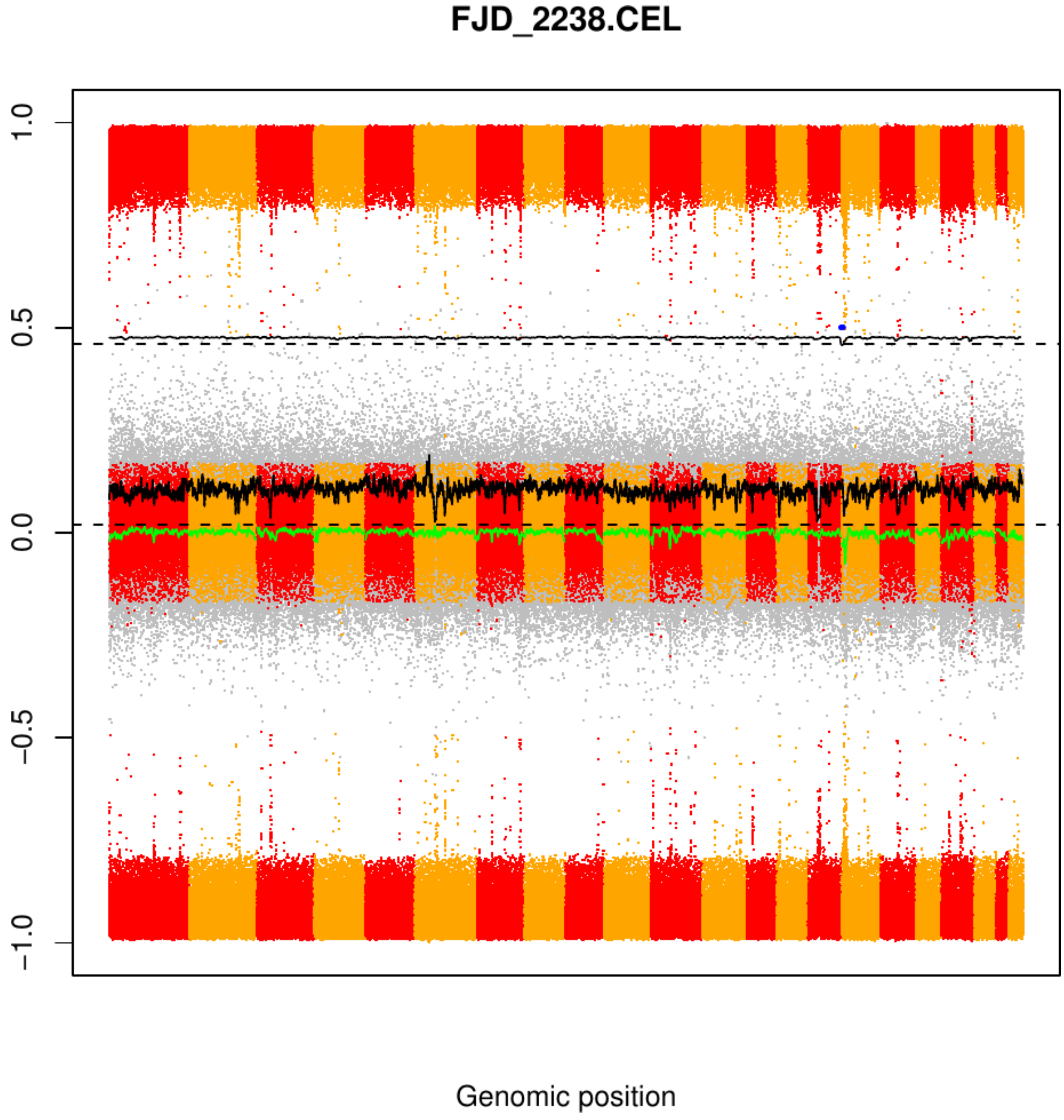

FJD_2238.CEL
1.0
0.5
0.0
−0.5
−1.0
Genomic position

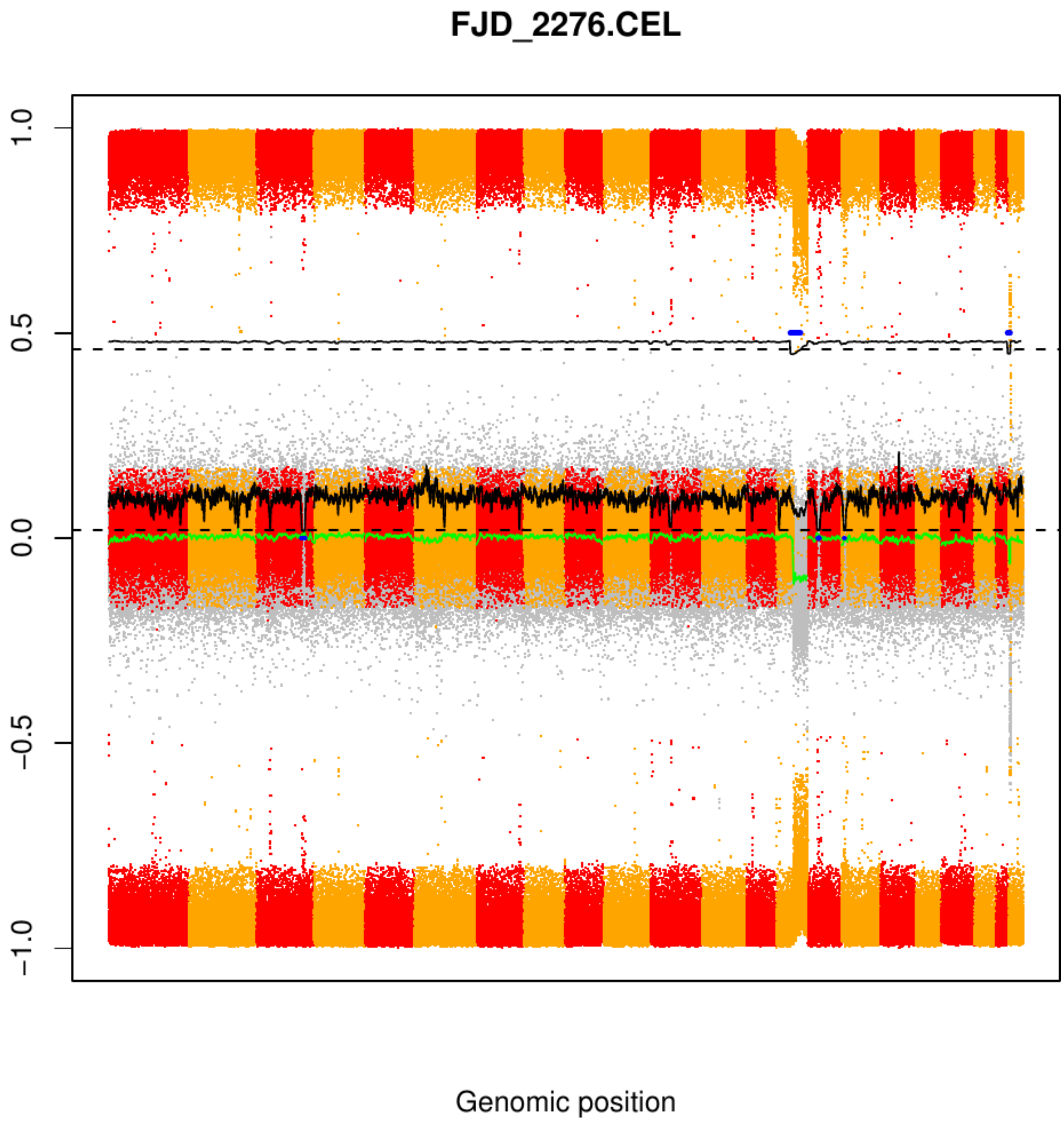

FJD_2276.CEL
1.0
0.5
0.0
−0.5
−1.0
Genomic position

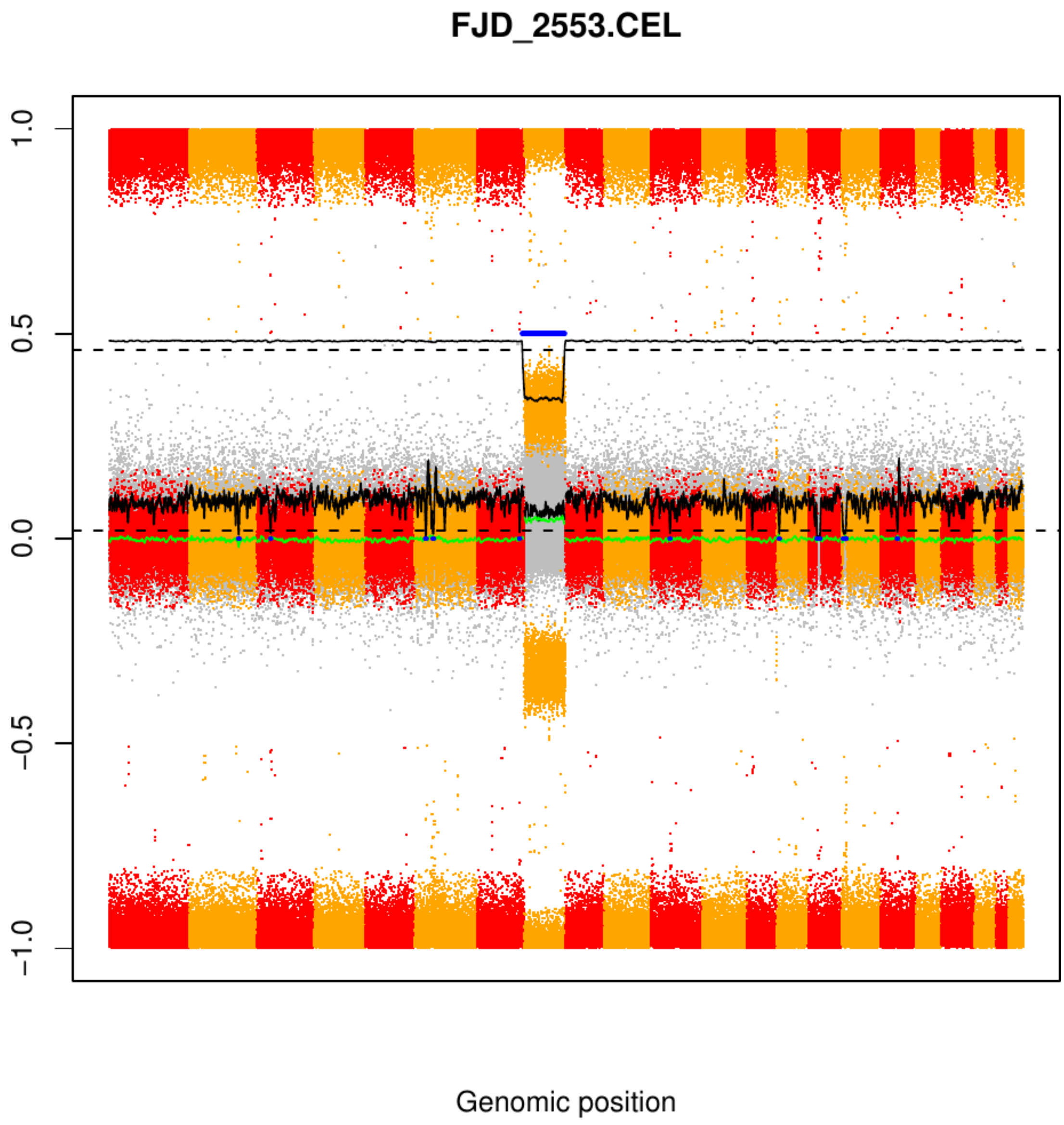

FJD_2553.CEL
1.0
0.5
0.0
−0.5
−1.0
Genomic position

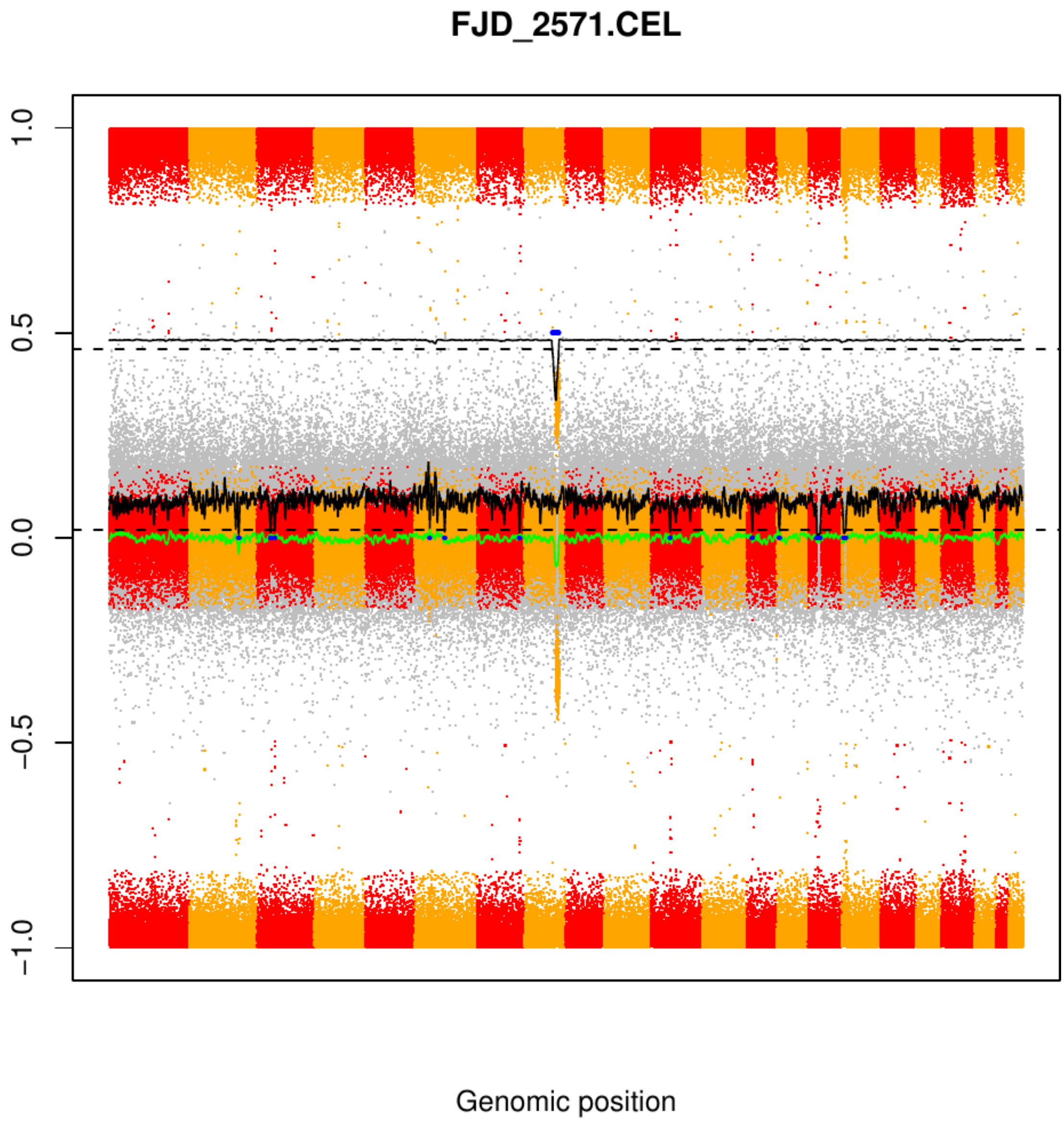
FJD_2571.CEL
1.0
0.5
0.0
−0.5
−1.0
Genomic position

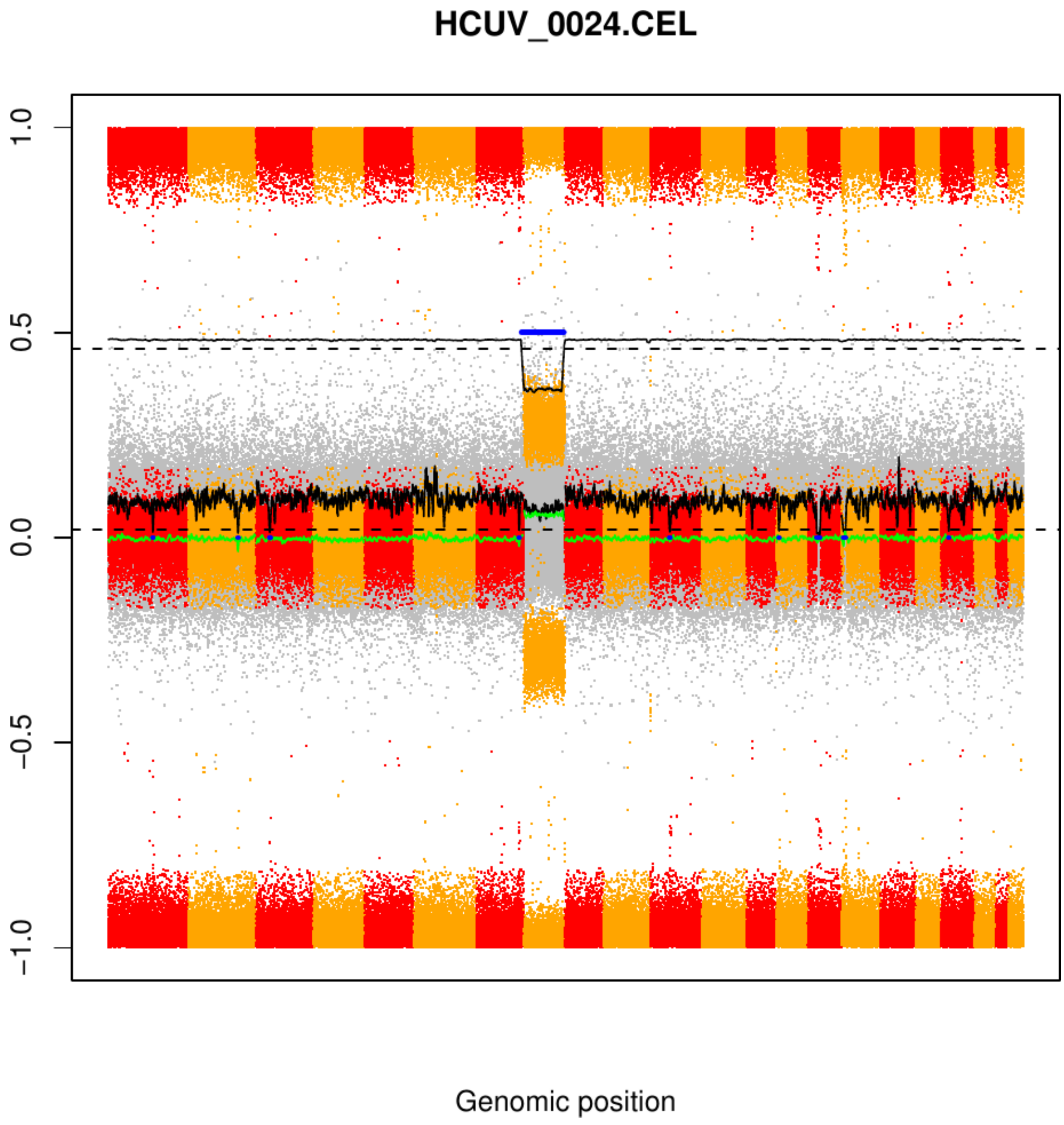

HCUV_0024.CEL
1.0
0.5
0.0
−0.5
−1.0
Genomic position

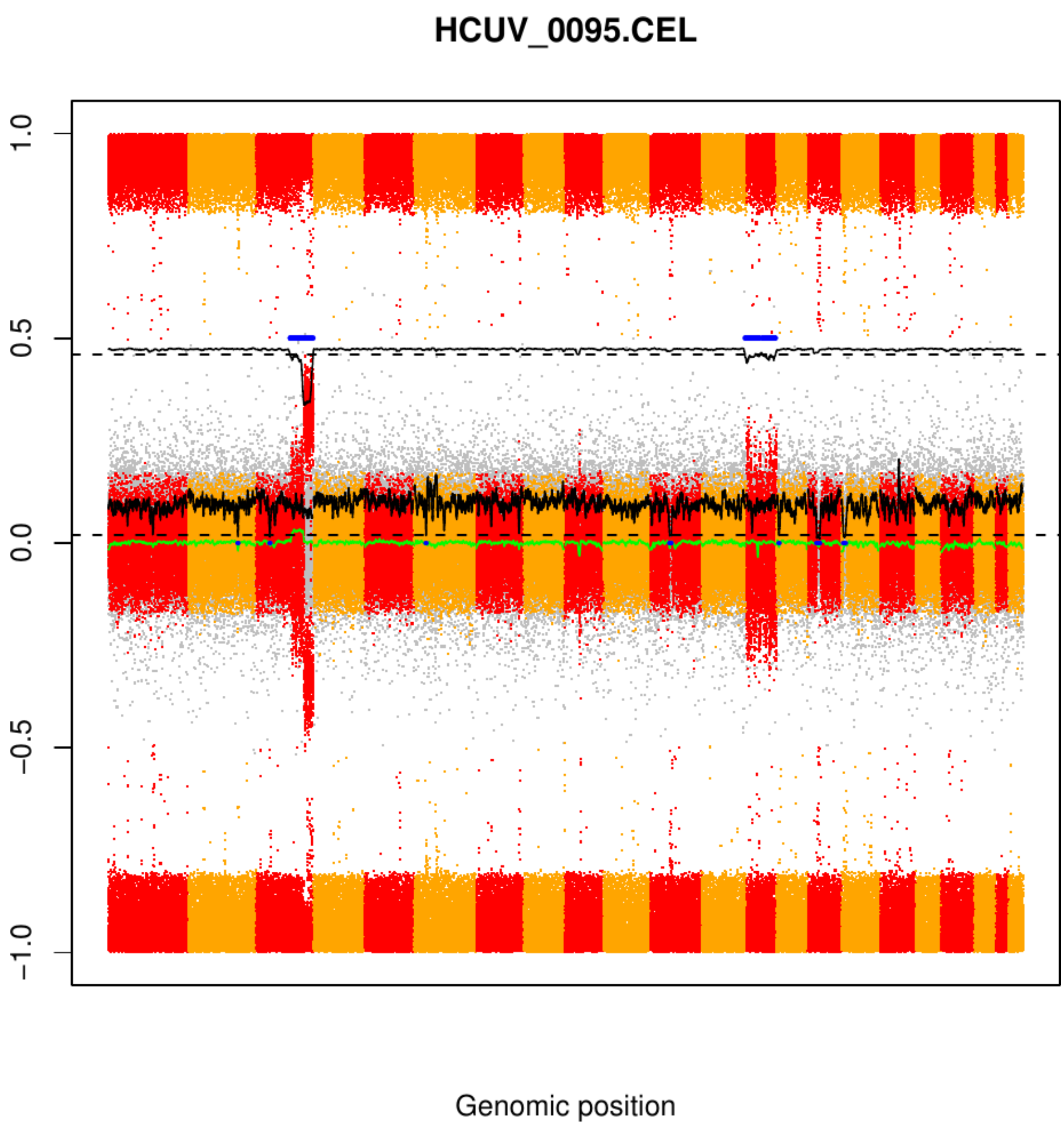
HCUV_0095.CEL
1.0
0.5
0.0
−0.5
−1.0
Genomic position

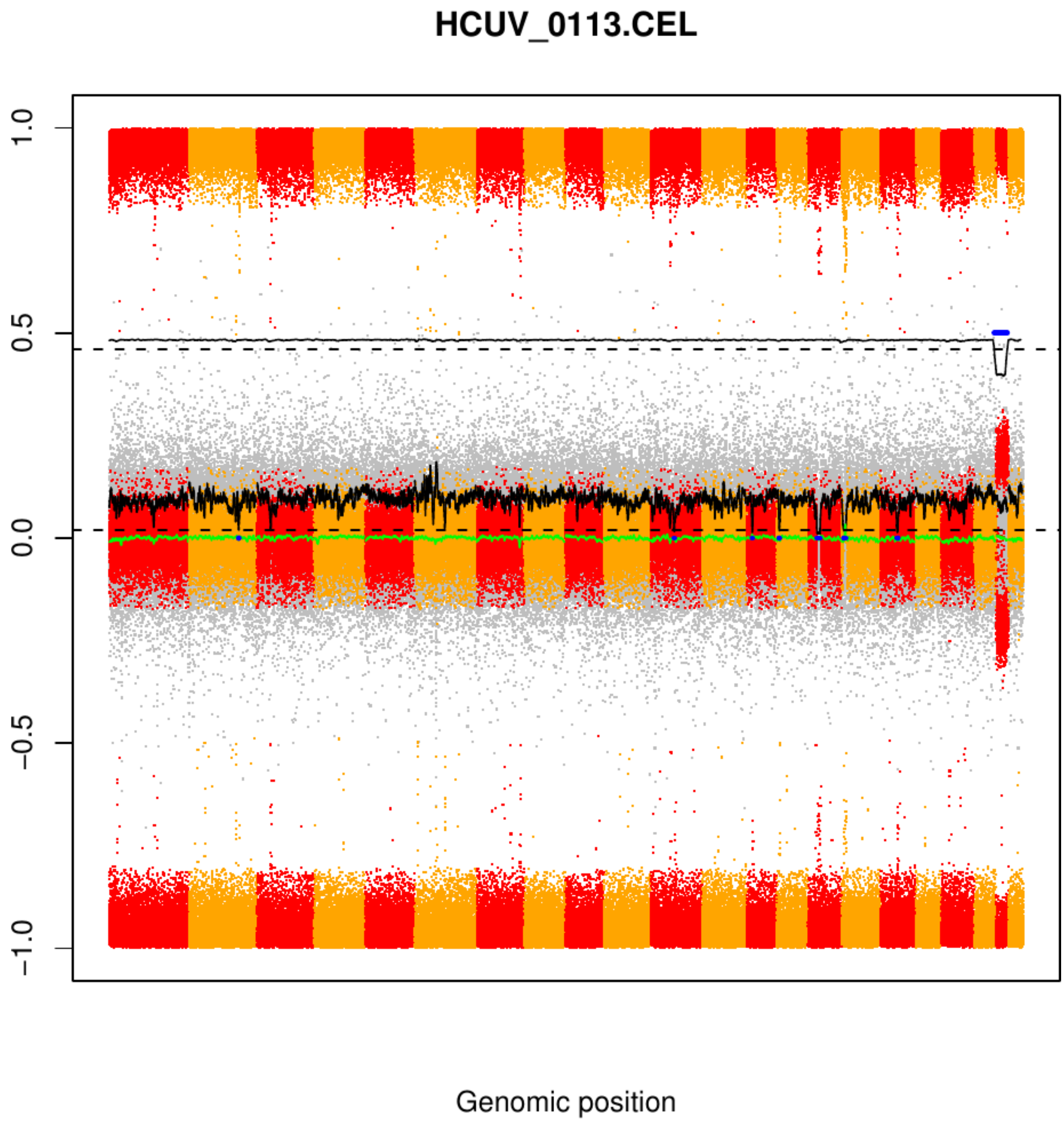
HCUV_0113.CEL
1.0
0.5
0.0
−0.5
−1.0
Genomic position

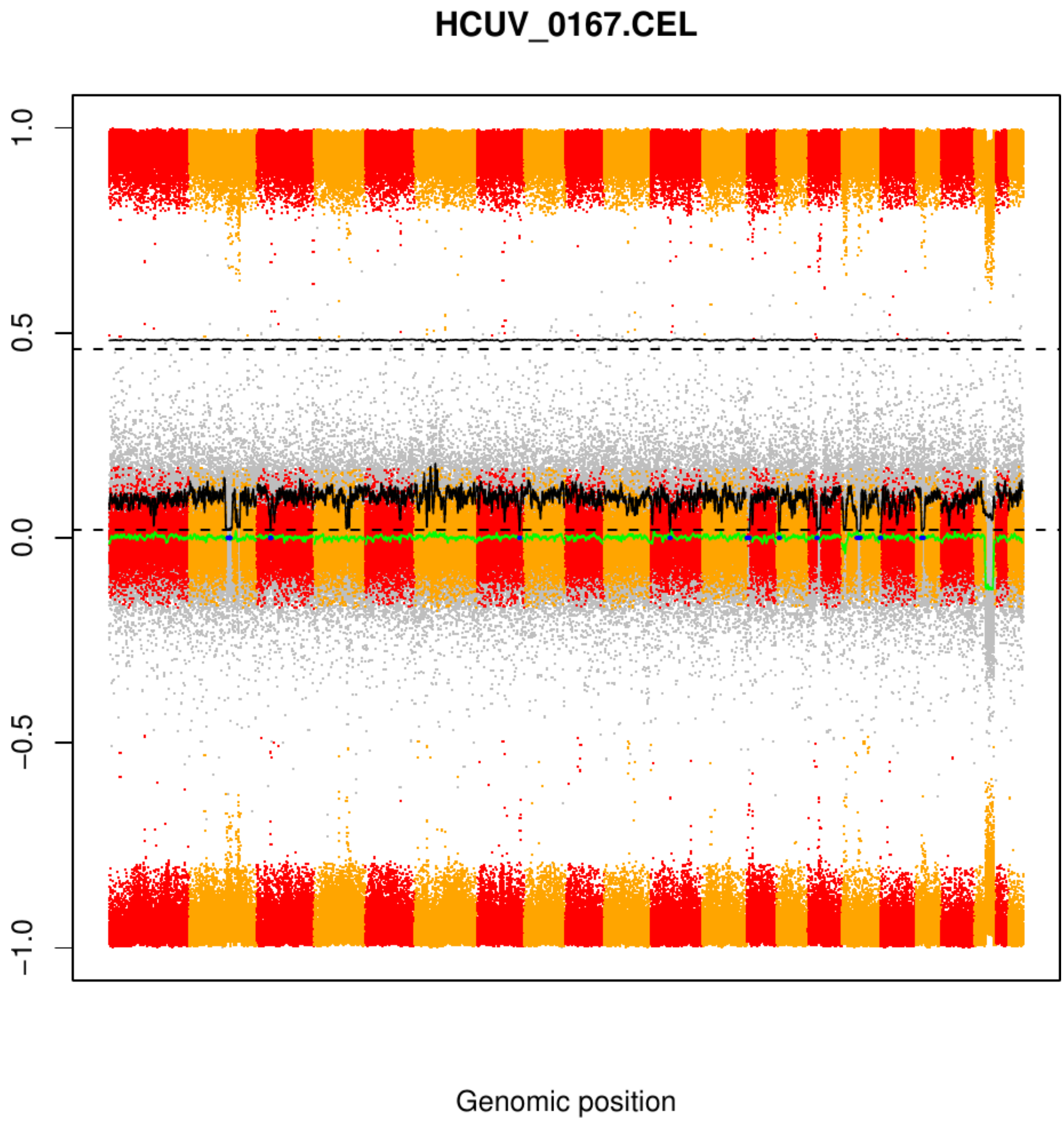
HCUV_0167.CEL
1.0
0.5
0.0
−0.5
−1.0
Genomic position

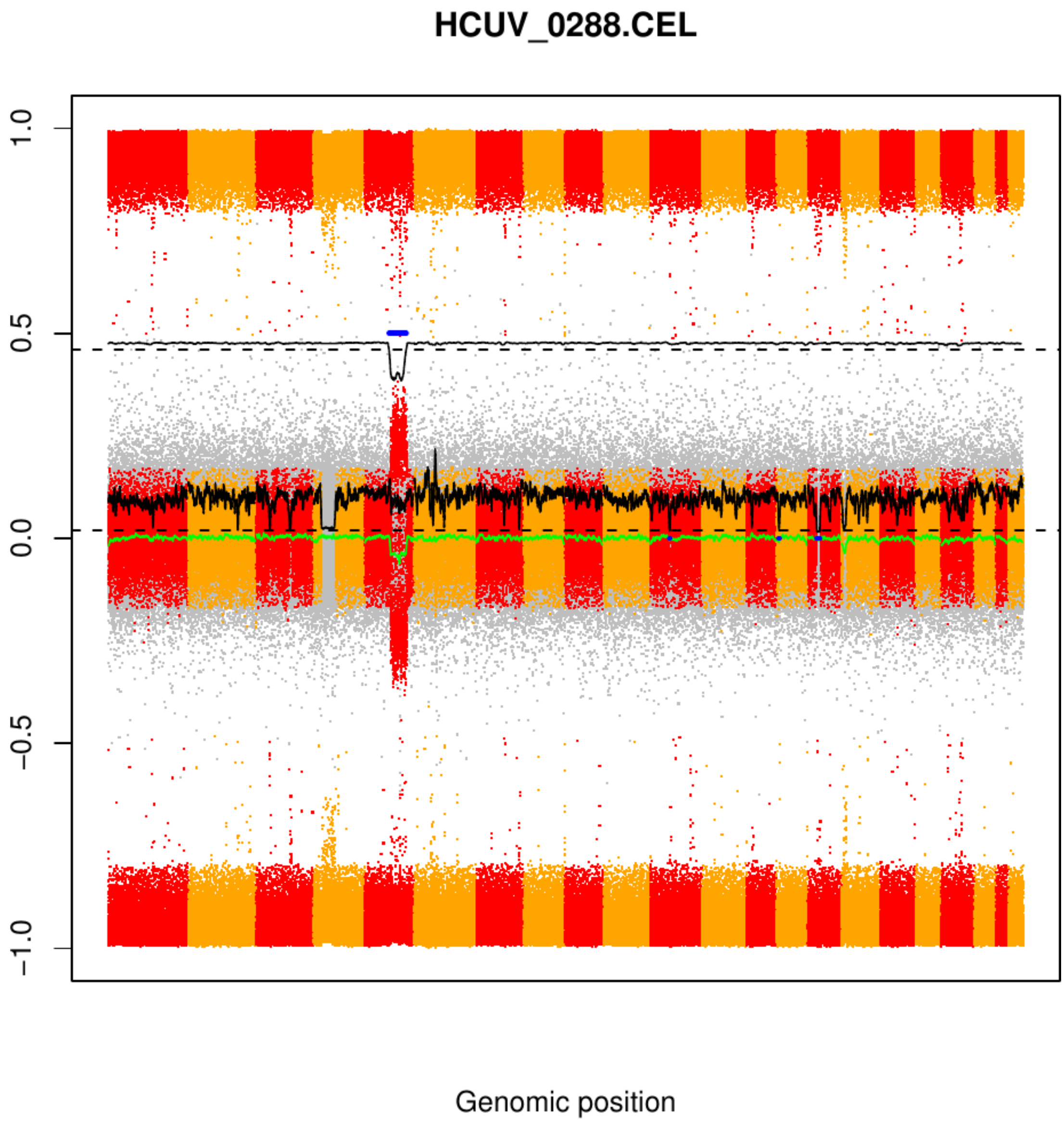
HCUV_0288.CEL
1.0
0.5
0.0
−0.5
−1.0
Genomic position

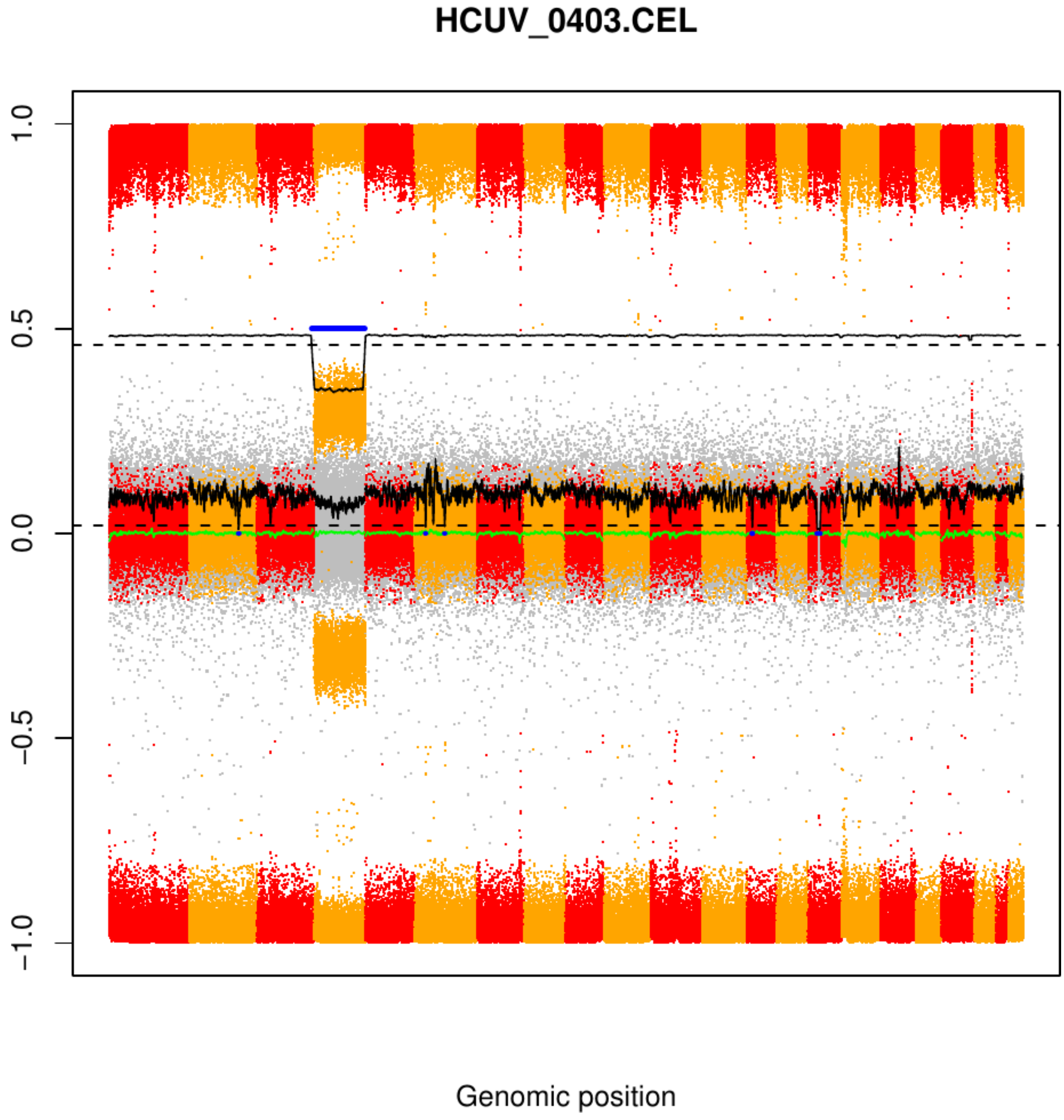
HCUV_0403.CEL
1.0
0.5
0.0
−0.5
−1.0
Genomic position

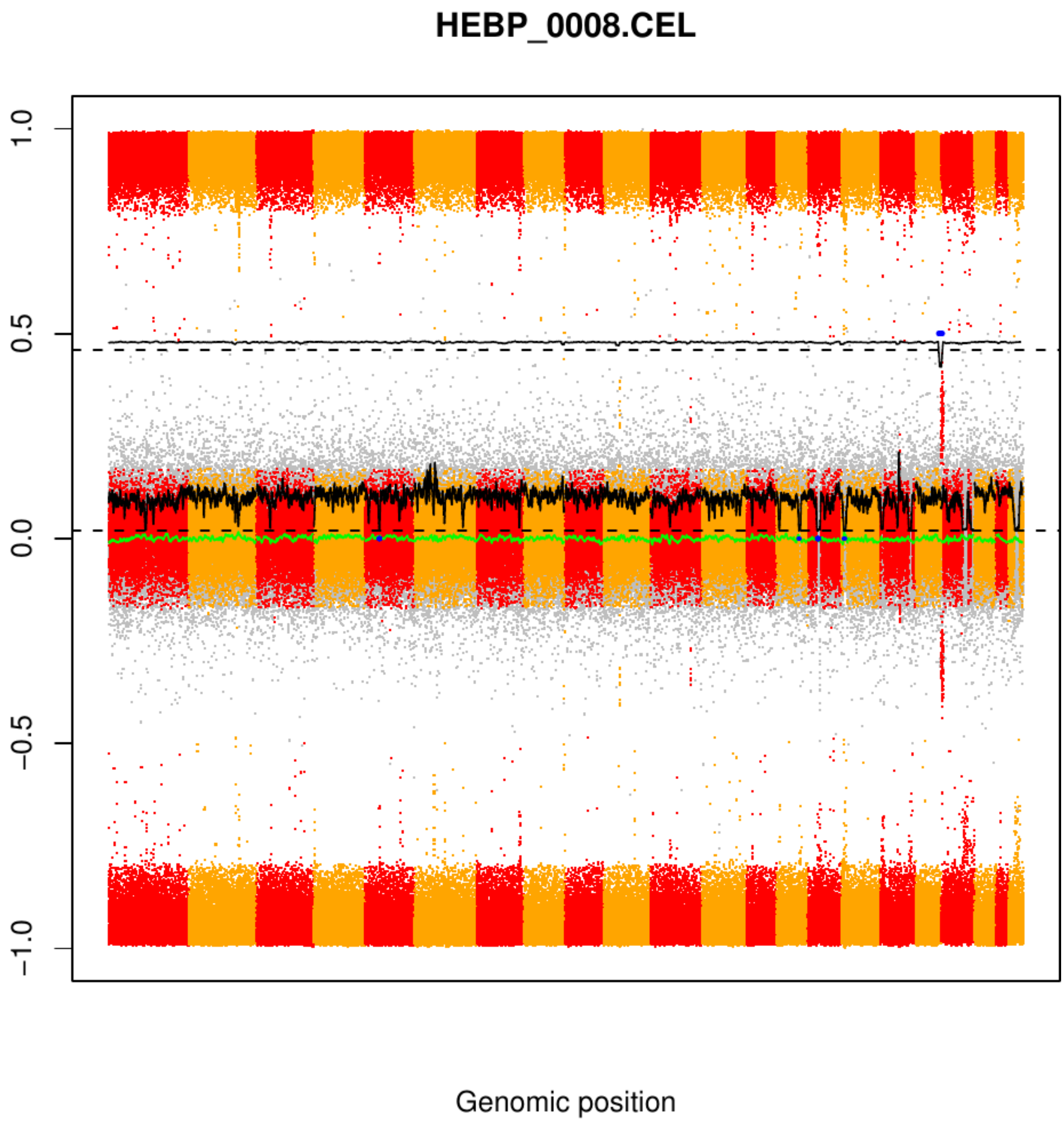
HEBP_0008.CEL
1.0
0.5
0.0
−0.5
−1.0
Genomic position

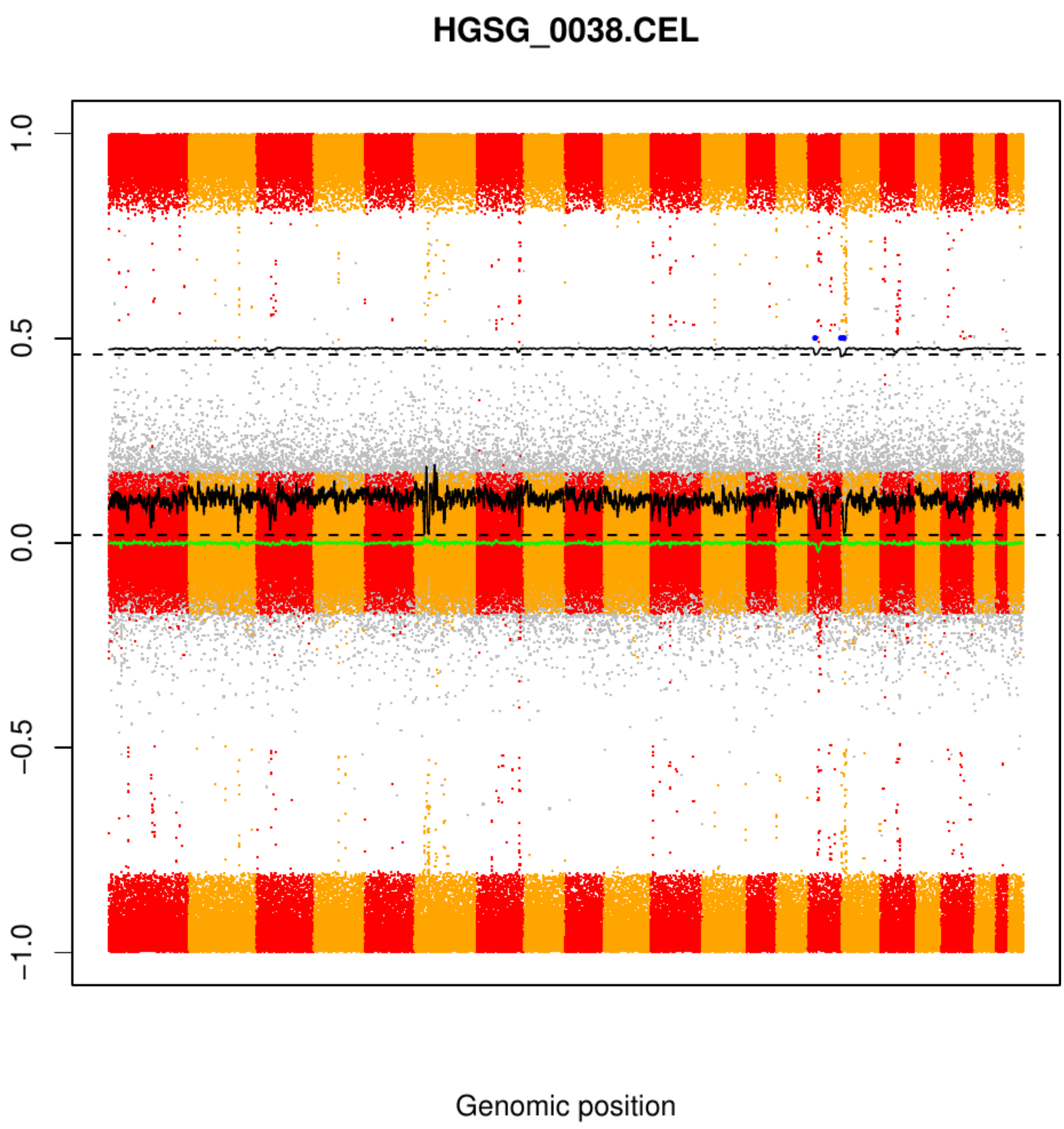
HGSG_0038.CEL
1.0
0.5
0.0
−0.5
−1.0
Genomic position

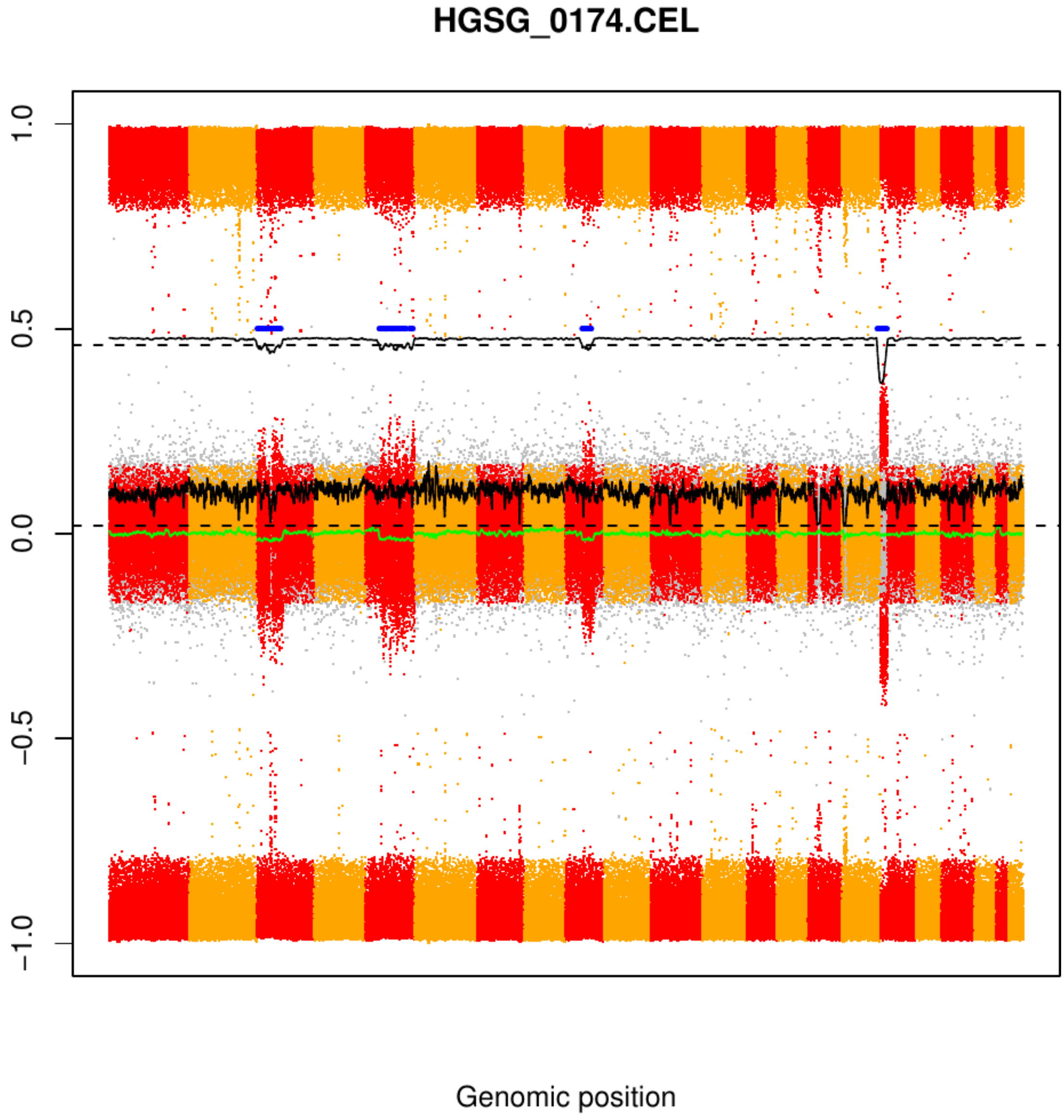
HGSG_0174.CEL
1.0
0.5
0.0
−0.5
−1.0
Genomic position

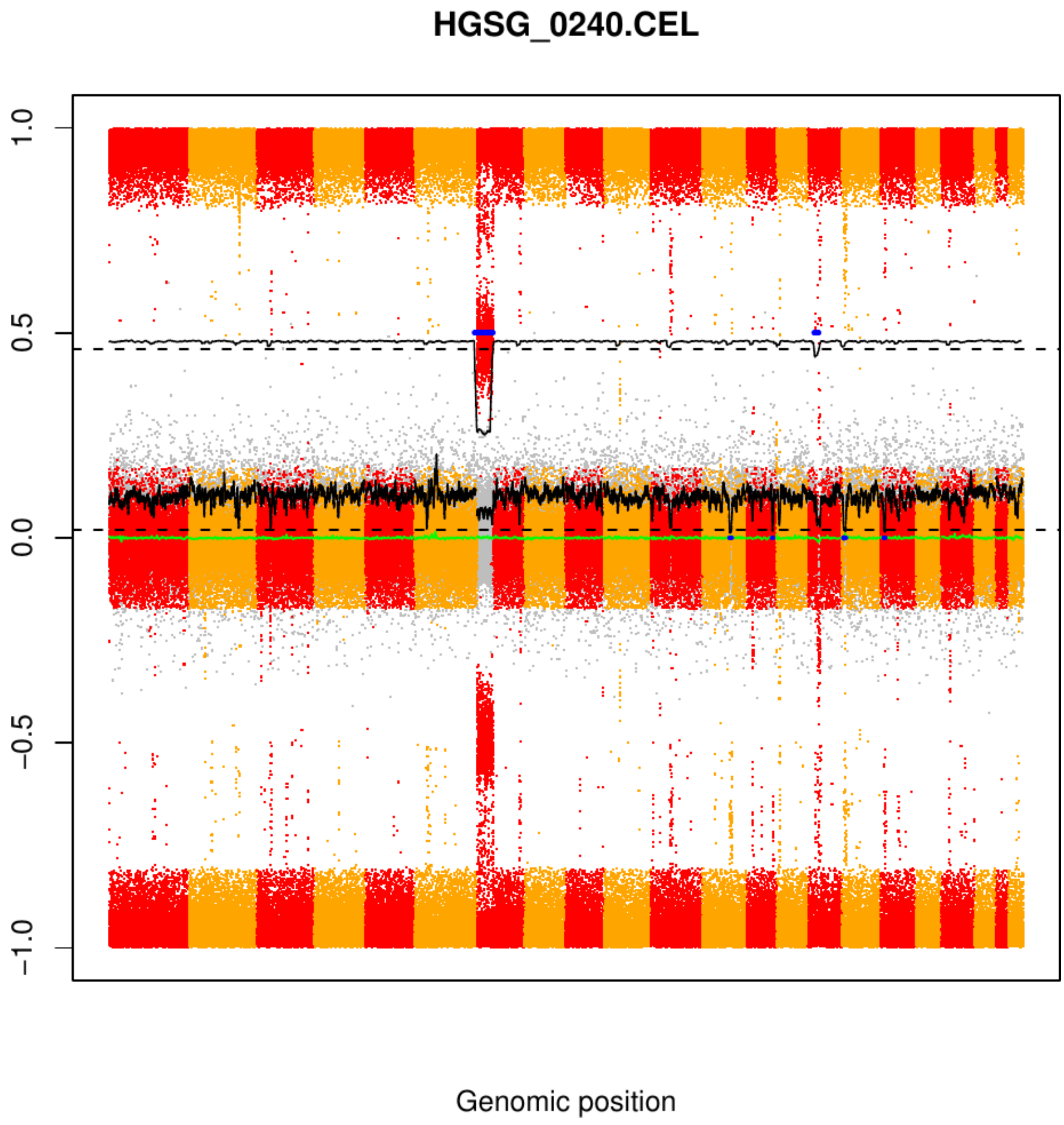
HGSG_0240.CEL
1.0
0.5
0.0
−0.5
−1.0
Genomic position

HNSC_0076.CEL

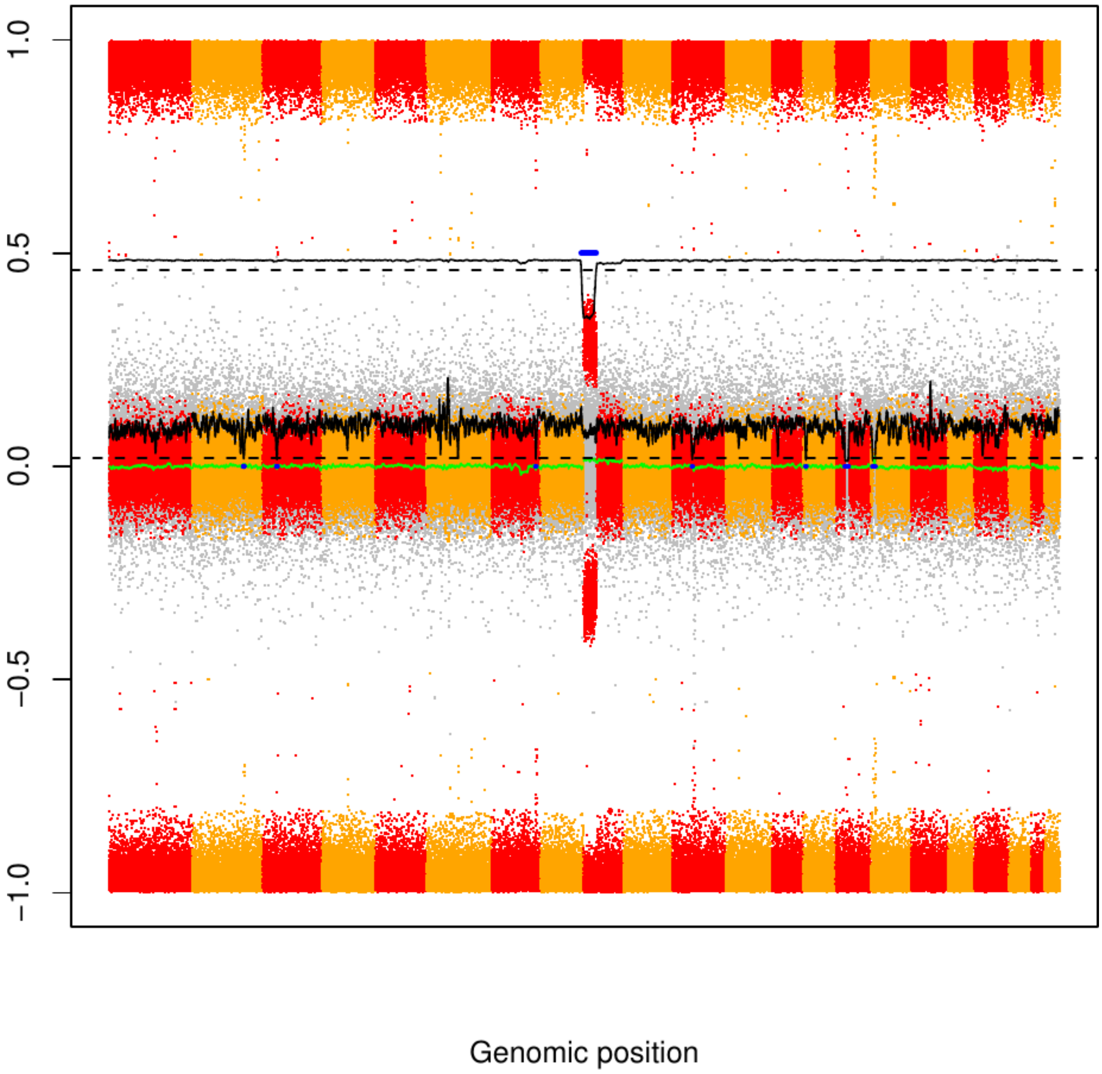

HNSC_0080.CEL

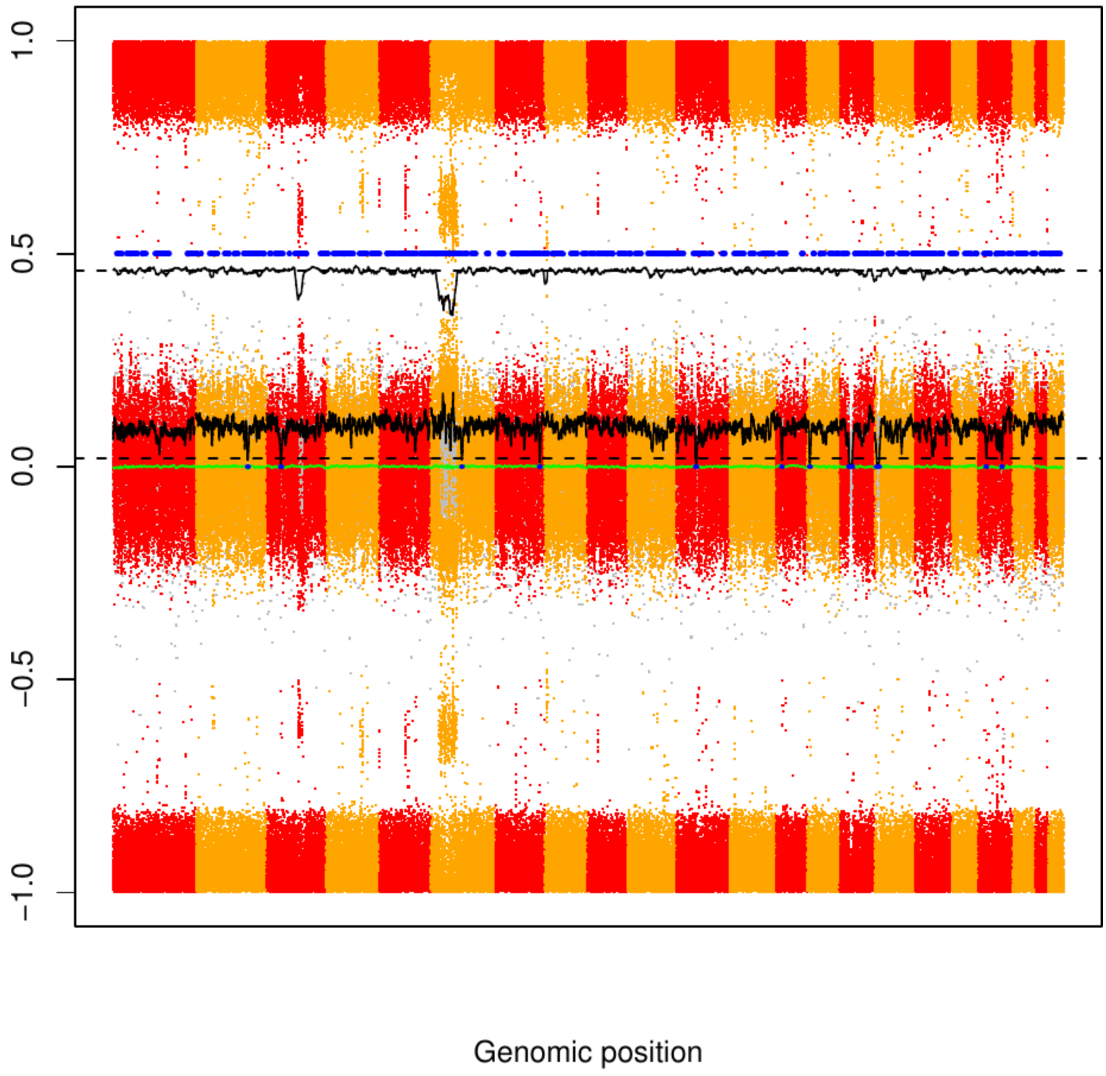

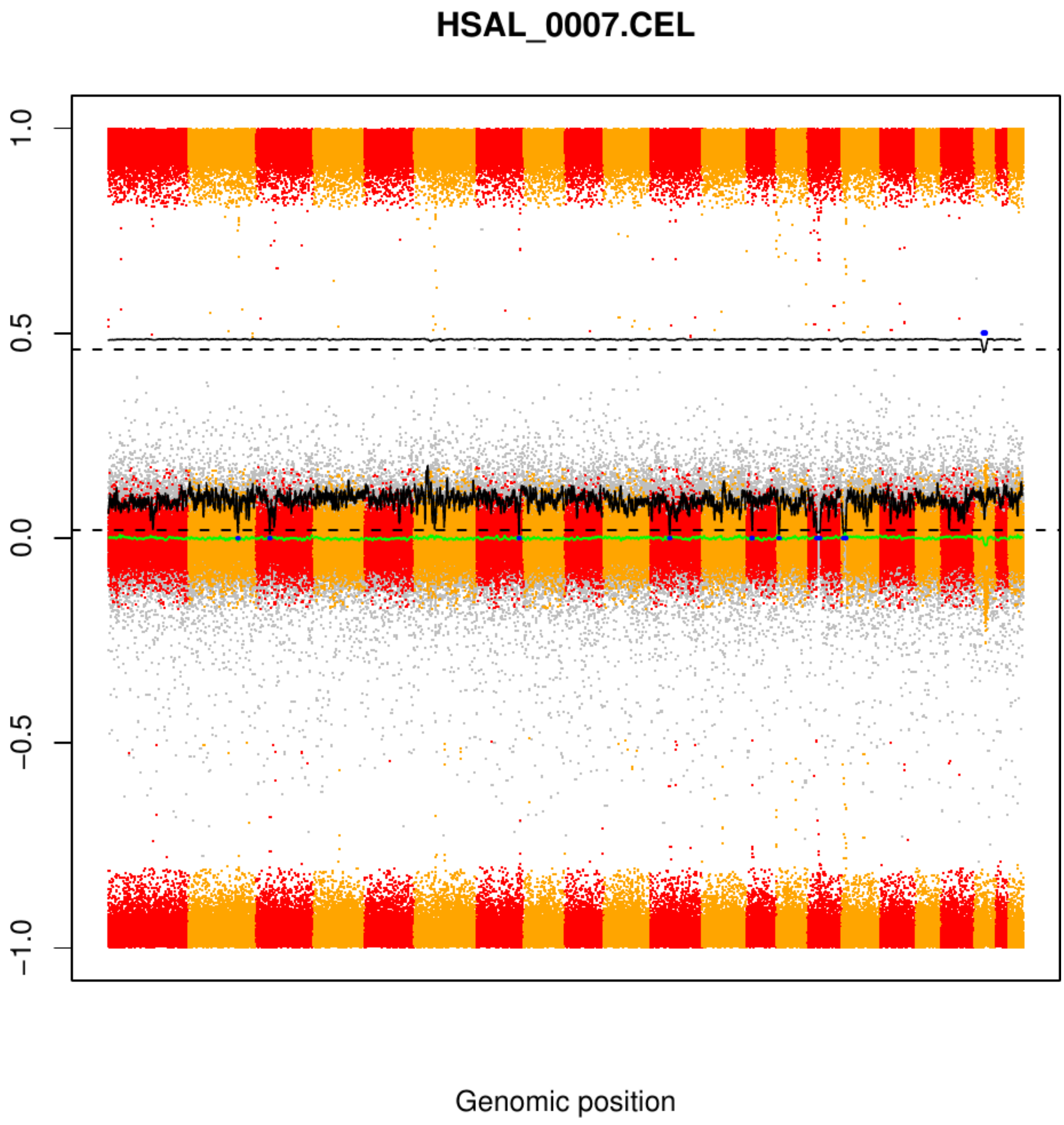

HSAL_0007.CEL
1.0
0.5
0.0
−0.5
−1.0
Genomic position

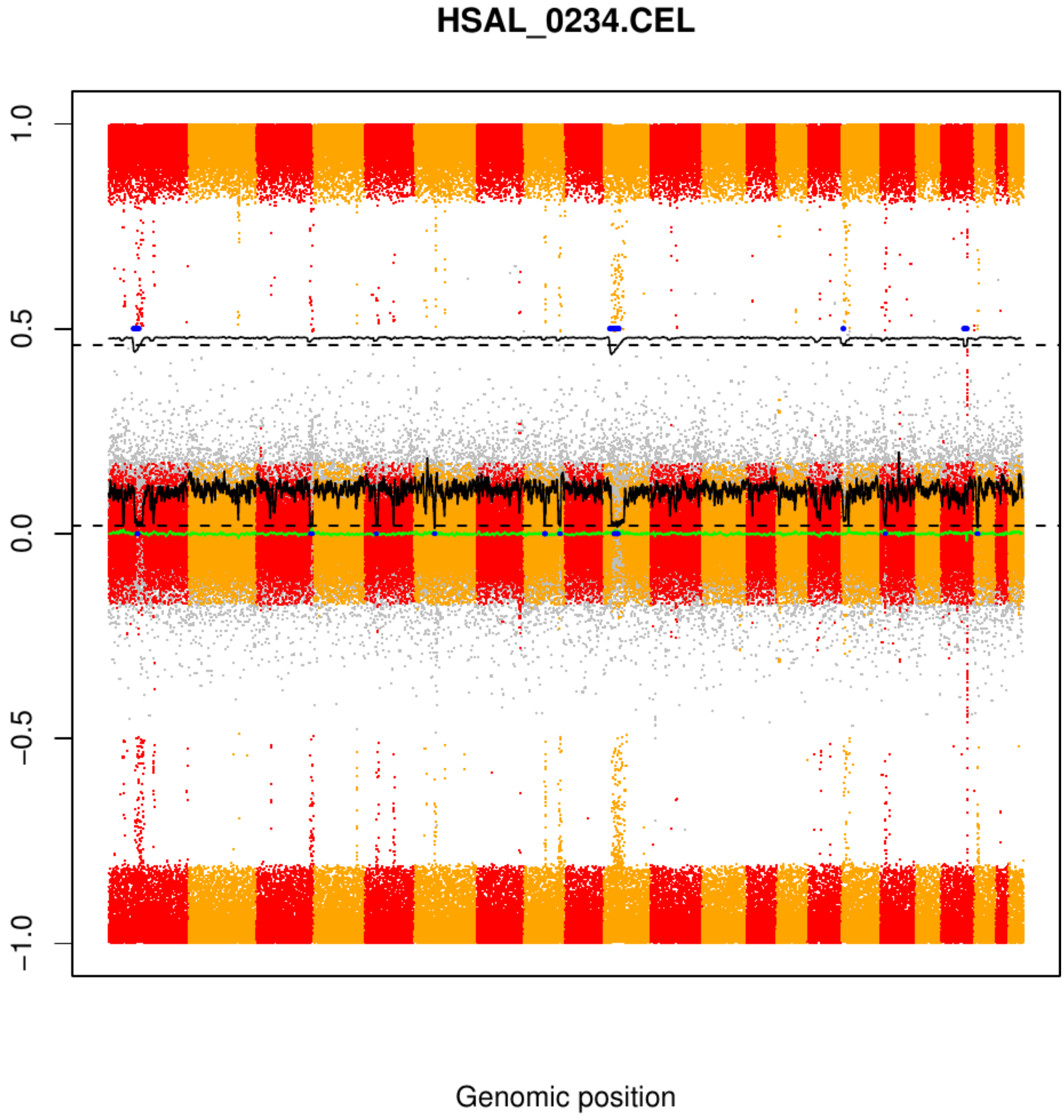
HSAL_0234.CEL
1.0
0.5
0.0
−0.5
−1.0
Genomic position

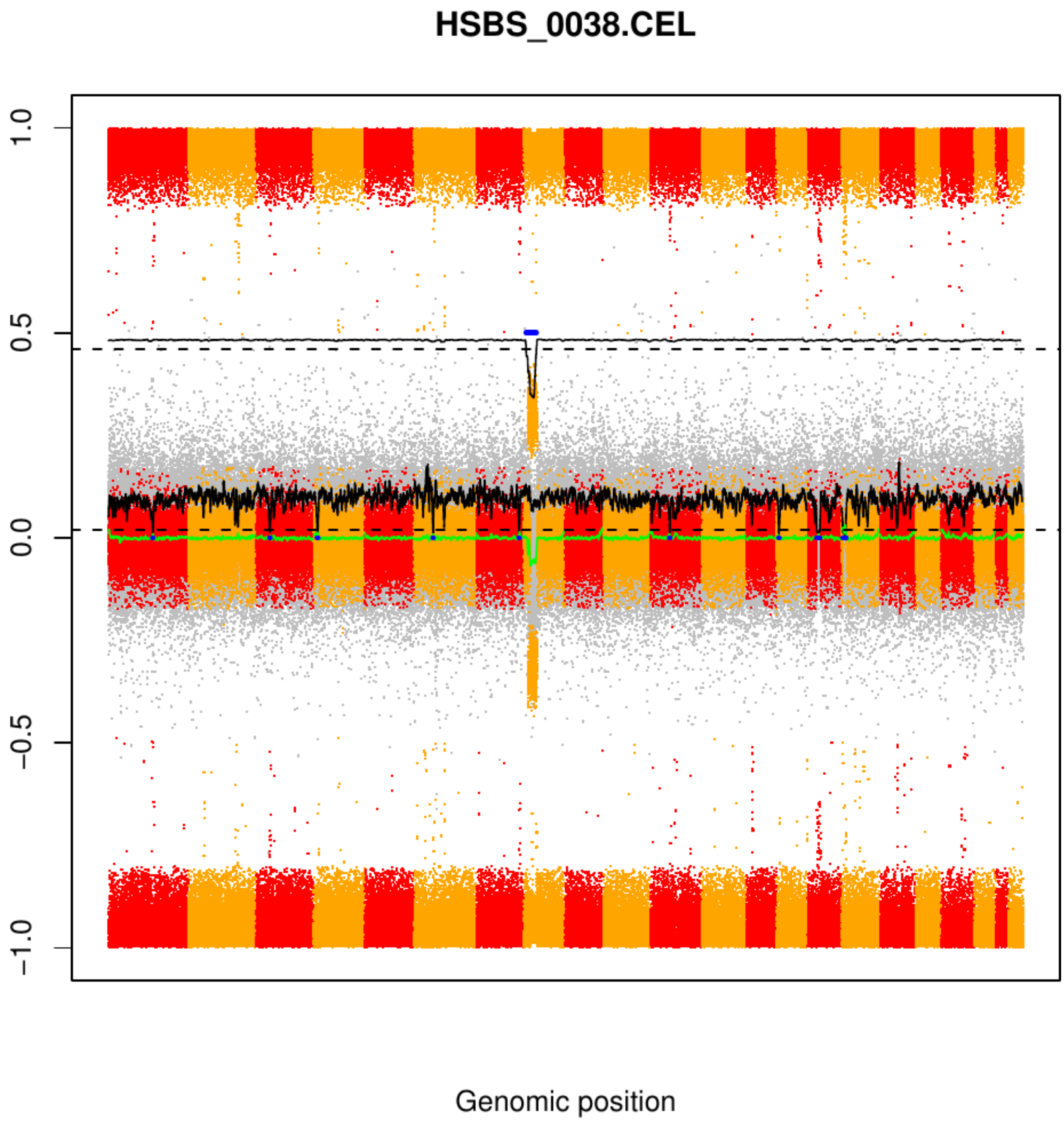
HSBS_0038.CEL
1.0
0.5
0.0
−0.5
−1.0
Genomic position

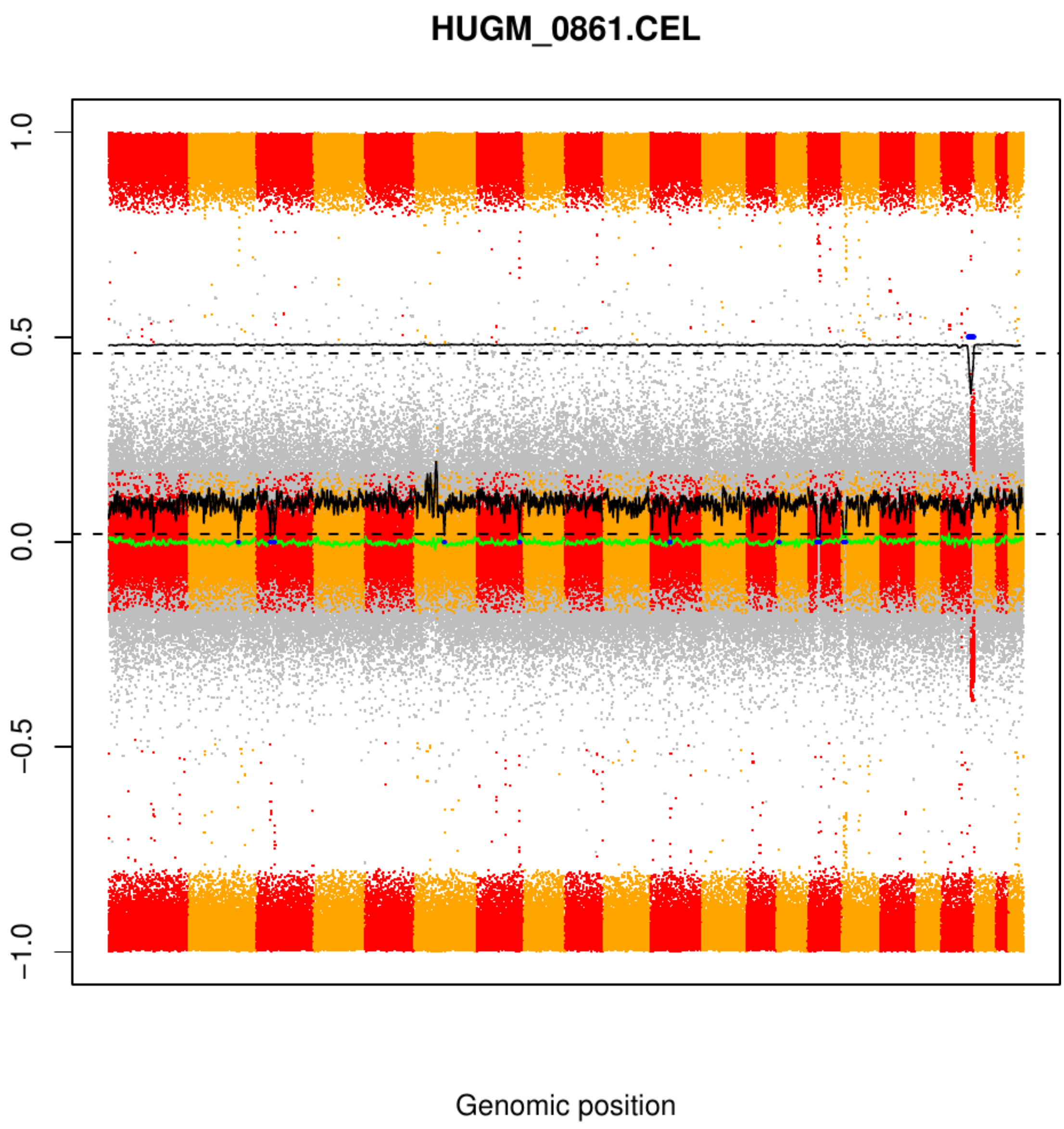
HUGM_0861.CEL
1.0
0.5
0.0
−0.5
−1.0
Genomic position

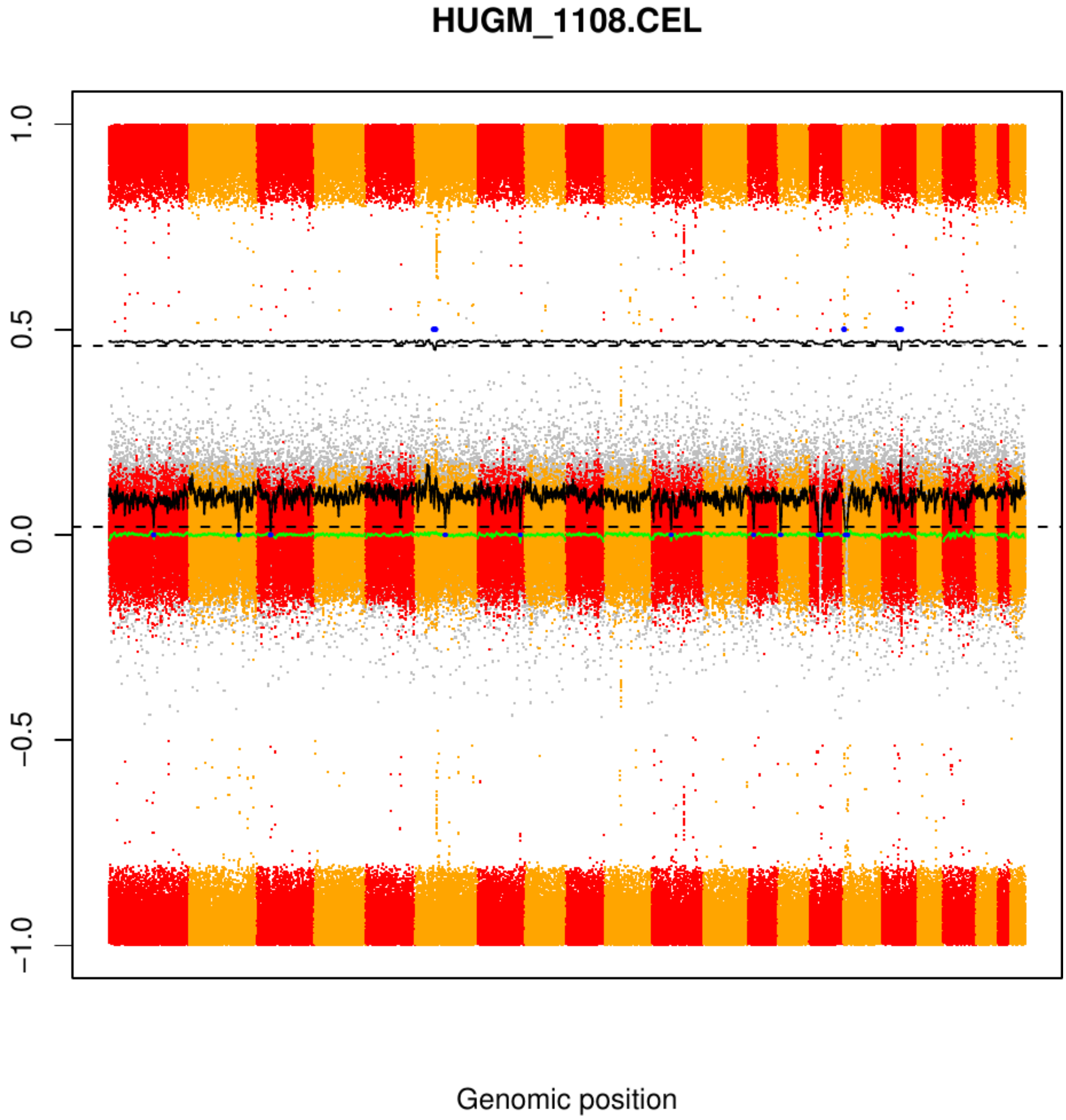
HUGM_1108.CEL
1.0
0.5
0.0
−0.5
−1.0
Genomic position

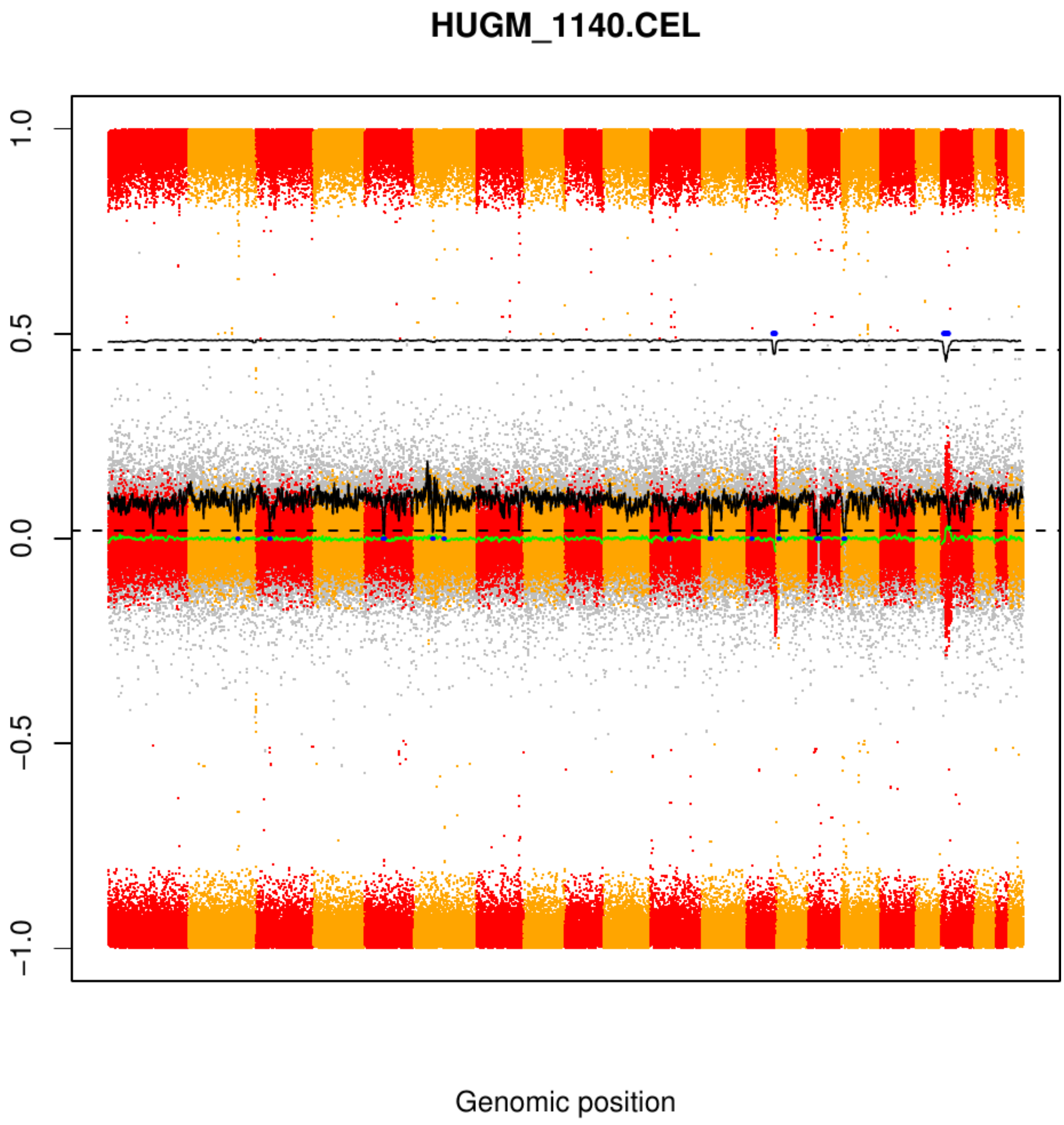
HUGM_1140.CEL
1.0
0.5
0.0
−0.5
−1.0
Genomic position

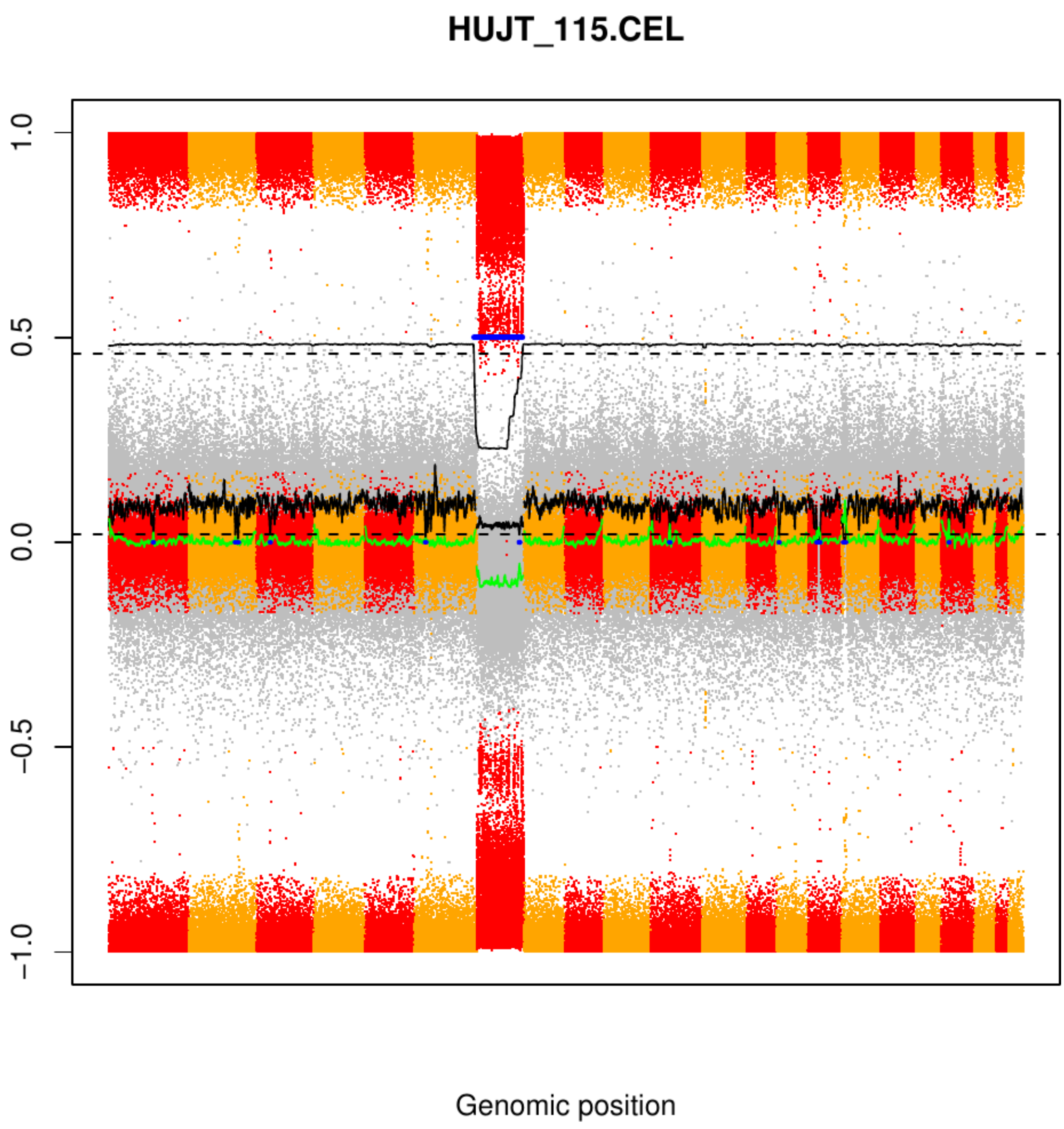
HUJT_115.CEL
1.0
0.5
0.0
−0.5
−1.0
Genomic position

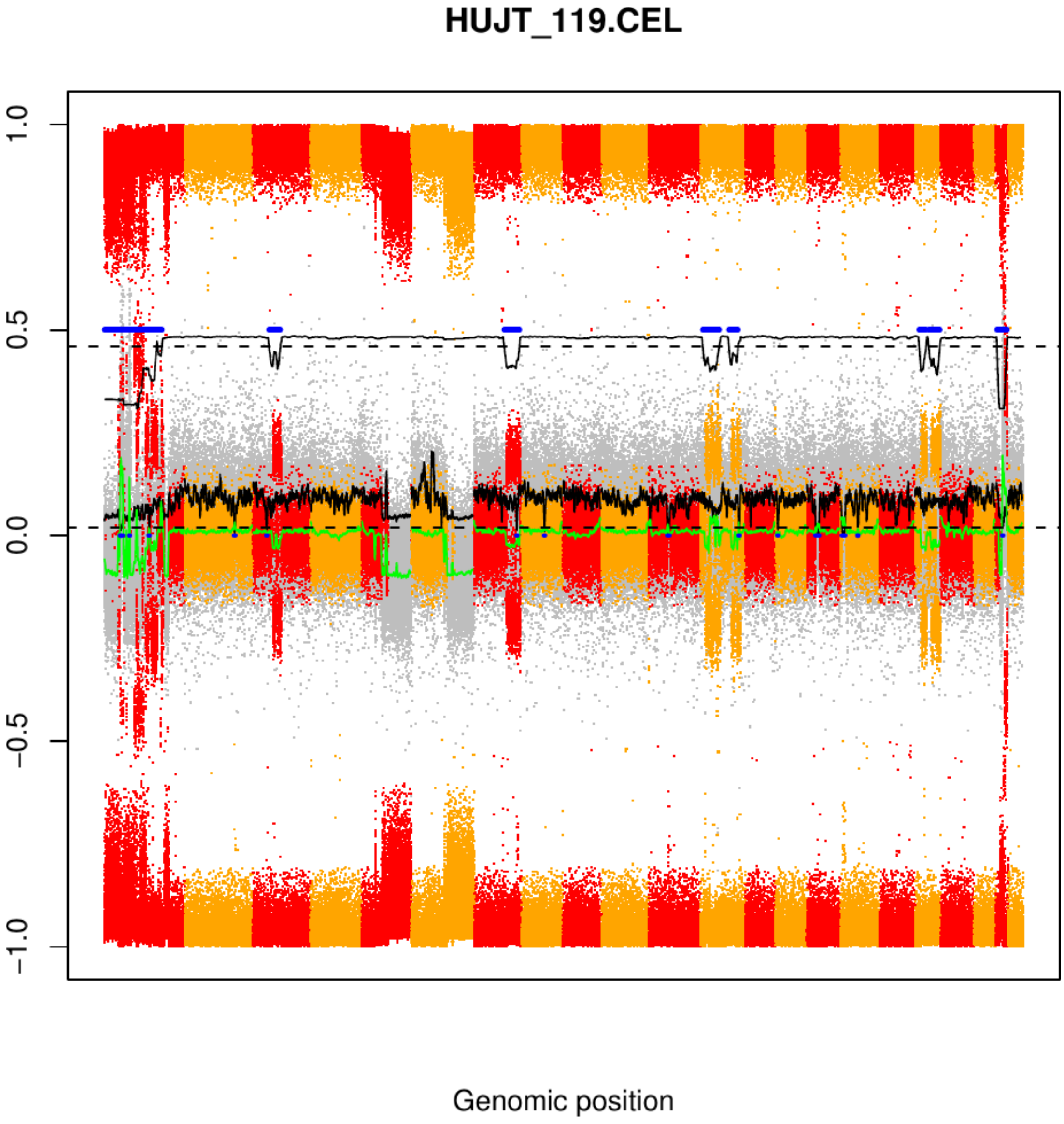
HUJT_119.CEL
1.0
0.5
0.0
−0.5
−1.0
Genomic position

HUJT_139.CEL

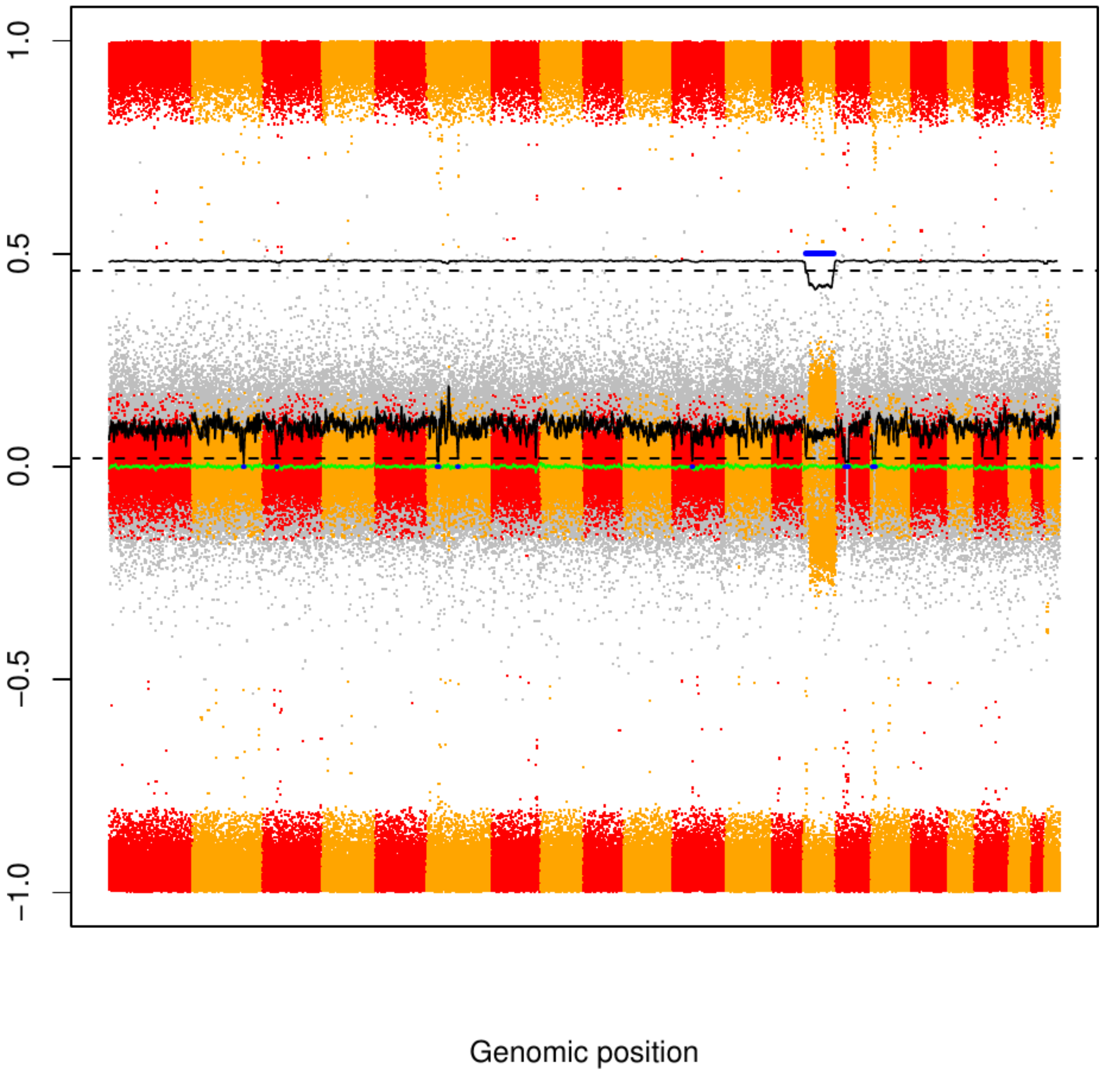

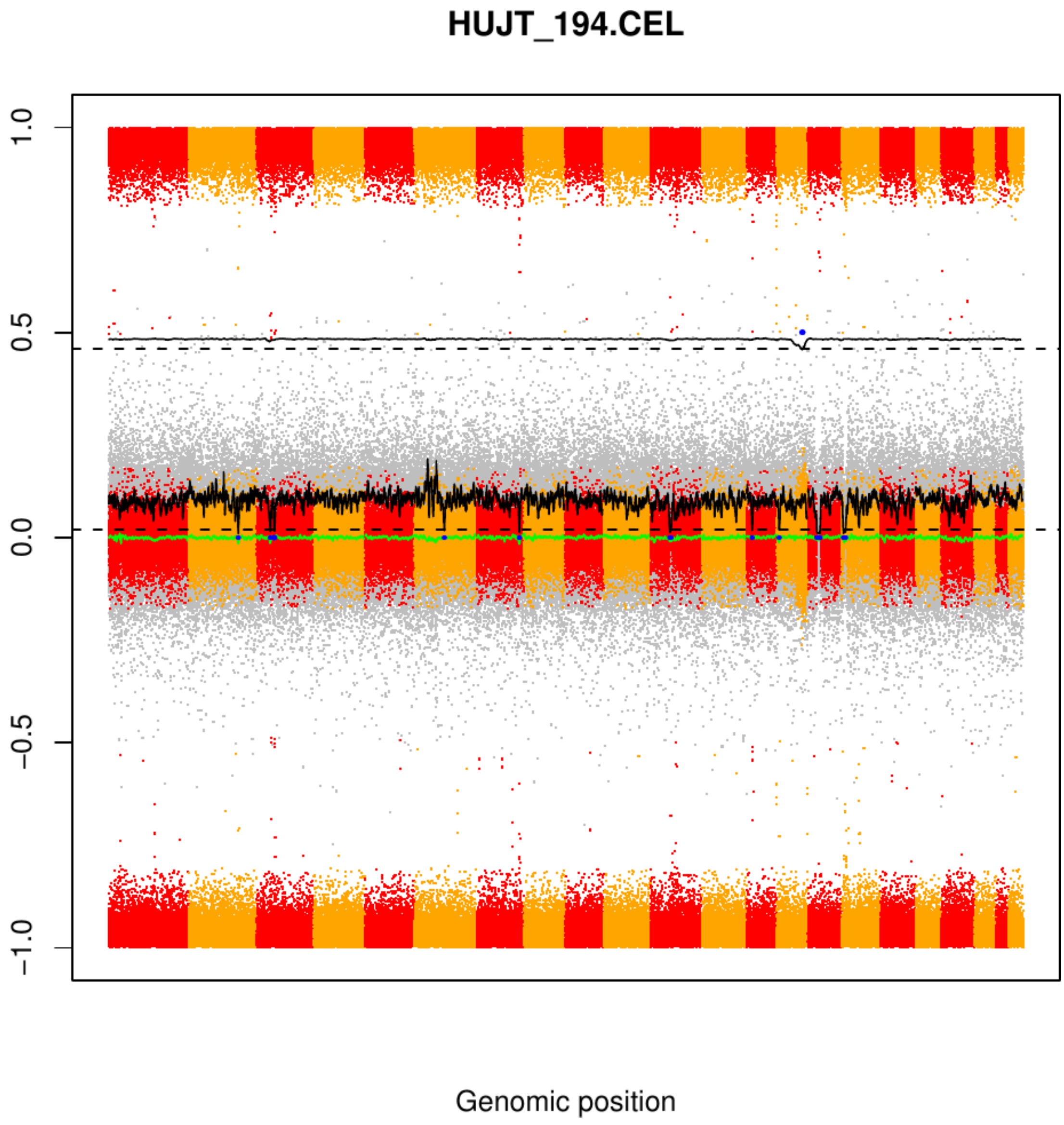
HUJT_194.CEL
1.0
0.5
0.0
−0.5
−1.0
Genomic position

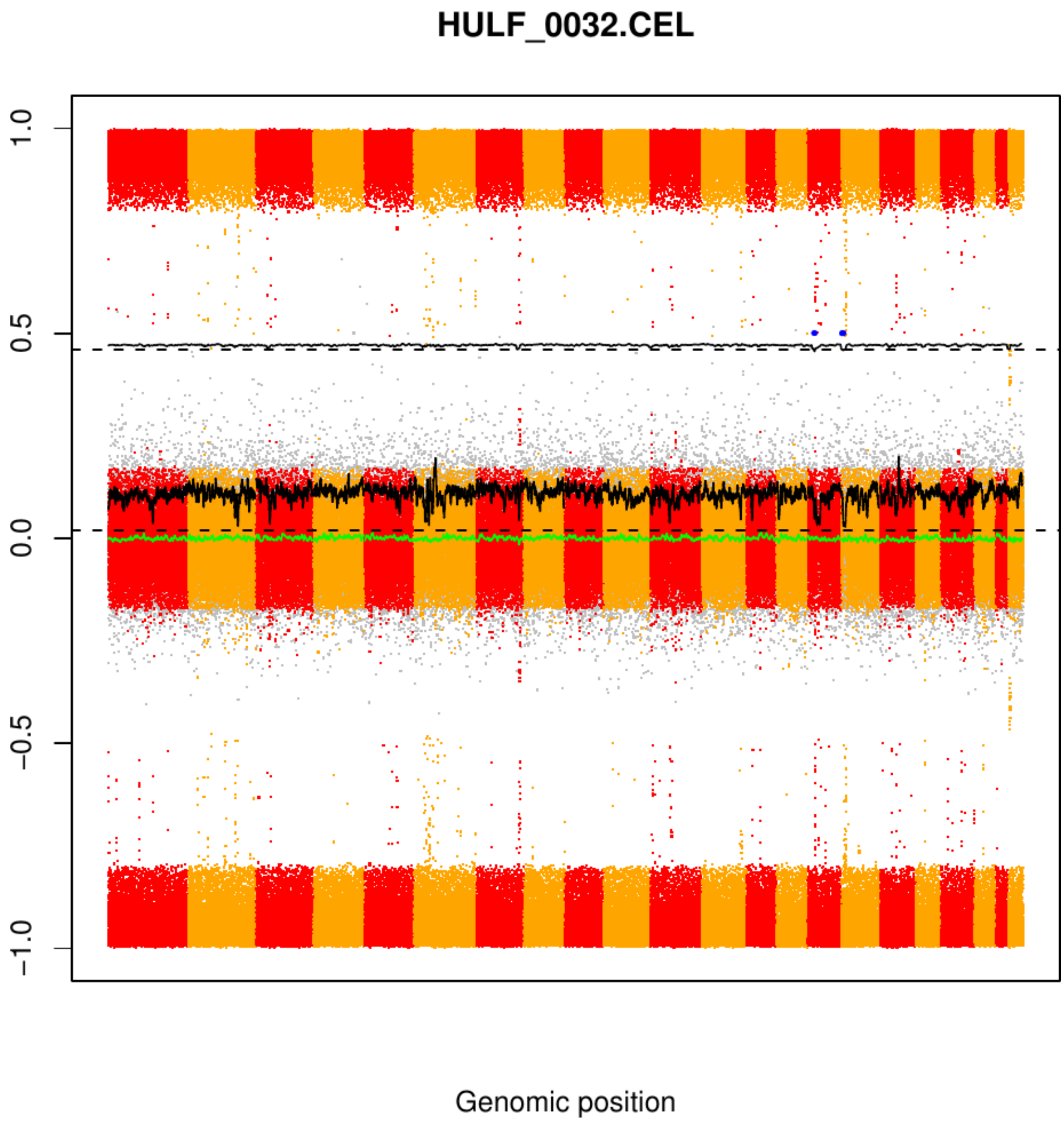
HULF_0032.CEL
1.0
0.5
0.0
−0.5
−1.0
Genomic position

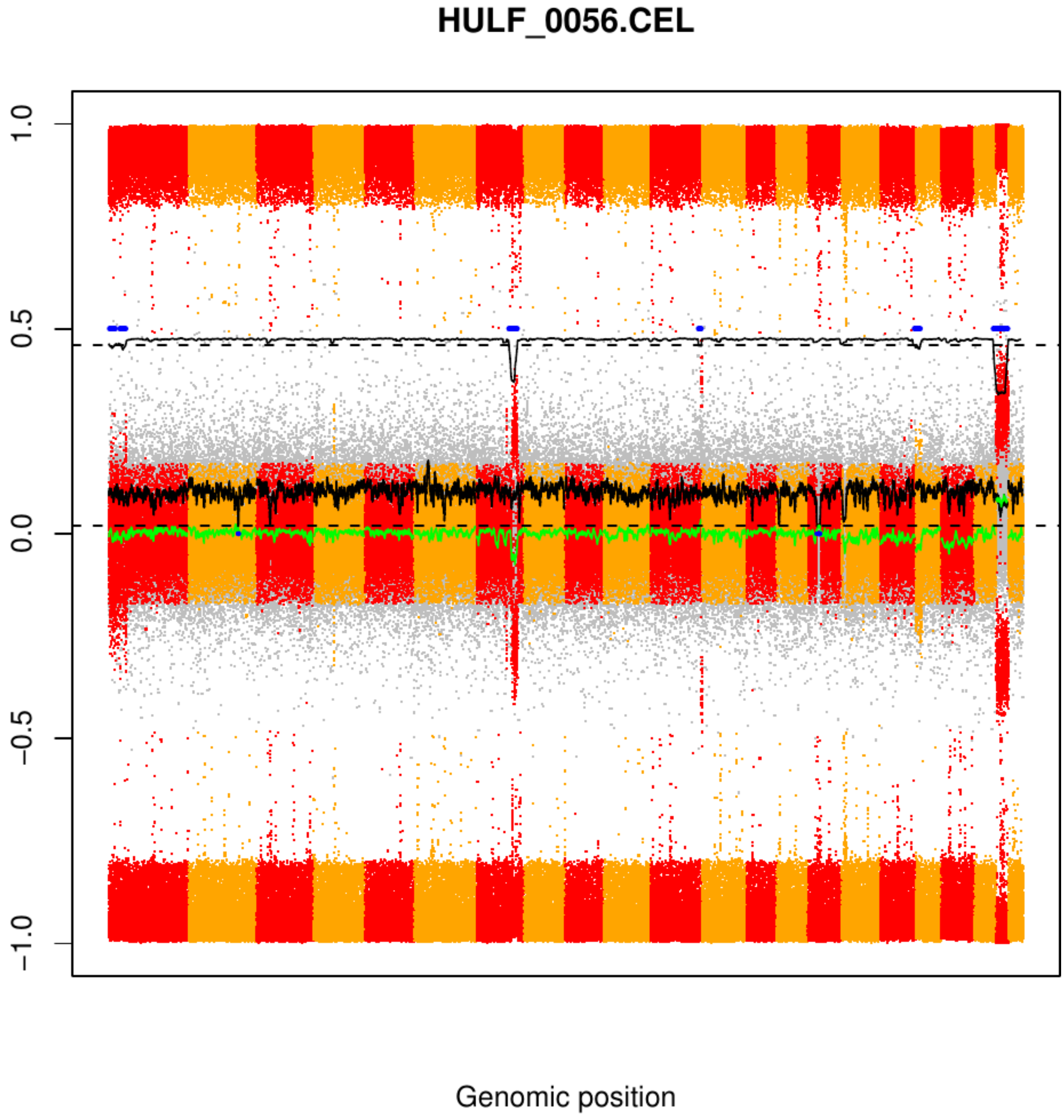
HULF_0056.CEL
1.0
0.5
0.0
−0.5
−1.0
Genomic position

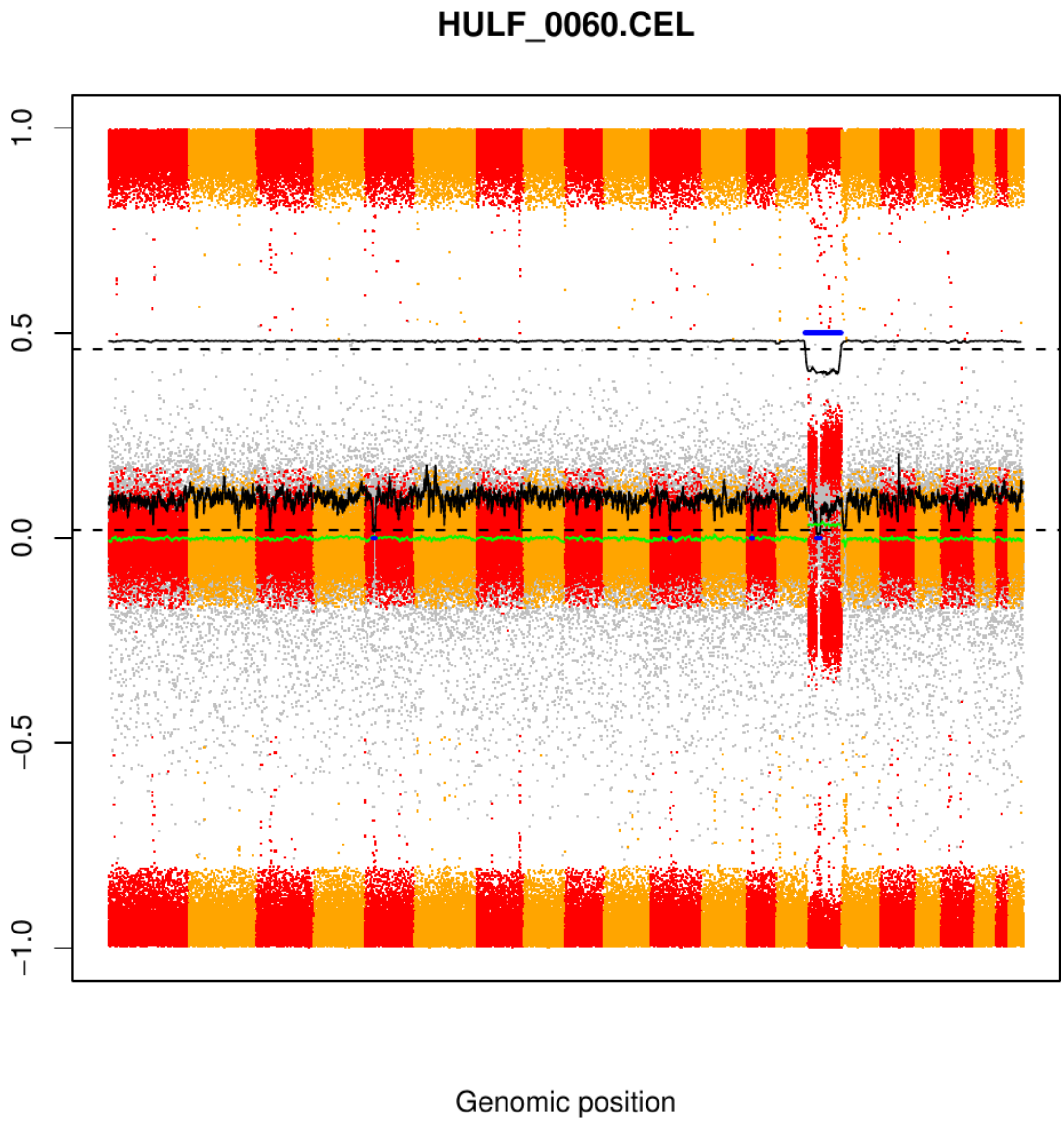
HULF_0060.CEL
1.0
0.5
0.0
−0.5
−1.0
Genomic position

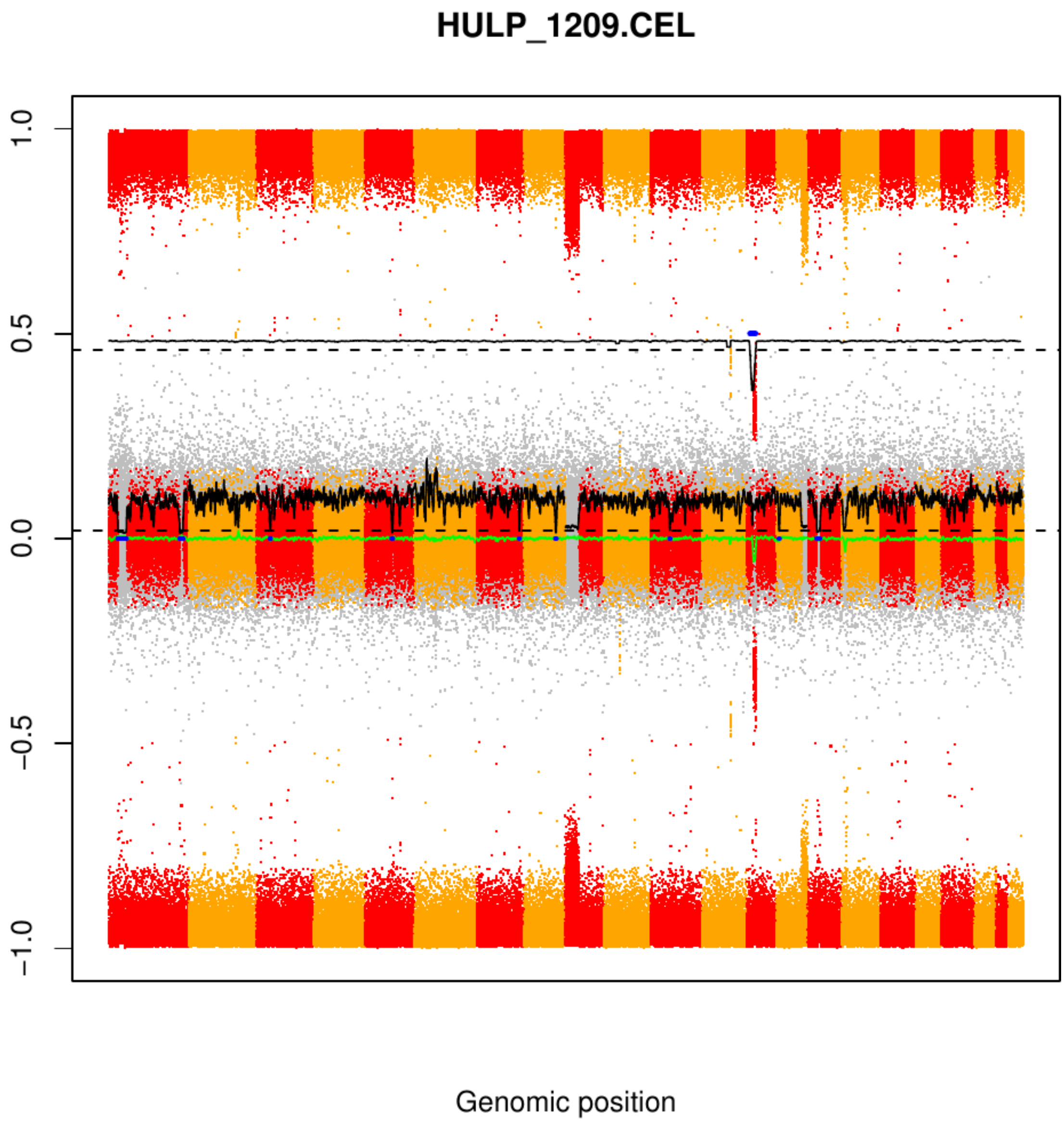
HULP_1209.CEL
1.0
0.5
0.0
−0.5
−1.0
Genomic position

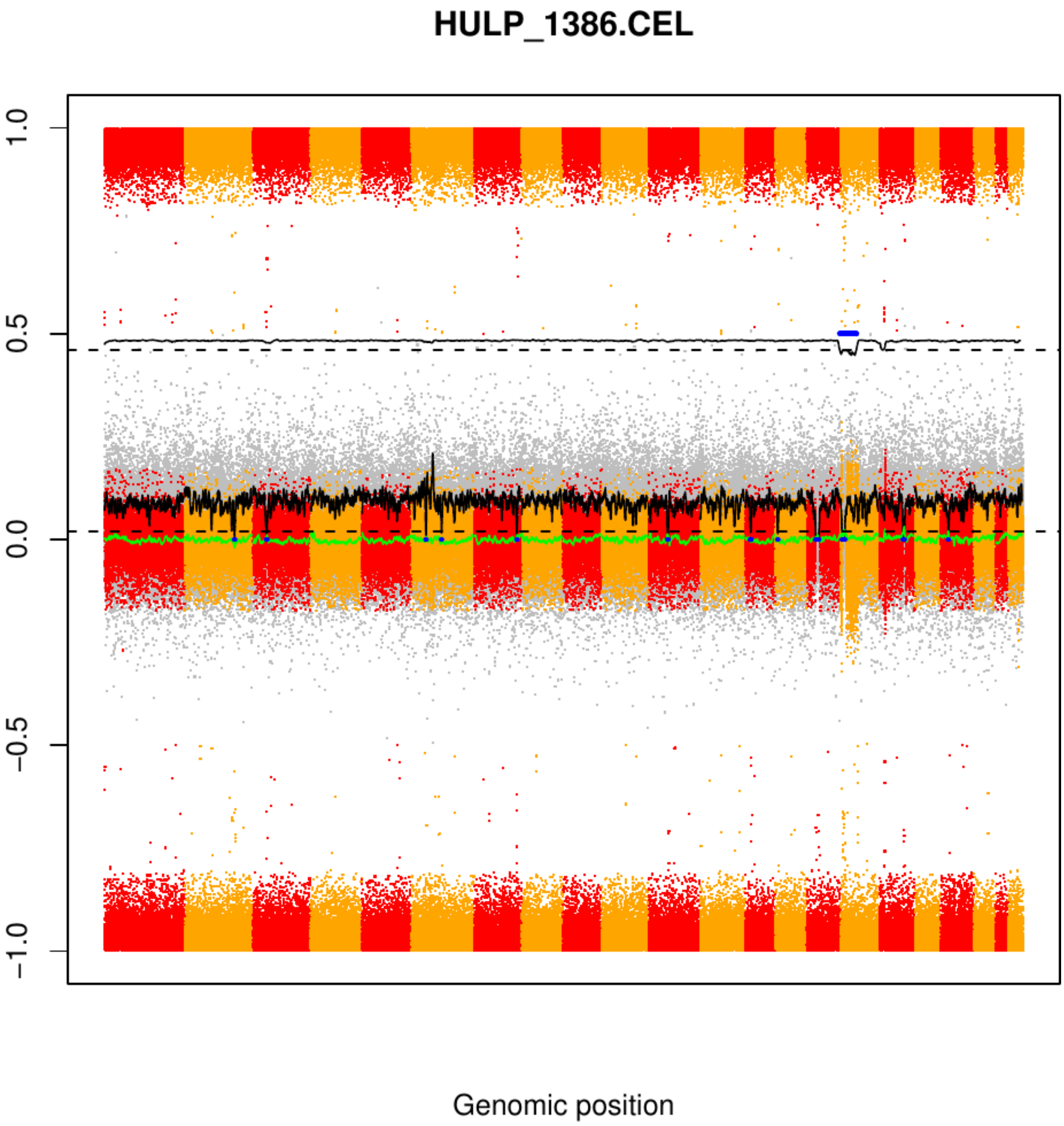

HULP_1386.CEL
1.0
0.5
0.0
−0.5
−1.0
Genomic position

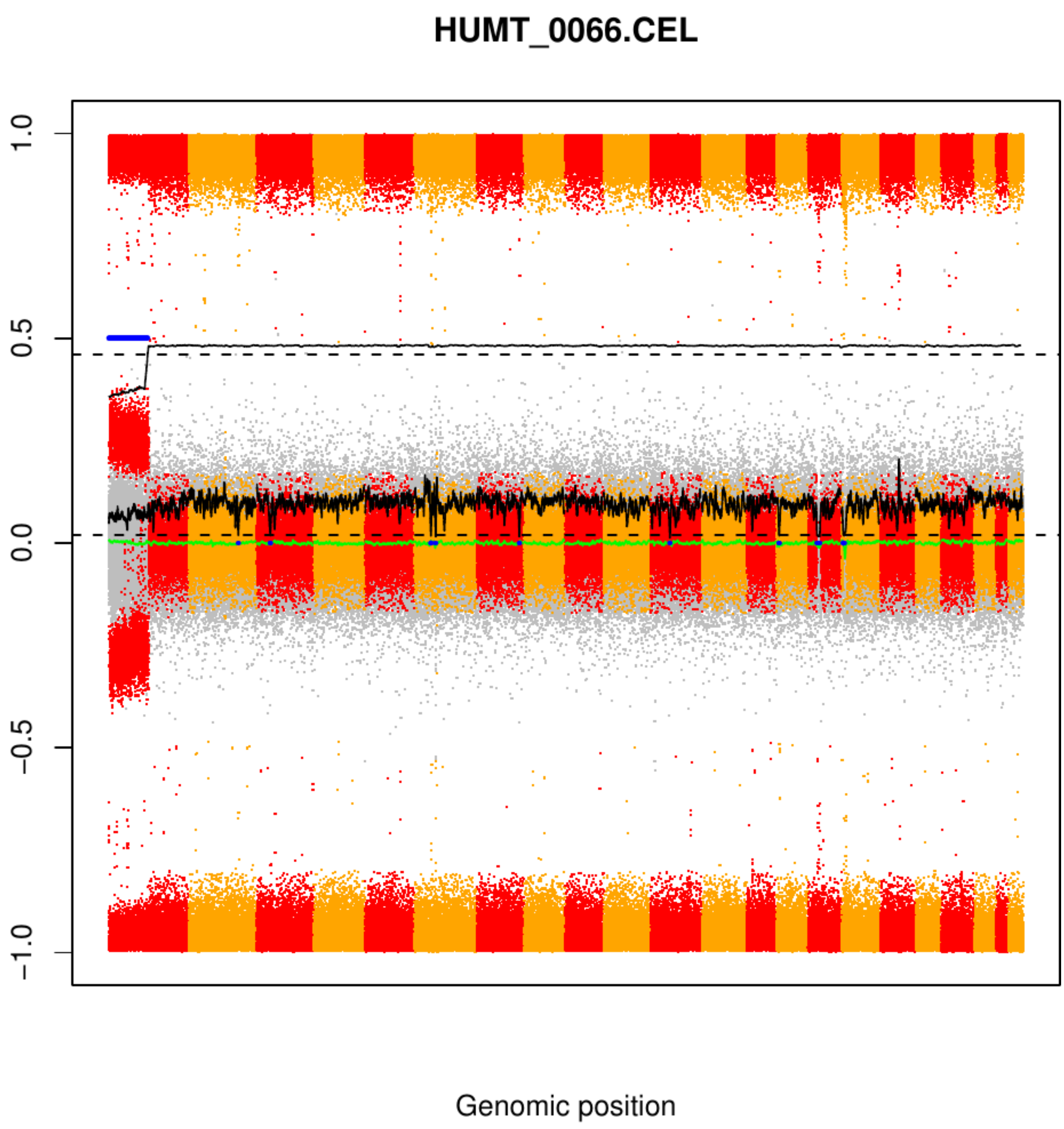
HUMT_0066.CEL
1.0
0.5
0.0
−0.5
−1.0
Genomic position

HUMT_0333.CEL

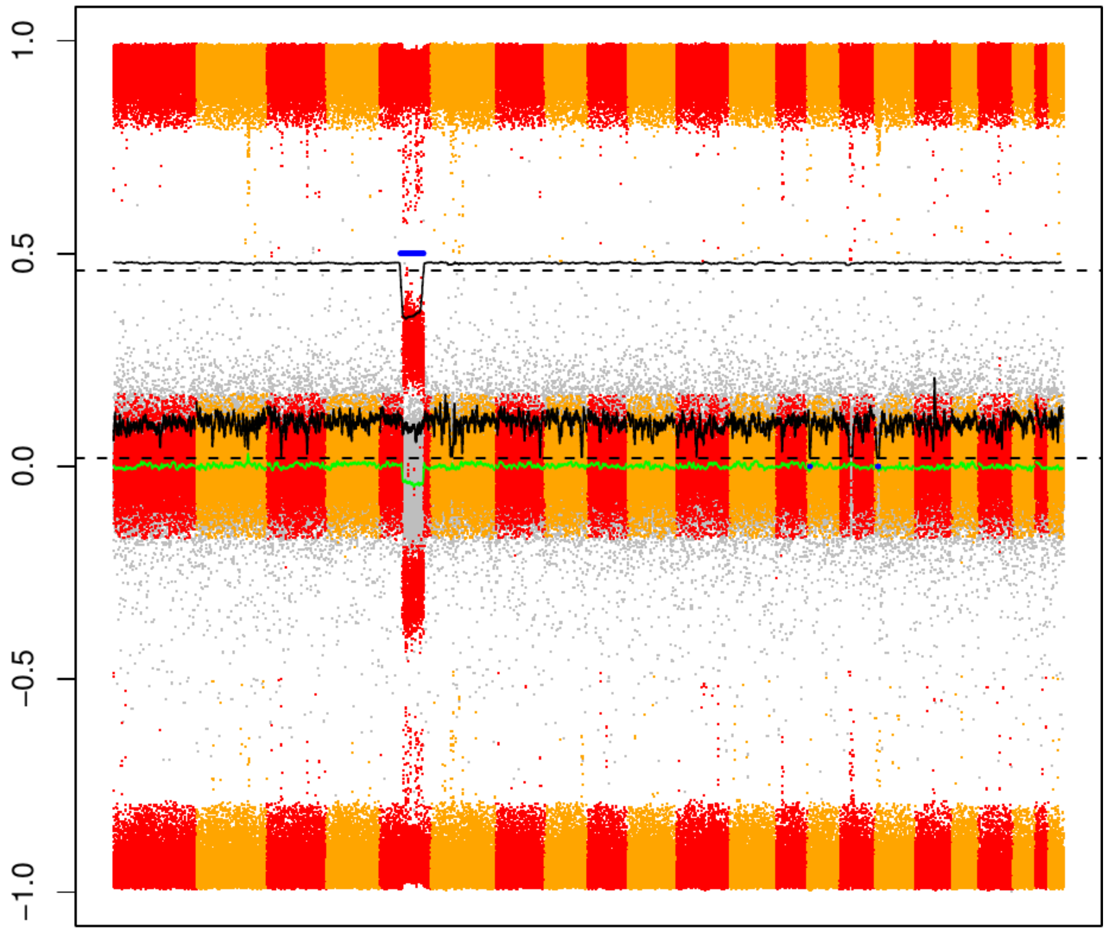

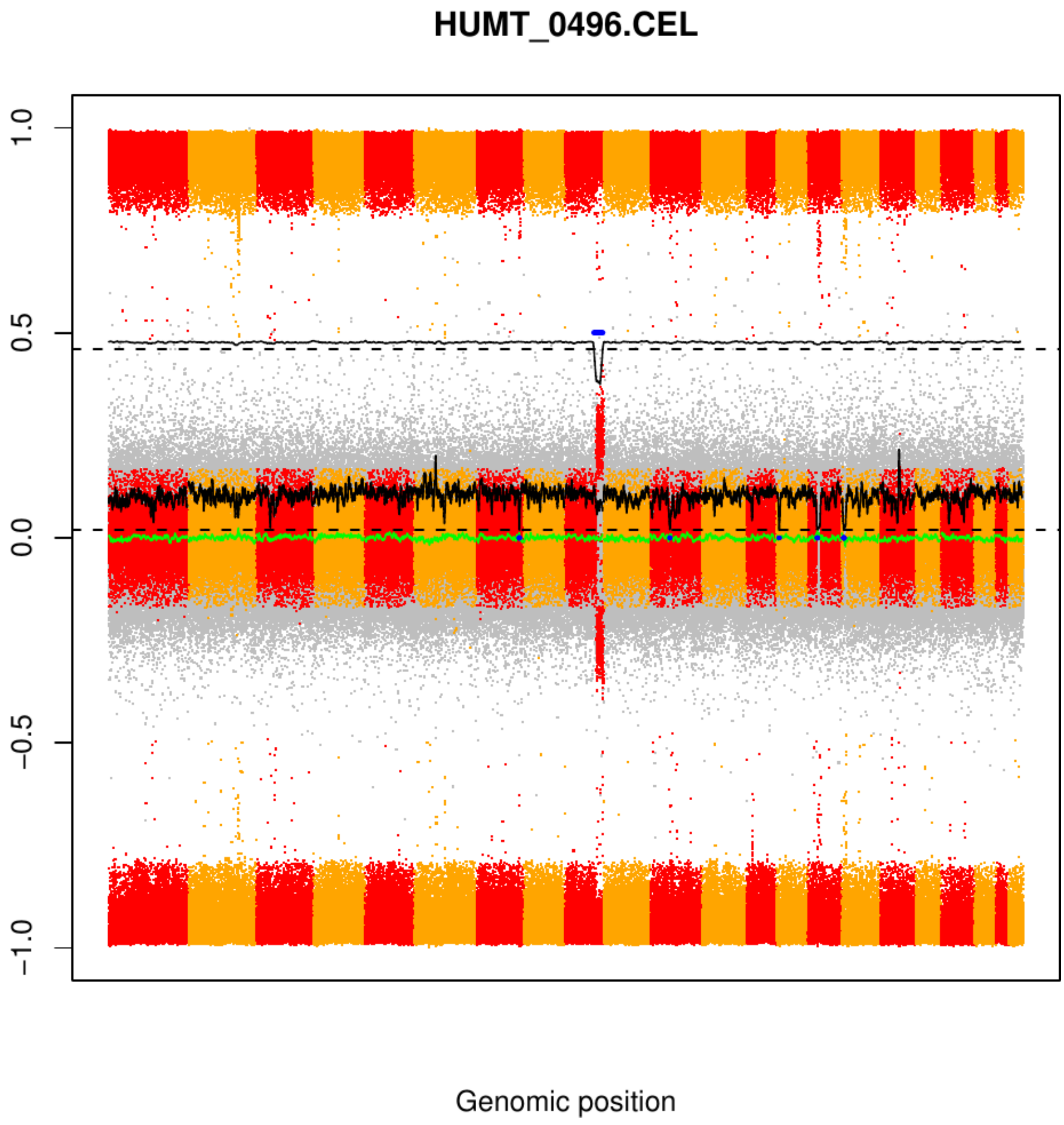
HUMT_0496.CEL
1.0
0.5
0.0
−0.5
−1.0
Genomic position

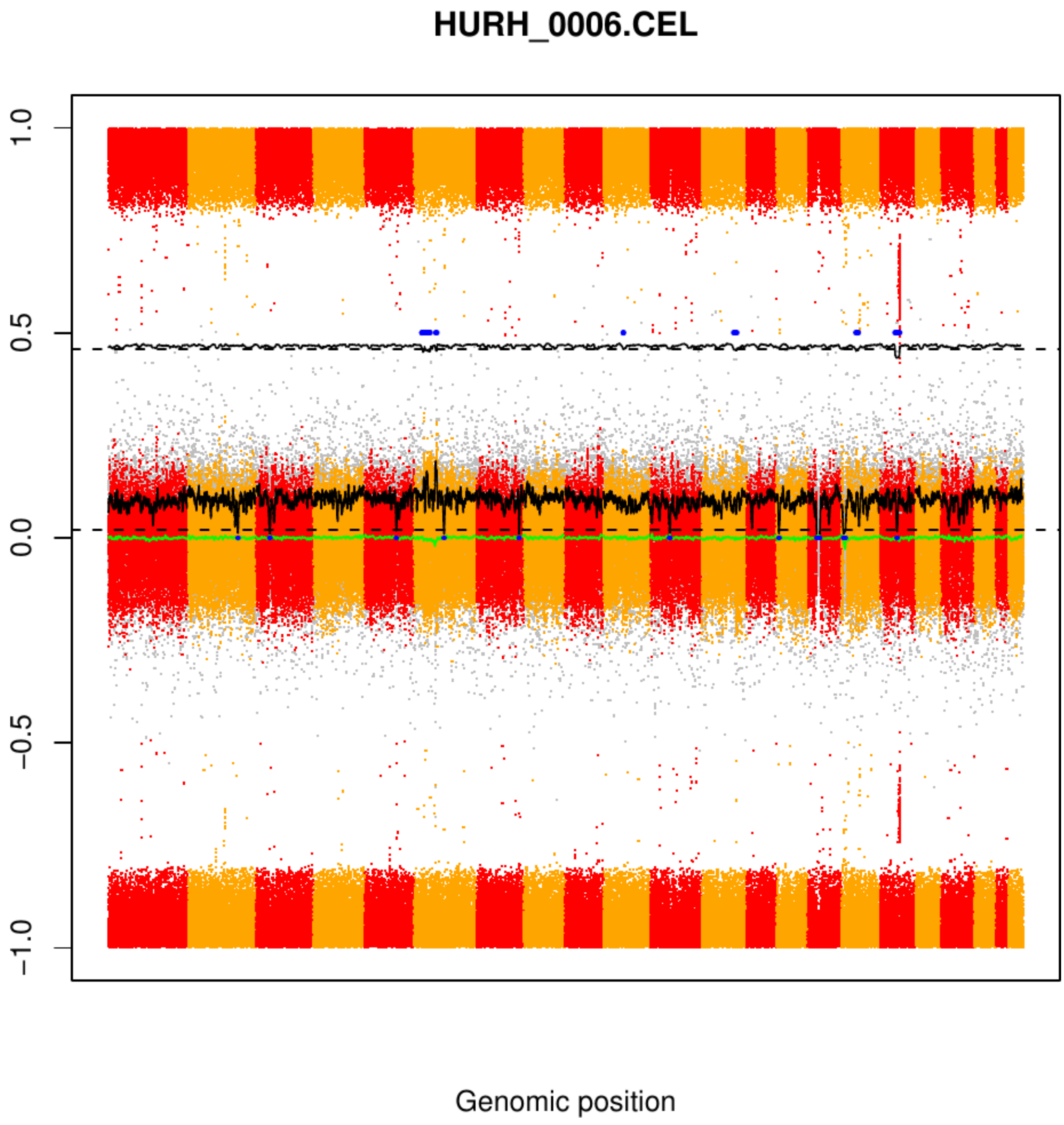
HURH_0006.CEL
1.0
0.5
0.0
−0.5
−1.0
Genomic position

HURH_0050.CEL

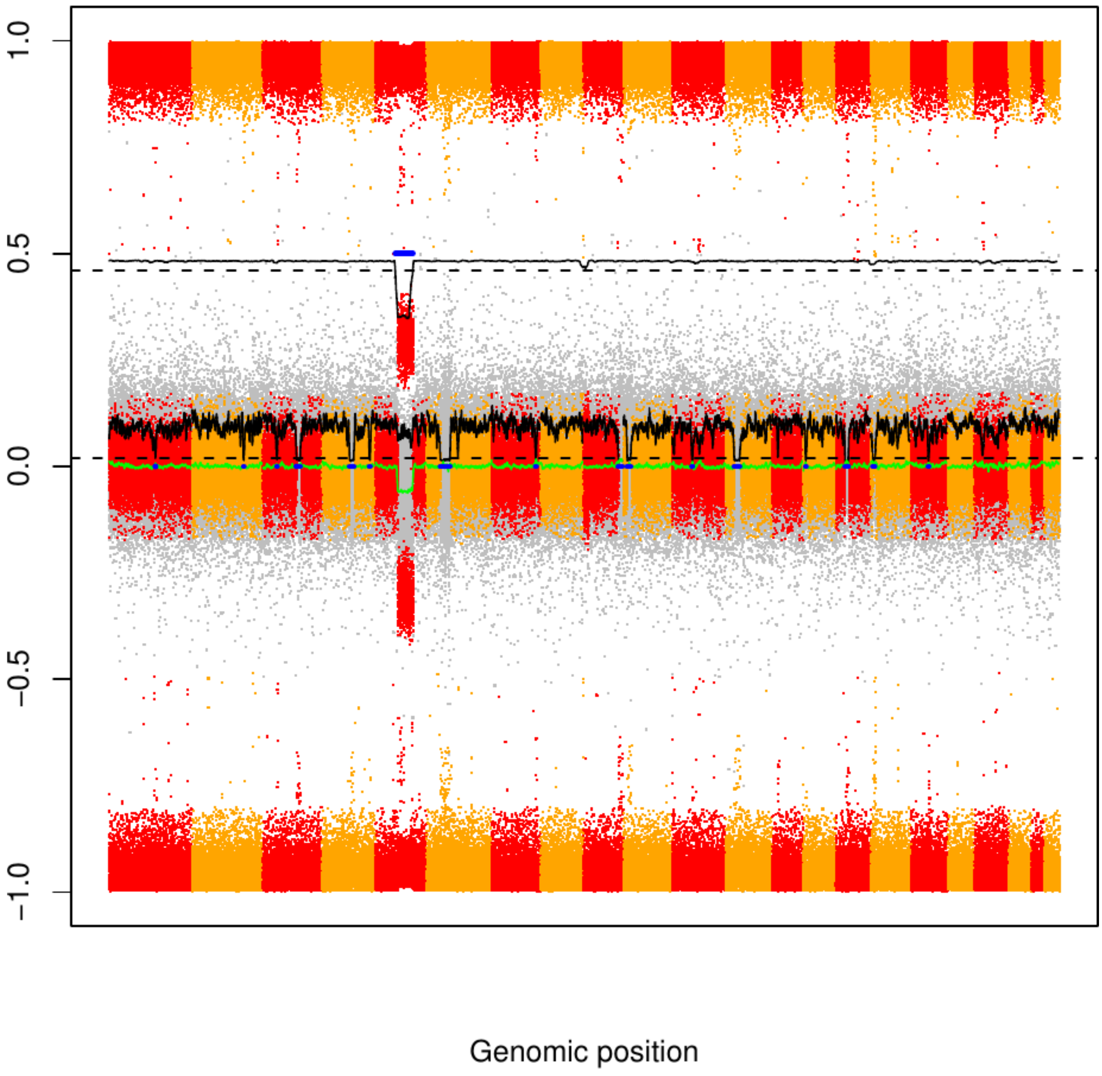

HURH_0122.CEL

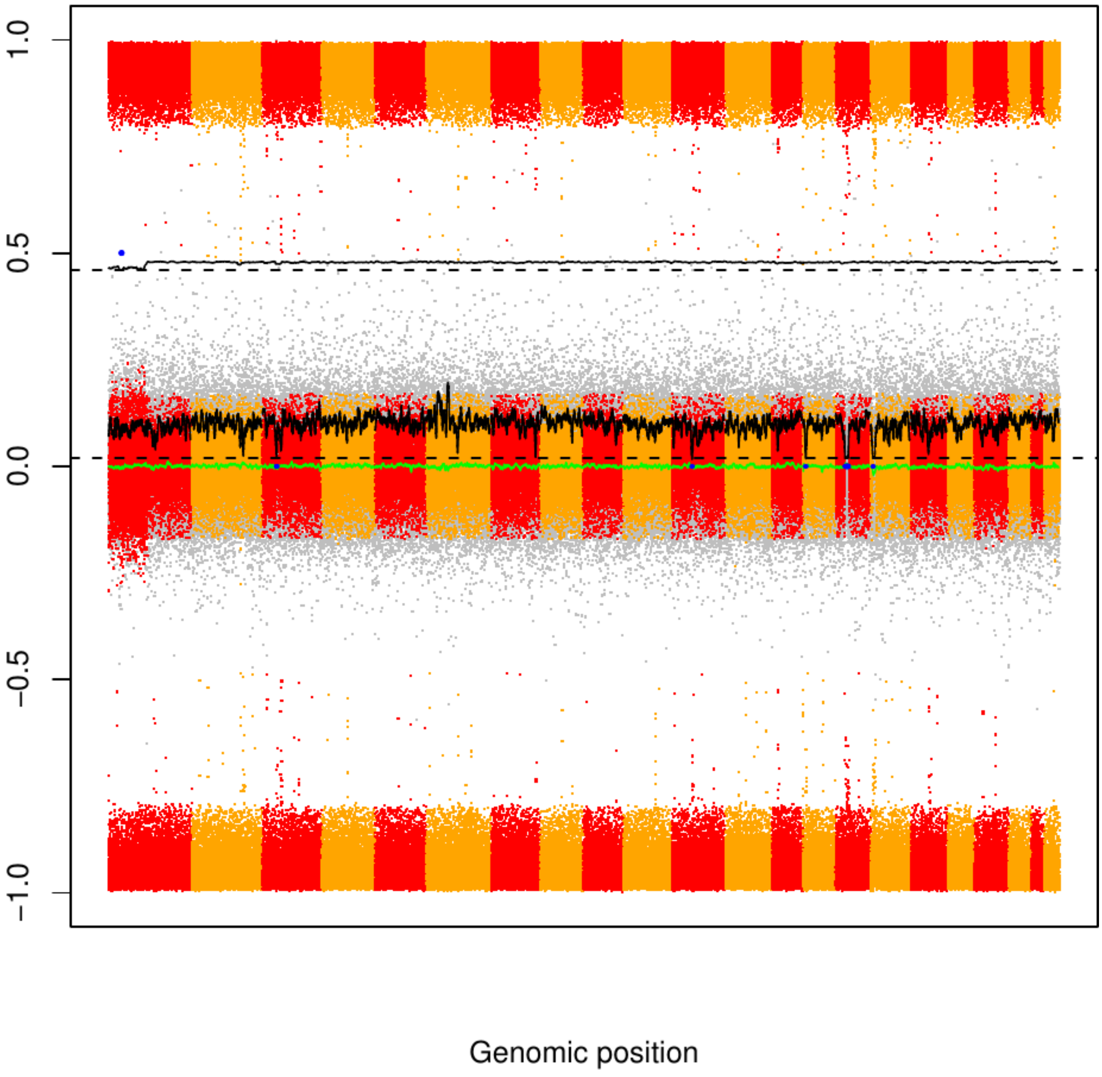

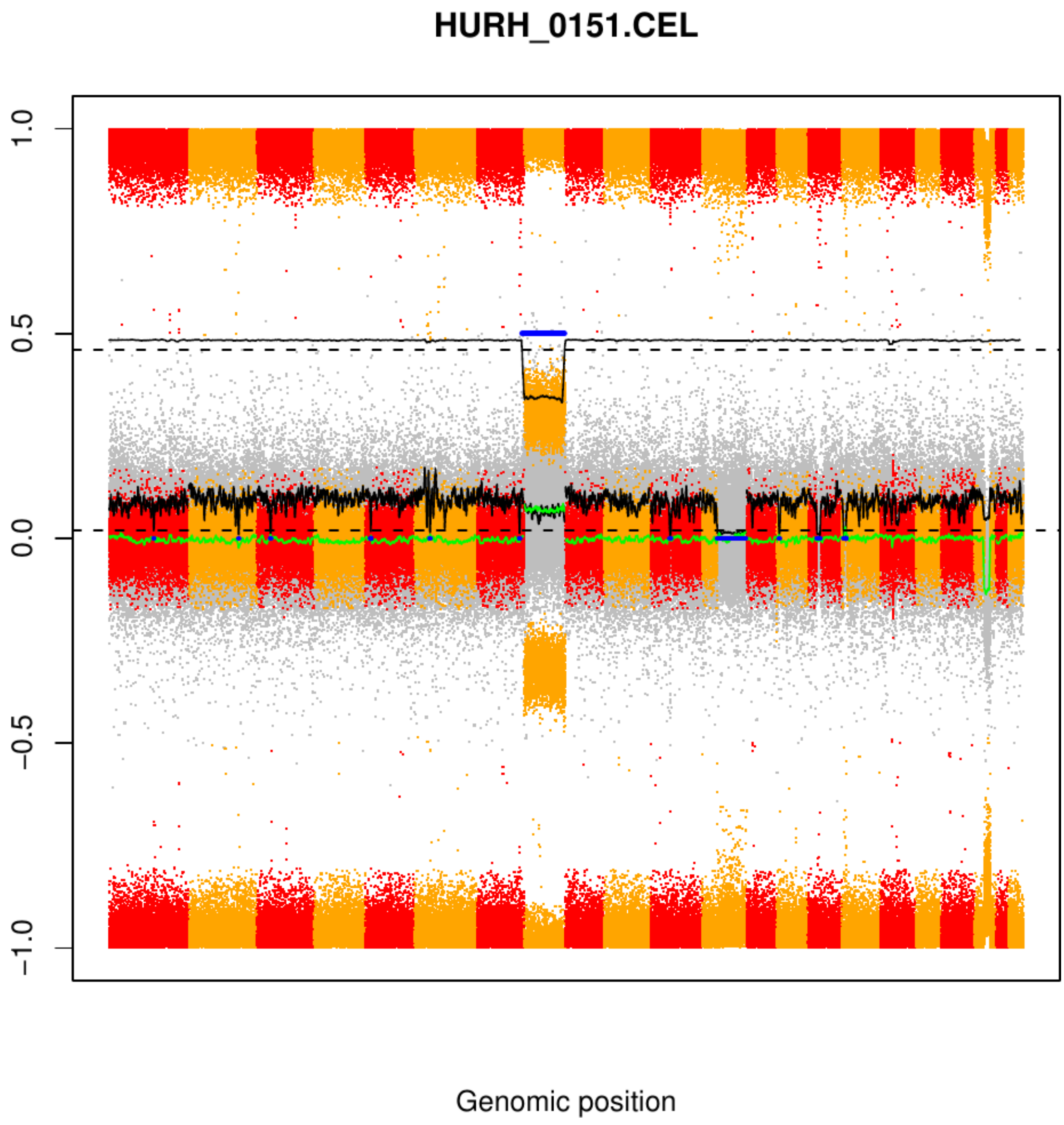
HURH_0151.CEL
1.0
0.5
0.0
−0.5
−1.0
Genomic position

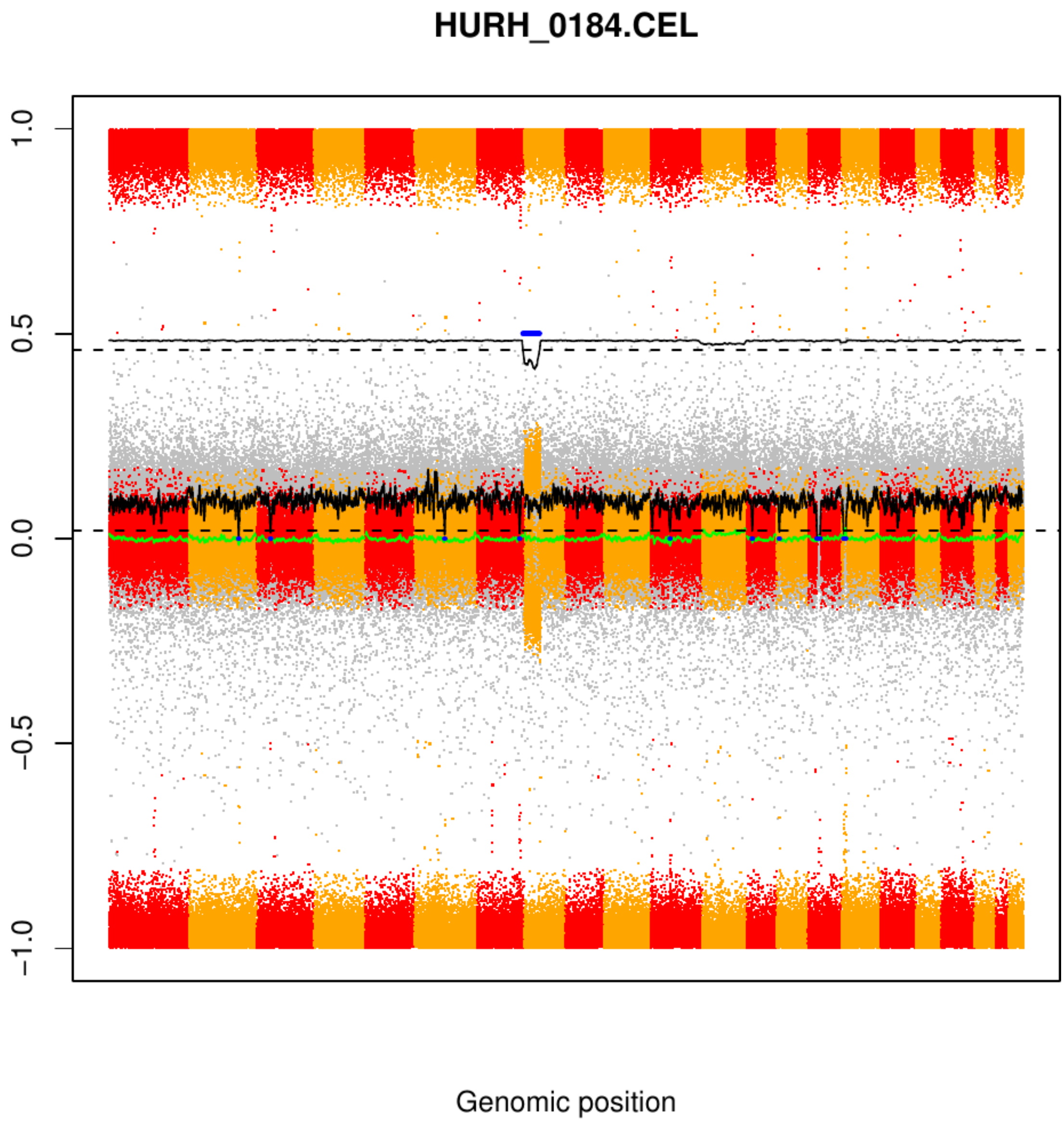
HURH_0184.CEL
1.0
0.5
0.0
−0.5
−1.0
Genomic position

HURH_0243.CEL

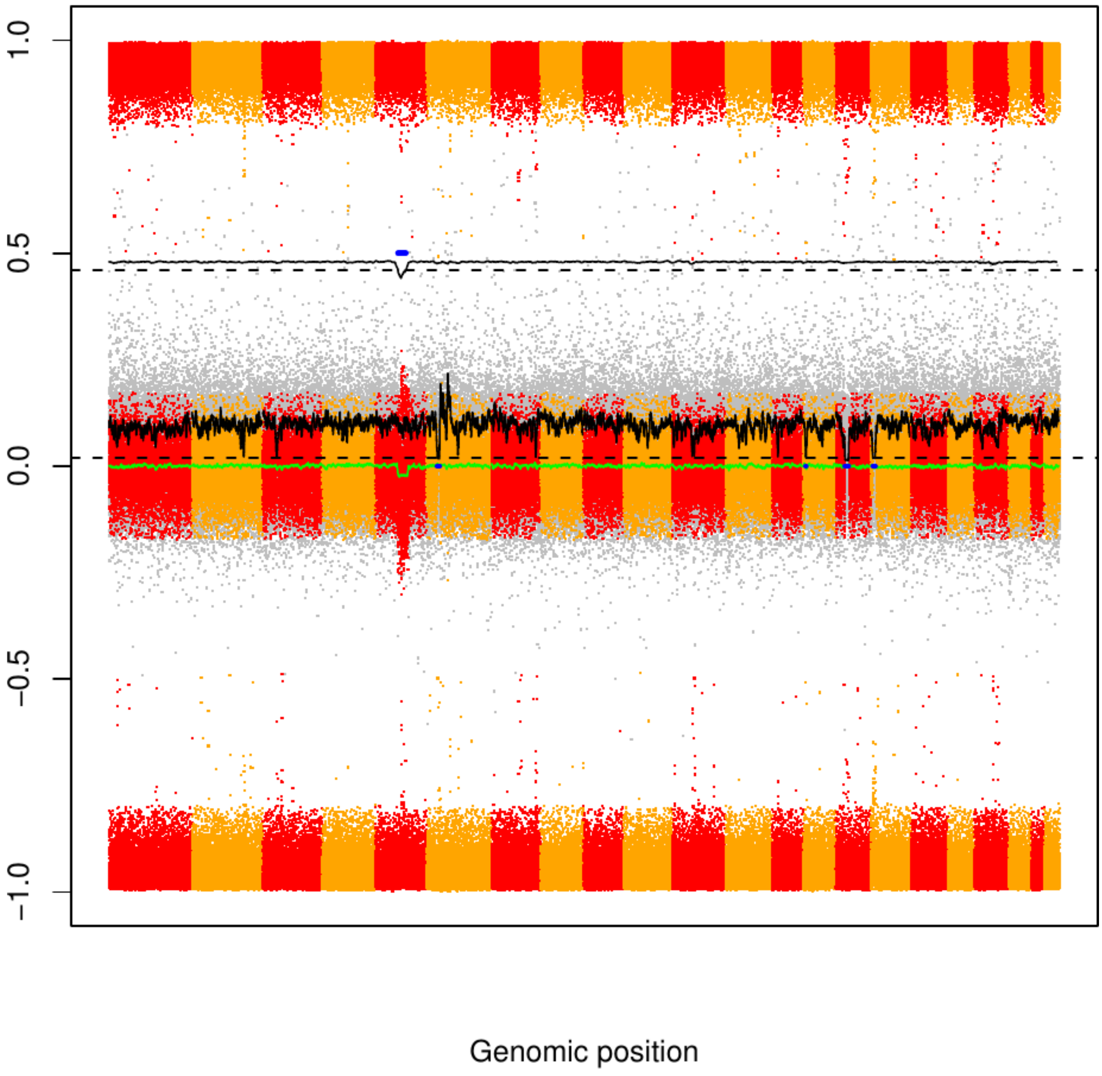

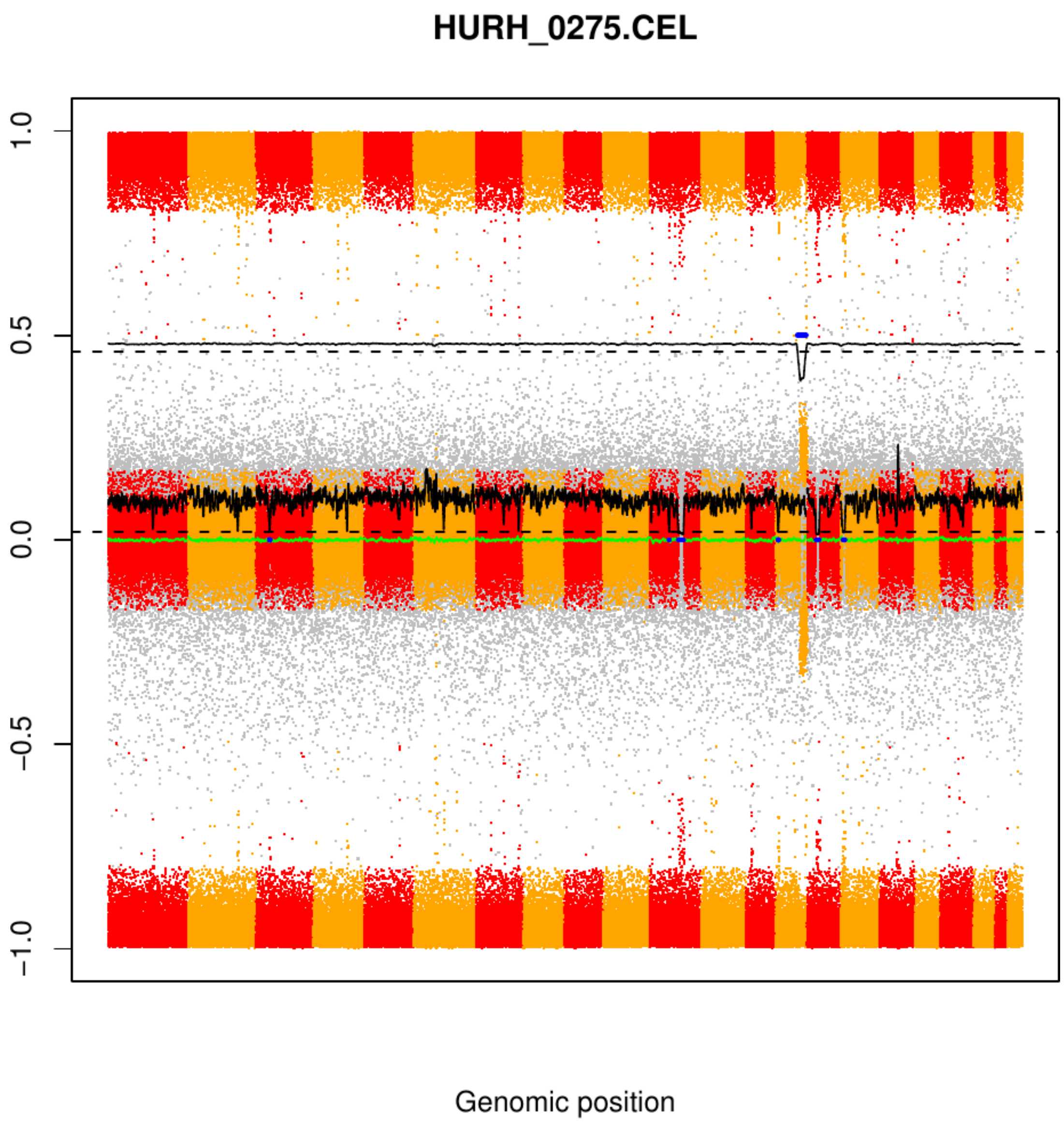
HURH_0275.CEL
1.0
0.5
0.0
−0.5
−1.0
Genomic position

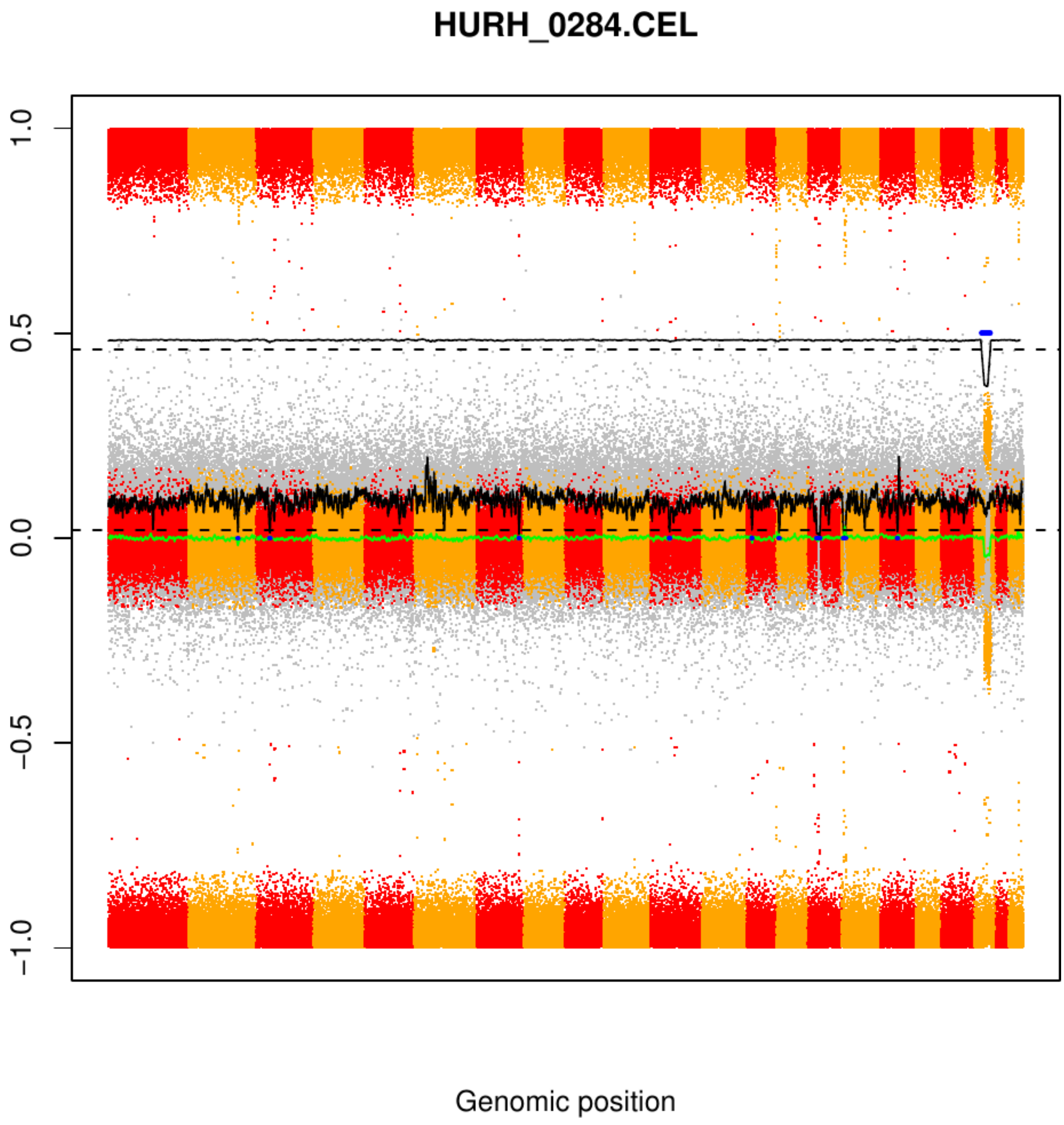

HURH_0284.CEL
1.0
0.5
0.0
−0.5
−1.0
Genomic position

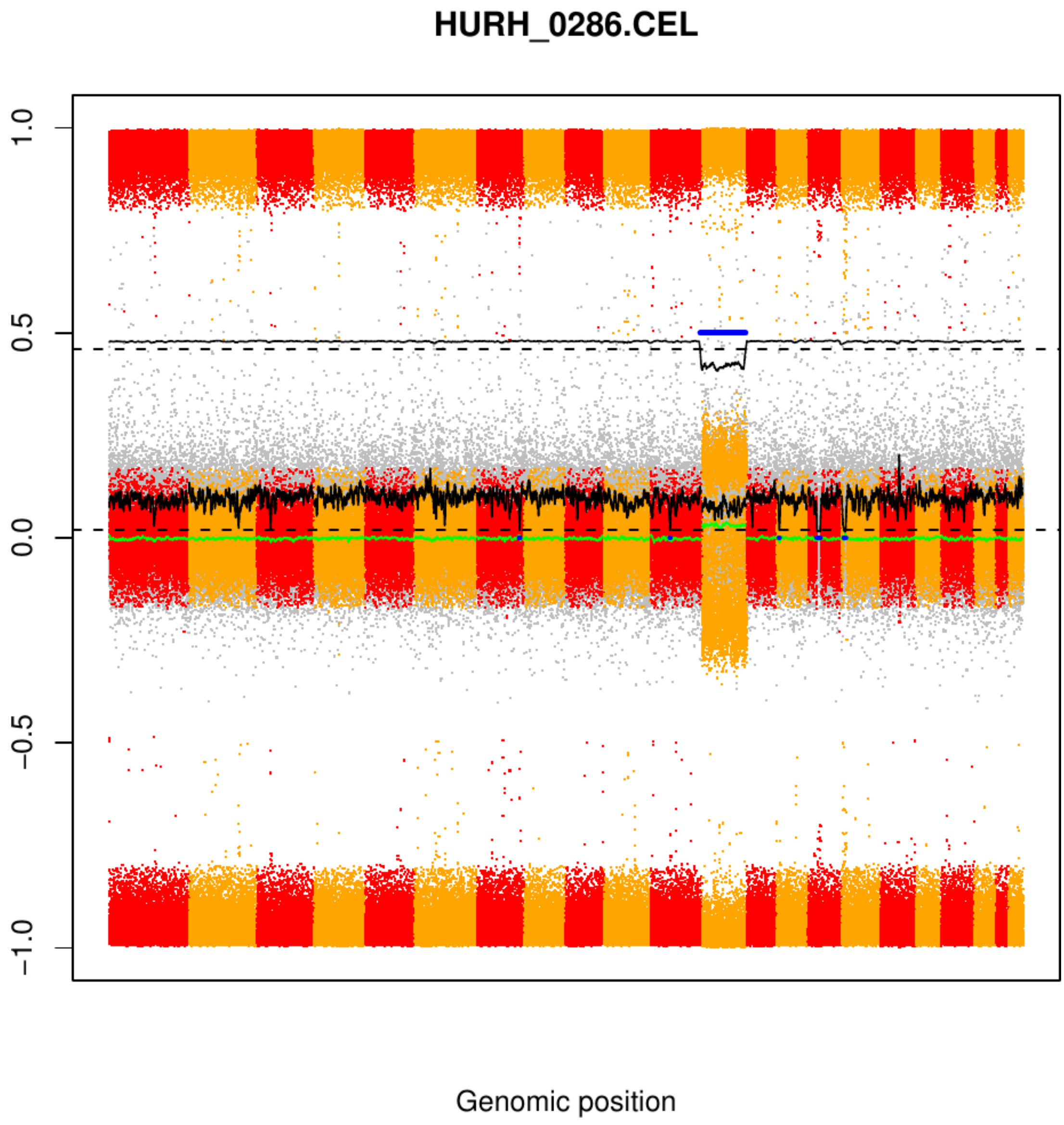
HURH_0286.CEL
1.0
0.5
0.0
−0.5
−1.0
Genomic position

HURH_0346.CEL

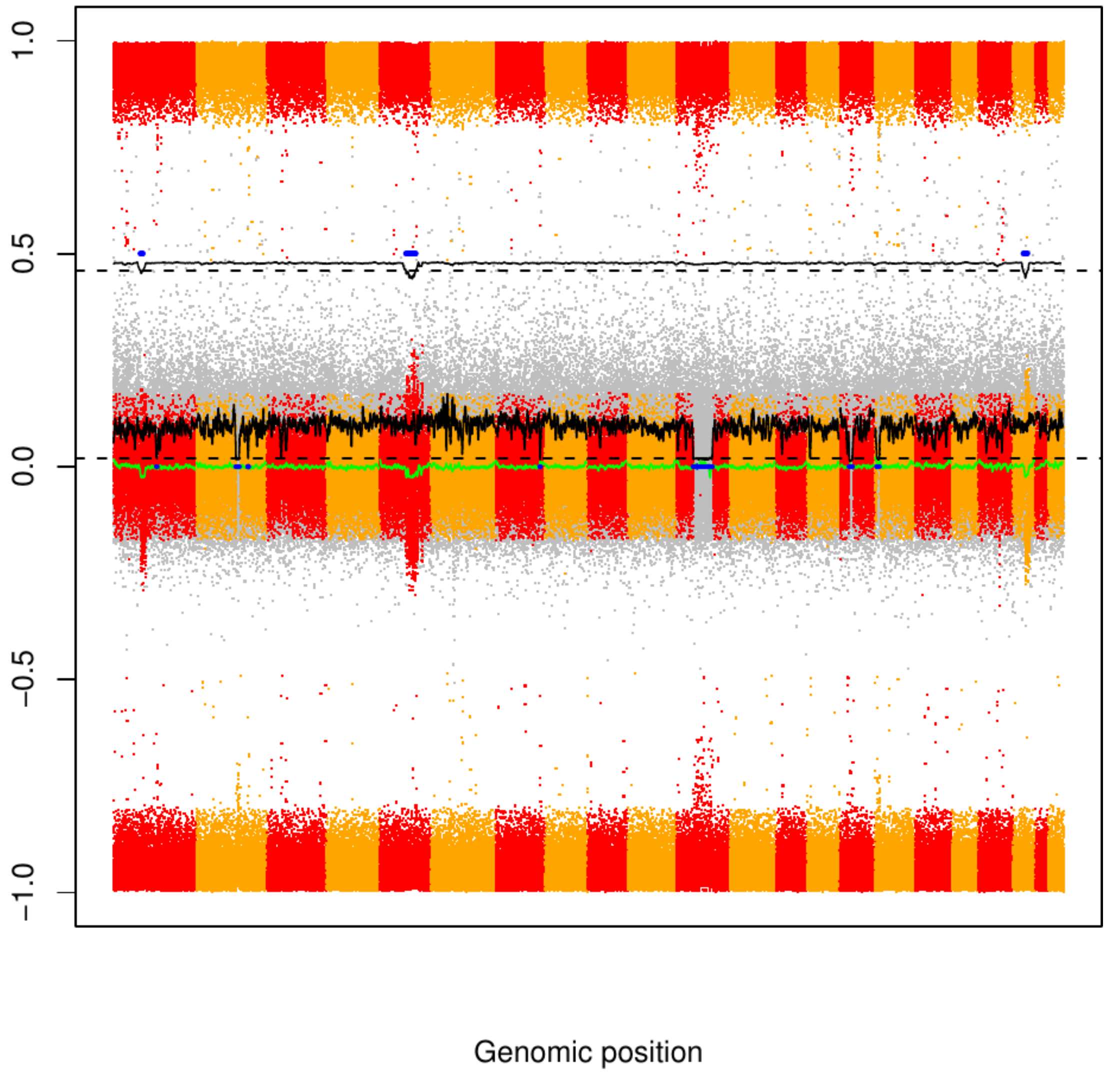

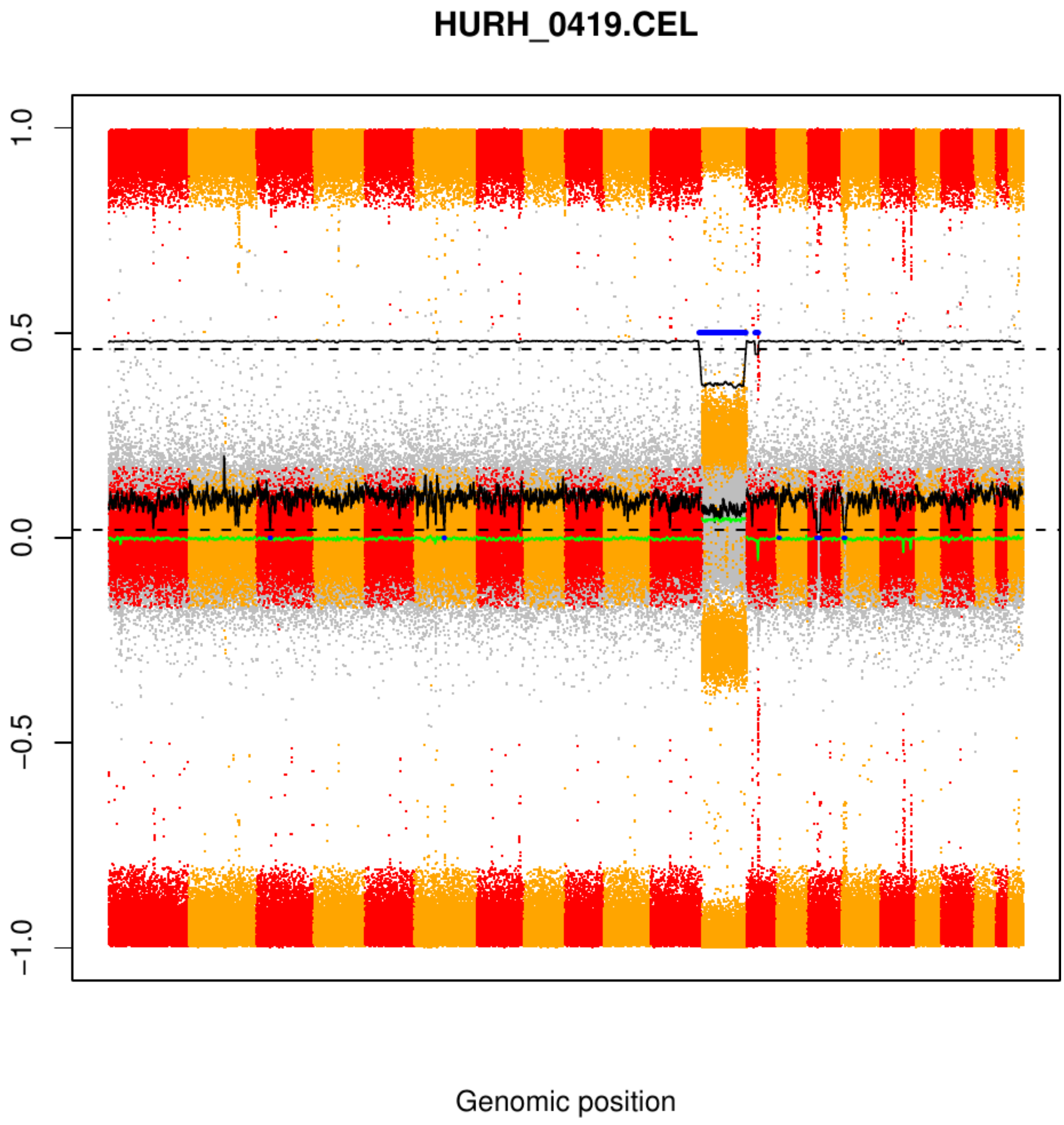
HURH_0419.CEL
1.0
0.5
0.0
−0.5
−1.0
Genomic position

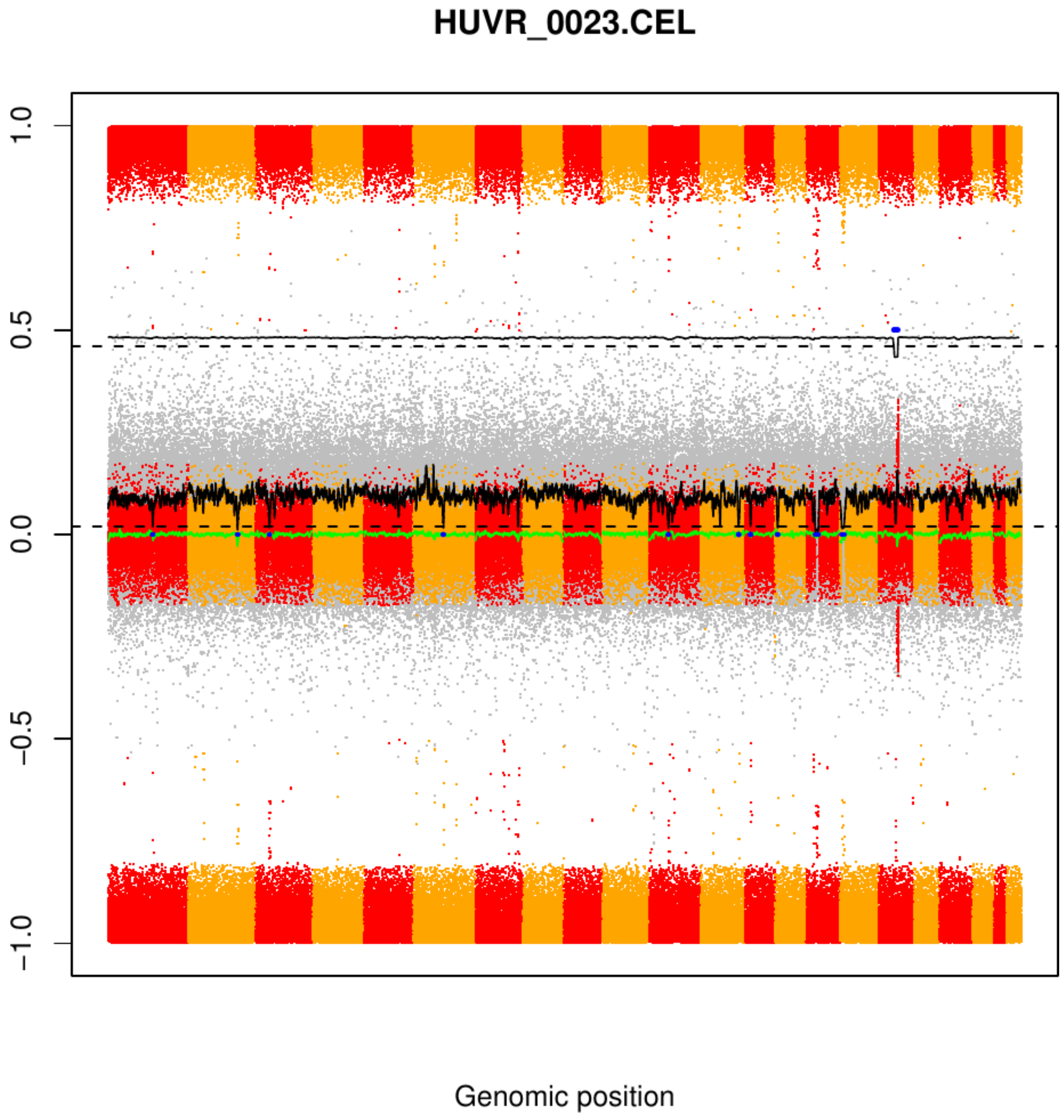
HUVR_0023.CEL
1.0
0.5
0.0
−0.5
−1.0
Genomic position

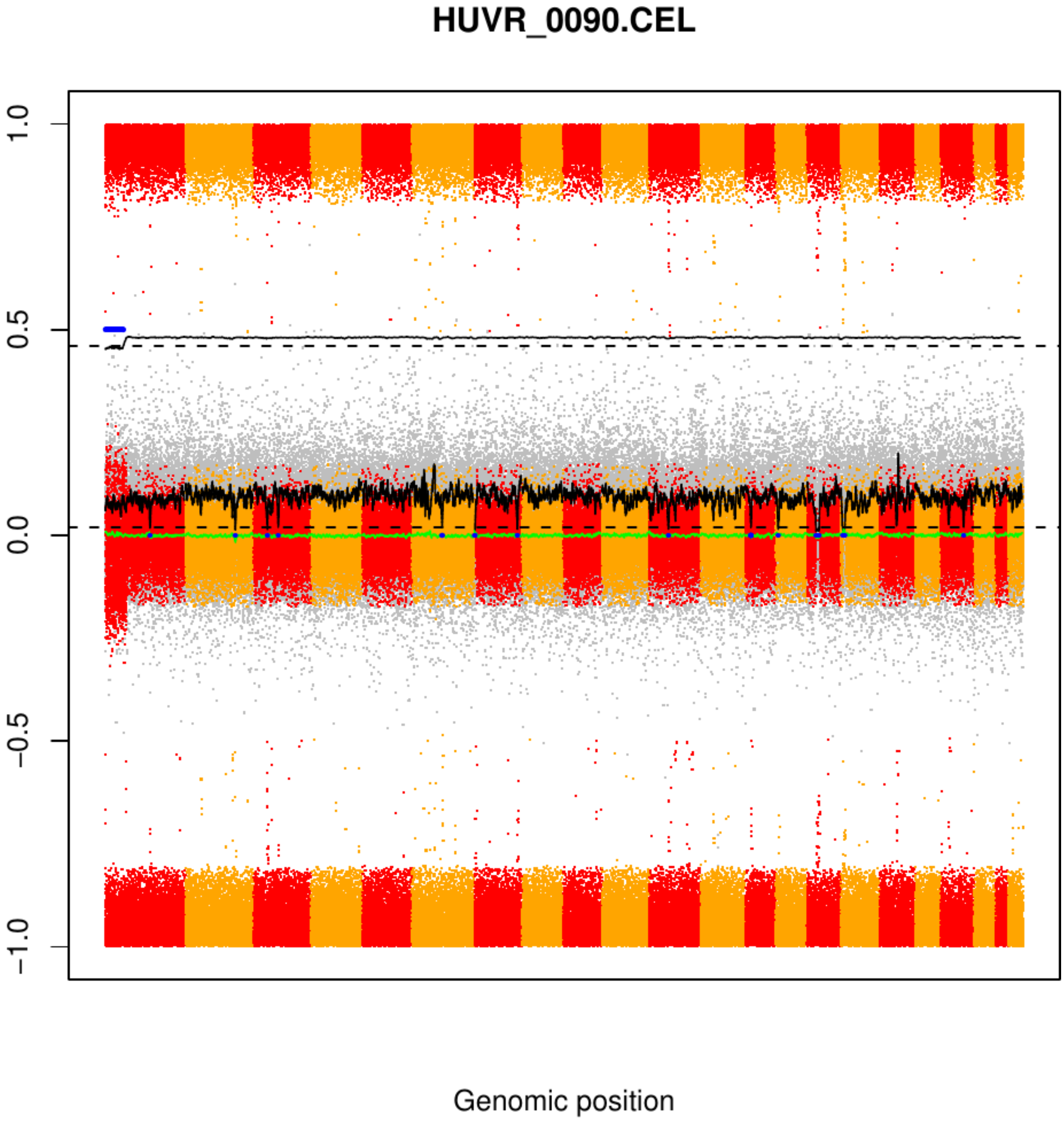
HUVR_0090.CEL
1.0
0.5
0.0
−0.5
−1.0
Genomic position

HUVR_0149.CEL

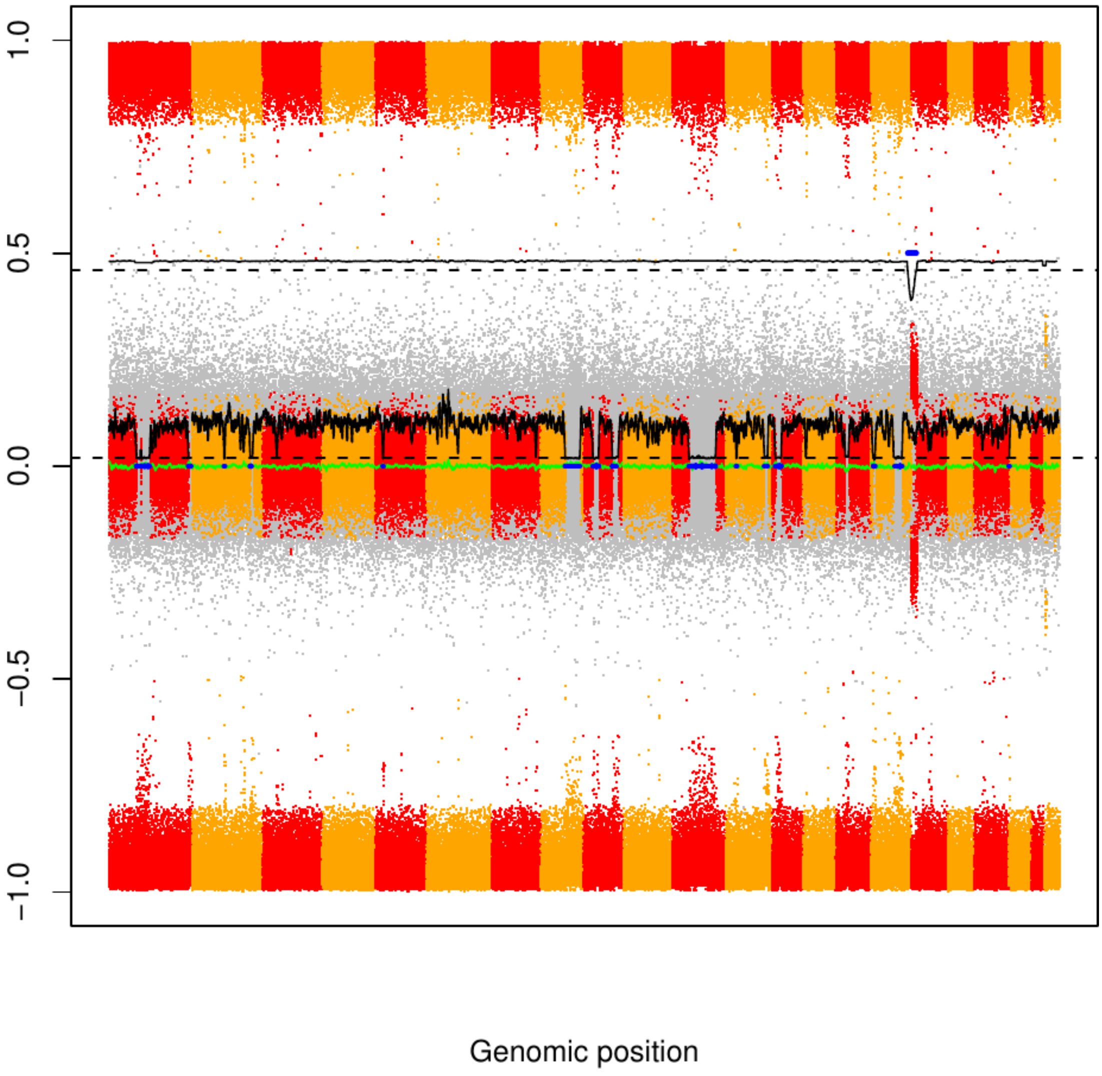

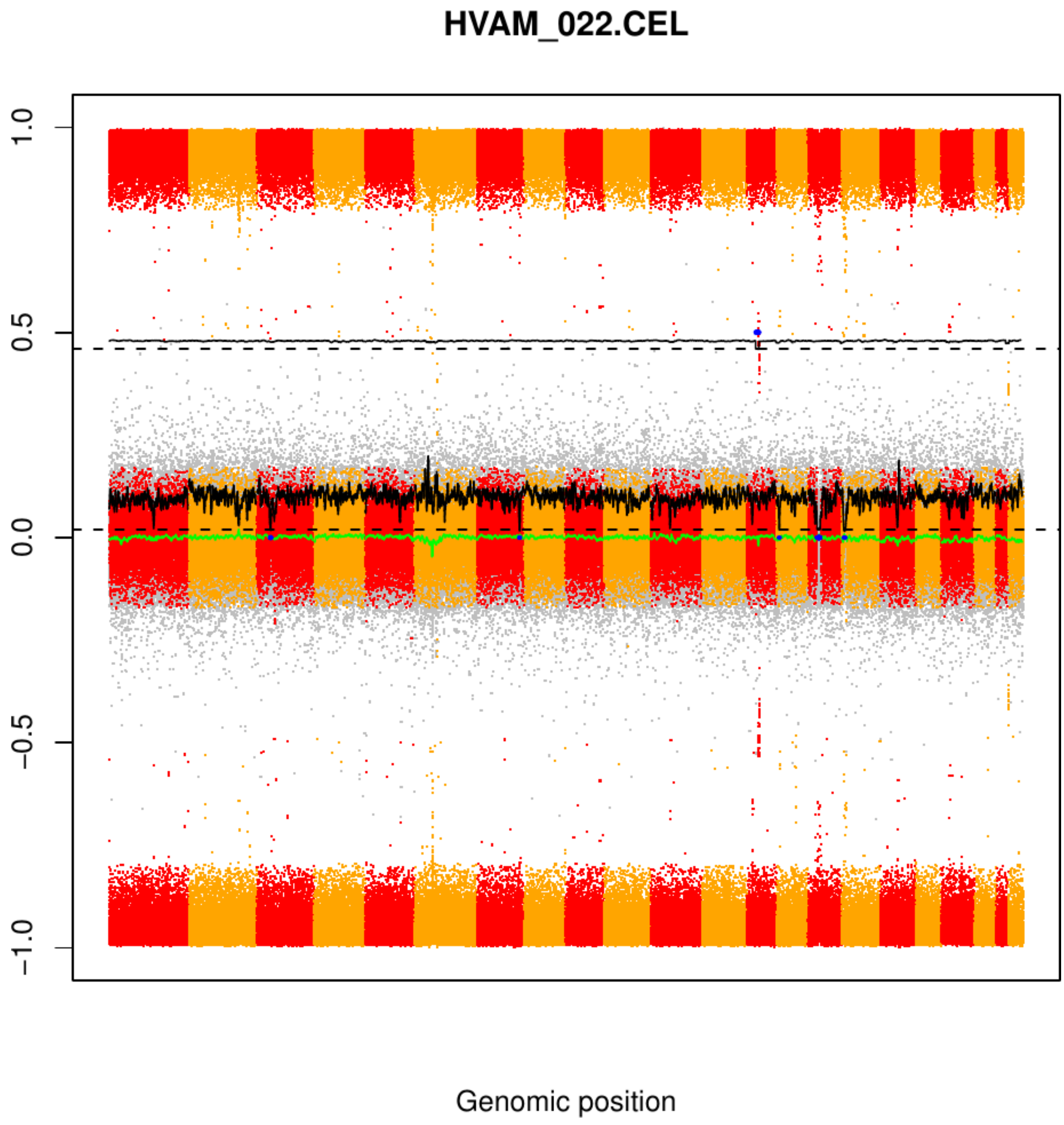
HVAM_022.CEL
1.0
0.5
0.0
−0.5
−1.0
Genomic position

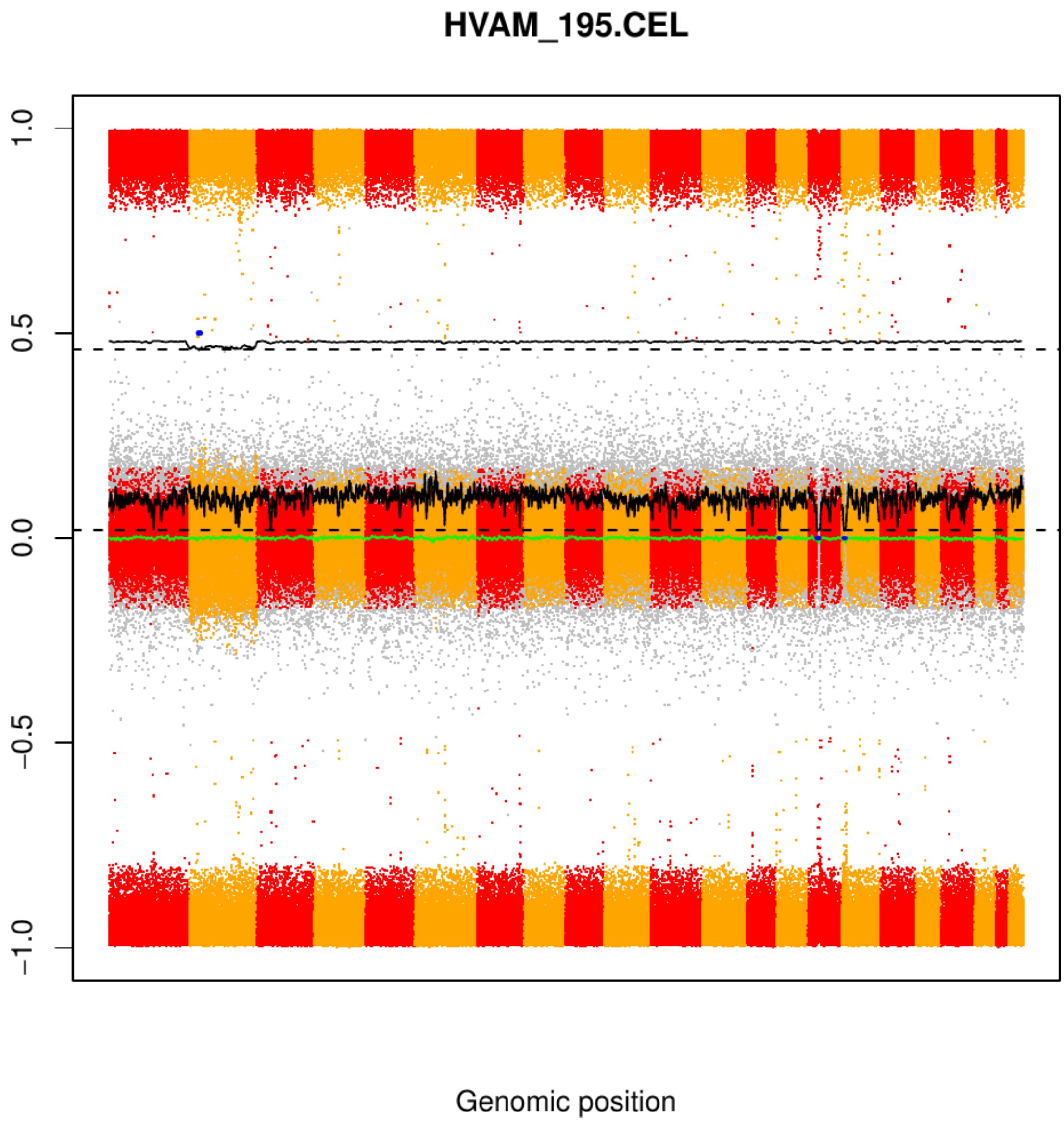
HVAM_195.CEL
1.0
0.5
0.0
−0.5
−1.0
Genomic position

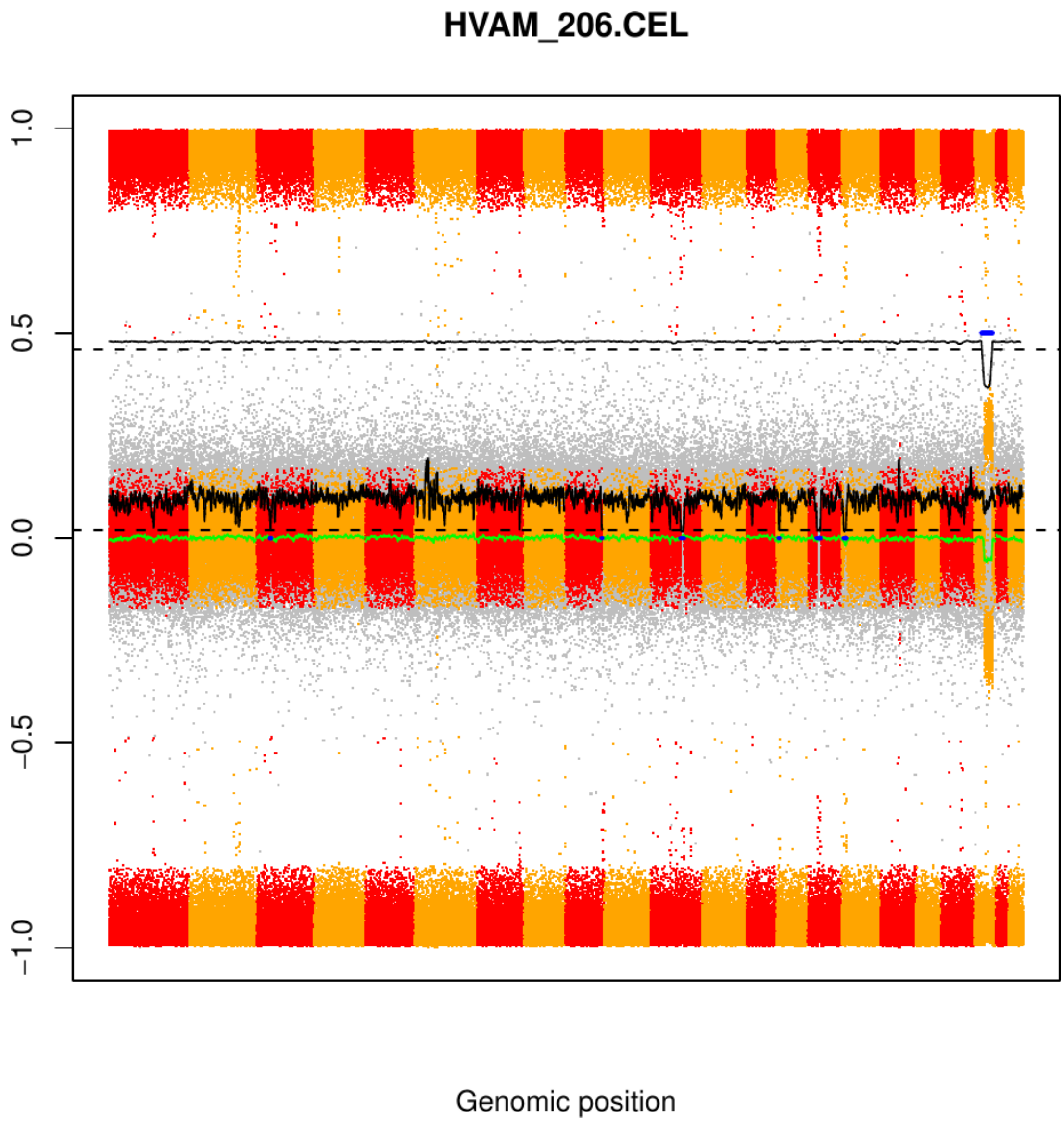
HVAM_206.CEL
1.0
0.5
0.0
−0.5
−1.0
Genomic position

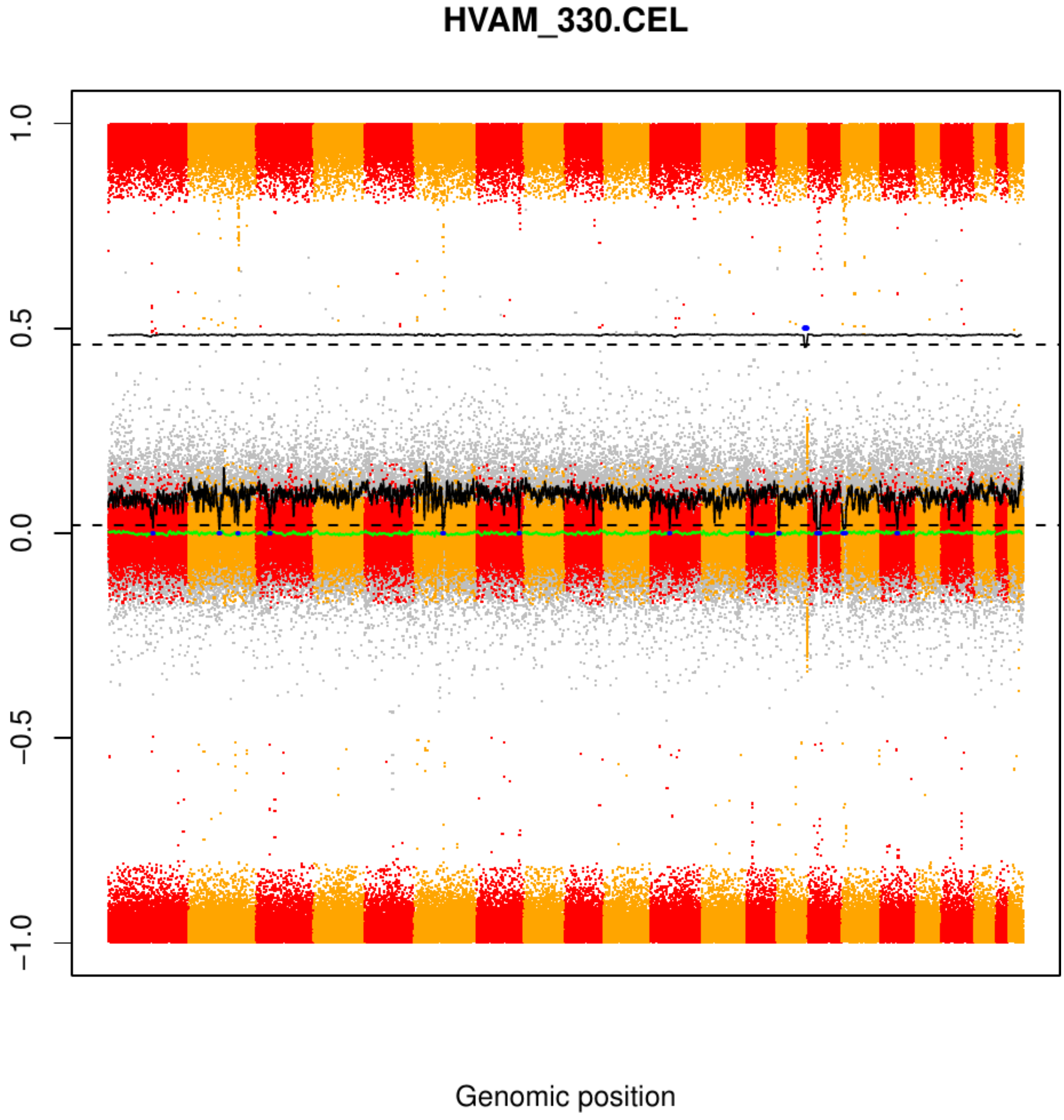

HVAM_330.CEL
1.0
0.5
0.0
−0.5
−1.0
Genomic position

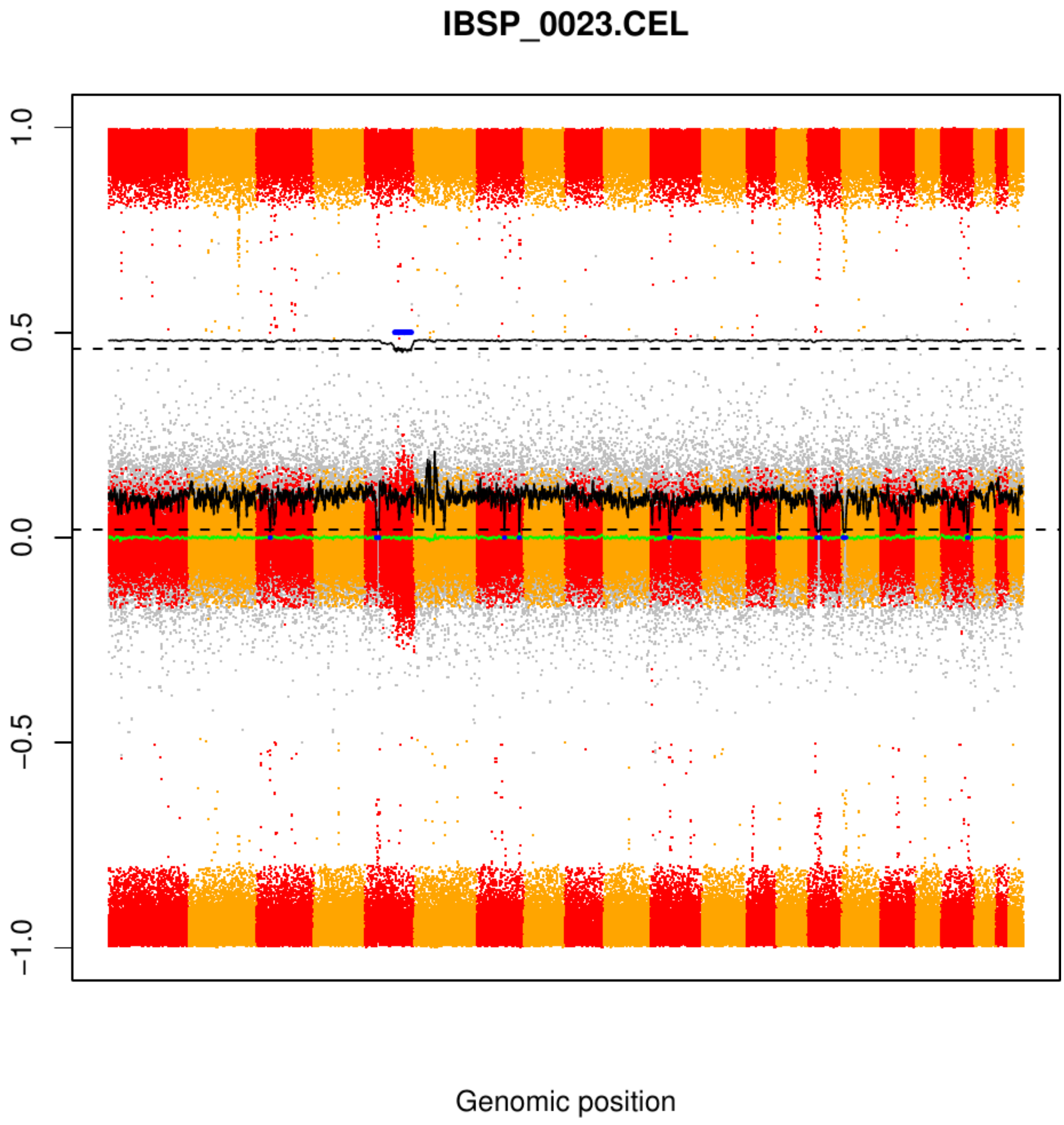
IBSP_0023.CEL
1.0
0.5
0.0
−0.5
−1.0
Genomic position

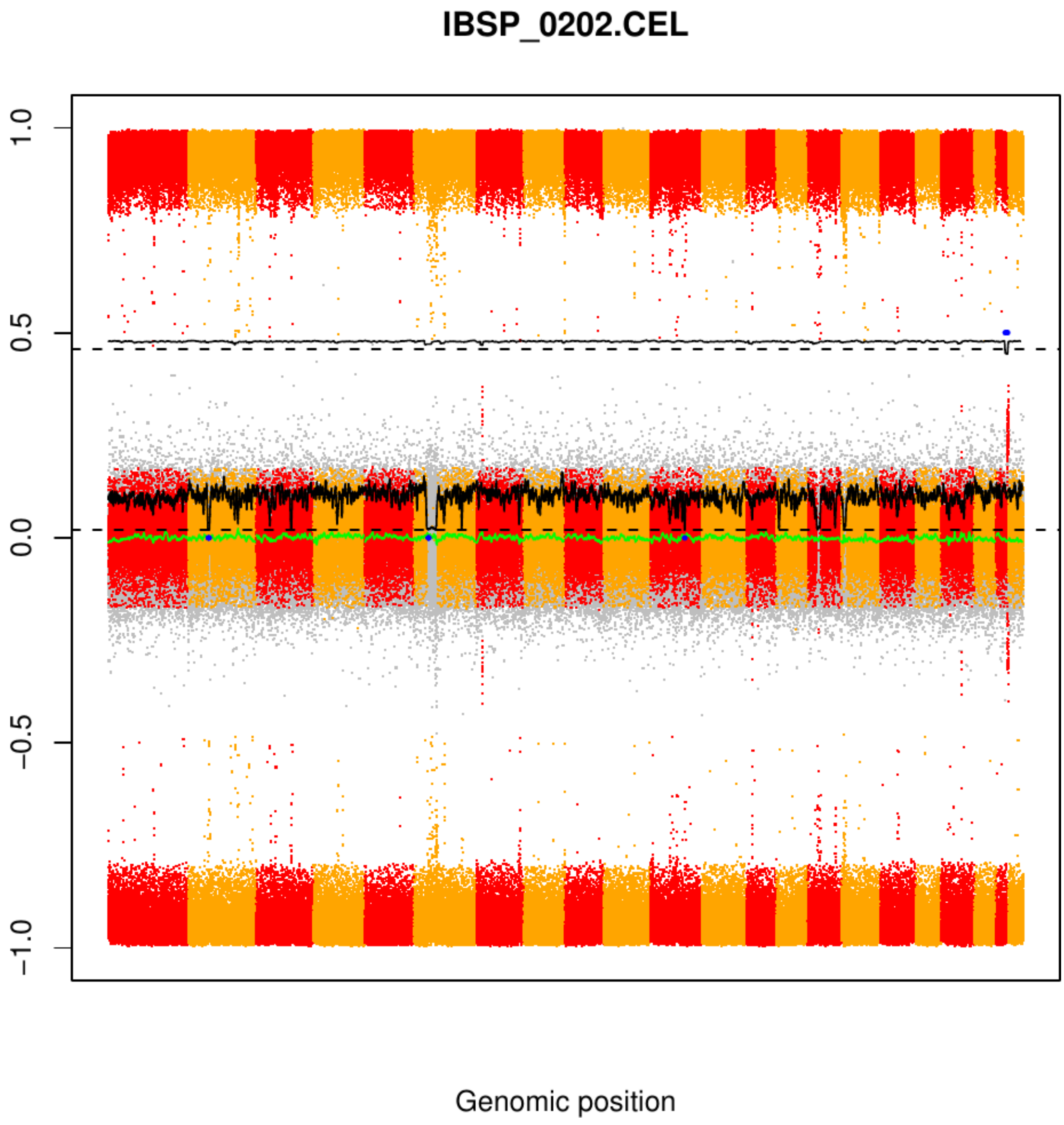
IBSP_0202.CEL
1.0
0.5
0.0
−0.5
−1.0
Genomic position

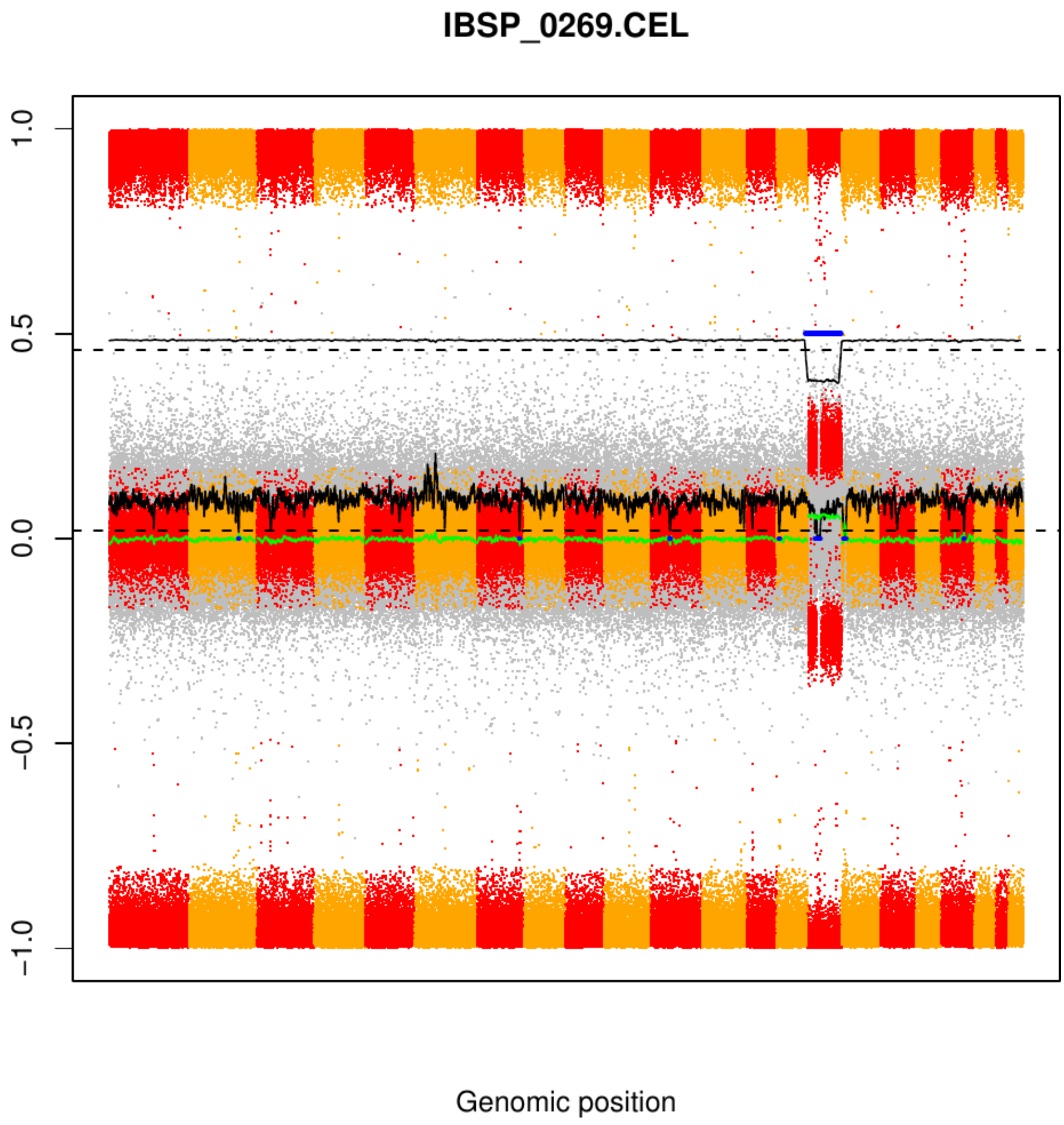
IBSP_0269.CEL
1.0
0.5
0.0
−0.5
−1.0
Genomic position

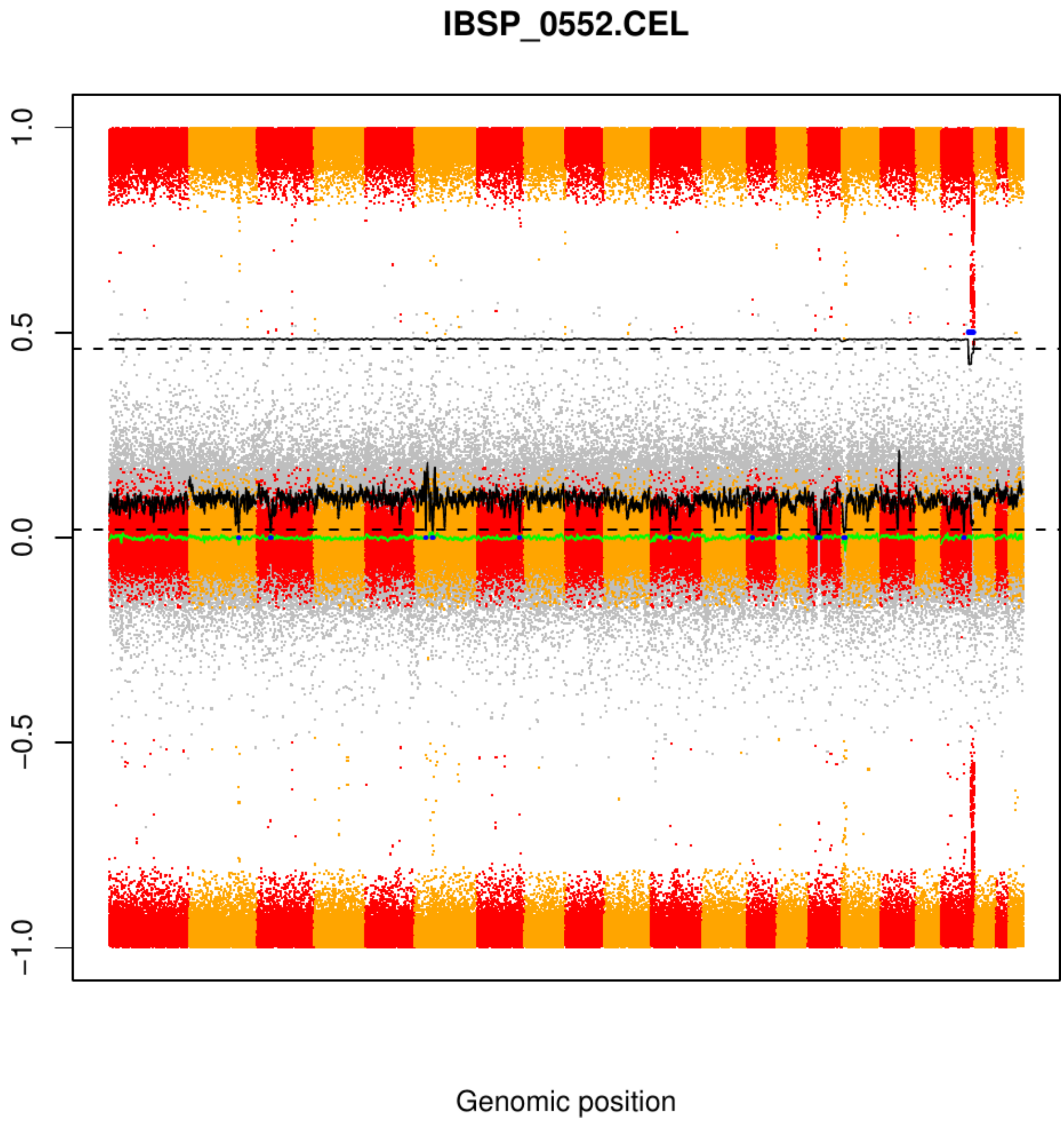

IBSP_0552.CEL
1.0
0.5
0.0
−0.5
−1.0
Genomic position

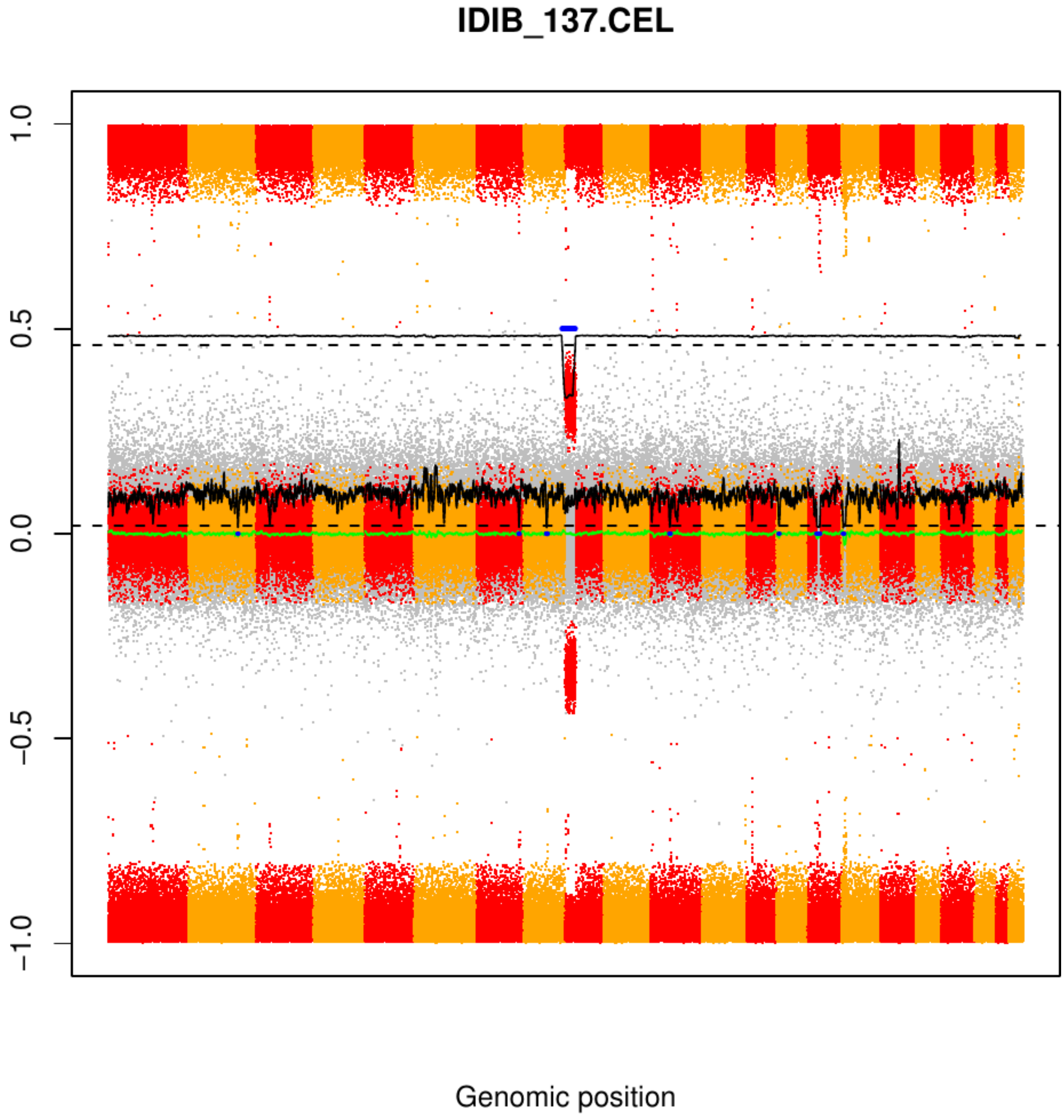
IDIB_137.CEL
1.0
0.5
0.0
−0.5
−1.0
Genomic position

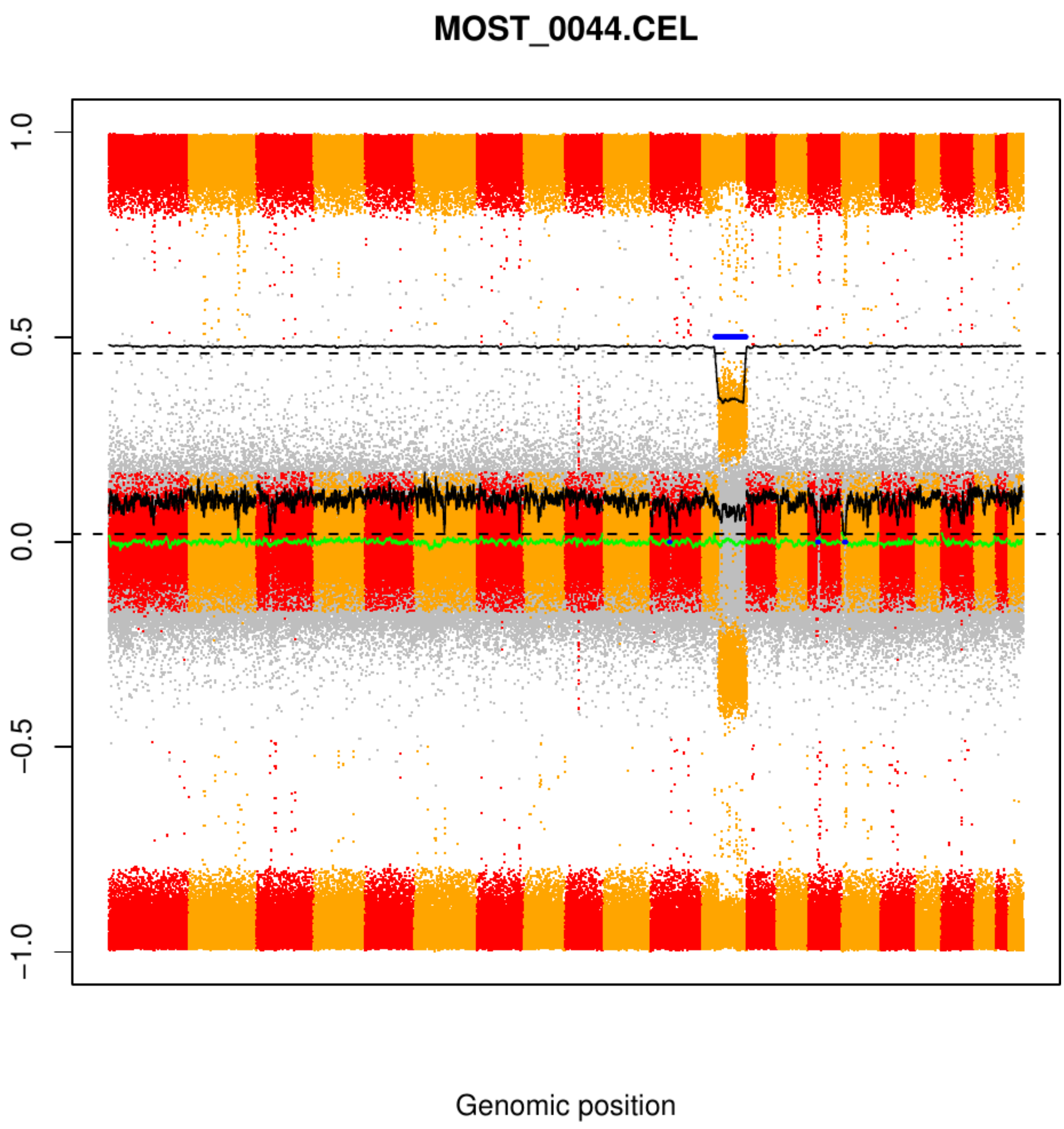
MOST_0044.CEL
1.0
0.5
0.0
−0.5
−1.0
Genomic position

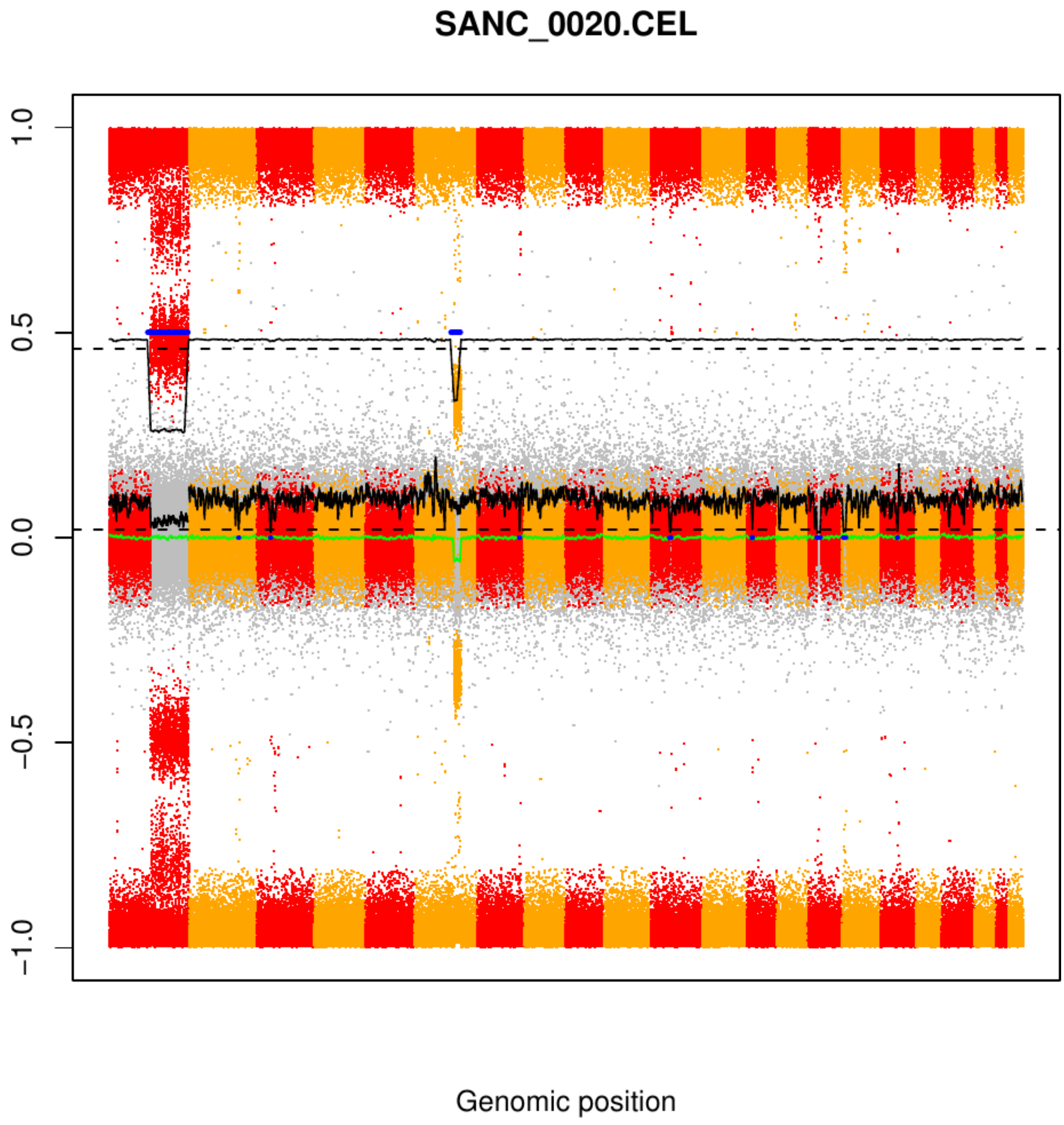
SANC_0020.CEL
1.0
0.5
0.0
−0.5
−1.0
Genomic position

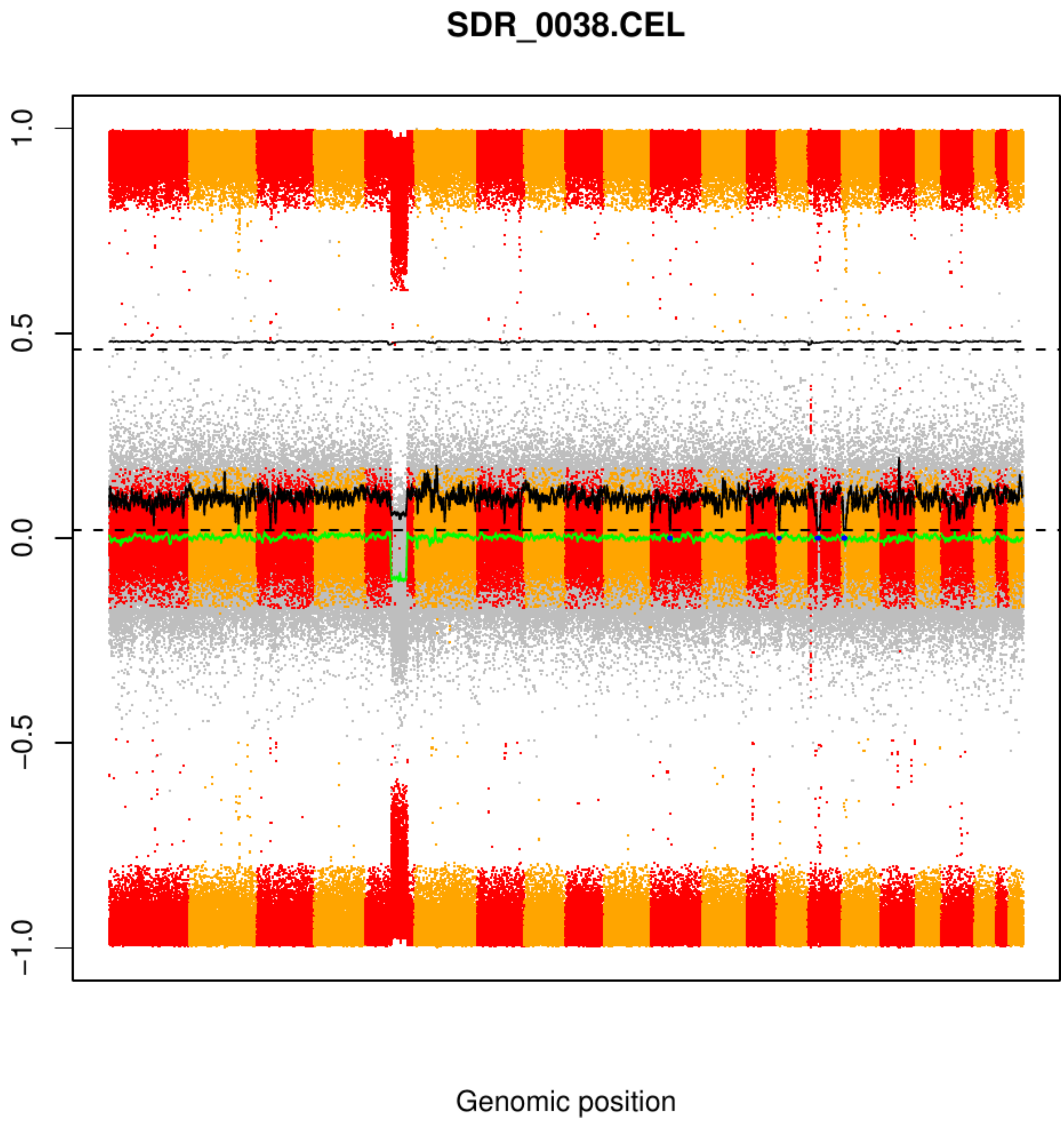
SDR_0038.CEL
1.0
0.5
0.0
−0.5
−1.0
Genomic position

SDR_0159.CEL

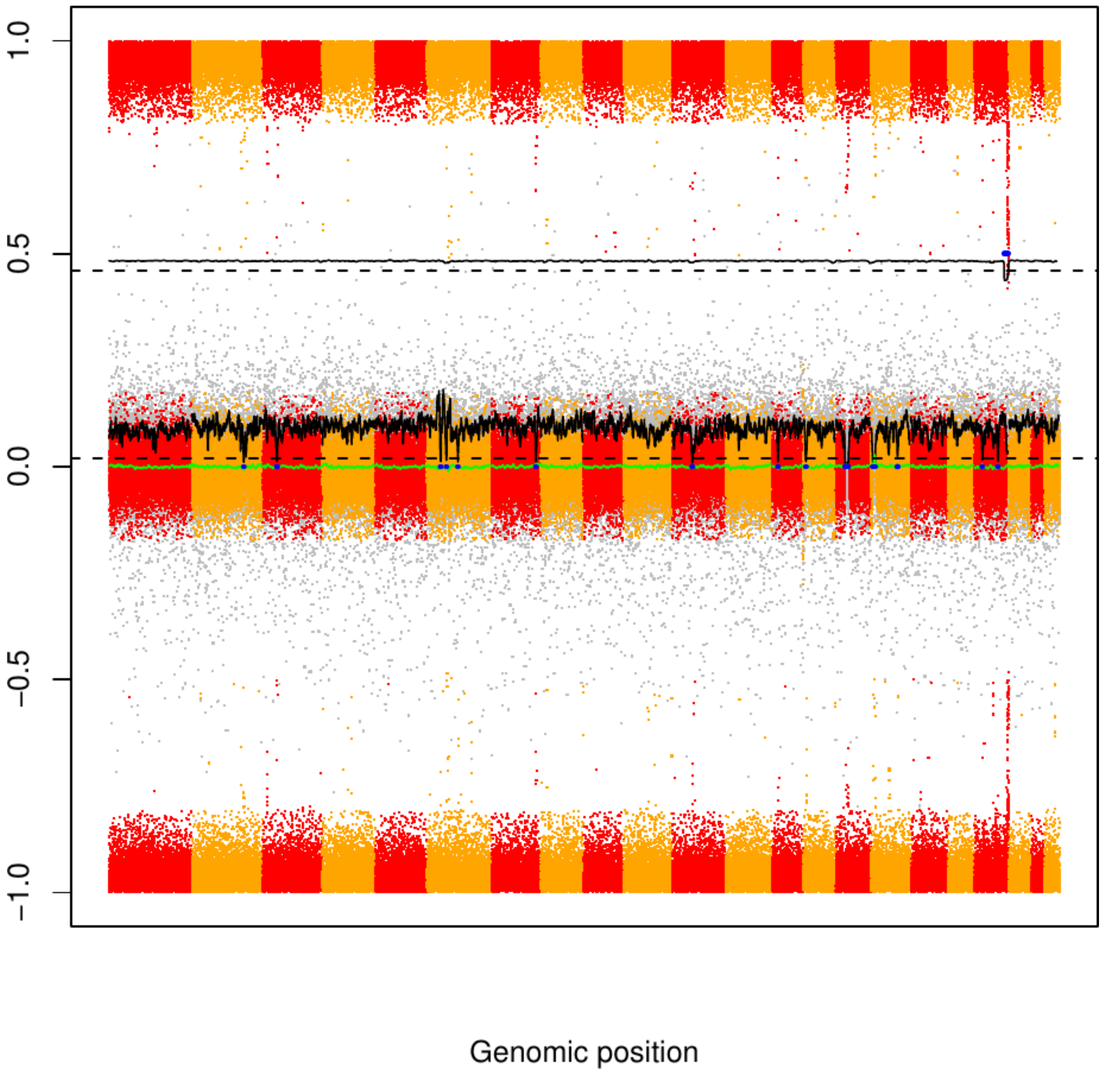

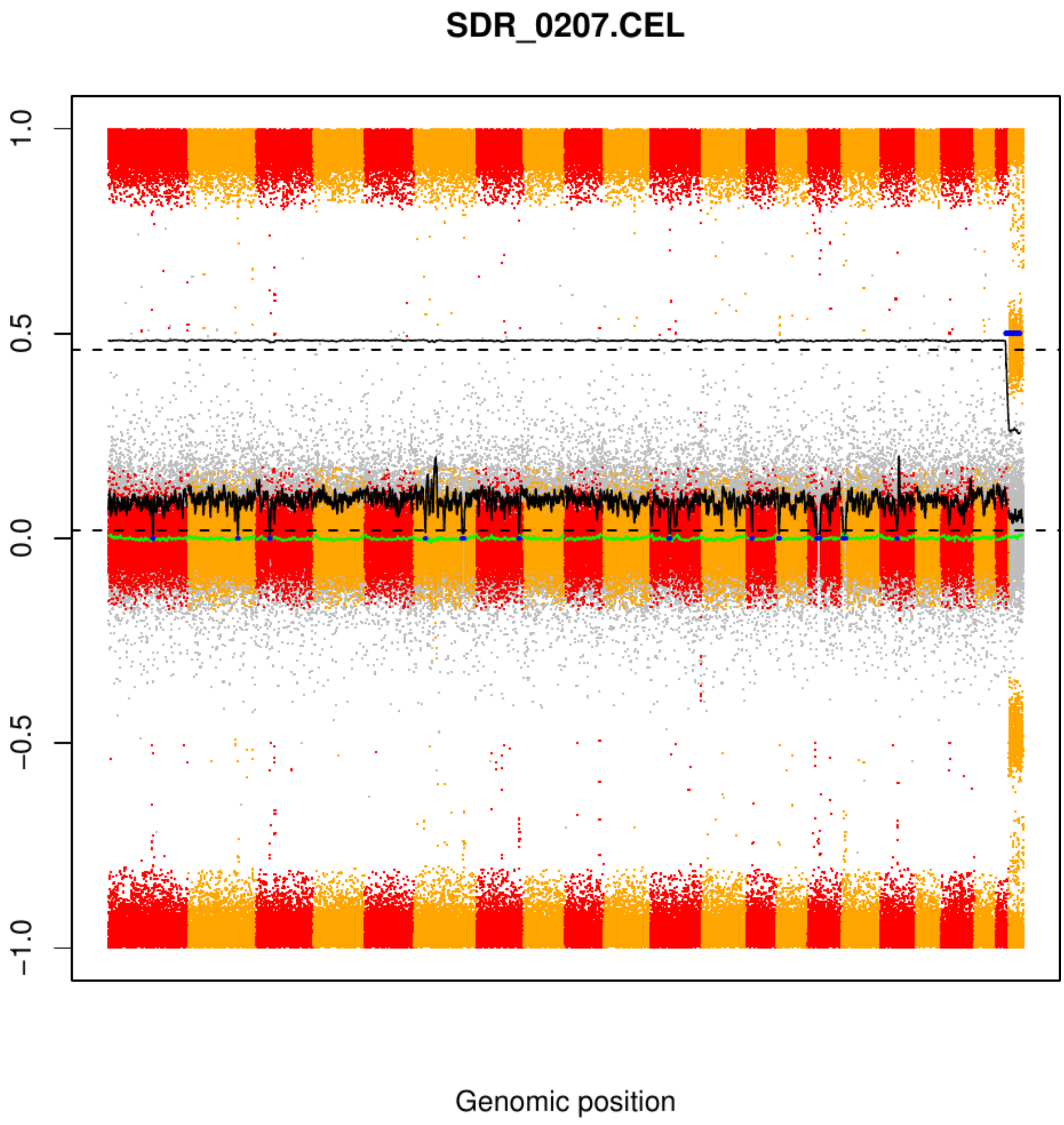
SDR_0207.CEL
1.0
0.5
0.0
−0.5
−1.0
Genomic position

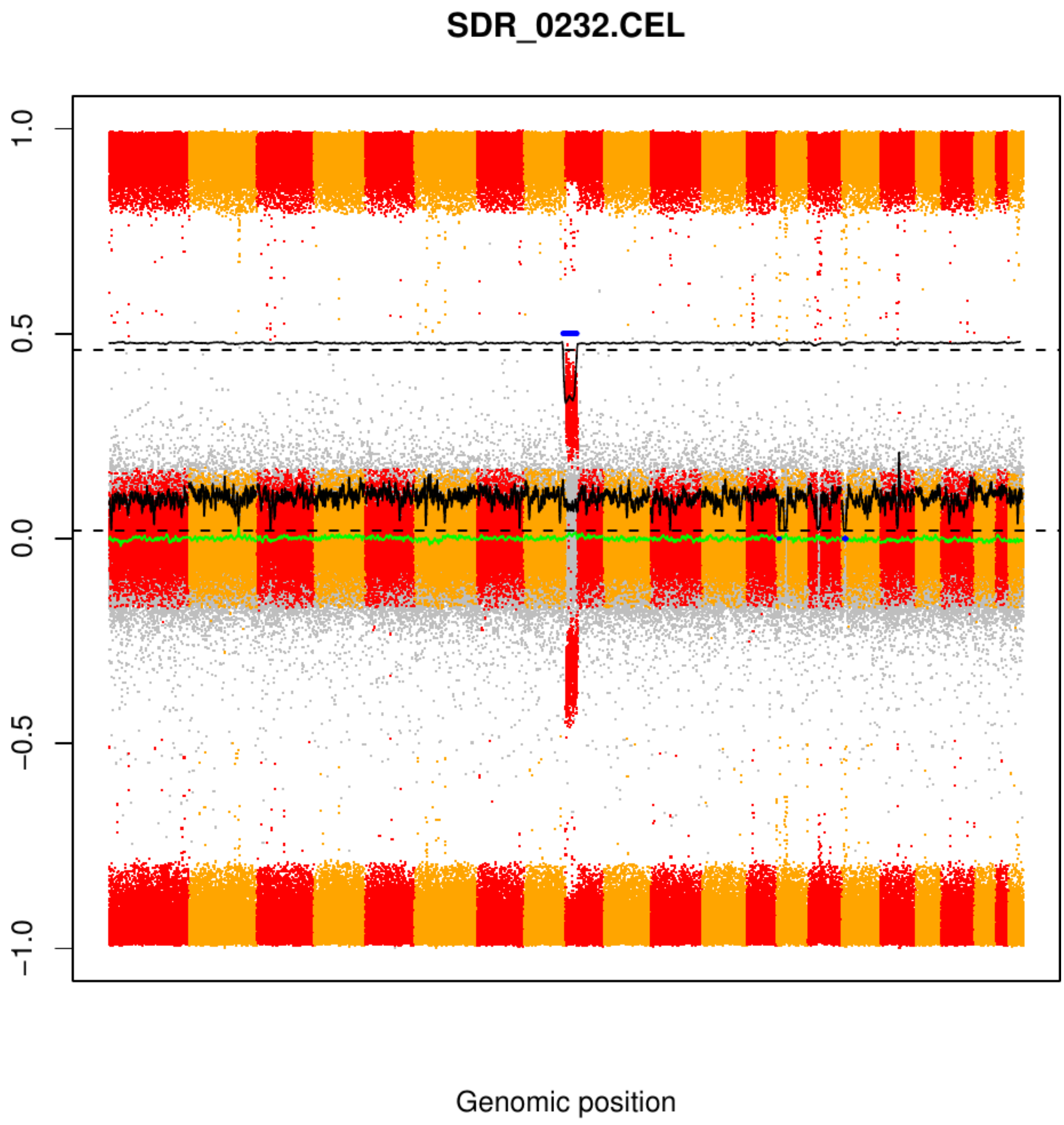
SDR_0232.CEL
1.0
0.5
0.0
−0.5
−1.0
Genomic position

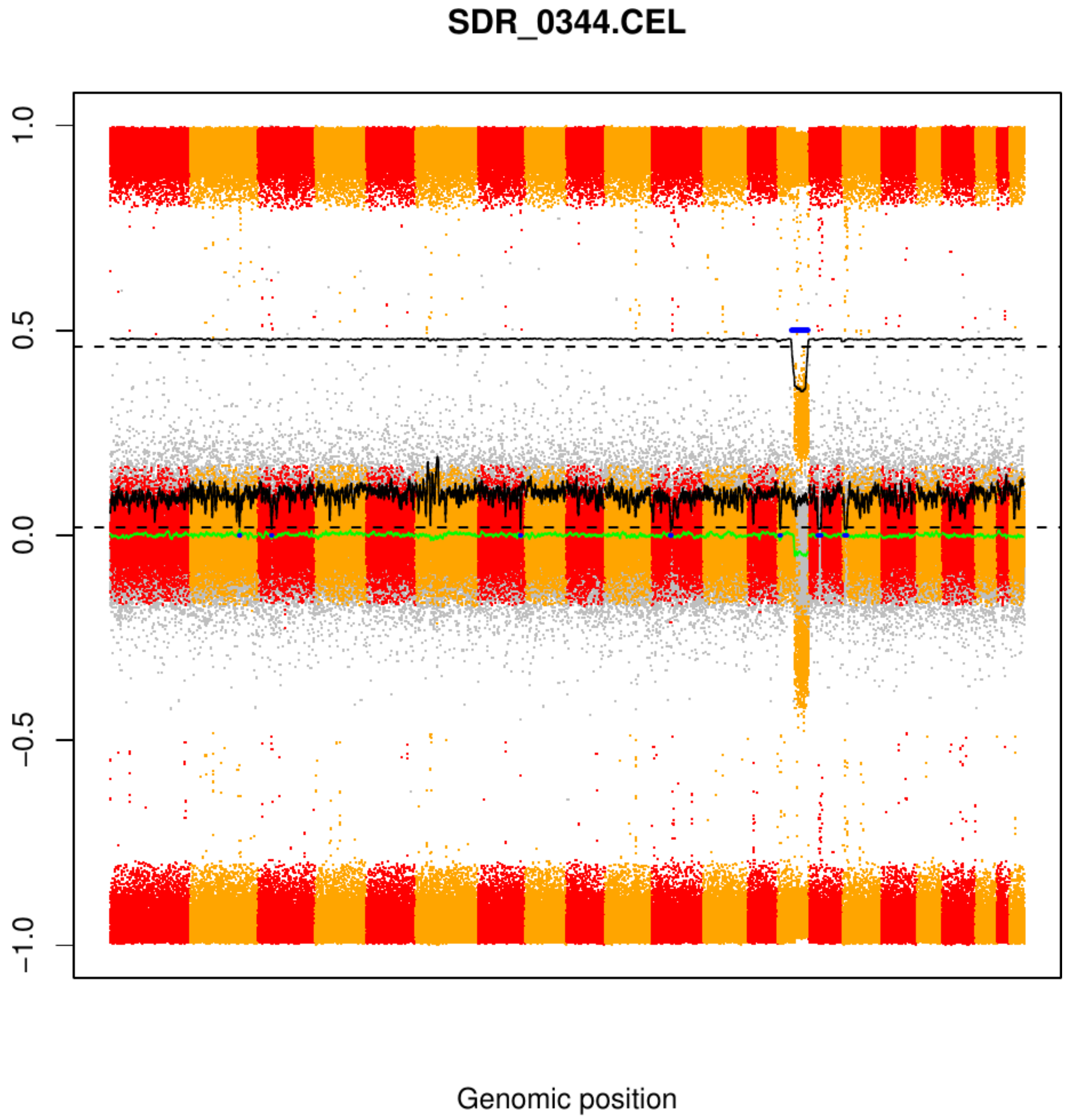
SDR_0344.CEL
1.0
0.5
0.0
−0.5
−1.0
Genomic position

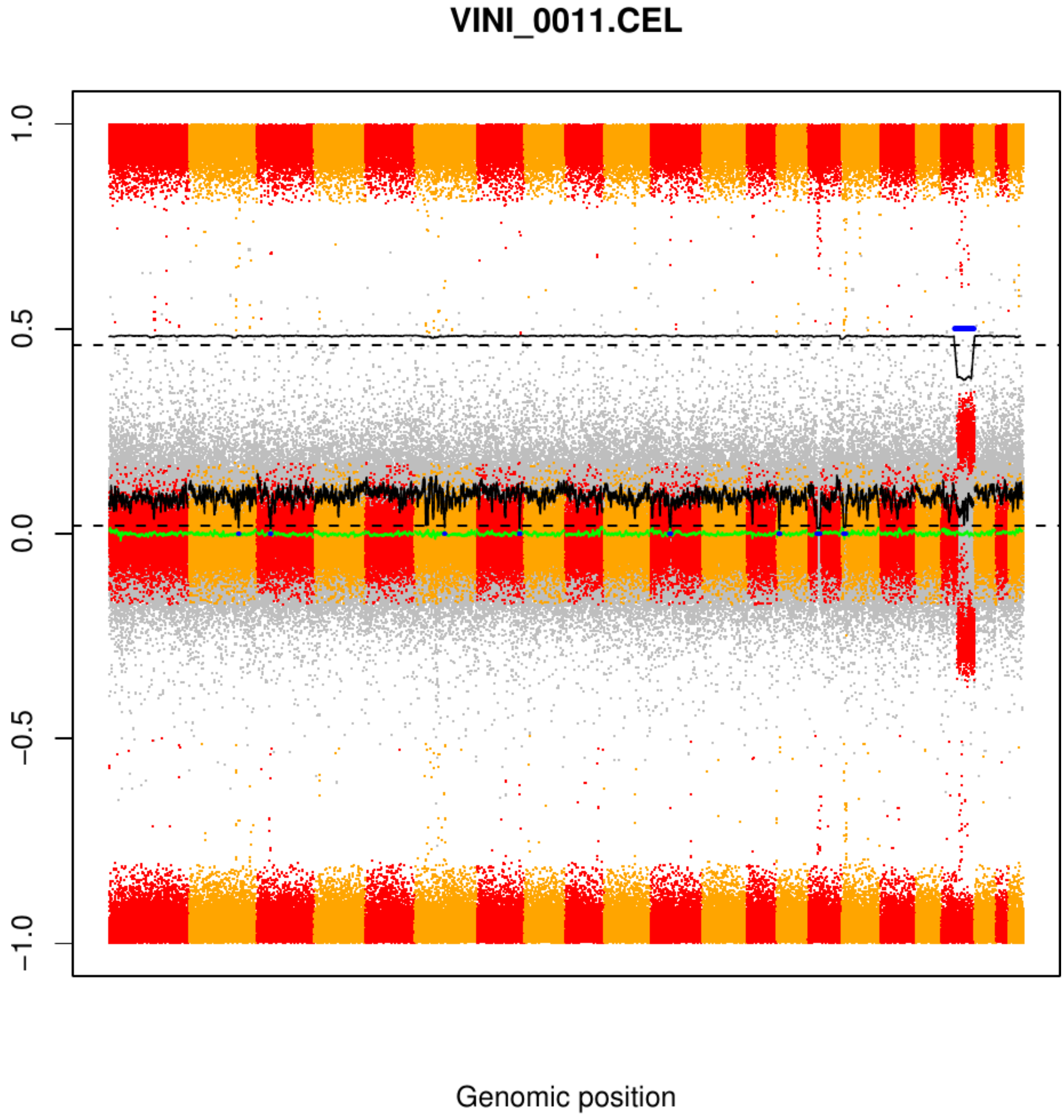
VINI_0011.CEL
1.0
0.5
0.0
−0.5
−1.0
Genomic position

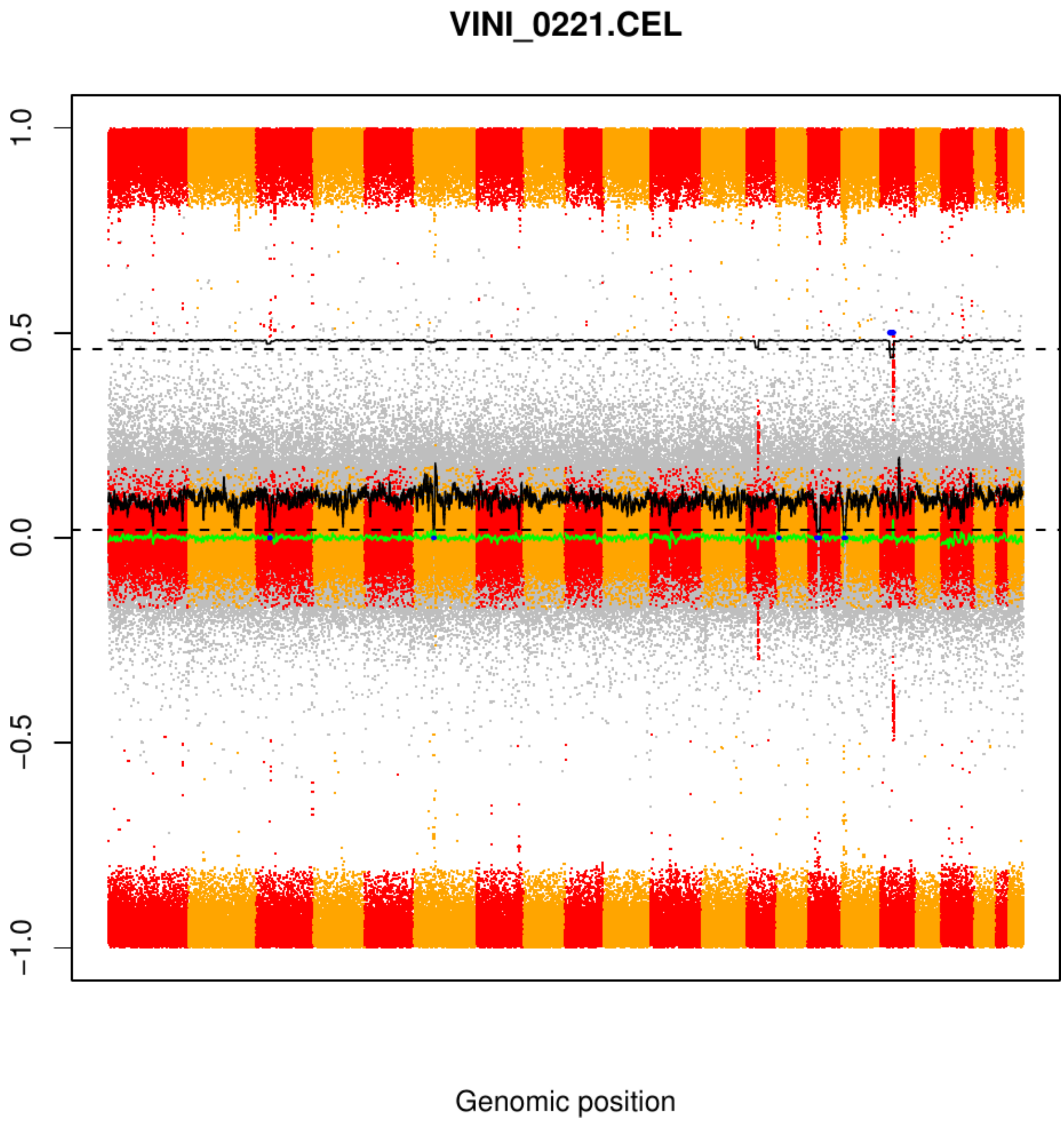

VINI_0221.CEL
1.0
0.5
0.0
−0.5
−1.0
Genomic position

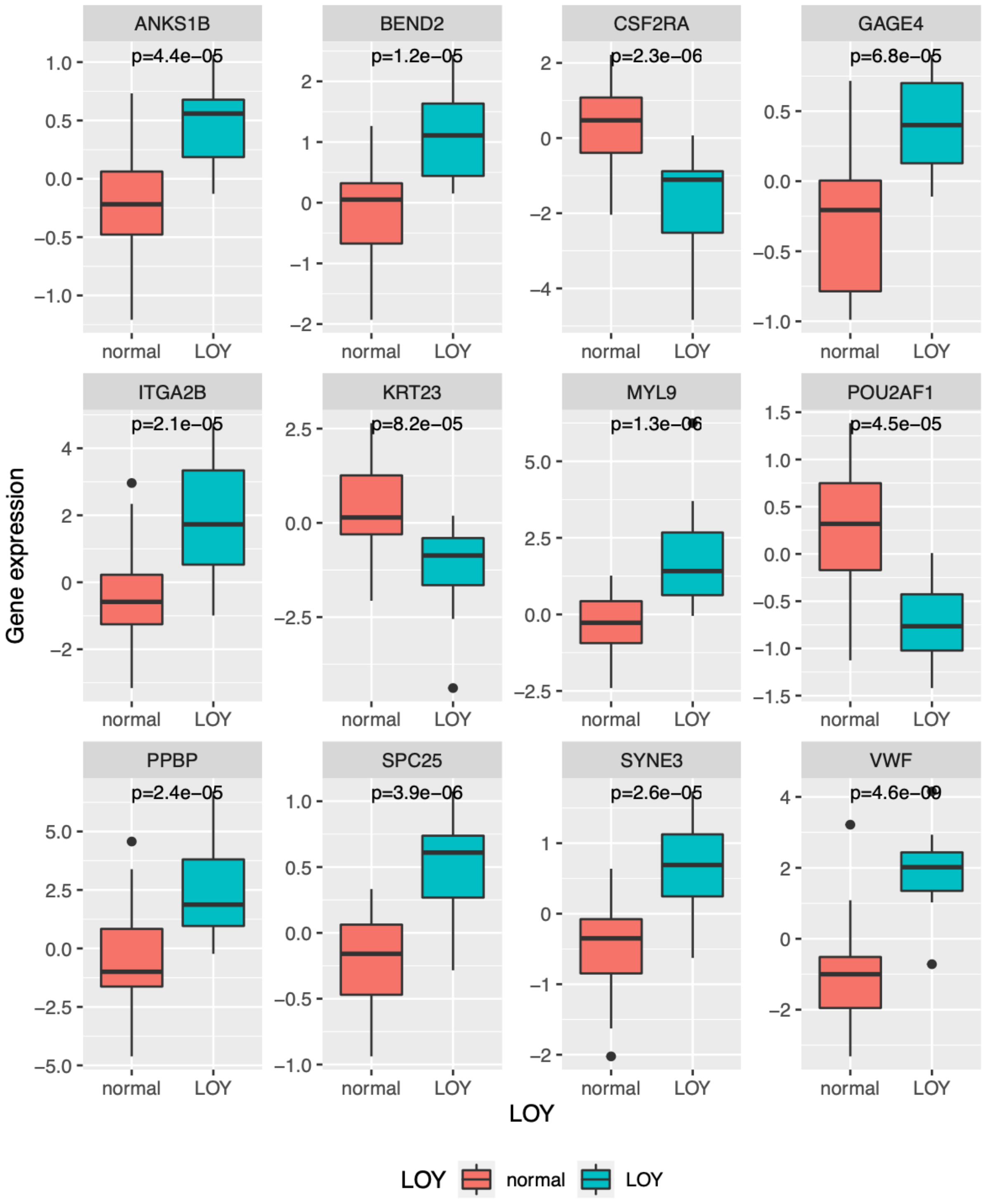

Figure S2: Top differentially expressed genes in blood between individual with LOY and controls at genome level. The plots show the gene expression for individuals with (LOY) and without LOY (normal). The p-values correspond to a linear model adjusted for age and surrogate variables using limma

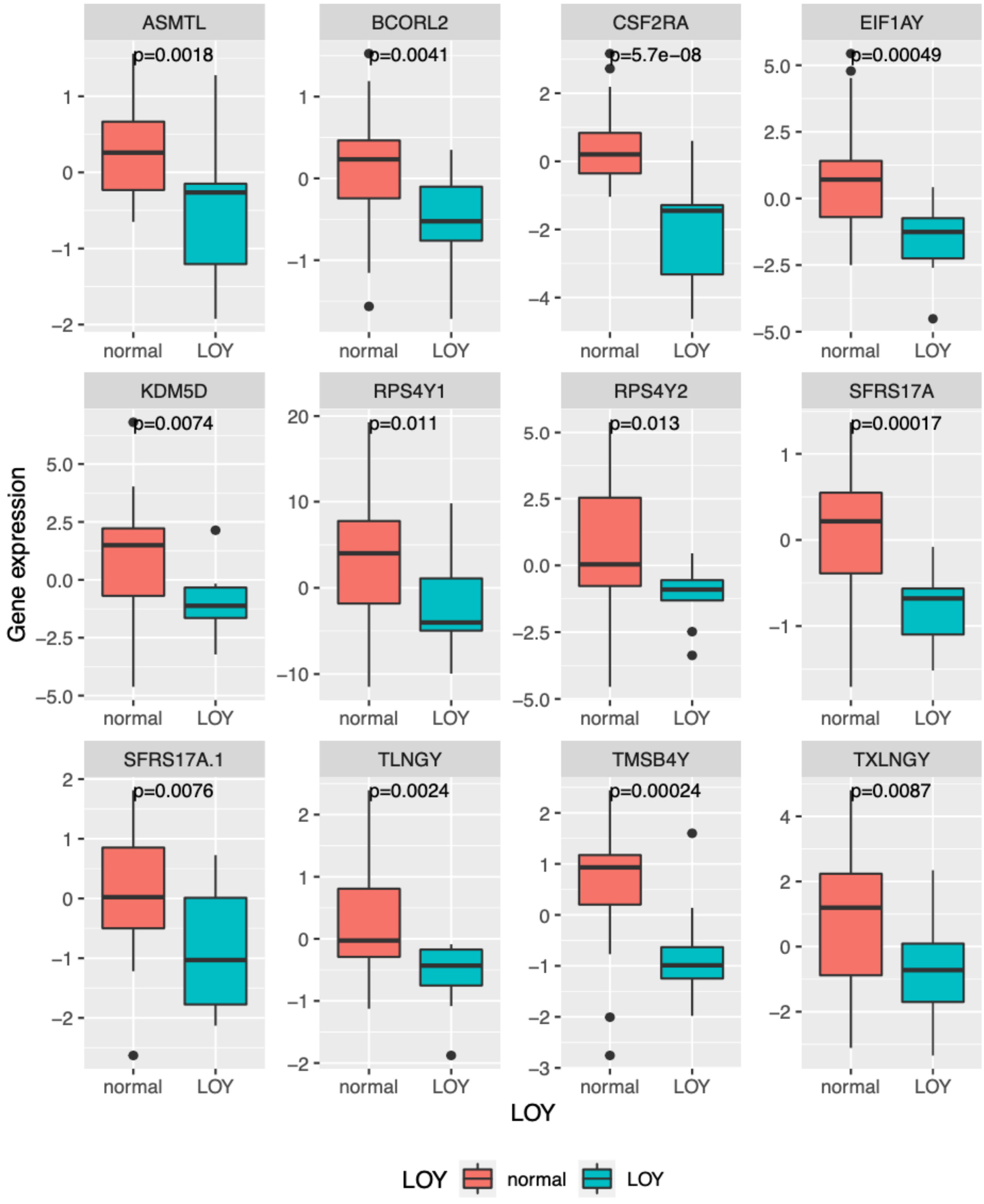

Figure S3: Top differentially expressed chromosome Y genes between individual with LOY and controls. The plots show the gene expression for individuals with (LOY) and without LOY (normal). The p-values correspond to a linear model adjusted for age and surrogate variables using limma
