# Supplementary material for: Clonal chromosomal mosaicism and loss of chromosome Y in elderly men increase vulnerability for SARS-CoV-2
Source: Commun Biol. 2024 Feb 19;7:202. doi: 10.1038/s42003-024-05805-6 (PMC10876565; doi:10.1038/s42003-024-05805-6)
Supplement: Supplementary file 3 — Description of Additional Supplementary Files [file 42003_2024_5805_MOESM3_ESM.pdf]

Description of Additional Supplementary Files

File name: Supplementary Data 1
Description: Patients with mCAs.

File name: Supplementary Data 2
Description: Patients with LOY/XCM.

File name: Supplementary Data 3
Description: Individuals with germinal line.

File name: Supplementary Data 4
Description: Genes located on the human Y chromosome with homologs on X and a possible role in immunity.

File name: Supplementary Data 5
Description: Additional genes located on the human Y chromosome with homologs on X and no known role in immunity.

File name: Supplementary Data 6
Description: Top 30 differentially expressed genes in blood of individuals with mCAs.
