# Supplementary material for: Clonal chromosomal mosaicism and loss of chromosome Y in elderly men increase vulnerability for SARS-CoV-2
Source: Commun Biol. 2024 Feb 19;7:202. doi: 10.1038/s42003-024-05805-6 (PMC10876565; doi:10.1038/s42003-024-05805-6)
Supplement: Supplementary file 5 — Reporting Summary [file 42003_2024_5805_MOESM5_ESM.pdf]

nature portfolio

Corresponding author(s): Luis A Perez-Jurado

Last updated by author(s): 25/02/2022

Reporting Summary

Nature Portfolio wishes to improve the reproducibility of the work that we publish. This form provides structure for consistency and transparency in reporting. For further information on Nature Portfolio policies, see our Editorial Policies and the Editorial Policy Checklist.

Statistics

For all statistical analyses, confirm that the following items are present in the figure legend, table legend, main text, or Methods section.

n/a	Confirmed	
☐	☒	The exact sample size (*n*) for each experimental group/condition, given as a discrete number and unit of measurement
☐	☒	A statement on whether measurements were taken from distinct samples or whether the same sample was measured repeatedly
☐	☒	The statistical test(s) used AND whether they are one- or two-sided<br>*Only common tests should be described solely by name; describe more complex techniques in the Methods section.*
☐	☒	A description of all covariates tested
☐	☒	A description of any assumptions or corrections, such as tests of normality and adjustment for multiple comparisons
☐	☒	A full description of the statistical parameters including central tendency (e.g. means) or other basic estimates (e.g. regression coefficient) AND variation (e.g. standard deviation) or associated estimates of uncertainty (e.g. confidence intervals)
☐	☒	For null hypothesis testing, the test statistic (e.g. *F*, *t*, *r*) with confidence intervals, effect sizes, degrees of freedom and *P* value noted<br>*Give P values as exact values whenever suitable.*
☒	☐	For Bayesian analysis, information on the choice of priors and Markov chain Monte Carlo settings
☒	☐	For hierarchical and complex designs, identification of the appropriate level for tests and full reporting of outcomes
☒	☐	Estimates of effect sizes (e.g. Cohen's *d*, Pearson's *r*), indicating how they were calculated

Our web collection on statistics for biologists contains articles on many of the points above.

Software and code

Policy information about availability of computer code

Data collection	Transcriptomic data was obtained from GEO.
Data analysis	R Version 3.5.2; Bioconductor Version 3.8; MADLoy (R-Package) Version 1.01.

For manuscripts utilizing custom algorithms or software that are central to the research but not yet described in published literature, software must be made available to editors and reviewers. We strongly encourage code deposition in a community repository (e.g. GitHub). See the Nature Portfolio guidelines for submitting code & software for further information.

Data

Policy information about availability of data

All manuscripts must include a data availability statement. This statement should provide the following information, where applicable:

- Accession codes, unique identifiers, or web links for publicly available datasets
- A description of any restrictions on data availability
- For clinical datasets or third party data, please ensure that the statement adheres to our policy

LOY and CME status obtained from genomic data are provided in Tables S1-S2. Any other relevant data relating the SCOURE project are available upon reasonable request. Transcriptomic data from EGCUT individuals are available at GEO repository.

Field-specific reporting

Please select the one below that is the best fit for your research. If you are not sure, read the appropriate sections before making your selection.

☒ Life sciences ☐ Behavioural & social sciences ☐ Ecological, evolutionary & environmental sciences

For a reference copy of the document with all sections, see nature.com/documents/nr-reporting-summary-flat.pdf

Life sciences study design

All studies must disclose on these points even when the disclosure is negative.

Sample size	9578
Data exclusions	2 individuals with no clinical data.
Replication	This is a large discovery multicentre study.
Randomization	Patients from the SCOURGE consortia (34 centres in 25 cities) with COVID19 diagnosis, clinical and genotype data were included in this study.
Blinding	The authors of the manuscript were not involved in data collection.

Reporting for specific materials, systems and methods

We require information from authors about some types of materials, experimental systems and methods used in many studies. Here, indicate whether each material, system or method listed is relevant to your study. If you are not sure if a list item applies to your research, read the appropriate section before selecting a response.

Materials & experimental systems

n/a	Involved in the study	
☒	☐	Antibodies
☒	☐	Eukaryotic cell lines
☒	☐	Palaeontology and archaeology
☒	☐	Animals and other organisms
☐	☒	Human research participants
☒	☐	Clinical data
☒	☐	Dual use research of concern

Methods

n/a	Involved in the study	
☒	☐	ChIP-seq
☒	☐	Flow cytometry
☒	☐	MRI-based neuroimaging

Human research participants

Policy information about studies involving human research participants

Population characteristics	In Spain, 11,939 COVID-19 positive cases were recruited as part of SCOURGE study from 34 centres in 25 cities.
Recruitment	Study samples and data were collected by the participating centers, through their respective biobanks after informed consent, with the approval of the respective Ethic and Scientific Committees.
Ethics oversight	The whole project was approved by the Galician Ethical Committee Ref 2020/197

Note that full information on the approval of the study protocol must also be provided in the manuscript.
